# Crystal Structure and NMR of an α,δ‐Peptide Foldamer Helix Shows Side‐Chains are Well Placed for Bifunctional Catalysis: Application as a Minimalist Aldolase Mimic**

**DOI:** 10.1002/ange.202305326

**Published:** 2023-06-14

**Authors:** Qi Lin, Hao Lan, Chunmiao Ma, Ryan T. Stendall, Kenneth Shankland, Rebecca A. Musgrave, Peter N. Horton, Carsten Baldauf, Hans‐Jörg Hofmann, Craig P. Butts, Manuel M. Müller, Alexander J. A. Cobb

**Affiliations:** ^1^ Department of Chemistry King's College London 7 Trinity Street London SE1 1DB UK; ^2^ School of Chemistry University of Bristol Cantocks Close Bristol BS8 1TS UK; ^3^ School of Chemistry and Chemical Engineering Huazhong University of Science and Technology Wuhan 430074 P. R. China; ^4^ School of Chemistry Food and Pharmacy (SCFP) University of Reading Whiteknights Berks Reading RG6 6AD UK; ^5^ EPSRC National Crystallography Service School of Chemistry University of Southampton Highfield Southampton SO17 1BJ UK; ^6^ Fritz-Haber-Institut der Max-Planck-Gesellschaft Faradayweg 4–6 14195 Berlin Germany; ^7^ Institut für Biochemie Universität Leipzig Brüderstrasse 34 04103 Leipzig Germany

**Keywords:** catalysis, peptidic foldamers, peptides, aldolase, helical conformation

## Abstract

We report the first NMR and X‐ray diffraction (XRD) structures of an unusual 13/11‐helix (alternating i, i+1 {NH−O=C} and i, i+3 {C=O−H−N} H‐bonds) formed by a heteromeric 1 : 1 sequence of α‐ and δ‐amino acids, and demonstrate the application of this framework towards catalysis. Whilst intramolecular hydrogen bonds (IMHBs) are the clear driver of helix formation in this system, we also observe an apolar interaction between the ethyl residue of one δ‐amino acid and the cyclohexyl group of the next δ‐residue in the sequence that seems to stabilize one type of helix over another. To the best of our knowledge this type of additional stabilization leading to a specific helical preference has not been observed before. Critically, the helix type realized places the α‐residue functionalities in positions proximal enough to engage in bifunctional catalysis as demonstrated in the application of our system as a minimalist aldolase mimic.

## Graphical abstract

A new catalytic foldamer constructed of α‐ and bespoke δ‐residues forms a helix which is not only stabilized by intramolecular H‐bonds, but also by apolar interactions not observed in foldamer secondary structures until now. The resulting scaffold gives a useful juxtaposition of functionality and leads to bifunctional catalysis as demonstrated through an aldolase‐like process.

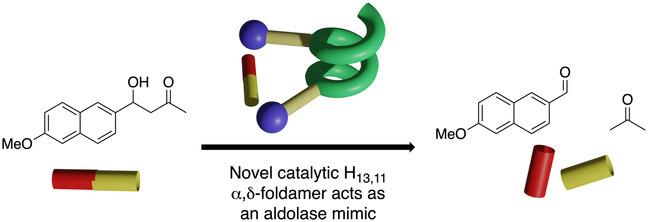

## Introduction

The folding of linear molecular species within biology into more complex architectures is critical in dictating their overall function as it results in the presentation of functionality in a very precise way within three‐dimensional space. This complex relationship between structure and function has inspired chemists to design their own minimalistic scaffolds, known as foldamers,^[1]^ with the purpose of uncovering functions that are inextricably linked to this higher order structure. These goals are diverse and range from peptidomimetics^[2]^ through to molecular recognition systems^[3]^ and catalysis, the ultimate focus of this report.^[4]^ Understandably, owing to the elegance with which nature can combine a relatively simple set of just twenty amino acids and fashion them towards a vast range of structures and functionalities, a great deal of focus has been towards novel peptidic foldamers. This ranges from peptoids^[5]^ through to homologated amino acids—particularly β‐,^[6]^ and γ‐systems.^[7]^ These unnatural sequences have advantages over naturally occurring α‐peptides in that the secondary structures formed are more predictable, and—critically for any therapeutic application—resistant to metabolism and proteolysis.

**Figure 1:**
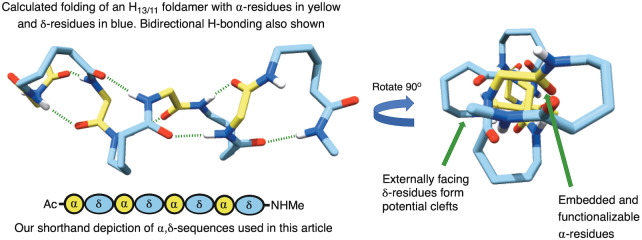
The most stable theoretical 13/11‐helix with embedded α‐residues, proposed by Hofmann and co‐workers in reference [9], including the shorthand blue and yellow symbols we use in this article.

Foldamer catalysis is itself a relatively new area, with one of the first examples coming from the groups of Gellman and Hilvert in 2009.^[8]^ Inspired by both this and the recent report of Gellman into the use of foldamers within catalysis,^[4]^ where we noticed a dearth of systems that could be easily modified, we sought to identify a scaffold that would not only present catalytic functionality at the appropriate positions but could also be easily adapted. In our view, this would give a central scaffold that could be applied to a wide range of processes depending on the functionality incorporated. In that regard, the most effective way to achieve this would be to use α‐amino acids within our sequence, with the immense variety of functionality that this would allow us to access. Of particular interest to us, therefore, were the peptidic 1 : 1 heteromeric sequences of α‐ and homologated amino acids. First modelled by Hofmann and co‐workers, these systems can form a diverse array of novel helices as reported in a series of in silico papers.[^9^, ^10^] Such helices are described as being “mixed”—and have the potential for hydrogen bonds to alternately point into forward and backward directions along the sequence, in contrast to helices with unidirectional hydrogen bonds. Among the various possible 1 : 1 heteromers of α‐amino acids with higher homologated amino acids, α,β‐ and α,γ‐hybrid peptides predominate the literature^[11]^—for example by the groups of Gellman,[^12^, ^13^] Sharma,[^9c^, ^9h^, ^14^] Gopi,[^9a^, ^9b^, ^15^] Tomasini,^[16]^ and recently our own where we described a highly stable 10/12‐helix made from a *cis*‐cyclopentyl‐γ‐amino acid.^[17]^ However, despite their broad predicted range of helices, the corresponding δ‐hybrid systems proposed by Hofmann and co‐workers,^[10]^ have recieved far less attention—with the only examples coming from the same report, and from Gervay‐Hague and co‐workers.^[18]^ This shortfall is partly due to the paucity in synthetic methods towards the appropriate δ‐amino acid monomers.^[19]^ Furthermore, these hybrid systems are of potential biological interest, because an α,δ‐dipeptide corresponds to an α‐amino acid trimer unit and has the potential to replace this in peptides and proteins. Nevertheless, with our eye on the catalytic potential we desired, we were drawn to the most stable of these δ‐amino acid heteromers—the H_13/11_ system—because according to Hofmann‘s modelling, the α‐residues appear to be *embedded* within the helix, whilst the δ‐residues occupy the perimeter (Figure 1).^[9]^ We anticipated that this arrangement would provide a highly defined binding pocket that could be exploited for catalysis, whereby the α‐residues might provide the functionality and the δ‐residues the steric environment.

## Results and Discussion

**Figure 2:**
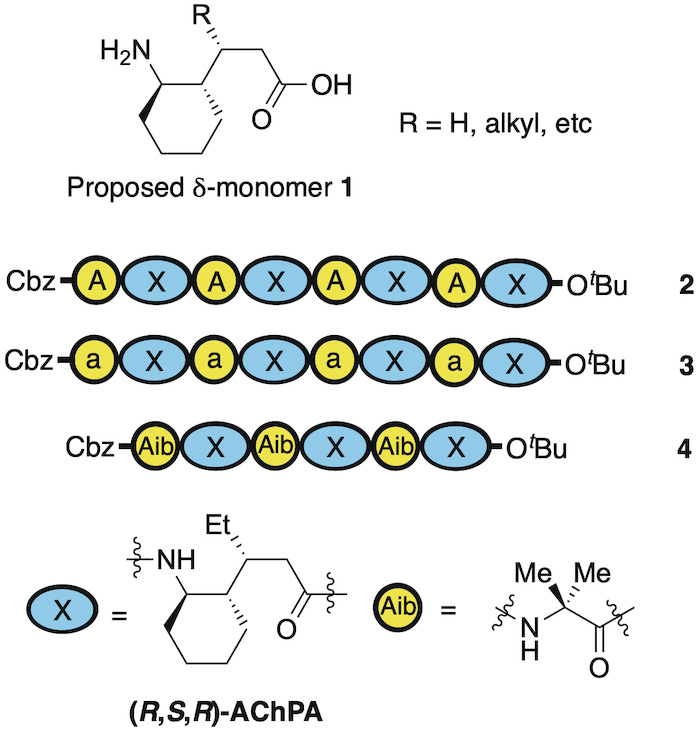
Sequences for initial oligomeric α,δ‐peptides **2**, **3** and **4** using either l‐Ala (A), d‐ala (a) or Aib as the α‐residue, and X as the δ‐residue.

To assess our hypothesis, we first needed to identify a δ‐residue that would be capable of adopting the required conformation to form this predicted 13/11‐helix. According to our minimization studies, we felt that cyclic δ‐residue **1** (Figure 2) would satisfy these conditions (See Supporting Information, Section 4.1). This residue could be accessed from the corresponding γ‐amino acid previously described by our group,^[20]^ as well as Gellman and others.[^13^, ^21^] Owing to the scalability of Gellman's method, we chose this for our studies to generate the δ‐subunit (*R*,*S*,*R*)‐AchPA which we denote as “X” in the sequences of Figure 2. Our first efforts involved the incorporation of this into 1 : 1‐heteromeric oligomer sequences using either l‐alanine, d‐alanine, or Aib as the α‐unit leading to sequences **2**, **3** and **4** respectively (see Supporting Information for details of solution phase syntheses).

Of these three sequences, only octamer **2** showed the characteristic downfield shift in multiple NH signals indicative of intramolecular H‐bonding (IMHB, see Figures S1–S3), which were also confirmed with temperature coefficients from VT experiments in chloroform. The other sequences failed to show this, demonstrating the importance of the stereocenter in the α‐residue for helix formation.

**Figure 3:**
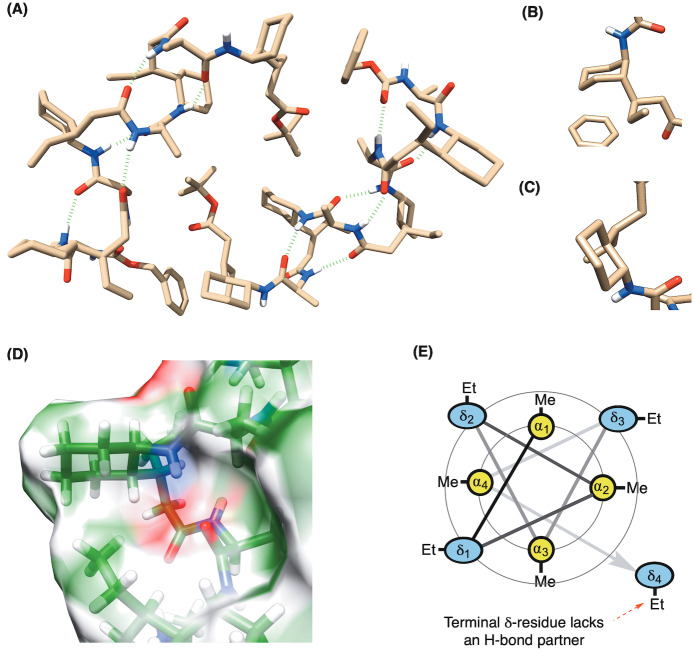
A) Crystal structure of foldamer **2** showing an unusual 13/11‐helix, where two packing conformations exist in the unit cell (CCDC: 2252028—see Supporting Information for ellipsoid plots)—differing only in the terminal δ‐residue which adopts either B) (*a*,*a*) or C) (*e*,*e*)‐conformations. D) Surface plot showing the ethyl‐group of one δ‐residue pointing towards the cyclohexyl unit of the (i+2) δ‐system. E) Wheel diagram representation of the octamer showing the interior α‐residues and the exterior facing δ‐residues, along with the terminal residue that lacks an H‐bond partner.

To ascertain whether the observed hydrogen bonding of these NH signals resulted in a defined secondary structure, we first successfully obtained a single crystal diffraction of octamer **2** (Figure 3A). Remarkably this resulted in three interesting observations.

First, it made clear why the stereogenicity of the α‐amino acid is crucial, as if it is epimeric at that center, it points *into* the part of the helix where H‐bonds occur, thus preventing their formation. Second, that there are two crystallographically‐independent molecules of octamer **2** within the unit cell. Both molecules show the characteristic hydrogen bonding pattern for a 13/11‐helix, but differ in the placement of the terminal δ‐residue, which adopts either an (*a*,*a*) or (*e*,*e*) conformation (Figures 3B and C respectively). The underlying reason for this is that the terminal δ‐residue lacks a hydrogen bonding partner. Its conformational flexibility, therefore, increases and makes the two structural alternatives possible. According to crystallography, the diaxial conformer may additionally be stabilized by an apolar interaction between the ethyl group of the terminal δ‐residue and the cyclohexyl residue of the nearest δ‐residue at (i+2). Whilst this interaction is missing at the terminal (*e*,*e*)‐conformer in the crystal, it is prevalent between the other corresponding residues throughout both helices (Figure 3D) as we discuss in more detail later. The structure also confirms the arrangement of interior lying α‐residues and exterior facing δ‐residues as represented by the wheel diagram (Figure 3E).

The third point of interest arising from these structures is that the 13/11‐helix realized in octamer **2** is not the most stable predicted by Hofmann for the unsubstituted backbone by theory (denoted H_13/11_^I^),^[10]^ but is actually the *second* most stable (denoted H_13/11_^II^) according to DFT (Density Functional Theory) calculations at the B3LYP/6‐31G* level. We believe the aforementioned apolar interactions between the ethyl and cyclohexyl groups, are partially responsible for this change in helix preference, because although they are possible in both H_13/11_^I^ and H_13/11_^II^, the stabilization conferred by it is greater for the latter. This conclusion comes from our investigation into the stability relationships of these helices, where we undertook in silico studies of the octamers of *both* helix types by the step‐wise addition of the substituents of our δ‐amino acid constituents.^[22]^

Starting with *unsubstituted* δ‐amino acid constituents, and l‐Ala as the α‐residue, H_13,11_^I^ remains the preferred helix over H_13,11_^II^ by 21.8 kJ mol^−1^ as described in Hofmann's report. However, introduction of a β‐ethyl substitution in the δ‐amino acid constituents changes the stability such that H_13/11_^II^ is now favored over H_13/11_^I^ by 26.4 kJ mol^−1^. Introduction of the cyclohexane substituent without this β‐ethyl group still favors H_13/11_^II^, but only by 5.4 kJ mol^−1^ this time. It is only when we introduce *both* ethyl and cyclohexyl residues that the oligomer shows a huge preference for H_13/11_^II^ over H_13/11_^I^ by 60.2 kJ mol^−1^ indicating the stabilizing importance of these side chain interactions. i.e. if you remove one or other partner from this apolar interaction, the preference for the 13/11(II) helix becomes far less pronounced. Thus the preference of one helix type over the other is essentially a matter of balance between an intrinsic factor of helix stability, which seems to favor helix type‐I, and the apolar side chain interactions which change the stability towards helix type‐II (see Table S2). Whilst it is known (specifically in β‐peptides) that side‐chains can dictate a helix type,^[6k]^ this is due to a different pattern of hydrogen‐bonding being enforced, rather than a distinct stabilizing apolar interaction as observed in our system.

**Figure 4:**
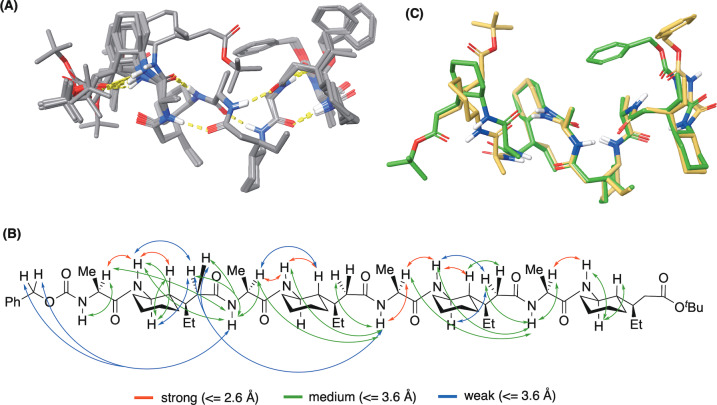
A) Unrestrained Monte‐Carlo Multiple minimization (MCMM), followed by clustering and abundant DFT energy minimizations (ωB97M‐D3BJ/def2‐TZVP// ωB97XD/6‐31G*) on hundreds of distinct conformations including both helical and non‐helical scaffolds, gave 13 low‐energy geometries. B) Experimental restraints and DFT‐calculated values show good correlation, where mean absolute error (MAE) for all strong and medium ROEs <3.6 Å is below 8 % C) Overlay of lowest energy solution structure (green) and the XRD (beige) of the (*e*,*e*)‐conformer shows close correlation.

In order to understand the conformation in the *solution* state, we undertook further extensive modelling and NMR studies. To begin with, the observation of both (*a*,*a*) and (*e*,*e*)‐conformations of the terminal δ‐residue that appear in the unit cell of the XRD was re‐investigated. Unsurprisingly, the (*e*,*e*) was observed as the major state of **2** in solution, confirmed by a large ^3^ *J*_HH_‐coupling (10.3 Hz) of the vicinal protons on the substituted cyclohexyl ring at the terminus.^[23]^ Then, using unrestrained Monte‐Carlo Multiple minimization (MCMM), followed by clustering and abundant DFT energy minimizations (ωB97M‐D3BJ/def2‐TZVP//ωB97XD/6‐31G*) on hundreds of distinct conformations including both helical and non‐helical scaffolds,^[24]^ 13 geometries were afforded as low energy conformers of **2** under solution states (15 kJ mol^−1^ threshold, Figure 4A and Supporting Information). The computational ensembles of these 13 low‐energy geometries in solution also suggested the (*e*,*e*)‐helix are predominant whereas the (*a*,*a*)‐helix were calculated to be less stable according to DFT energies (>30 kJ mol^−1^).

We then validated the solution ensembles of these 13 low‐energy snapshots from unrestrained MCMM–DFT modelling with 37 H‐H ROE restraints collected from ROESY (Rotating frame NOESY) experiment (Figure 4D and Supporting Information).[^25^, ^26^] Their close alignment suggests that the core helix itself is highly stable as indicated from modelling. A satisfactory consistency was achieved between experimental restraints and DFT‐calculated values, where mean absolute error (MAE) for all strong and medium ROEs (<3.6 Å, Figure 4B) is below 8 %. This includes the fitting of several cross‐residue correlations under IMHBs for helicity. Moreover, we also observed two weak NOEs between the ethyl and cyclohexane moieties on different delta residues, attributed to the apolar interactions we described above (see Supporting Information, Section 7.0).

Meanwhile extractable ^3^ *J*_HH_ coupling constants along the foldamer backbone were also reproduced using the Gauge‐Independent Atomic Orbital (GIAO) method based on DFT minimized ensembles (see Supporting Information), again supporting the distributions of the corresponding dihedral angles. No obvious violation of torsional regularity was observed for the core helix. Although the calculated ^3^ *J*_HH_ for the vicinal protons on δ_4_ cyclohexane (11.0 Hz) approach the value observed from experiment (10.3 Hz), larger errors were identified for other ^3^ *J*_HH_ on the terminal δ_4_ residue. We hypothesized a high flexibility of such regions in solution, of which detailed dynamics can be hardly covered by modelling and NMR restraints.

To validate the distribution of IMHBs for foldamer **2**, we also investigated temperature coefficients on all acidic protons in solution. The VT study in chloroform (from 298.15 K to 323.15 K) suggested the carbamate NH proton has a unique low value (0.6 ppb/T) compared to all other amide NH protons (>1.9 ppb/T). This was consistent with modelling which shows the carbamate NH as the only solvent exposed acidic proton in low‐energy ensembles. During the heating of solution towards higher temperatures, thermodynamic exchange between the stable hydrogen‐bonding state and other non‐bonding states may be allowed. This probably leads to a significant decrease in the conformationally average chemical shift for all amide protons which were originally de‐shielded in IMHBs at room temperature.^[27]^ So whilst we can say that the helical nature is not lost under heating according to our NMR analysis of temperature effects, it is likely that there are more non‐helical states being populated. For the chemical shift of carbamate NH, much less variation was observed along the increase of temperature as a likely result of constant exposure to solvent (See Supporting Information, Section 7). We then aligned the lowest energy structure from NMR‐verified MCMM–DFT modelling (4C, green) with the (*e*,*e*)‐crystal structure (Figure 4C, beige). Only slight differences‐ occuring at the two termini were observed which are: (1) The benzyl moiety in crystallography showed hydrophobic contact with the ethyl side chain on the N‐terminal δ‐residue, whilst the solution‐state model suggested contact with the methyl side chain of the N‐terminal alanine. (2) An extra IMHB was formed within C‐terminal delta residue according to our NMR snapshot (large temperature coefficient in chloroform). Nevertheless, the helicity of the core region is highly preserved and almost identical in both states, providing a scaffold that can predictably orient functional groups for diverse applications in catalysis and molecular recognition, with our immediate interests being on the former.

**Scheme 1:**
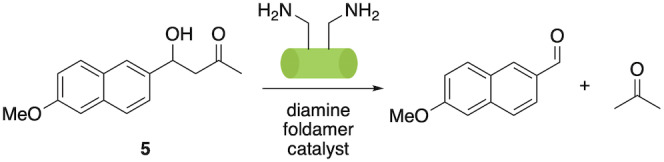
Aldolase‐like foldamer catalyzed retroaldol reaction of methodol **5**.

To test this possibility and inspired by the seminal work of Gellman and Hilvert, who first demonstrated the application of foldamers in catalysis via a retroaldol process,^[8]^ we installed two l‐α‐Orn residues featuring in i and i+2 positions. We anticipated that this arrangement of primary amine functional groups on one face of the foldamer helix would provide a bifunctional system capable of achieving the same reaction through nucleophilic catalysis via a bifunctional imine/enamine mechanism (Scheme 1). As a substrate, we chose methodol (4‐hydroxy‐4‐(6‐methoxy‐2‐naphthyl)‐2‐butanone) **5**, which is frequently used to benchmark artificial aldolases.^[28]^

**Figure 5:**
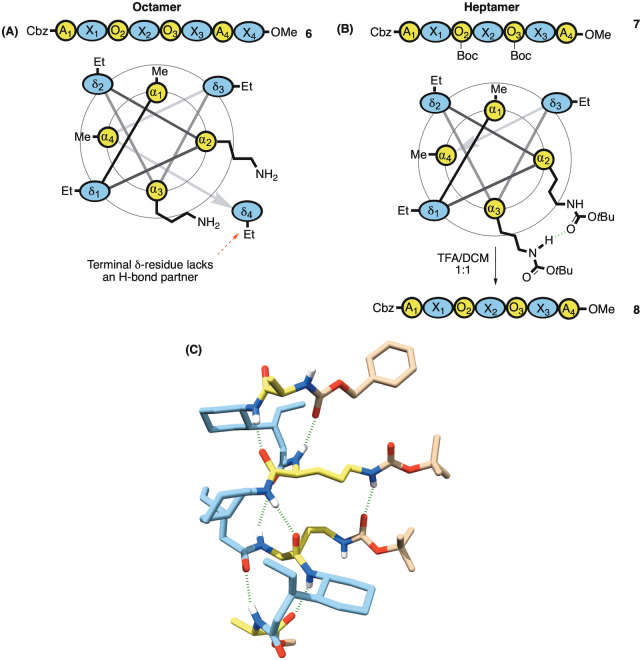
A) Bis‐ornithine octamer with wheel diagram showing the terminal δ‐residue lacking an H‐bond partner B) Heptamer **7** also with wheel diagram (lacking the labile terminal residue) with two of the α‐residues replaced by protected ornithines, which on deprotection gives diamine **8**. C) Single crystal XRD (CCDC: 2252029) of the protected bis‐ornithine shows an H‐bond interaction between carbamates along with the same apolar interaction.

**Figure 6:**
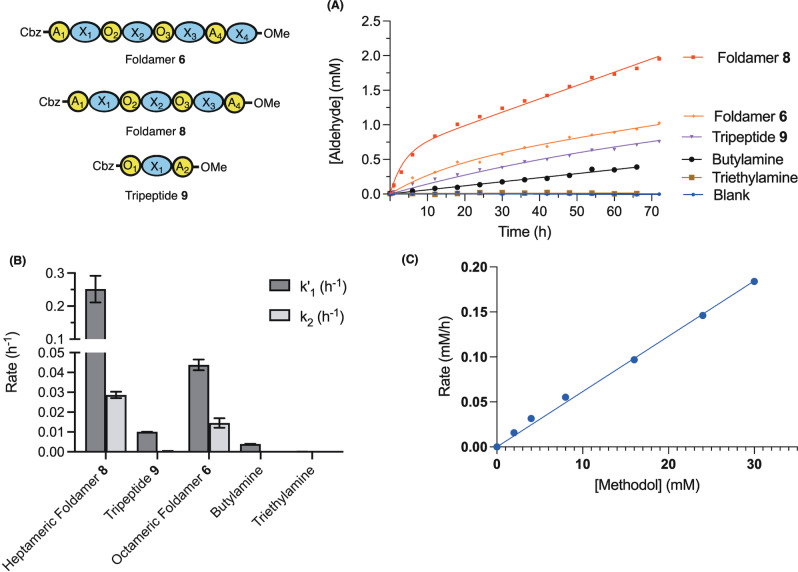
A) Action of different catalysts (at 1.5 mM total amine) on methodol (15 mM) at 20 °C in CDCl_3_ using ^1^H NMR. Sequences **6**, **8** and **9** are fit to burst kinetics, Et_3_N and BuNH_2_ to a linear model. The data points for heptamer **8** correspond to the average from two independent measurements. B) Reaction rates obtained from the fit in (A)—error bars depict the error of the fit. C) Dependence of velocity on [methodol] (2–30 mM) using foldamer **8** at 20 °C in CHCl_3_ monitored by HPLC.

We therefore synthesized the octameric diamine **6** (Figure 5A), as well as heptameric diamine **8** accessed via its protected intermediate **7** (Figure 5B). We reasoned that the conformational freedom of the terminal δ‐residue of the octamer might lead to some unpredictability with regards to application. Gratifyingly, we also managed to obtain the crystal structure of heptamer **7** and to our delight it showed a hydrogen bond between the NH proton of one carbamate and the carbonyl of the other across the helix pitch (Figure 5C).[^29^, ^30^] This further encouraged our prediction that these functionalities were proximal enough to engage in bifunctional catalysis. With foldamers **6** and **8** in hand, we proceeded to measure their rate acceleration of the retro‐aldol reaction in chloroform (Figure 6).

Foldamer **8** proved to be the best catalyst, with a burst‐phase rate *k*_1′_=0.25±0.04 h^−1^ (15 mM methodol, 0.75 mM foldamer) and a steady state rate *k*_2_=0.029±0.002 h^−1^. This observation suggests that hydrolysis of the foldamer‐acetone imine intermediate is rate limiting under the conditions of the assay. Experiments whereby we have trapped iminium ion intermediates by reduction with sodium acetoxyborohydride confirmed the presence of imines resulting from the reduction of both condensed starting material and acetone (see Figure S7) and are thus compatible with a nucleophilic mechanism.

In comparison, the rate of the reaction in the presence of either 1.5 mM butylamine, α,δ‐tripeptide **9** or the tertiary amine triethylamine was considerably lower (Figure 6A,B), demonstrating the importance of the juxtaposition of amines for the catalytic activity of Foldamer **8**. The extended octameric foldamer **6** with the additional δ‐amino acid exhibited a lower activity than the heptamer **8** (*k*_1′_=0.044±0.03 h^−1^ and *k*_2_=0.015±0.002 h^−1^, Figures 6A amd B). It is possible that this is due to either the aforementioned inability of this residue to participate in helix formation, or that the additional bulk from this building block inhibits substrate access to the catalytic amines. Under the conditions tested, the rate of retroaldol cleavage using **8** was linearly dependent on the concentration of Methodol (Figure 6C).

These observations demonstrate that the foldamers are conducive to arranging multiple functional groups in a catalytically competent manner.

## Conclusion

In conclusion, we have uncovered a new foldamer structure in α,δ‐hybrid peptides—the 13/11(II)‐helix which was confirmed by both XRD and NMR. These structures are closely aligned, save for the terminal residues, and our studies demonstrate that the helix is very stable. We also show that apolar interactions observed between the ethyl group of one δ‐residue and the cyclohexyl system of the (i+2) δ‐residue are responsible for the stabilization of the novel helix type H_13/11_^II^ over H_13/11_^I^ which was theoretically predicted as most stable for an unsubstitued backbone, by +60.2 kJ mol^−1^. We then demonstrate that replacing two proximal α‐residues of the sequence with ornithines leads to a catalyst that is a mimic of an aldolase and accelerates the retroaldol reaction of methodol. The simplicity of the design process for endowing these α,δ‐foldamer scaffolds with distinct reactive groups and their unique structure—featuring a deep helical groove—augur well for the development of functional α,δ‐foldamers. Further catalyst application and refinement will be the subject of future reports.

## Supporting Information

Experimental procedures, spectroscopic data, copies of NMR spectra for all compounds, including X‐ray structure reports for **2**, **7** and the heptameric foldamer ZAXAXAXAOMe.

## Conflict of interest

The authors declare no conflict of interest.

1

## Supporting information

As a service to our authors and readers, this journal provides supporting information supplied by the authors. Such materials are peer reviewed and may be re‐organized for online delivery, but are not copy‐edited or typeset. Technical support issues arising from supporting information (other than missing files) should be addressed to the authors.

Supporting Information

Supporting Information

Supporting Information

Supporting Information

## Data Availability

The data that support the findings of this study are openly available in ChemRxiv at https://doi.org/10.26434/chemrxiv‐2023‐ks4t3.
