# Supplementary material for: Crystal Structure and NMR of an α,δ‐Peptide Foldamer Helix Shows Side‐Chains are Well Placed for Bifunctional Catalysis: Application as a Minimalist Aldolase Mimic
Source: Angew Chem Weinheim Bergstr Ger. 2023 Jun 14;135(36):e202305326. doi: 10.1002/ange.202305326 (PMC10952562; doi:10.1002/ange.202305326)
Supplement: Supplementary file 4 — Supporting Information [file ANGE-135-0-s004.pdf]

Angewandte Chemie
Eine Zeitschrift der Gesellschaft Deutscher Chemiker

Supporting Information

Crystal Structure and NMR of an α,δ-Peptide Foldamer Helix Shows Side-Chains are Well Placed for Bifunctional Catalysis: Application as a Minimalist Aldolase Mimic

*Q. Lin, H. Lan, C. Ma, R. T. Stendall, K. Shankland, R. A. Musgrave, P. N. Horton, C. Baldauf, H.-J. Hofmann, C. P. Butts, M. M. Müller, A. J. A. Cobb**

Supporting Information

Contents

1.0 General Information 4

2.0 Preparation of δ-amino acid precursor 5

(S)-2-((1S,2S)-2-nitrocyclohexyl)butanal P1 5

(1*S*,2*S*)-1-((*S,E*)-1-methoxypent-1-en-3-yl)-2-nitrocyclohexane P2 5

(*R*)-3-((1*S*,2*S*)-2-nitrocyclohexyl)pentanal P3 6

(*R*)-3-((1*S*,2*S*)-2-nitrocyclohexyl)pentan-1-ol P4 6

(*R*)-3-((1*S*,2*R*)-2-nitrocyclohexyl)pentan-1-ol P5 6

tert-butyldimethyl(((*R*)-3-((1*S*,2*R*)-2-nitrocyclohexyl)pentyl)oxy)silane P6 6

(1*R*,2*S*)-2-((*R*)-1-((tert-butyldimethylsilyl)oxy)pentan-3-yl)cyclohexan-1-amine P7 7

2.1 Foldamer Synthesis 7

General procedure A: Peptide coupling 7

General procedure B: TBS-deprotecting. 8

General procedure C: Oxidizing alcohol to carboxylic acid. 8

General procedure D: Protecting carboxylic acid with tert-butyl ester. 8

General procedure E: Cbz-ester deprotection. 9

2.2 Synthesis of Non-Catalytic α,δ-Oligomers 9

Synthesis of Foldamer 2 10

Cbz-L-Ala-AChPA•(CH_2OTBS) P8 10

Cbz-L-Ala-AChPA•(CH_2OH) P9 10

Cbz-L-Ala-AChPA-OH P10 10

Cbz-L-Ala-AChPA-O^{t}Bu P11 10

Cbz-L-Ala-AChPA-L-Ala-ACPA-O^{t}Bu P12 11

Cbz-L-Ala-AChPA-L-Ala-ACPA-L-Ala-AChPA-O^{t}Bu P13 11

Cbz-L-Ala-AChPA-L-Ala-AChPA-L-Ala-AChPA-L-Ala-AChPA-O^{t}Bu 2 12

Synthesis of Octamer 3 12

Cbz-D-Ala-AChPA•(CH_2OTBS) P14 12

Cbz-D-Ala-AChPA•(CH_2OH) P15 13

Cbz-D-Ala-AChPA-OH P16 13

Cbz-D-Ala-AChPA-O^{t}Bu P17 13

Cbz-D-Ala-AChPA-D-Ala-AChPA-O^{t}Bu P18 14

Cbz-D-Ala-AChPA-D-Ala-AChPA-D-Ala-AChPA-O^{t}Bu P19 14

Cbz-D-Ala-AChPA-D-Ala-AChPA-D-Ala-AChPA-D-Ala-AChPA-O^{t}Bu 3 14

Synthesis of Hexamer 4 15

Cbz-Aib-AChPA•(CH_2OTBS) P20 15

Cbz-Aib-AChPA• (CH_2OH) P21 15

Cbz-Aib-AChPA-OH P22 15

Cbz-Aib-ACPA-O^{t}Bu P23 16

Cbz-Aib-AChPA-Aib-AChPA-O^{t}Bu P24 16

Cbz-Aib-AChPA-Aib-AChPA-Aib-AChPA-O^{t}Bu 4 16

2.3 Preparation of Catalytic Octamers 17

Synthesis of Catalytic α,δ-Foldamer 6 17

General procedure F: Boc-deprotection 17

Cbz-L-Ala-AChPA-OMe P31 18

Cbz-L-Orn•Boc-AChPA-L-Ala-AChPA-OMe P32 18

Cbz-L-Orn•Boc-AChPA-L-Orn•Boc-AChPA-L-Ala-AChPA-OMe P33 19

Cbz-L-Ala-AChPA-L-Orn•Boc-AChPA-L-Orn•Boc-AChPA-L-Ala-AChPA-OMe 19

Cbz-L-Ala-AChPA-L-Orn•NH_2-AChPA-L-Orn•NH_2-AChPA-L-Ala-AChPA-OMe 6 20

2.4 Preparation of non-catalytic/catalytic Heptamers 21

Synthesis of Heptamer Foldamer Z-A-X-A-X-A-X-A-OMe 22

Cbz-L-Ala-AchPA-L-Ala-Ome P25 22

Cbz-L-Ala-ACPA-L-Ala-ACPA-L-Ala-ACPA-L-Ala-Ome Z-A-X-A-X-A-X-A-Ome 22

Synthesis of Foldamer 7/Foldamer 8 23

Cbz-L-Orn•O^{t}Bu-AchPA-L-Orn•O^{t}Bu-AchPA-L-Ala-Ome 23

Cbz-L-Ala-AchPA-L-Orn•O^{t}Bu-AchPA-L-Orn•O^{t}Bu-AchPA-L-Ala-Ome 7 24

Cbz-L-Ala-AchPA-L-Orn•NH_2-AchPA-L-Orn•NH_2-AchPA-L-Ala-Ome 8 24

Synthesis of Tripeptide 9 26

Cbz-L-Boc•NH_2-ACPA-L-Ala-Ome 26

2.5 Characterization of non-catalytic foldamers 28

3.0 Retro-aldol cleavage in chloroform 30

4.0 Modelling Studies 33
4.1 Monomer Conformation Predictions 33
4.2 Density functional theory calculations 33
Comparison of the backbone torsion angles of the two monomers in the crystal cell arising from X-ray and NMR in solution with those of the two most stable 13/11-helices of α,δ-helices predicted by theory 34
Calculations on various 13/11-helices of α,δ-hybrid helices with varying backbone substitution 34
5.0 NMR spectra of all products 37
6.0 X-Ray Crystallography 63
7.0 Solution Conformational Analysis and Modelling of Foldamer 2 67
General Protocol of NMR Assignment and Analysis 67
NMR Assignment and Spectra ($CDCl_3$, 500 MHz) 68
7.1 General Protocol of Computational Modelling and NMR Calculations 86

1.0 General Information

Substrates: All starting materials were received from commercial suppliers unless otherwise stated. Anhydrous THF was supplied as Sureseal® bottles by Sigma Aldrich. All procedures were performed using dried solvents and reagents under an atmosphere of nitrogen with oven-dried glassware. Air and moisture-sensitive liquids/solutions were transferred to reaction vessels by syringe under an atmosphere of nitrogen. Agitation was achieved using Teflon coated stirrer bars by magnetic induction.

NMR data: Nuclear Magnetic Resonance (NMR) spectra were recorded either using a Bruker Ascend 400 (400 MHz) spectrometer (KCL) or a Bruker Cryo500/950 (Bristol). The ^{1}H NMR and ^{13}C NMR spectra were analysed with Mestrenova. All NMR characterisation experiments were performed at 25 °C and 1 atm unless otherwise specified. Multiplicity is reported as follows – s = singlet, d = doublet, t = triplet, q = quartet, m = multiplet. All spin-spin coupling constants (J) are reported in hertz (Hz) to the nearest 0.1 Hz.

MS data: High-resolution mass spectra were recorded on Waters Xevo G2-XS QTof Quadrupole Time-of-Flight Mass Spectrometer

Optical Rotation: Optical rotation readings were recorded using an Anton Parr 100 mm Polarimeter. Specific rotations are reported as ($[\alpha]_D$), and solution concentrations (c) are given in units of g/100 mL, temperatures are 25 °C.

HPLC: HPLC analysis was determined on Agilent Technologies with G7161BR 1290 Infinity II Sampler, G7112BR 1260 Infinity II Binary pump – up to 600 bar, G7116AR 1260 Infinity II MCT, G7115A 1260 Infinity II DAD.

Chromatography: Reactions were monitored by thin layer chromatography on silica gel precoated aluminium sheets (TLC Silica Gel 60, Merck). Visualisation was accomplished by potassium permanganate stain. Column chromatography was performed on Merck silica gel (60 ˚A, 230 - 400 mesh, 40 - 63 µm) or on a CombiflashRF+ system.

Single Crystal X-ray: X-ray data were collected on several diffractometers: Oxford Diffraction Gemini S-ultra (University College London); Rigaku Synergy (University of Reading); and Rigaku 007 HF four-circle (UK National Crystallography Service, University of Southampton). Data were diffracted using CuKα radiation, and data-collections performed at 100(2) K. Fuller details of individual data collections are given in the XRD Methodology section (Section X).

Circular Dichroism: All CD spectra were measured in an Applied Photophysics Circular Dichroism Spectrophotometer, Chirascan V100 using cuvettes of pathlength 1 cm (Hellma).

2.0 Preparation of δ-amino acid precursor

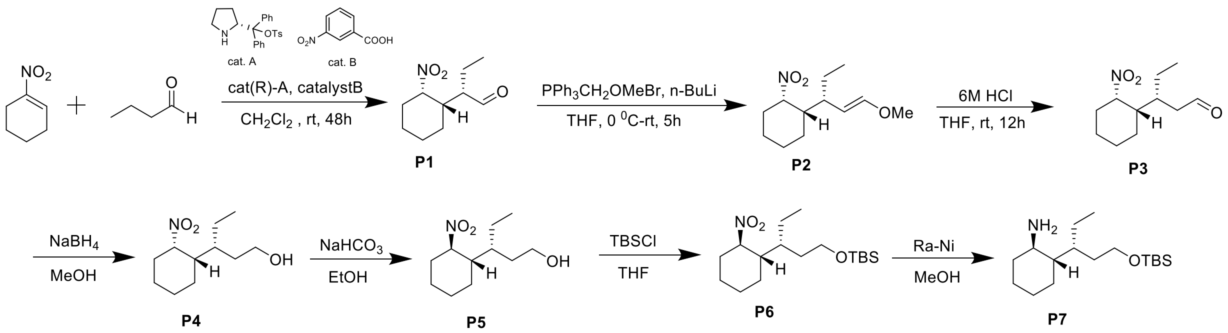

(S)-2-((1S,2S)-2-nitrocyclohexyl)butanal P1

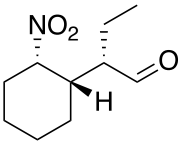

P1

To a 100mL round bottle flask was added catalyst A (1.276 g, 4.12 mmol), catalyst B (0.328 g, 1.96 mmol), 20 mL CH_2Cl_2, *n*-butanal (8.82 mL, 98.16 mmol), 1-Nitro1-cyclohexene (2.22 mL, 19.66 mmol), the mixture was stirred at room temperature for 2 days. Solvent was removed under reduced pressure and the crude reaction mixture was purified *via* column chromatography eluting with EtOAc/hexane to give the desired product as a pale-yellow oil in 59 % yield.[1] **^{1}H NMR (400 MHz, $CDCl_3$)** d 9.66 (d, J = 1.8 Hz, 1H), 4.86 (q, J = 3.6 Hz, 1H), 2.49 (dddd, J = 9.9, 8.0, 3.6, 1.8 Hz, 1H), 2.27 (dtd, J = 10.9, 2.9, 1.9 Hz, 1H), 2.12 (m, J = 10.0, 6.0, 4.0 Hz, 1H), 1.90 – 1.81 (m, 1H), 1.80 – 1.70 (m, 3H), 1.69 – 1.52 (m, 4H), 1.32 (m, 1H), 0.83 (t, J = 7.5 Hz, 3H). **^{13}C NMR (101 MHz, $CDCl_3$)** d 203.70, 83.61, 53.20, 37.34, 29.70, 26.77 – 18.01 (m), 9.98.

(1*S*,2*S*)-1-((*S*,*E*)-1-methoxypent-1-en-3-yl)-2-nitrocyclohexane P2

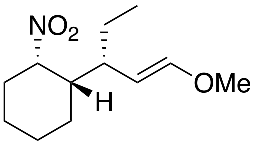

P2

Under a nitrogen atmosphere, (Methoxymethyl)triphenylphosphonium chloride (8.92 g, 26.02 mmol) was dissolved in anhydrous THF (40 mL) and the mixture was cooled down to 0 °C, *n*-BuLi (2.0 M in hexane, 9.50 mL, 23.12 mmol) was added dropwise to the solution. Then the solution was stirred for 45 minutes at 0 °C. **P1** (2.30 g, 11.56 mmol) was added to the mixture, and the solution was allowed to warm to room temperature and stirred for 4 hours. The reaction was quenched with saturated aqueous NH_4Cl solution, extracted with CH_2Cl_2, the organic layer was washed with aqueous $NaHCO_3$ and brine, dried over $MgSO_4$, filtered and concentrated, run through flash chromatography. The crude product was carried on without further purification.

(*R*)-3-((1*S*,2*S*)-2-nitrocyclohexyl)pentanal P3

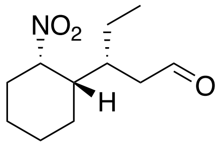

P3

HCl (6M, 5.5 mL) was added to the solution of **P2** (1.78 g, 7.82 mmol) in 40 mL THF, the solution was stirred at room temperature overnight. EtOAc was added then washed with brine three times, dried over $MgSO_4$, filtered and concentrated. The residue was purified via column chromatography eluting with EtOAc/hexane to give the mixture as a yellow oil. The crude product was carried on without further purification.

(*R*)-3-((1*S*,2*S*)-2-nitrocyclohexyl)pentan-1-ol P4

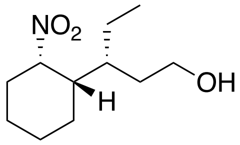

P4

To a stirred solution of **P3** (0.5 g, 2.34 mmol) in MeOH (30 mL) at 0 °C was added $NaBH_4$ (0.24 g, 7.03 mmol). The mixture was stirred for a few minutes. The mixture was slowly poured into a 200 mL beaker containing 50 mL 1M NH_4Cl at 0 °C, and the resulting mixture was extracted with EtOAc, the organic layers were collected, washed with brine, dried over $MgSO_4$ filtered and concentrated. The residue was purified via column chromatography eluting with EtOAc/hexane to give a colourless oil in 95% yield. **^{1}H NMR** (400 MHz, $CDCl_3$) d 4.89 (q, J = 3.5 Hz, 1H), 3.77 – 3.40 (m, 2H), 2.27 – 2.16 (m, 1H), 1.82 (dddd, J = 13.3, 8.0, 6.7, 4.0 Hz, 1H), 1.75 – 1.59 (m, 5H), 1.57 – 1.49 (m, 2H), 1.48 – 1.15 (m, 5H), 0.76 (t, J = 7.4 Hz, 3H). **^{13}C NMR** (101 MHz, $CDCl_3$) d 84.23, 60.82, 42.11, 37.13, 32.31, 30.84, 25.47, 23.56, 21.78, 20.18, 9.63. $[\alpha]^{25}_D$ +1.00 (*c* 1.00, $CHCl_3$).

(*R*)-3-((1*S*,2*R*)-2-nitrocyclohexyl)pentan-1-ol P5

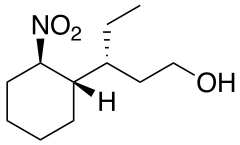

P5

To **P4** (0.51 g, 2.37 mmol) and $NaHCO_3$ (1.9 g, 23.70 mmol) was added 30 mL absolute ethanol, the mixture was refluxed for 3 hours. Then the mixture was filtered through filter paper and the filtrate was concentrated to give the desired product in 98 % yield. **^{1}H NMR** (400 MHz, $CDCl_3$) d 4.50 (td, J = 11.4, 4.1 Hz, 1H), 3.64 (t, J = 6.9 Hz, 2H), 2.25 (dddd, J = 12.0, 6.3, 3.0, 1.4 Hz, 1H), 2.04 (dddd, J = 13.0, 11.3, 3.5, 2.1 Hz, 1H), 1.93 – 1.83 (m, 2H), 1.82 – 1.74 (m, 2H), 1.67 (dtd, J = 13.9, 7.5, 4.2 Hz, 1H), 1.48 – 1.37 (m, 2H), 1.32 – 1.23 (m, 2H), 1.15 (dddd, J = 12.5, 9.4, 5.1, 2.6 Hz, 1H), 1.10 – 0.99 (m, 2H), 0.89 (t, J = 7.2 Hz, 3H). **^{13}C NMR** (101 MHz, $CDCl_3$) d 88.91, 61.29, 43.59, 37.01, 33.65, 32.63, 25.19, 24.71, 24.16, 22.59, 12.93. $[\alpha]^{25}_D$ -58.00 (*c* 1.00, $CHCl_3$).

***tert*-butyldimethyl(((*R*)-3-((1*S*,2*R*)-2-nitrocyclohexyl)pentyl)oxy)silane P6**

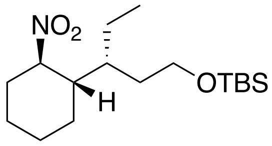

P6

To a solution of **P5** (0.51 g, 2.37 mmol) and imidazole (0.32 g, 4.74 mmol) in THF (20 mL) was added *tert*-butylchlorodimethylsilane (0.71g, 4.74 mmol) in THF (4 mL) at 0 °C. Cooling was removed after 30 min, after stirring overnight at rt, the saturated aqueous NH_4Cl (15 mL) was added, and the mixture was extracted with CH_2Cl_2, dried with $MgSO_4$ and concentrated, the crude product was

purified via column chromatography eluting with EtOAc/hexane to give the desired product in 73 % yield. **^{1}H NMR** (400 MHz, $CDCl_3$) d 4.49 (td, J = 11.3, 4.0 Hz, 1H), 3.57 (ddt, J = 10.2, 7.4, 3.6 Hz, 2H), 2.29 – 2.17 (m, 1H), 2.11 – 2.01 (m, 1H), 1.93 – 1.71 (m, 4H), 1.68 – 1.54 (m, 1H), 1.49 – 1.36 (m, 2H), 1.27 (tq, J = 9.0, 2.8 Hz, 2H), 1.14 (dddd, J = 14.5, 12.2, 6.1, 3.4 Hz, 1H), 1.09 – 0.96 (m, 2H), 0.89 (d, J = 2.4 Hz, 12H), 0.05 (s, 6H). **^{13}C NMR** (101 MHz, $CDCl_3$) d 88.83, 61.92, 43.99, 37.48, 34.05, 32.49, 26.08, 25.25, 24.72, 24.41, 22.54, 18.40, 12.95, -5.23. $[\alpha]^{25}_{D}$ -31.00 (*c* 1.00, $CHCl_3$).

(1*R*,2*S*)-2-((*R*)-1-((tert-butyldimethylsilyl)oxy)pentan-3-yl)cyclohexan-1-amine P7

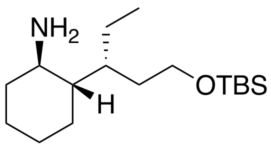

P7

P6 (0.55 g, 1.67 mmol) was dissolved in 25 mL MeOH, and the flask was flushed with N_2, then the Raney Nickel (1 g) was added, the mixture was stirred under a hydrogen balloon for 2 hours at room temperature. The reaction mixture was filtered through a pad of celite and concentrated to give a crude product in 93 % yield. **^{1}H NMR** (400 MHz, $CDCl_3$) δ 3.74 – 3.51 (m, 2H), 2.58 – 2.50 (m, 1H), 1.90 – 1.81 (m, 1H), 1.75 – 1.54 (m, 5H), 1.50 – 1.39 (m, 2H), 1.23 – 1.01 (m, 4H), 1.01 – 0.85 (m, 14H), 0.04 (s, 6H). **^{13}C NMR** (101 MHz, $CDCl_3$) δ 62.29, 51.59, 48.68, 37.39, 35.72, 34.47, 26.58, 26.12, 25.89, 25.08, 22.14, 18.45, 13.30, -5.10. **HRMS-ESI** (m/z) calc'd for $C_{17}H_{37}NOSi$ $[M+H]^+$, 300.2717; found, 300.2732. $[\alpha]^{25}_{D}$ -30.00 (*c* 1.00, $CHCl_3$).

2.1 Foldamer Synthesis

General procedure A: Peptide coupling.

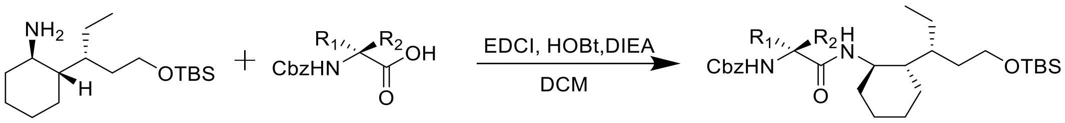

Carboxylic acid (1.60 mmol) was added to the solution of amine (1.45 mmol), EDCI (1.3 mmol), HOBt (1.3 mmol) in 10 mL CH_2Cl_2. The resulting solution was stirred at room temperature overnight. EtOAc was added to the solution, the mixture was washed with aqueous citric acid, aqueous saturated $NaHCO_3$ and brine, organic layer was dried over $MgSO_4$, filtered and concentrated. The residue was purified via column chromatography eluting with EtOAc/hexane to give the desired product. Yield (dimer: 80%~95%; tetramer: 65%~75%; hexamer: 50%~60%; octamer: 40%~65%).

General procedure B: TBS-deprotecting.

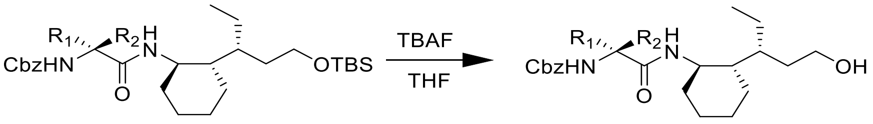

TBAF (1.30 mmol) and acetic acid (1.3 mmol) was added to a stirred solution of *tert*-butyldimethylsilane (0.81 mmol) in THF (20 mL) at 0 °C under nitrogen atmosphere. After addition, the reaction mixture was brought to room temperature and stirred overnight. The reaction mixture was quenched with brine and extracted with EtOAc, the organic layer was dried over $MgSO_4$, filtered and concentrated. The residue was purified via column chromatography eluting with EtOAc/hexane to give the desired product. Yield (85%~95%)

General procedure C: Oxidizing alcohol to carboxylic acid.

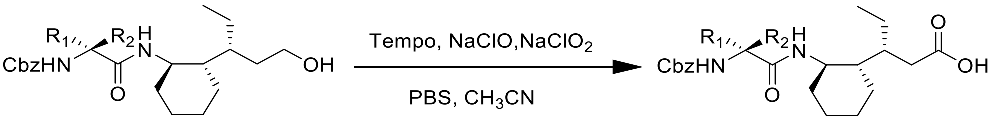

The alcohol compound (4 mmol) and Tempo (0.28 mmol) were dissolved in 20 mL of acetonitrile and 15 mL of 0.67M of sodium phosphate buffer (pH 6.7). 4 mL of sodium chlorite (2M in water) and 2 mL of sodium hypochlorite (0.3 % in water) were added simultaneously over 30 minutes, and the mixture was stirred at room temperature for another 30 minutes. The reaction mixture was quenched with sodium sulfite solution and extracted with ethyl acetate, the organic layer was dried over $MgSO_4$, filtered and concentrated. The crude product was carried on without further purification. Yield (92%~97%)

General procedure D: Protecting carboxylic acid with tert-butyl ester.

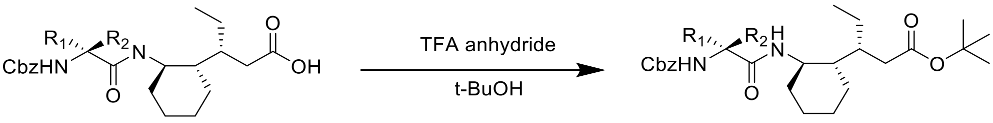

TFA anhydride (10 mmol) was added dropwise to a solution of carboxylic acid (2 mmol) in tert-butyl alcohol (20 mL) at 0 °C under nitrogen atmosphere. The mixture was brought to room temperature and reacted overnight, the mixture was quenched with saturated aqueous sodium bicarbonate, extracted with ethyl acetate, organic layer was dried over $MgSO_4$, filtered and concentrated. The residue was purified via column chromatography eluting with EtOAc/hexane to give the desired product. Yield (50%~56%)

General procedure E: Cbz-ester deprotection.

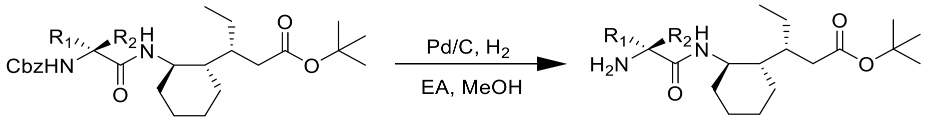

Benzyl ester compound (0.5 g) and Pd/C (50 mg) were dissolved in 10 mL of ethyl acetate and 10 mL of methanol, the reaction atmosphere was exchanged with hydrogen three times, and reacted under hydrogen over 2h at room temperature. The reaction mixture was filtered through a pad of celite, washed with ethyl acetate, and the organic layer was dried over $MgSO_4$, filtered and concentrated. The crude product was carried on without further purification. Yield (90%~95%)

2.2 Synthesis of Non-Catalytic α,δ-Oligomers

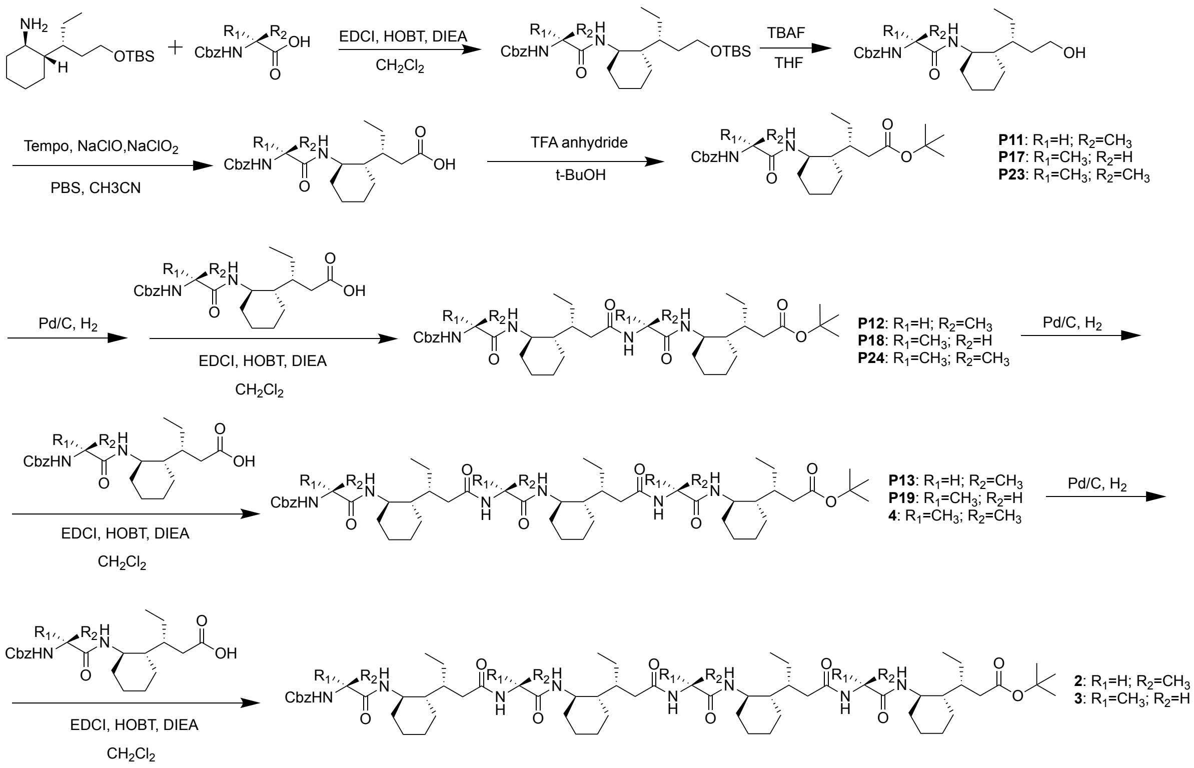

Synthesis of Foldamer 2

Cbz-L-Ala-AChPA•(CH_2OTBS) P8

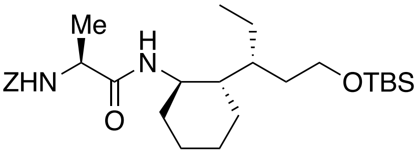

P8

Following general procedure A, **P8** was collected as a colourless oil. The crude product was carried on without further purification.

Cbz-L-Ala-AChPA•(CH_2OH) P9

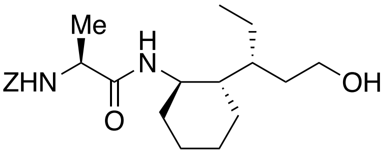

P9

Following general procedure B, **P9** was collected as a colourless oil. **^{1}H NMR** (400 MHz, $CDCl_3$) δ 7.41 – 7.27 (m, 5H), 6.32 (d, J = 9.3 Hz, 1H), 5.62 (d, J = 7.8 Hz, 1H), 5.22 – 4.91 (m, 2H), 4.11 (m, J = 7.1 Hz, 1H), 3.76 (h, J = 9.3, 8.4 Hz, 1H), 3.69 – 3.54 (m, 2H), 1.95 (d, J = 12.3 Hz, 1H), 1.83 – 1.58 (m, 4H), 1.58 – 1.42 (m, 2H), 1.41 – 1.20 (m, 6H), 1.20 – 0.98 (m, 3H), 0.98 – 0.84 (m, 1H), 0.79 (t, J = 7.1 Hz, 3H). **^{13}C NMR** (101 MHz, $CDCl_3$) δ 171.78, 156.42, 136.13, 128.67, 128.36, 128.19, 67.25, 60.76, 51.04, 49.88, 45.30, 34.79, 34.35, 33.74, 26.09, 25.52, 24.99, 22.60, 18.15, 13.06. **HRMS-ESI** (m/z) calc'd for $C_{22}H_{35}N_2O_4$ $[M+H]^+$, 391.2591; found, 391.2608.

Cbz-L-Ala-AChPA-OH P10

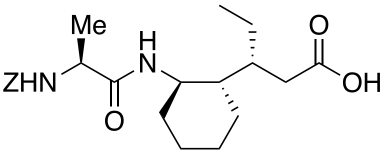

P10

Following general procedure C, **P10** was collected as a colourless oil. The crude product was carried on without further purification.

Cbz-L-Ala-AChPA-O^{t}Bu P11

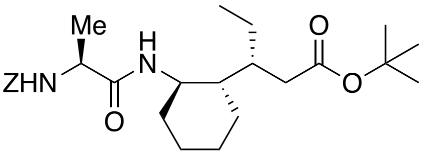

P11

Following general procedure D, **P11** was collected as a white solid. **^{1}H NMR** (400 MHz, $CDCl_3$) d 7.54 – 7.22 (m, 5H), 6.34 (d, J = 8.7 Hz, 1H), 5.78 (d, J = 8.0 Hz, 1H), 5.20 – 5.06 (m, 2H), 4.29 (p, J = 7.3 Hz, 1H), 3.67 (tdd, J = 10.7, 8.7, 4.1 Hz, 1H), 2.27 (dd, J = 16.3, 3.8 Hz, 1H), 2.11 – 1.97 (m, 2H), 1.86 (td, J = 10.6, 4.9 Hz, 1H), 1.77 – 1.57 (m, 3H), 1.52 – 1.32 (m, 13H), 1.23 (t, J = 7.1 Hz, 1H), 1.08 (tdd, J = 24.0, 12.0, 9.7 Hz,4), 0.97 – 0.86 (m, 1H), 0.81 (t, J = 7.2 Hz, 3H). **^{13}C NMR** (101 MHz, $CDCl_3$) d 173.78, 171.51, 155.91, 136.38, 128.43, 128.11, 128.05, 80.54, 66.85, 50.77, 49.76, 46.19,

37.27, 35.84, 33.99, 28.08, 25.93, 25.12, 25.04, 21.75, 19.15, 12.78. **HRMS-ESI** (m/z) calc'd for $C_{26}H_{40}N_2O_5Na$ $[M+Na]^+$, 483.2830; found, 483.2824.

Cbz-L-Ala-AChPA-L-Ala-ACPA-O^{t}Bu P12

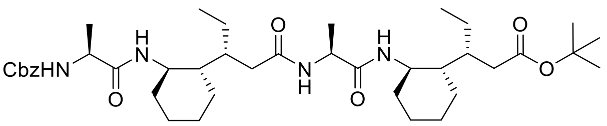

P12

Following general procedure A, **P12** was collected as a white solid. **^{1}H NMR** (400 MHz, $CDCl_3$) δ 7.67 (d, J = 9.5 Hz, 1H), 7.33 (d, J = 3.7 Hz, 5H), 7.21 (d, J = 7.8 Hz, 1H), 6.22 (d, J = 7.8 Hz, 1H), 5.49 (d, J = 7.8 Hz, 1H), 5.16 – 4.94 (m, 2H), 4.50 (p, J = 7.2 Hz, 1H), 4.20 (p, J = 7.1 Hz, 1H), 3.74 (qd, J = 10.7, 3.9 Hz, 1H), 3.64 (tdd, J = 11.2, 7.7, 4.1 Hz, 1H), 2.58 – 2.32 (m, 2H), 2.14 – 1.99 (m, 3H), 1.98 – 1.84 (m, 3H), 1.79 – 1.59 (m, 6H), 1.46 (m, 11H), 1.32 (d, J = 7.2 Hz, 3H), 1.35-1.27 (m, 6H), 1.18 – 0.94 (m, 8H), 0.91 – 0.88 (m, 4H), 0.80 (t, J = 6.9 Hz, 3H). **^{13}C NMR** (101 MHz, $CDCl_3$) δ 173.85, 173.43, 173.15, 172.75, 156.12, 136.45, 128.59, 128.24, 80.65, 67.01, 51.03, 50.54, 49.23, 48.82, 45.29, 44.69, 38.65, 37.27, 36.91, 35.48, 34.15, 34.07, 28.33, 26.08, 25.79, 25.51, 25.20, 25.14, 24.72, 22.23, 22.02, 18.53, 13.02, 12.88. **HRMS-ESI** (m/z) calc'd for $C_{40}H_{64}N_4O_7Na$ $[M+Na]^+$, 735.4668; found, 735.4672.

Cbz-L-Ala-AChPA-L-Ala-ACPA-L-Ala-AChPA-O^{t}Bu P13

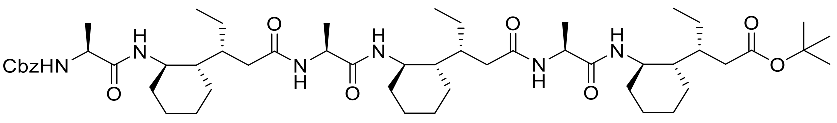

P13

Following general procedure A, **P13** was collected as a white solid.

^{1}H NMR (400 MHz, $CDCl_3$) δ 7.98 (d, J = 9.6 Hz, 1H), 7.79 (d, J = 9.7 Hz, 1H), 7.49 (d, J = 6.2 Hz, 1H), 7.44 (d, J = 7.3 Hz, 1H), 7.24 – 7.13 (m, 5H), 6.14 (d, J = 7.7 Hz, 1H), 5.15 (d, J = 7.0 Hz, 1H), 5.04 – 4.78 (m, 2H), 4.33 (p, J = 7.3 Hz, 1H), 4.22 (p, J = 7.0 Hz, 1H), 3.98 (p, J = 6.9 Hz, 1H), 3.71 – 3.47 (m, 3H), 2.41 (ddd, J = 23.7, 13.6, 3.1 Hz, 2H), 2.29 – 2.20 (m, 1H), 2.01 – 1.71 (m, 9H), 1.67 – 1.44 (m, 9H), 1.32 (m, 12H), 1.25 (d, J = 6.9 Hz, 3H), 1.21 – 1.12 (m, 12H), 1.04 – 0.88 (m, 9H), 0.80 – 0.68 (m, 12H). **^{13}C NMR** (101 MHz, $CDCl_3$) δ 174.38, 173.89, 173.86, 173. 77, 173.72, 172.84, 156.14, 136.33, 128.60, 128.25, 128.11, 80.65, 66.99, 51.27, 50.45, 49.96, 49.31, 49.26, 45.21, 44.61, 44.21, 39.44, 38.81, 37.38, 37.34, 36.62, 35.40, 34.36, 34.14, 33.74, 28.34, 26.10, 25.72, 25.49, 25.43, 25.30, 25.20, 25.01, 24.80, 23.01, 22.38, 22.07, 18.43, 18.06, 17.68, 13.97, 13.00, 12.85. **HRMS-ESI** (m/z) calc'd for $C_{54}H_{88}N_6O_9Na$ $[M+Na]^+$, 987.6505; found, 987.6491.

Cbz-L-Ala-AChPA-L-Ala-AChPA-L-Ala-AChPA-L-Ala-AChPA-O^{t}Bu 2

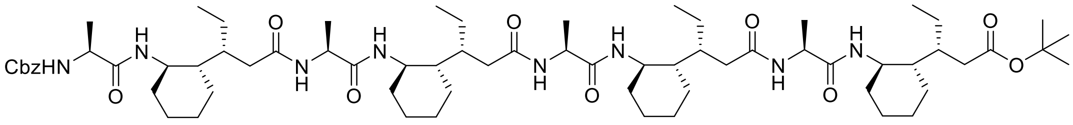

Following general procedure A, Foldamer **2** was collected as a white solid.

^{1}H NMR (400 MHz, $CDCl_3$) δ 8.15 (d, J = 4.4 Hz, 1H), 8.12 (d, J = 4.3 Hz, 1H), 7.98 (d, J = 6.0 Hz, 1H), 7.88 (d, J = 9.7 Hz, 1H), 7.71 (d, J = 7.4 Hz, 1H), 7.66 (d, J = 5.8 Hz, 1H), 7.38 – 7.27 (m, 5H), 6.23 (d, J = 7.8 Hz, 1H), 5.24 (d, J = 7.0 Hz, 1H), 5.13 (d, J = 12.3 Hz, 1H), 4.96 (d, J = 12.3 Hz, 1H), 4.47 (p, J = 7.3 Hz, 1H), 4.34 (dt, J = 11.3, 6.6 Hz, 2H), 4.09 (p, J = 6.9 Hz, 1H), 3.85 – 3.58 (m, 4H), 2.63 – 2.49 (m, 3H), 2.37 (d, J = 12.9 Hz, 1H), 2.09 (dd, J = 22.8, 9.3 Hz, 6H), 2.00 – 1.82 (m, 9H), 1.69 (d, J = 10.6 Hz, 12H), 1.46 (m, 13H), 1.38 (d, J = 6.9 Hz, 3H), 1.28 (m, 15H), 1.20 – 1.03 (m, 12H), 1.01 – 0.94 (m, 8H), 0.93 – 0.79 (m, 7H). **^{13}C NMR** (101 MHz, $CDCl_3$) δ 174.46, 174.20, 174.12, 173.79, 173.67, 172.73, 156.12, 136.31, 128.60, 128.25, 128.08, 80.58, 66.97, 60.53, 53.56, 51.35, 50.39, 50.20, 49.17, 49.13, 49.01, 45.18, 44.72, 44.38, 44.03, 39.61, 39.51, 38.91, 37.47, 37.32, 36.77, 36.47, 35.38, 34.36, 34.14, 34.07, 33.80, 31.71, 28.34, 26.09, 25.73, 25.51, 25.41, 25.15, 25.04, 24.97, 24.78, 23.03, 22.78, 22.44, 22.08, 21.20, 18.43, 17.86, 17.70, 17.47, 14.32, 14.26, 14.02, 13.99, 12.96, 12.83. **HRMS-ESI** (m/z) calc'd for $C_{68}H_{112}N_8O_{11}Na$ $[M+Na]^+$, 1239.8343; found, 1239.8347.

Synthesis of Octamer 3

Cbz-D-Ala-AChPA•(CH_2OTBS) P14

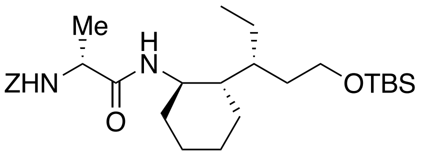

P14

Following general procedure A, **P14** was collected as a colourless oil. **^{1}H NMR** (400 MHz, $CDCl_3$) δ 7.35 (d, J = 3.7 Hz, 5H), 5.57 (d, J = 9.3 Hz, 1H), 5.47 (d, J = 7.3 Hz, 1H), 5.10 (s, 2H), 4.14 (t, J = 7.3 Hz, 1H), 3.88 – 3.69 (m, 1H), 3.61 (t, J = 6.6 Hz, 2H), 1.95 (t, J = 7.4 Hz, 1H), 1.69 (d, J = 9.1 Hz, 2H), 1.59 (dp, J = 11.0, 4.0 Hz, 1H), 1.47 (qd, J = 7.1, 4.3 Hz, 2H), 1.37 (d, J = 7.0 Hz, 4H), 1.32 – 1.21 (m, 4H), 1.14 – 1.01 (m, 2H), 0.90 (m, 10H), 0.82 (t, J = 7.1 Hz, 3H), 0.06 (d, J = 0.9 Hz, 6H). **^{13}C NMR** (101 MHz, $CDCl_3$) δ 171.12, 155.79, 136.32, 128.54, 128.16, 128.02, 66.86, 62.13, 53.44, 50.69, 49.76, 45.65, 36.20, 34.44, 33.92, 26.00, 25.40, 25.18, 22.37, 19.43, 18.35, 13.06, -5.18. **HRMS-ESI** (m/z) calc'd for $C_{28}H_{48}N_2O_4Na$ $[M+Na]^+$, 527.3276; found, 527.3295.

Cbz-D-Ala-AChPA•(CH_2OH) P15

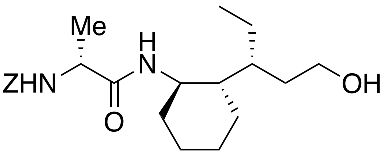

P15

Following general procedure B, **P15** was collected as a colourless oil. **^{1}H NMR** (400 MHz, $CDCl_3$) δ 7.40 – 7.29 (m, 5H), 6.08 (d, J = 9.2 Hz, 1H), 5.43 (d, J = 7.2 Hz, 1H), 5.10 (s, 2H), 4.21 – 4.12 (m, 1H), 3.74 (qd, J = 10.8, 10.0, 3.1 Hz, 1H), 3.67 – 3.56 (m, 2H), 1.99 – 1.92 (m, 1H), 1.76 – 1.59 (m, 3H), 1.59 – 1.45 (m, 2H), 1.38 (d, J = 7.1 Hz, 3H), 1.36 – 1.23 (m, 5H), 1.10 (dqd, J = 14.9, 12.5, 2.9 Hz, 2H), 0.99 – 0.90 (m, 1H), 0.85 (q, J = 7.2, 5.9 Hz, 3H). **^{13}C NMR** (101 MHz, $CDCl_3$) δ 171.34 (d, J = 6.9 Hz), 156.19, 136.27, 128.69, 128.37, 128.18, 67.21, 61.05, 60.53, 51.06, 49.89, 45.34, 35.42, 34.37, 33.73, 26.10, 25.53, 25.15, 22.64, 19.12, 13.12. **HRMS-ESI** (m/z) calc'd for $C_{22}H_{35}N_2O_4$ $[M+H]^+$, 391.2592; found, 391.2608.

Cbz-D-Ala-AChPA-OH P16

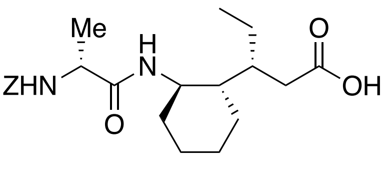

P16

Following general procedure C, **P16** was collected as a colourless oil. **^{1}H NMR** (400 MHz, $CDCl_3$) δ 7.35 (d, J = 4.7 Hz, 5H), 6.46 (d, J = 9.1 Hz, 1H), 6.28 (s, 1H), 5.86 (d, J = 7.8 Hz, 1H), 5.18 – 5.05 (m, 2H), 4.35 (t, J = 7.3 Hz, 1H), 3.77 (d, J = 11.6 Hz, 1H), 2.55 – 2.41 (m, 1H), 2.30 – 2.15 (m, 1H), 1.96 (d, J = 13.0 Hz, 2H), 1.71 (d, J = 16.3 Hz, 3H), 1.43 (d, J = 7.0 Hz, 3H), 1.31 – 1.27 (m, 3H), 1.08 (d, J = 11.2 Hz, 2H), 0.92 – 0.87 (m, 4H). **^{13}C NMR** (101 MHz, $CDCl_3$) δ 178.02, 172.30, 156.15, 136.22, 128.54, 128.17, 127.92, 66.99, 50.72, 49.66, 45.86, 36.00, 35.29, 33.86, 29.70, 25.80, 25.23, 21.51, 19.35, 12.77. **HRMS-ESI** (m/z) calc'd for $C_{22}H_{33}N_2O_5$ $[M+H]^+$, 405.2384; found, 405.2400.

Cbz-D-Ala-AChPA-O*t*Bu P17

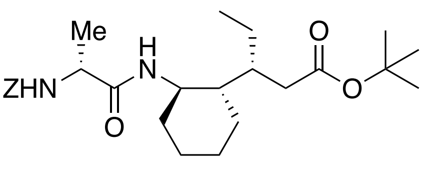

P17

Following general procedure D, **P17** was collected as a white solid. **^{1}H NMR** (400 MHz, $CDCl_3$) δ 7.39 – 7.27 (m, 5H), 6.12 (d, J = 8.9 Hz, 1H), 5.64 (d, J = 7.7 Hz, 1H), 5.08 (d, J = 3.2 Hz, 2H), 4.22 (p, J = 7.1 Hz, 1H), 3.69 (tdd, J = 11.0, 8.6, 4.1 Hz, 1H), 2.29 (dd, J = 16.0, 4.1 Hz, 1H), 2.12 – 1.96 (m, 2H), 1.94 – 1.84 (m, 1H), 1.75 – 1.61 (m, 3H), 1.53 – 1.37 (m, 13H), 1.35 – 1.17 (m, 3H), 1.06 (q, J = 12.5, 11.9 Hz, 3H), 0.98 – 0.87 (m, 1H), 0.82 (t, J = 7.2 Hz, 3H). **^{13}C NMR** (101 MHz, $CDCl_3$) δ 173.56, 171.59, 155.81, 136.54, 128.56, 128.40, 128.14, 128.08, 80.46, 66.83, 50.78, 50.00, 46.09, 37.43, 36.23, 34.28, 28.25, 26.04, 25.27, 25.11, 21.82, 19.71, 12.90. **HRMS-ESI** (m/z) calc'd for $C_{26}H_{40}N_2O_5Na$ $[M+Na]^+$, 483.2830; found, 483.2869.

Cbz-D-Ala-AChPA-D-Ala-AChPA-O^{t}Bu P18

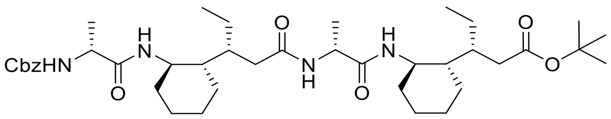

P18

Following general procedure A, **P18** was collected as a white solid. **^{1}H NMR** (400 MHz, $CDCl_3$) δ 7.30 – 7.15 (m, 5H), 6.90 (d, J = 8.2 Hz, 1H), 6.76 (d, J = 7.4 Hz, 1H), 6.53 (d, J = 8.8 Hz, 1H), 5.88 (d, J = 7.6 Hz, 1H), 5.13 – 4.88 (m, 2H), 4.41 (p, J = 7.0 Hz, 1H), 4.19 (p, J = 7.0 Hz, 1H), 3.60 (dddd, J = 23.4, 15.2, 13.2, 7.6 Hz, 2H), 2.23 (dd, J = 15.8, 4.5 Hz, 2H), 2.05 – 1.79 (m, 6H), 1.70 – 1.48 (m, 6H), 1.46 – 1.28 (m, 17H), 1.25 – 1.12 (m, 4H), 1.10 – 0.93 (m, 6H), 0.93 – 0.80 (m, 2H), 0.76 (td, J = 7.2, 3.2 Hz, 6H). **^{13}C NMR** (101 MHz, $CDCl_3$) δ 173.52, 172.82, 171.83, 171.65, 155.71, 136.54, 128.40, 127.92, 127.85, 80.41, 66.54, 50.65, 50.01, 49.82, 48.94, 46.08, 45.54, 38.03, 37.52, 36.24, 36.15, 34.19, 33.96, 28.12, 25.90, 25.85, 25.21, 25.17, 24.97, 21.96, 21.74, 19.71, 19.32, 12.92, 12.81. **HRMS-ESI** (m/z) calc'd for $C_{40}H_{64}N_4O_7Na$ $[M+Na]^+$, 713.4848; found, 713.4838.

Cbz-D-Ala-AChPA-D-Ala-AChPA-D-Ala-AChPA-O^{t}Bu P19

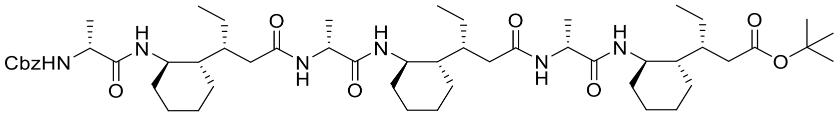

P19

Following general procedure A, **P19** was collected as a white solid.

^{1}H NMR (400 MHz, $CDCl_3$) δ 7.36 – 7.27 (m, 5H), 6.88 (d, J = 8.5 Hz, 1H), 6.68 (d, J = 8.2 Hz, 1H), 6.51 (d, J = 7.1 Hz, 1H), 6.45 (d, J = 8.8 Hz, 1H), 6.30 (d, J = 7.4 Hz, 1H), 5.81 (d, J = 7.6 Hz, 1H), 5.09 (s, 2H), 4.48 (td, J = 7.1, 4.0 Hz, 2H), 4.30 (t, J = 7.2 Hz, 1H), 3.70 (dq, J = 37.1, 10.1, 9.2 Hz, 3H), 2.38 – 2.23 (m, 6H), 2.11 – 1.94 (m, 8H), 1.92 (d, J = 10.6 Hz, 1H), 1.78 – 1.55 (m, 10H), 1.54 – 1.37 (m, 21H), 1.27 (dd, J = 13.3, 6.7 Hz, 7H), 1.18 – 1.01 (m, 9H), 1.01 – 0.89 (m, 4H), 0.89 – 0.79 (m, 9H). **^{13}C NMR** (101 MHz, $CDCl_3$) δ 173.47, 172.74, 172.60, 171.78, 171.63 171.58 155.70, 136.51, 128.48, 127.99, 127.81, 80.47, 66.61, 50.65, 50.46, 49.97, 49.09, 48.96, 46.22, 45.83, 45.52, 38.34, 37.83, 37.55, 36.25, 36.07, 34.22, 34.14, 34.08, 28.17, 25.94, 25.24, 25.20. 25.14, 25.01, 21.96, 21.82, 19.97, 19.75, 18.79, 13.00, 12.92, 12.87. **HRMS-ESI** (m/z) calc'd for $C_{54}H_{89}N_6O_9$ $[M+H]^+$, 987.6505; found, 987.6491.

Cbz-D-Ala-AChPA-D-Ala-AChPA-D-Ala-AChPA-D-Ala-AChPA-O^{t}Bu 3

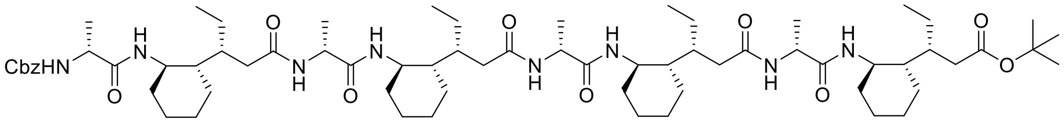

Following general procedure A, octamer **3** was collected as a white solid.

^{1}**H NMR** (400 MHz, $CDCl_3$) δ 7.42 – 7.27 (m, 5H), 7.01 (d, J = 8.5 Hz, 1H), 6.96 (d, J = 8.0 Hz, 1H), 6.87 (d, J = 8.3 Hz, 1H), 6.60 (d, J = 7.8 Hz, 2H), 6.46 (d, J = 7.4 Hz, 1H), 6.37 (d, J = 7.2 Hz, 1H), 5.92 (d, J = 7.6 Hz, 1H), 5.09 (d, J = 2.9 Hz, 2H), 4.49 (dt, J = 13.2, 6.9 Hz, 3H), 4.33 – 4.18 (m, 1H), 3.87 – 3.54 (m, 4H), 2.49 – 2.23 (m, 4H), 2.18 – 1.86 (m, 12H), 1.80 – 1.55 (m, 12H), 1.53 – 1.36 (m, 25H), 1.34 – 1.21 (m, 9H), 1.22 – 0.92 (m, 16H), 0.91 – 0.74 (m, 12H). ^{13}**C NMR** (101 MHz, $CDCl_3$) δ 173.59, 173.06, 172.89, 172.77, 172.04, 171.96, 171.88, 155.83, 136.75, 128.58, 128.07, 127.96, 80.53, 77.48, 66.67, 50.79, 50.54, 50.46, 50.06, 49.29, 49.11, 46.37, 45.71, 45.61, 38.25, 38.19, 38.08, 38.08, 37.73, 36.49, 36.44, 36.23, 36.14, 34.30, 34.17, 31.73, 28.29, 26.06, 25.39, 25.27, 25.20, 25.14, 22.79, 22.06, 21.95, 20.11, 19.90, 19.18, 18.98, 14.26, 13.10, 12.98. **HRMS-ESI** (m/z) calc'd for $C_{68}H_{112}N_8O_{11}Na$ [M+Na]$^+$, 1239.8343; found, 1239.8347.

Synthesis of Hexamer 4

Cbz-Aib-AChPA•(CH_2OTBS) P20

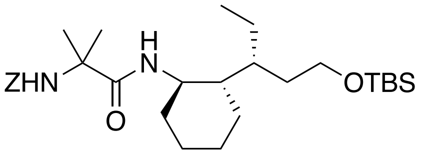

P20

Following general procedure A, **P20** was collected as a colourless oil. The crude product was carried on without further purification.

Cbz-Aib-AChPA• (CH_2OH) P21

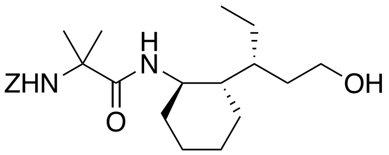

P21

Following general procedure B, **P21** was collected as a colourless oil. The crude product was carried on without further purification.

Cbz-Aib-AChPA-OH P22

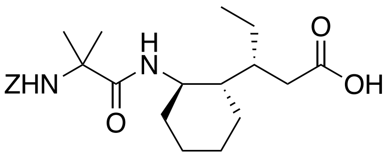

P22

Following general procedure C, **P22** was collected as a colourless oil. ^{1}**H NMR** (400 MHz, $CDCl_3$) δ 7.39 – 7.26 (m, 5H), 6.26 (b, 1H), 5.80 (b, 1H), 5.09 (d, J = 11.5 Hz, 2H), 3.69 (tdd, J = 11.0, 8.7, 4.1 Hz, 1H), 2.50 – 2.35 (m, 1H), 2.28 – 2.13 (m, 1H), 2.08 – 1.89 (m, 2H), 1.79 – 1.60 (m, 3H), 1.59 – 1.45 (m, 7H), 1.25 (m, J = 7.1 Hz, 2H), 1.15 – 1.03 (m, 2H), 1.03 – 0.89 (m, 2H), 0.84 (t, J = 7.1 Hz, 3H). ^{13}**C NMR** (101 MHz, $CDCl_3$) δ 178.23, 173.86, 128.64, 128.28, 128.19, 66.56, 50.08, 46.05, 35.88, 35.81, 33.94,

31.07, 29.83, 25.97, 25.28, 25.05, 21.80, 12.93. **HRMS-ESI** (m/z) calc'd for $C_{23}H_{35}N_2O_5$ $[M+H]^+$, 419.2541; found, 419.2550.

Cbz-Aib-ACPA-O^{t}Bu P23

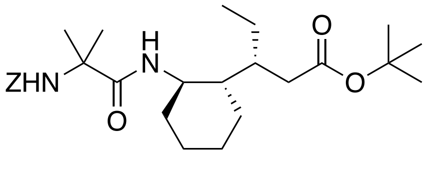

P23

Following general procedure D, **P23** was collected as a white solid. **^{1}H NMR** (400 MHz, $CDCl_3$) δ 7.41 – 7.27 (m, 5H), 6.33 (d, J = 8.3 Hz, 1H), 5.68 (s, 1H), 5.16 – 5.03 (m, 2H), 3.66 (tdd, J = 11.3, 8.2, 4.2 Hz, 1H), 2.30 (dd, J = 16.3, 3.8 Hz, 1H), 2.13 – 2.02 (m, 2H), 1.97 – 1.85 (m, 1H), 1.70 (m, 3H), 1.57 (m, 6H), 1.44 (m, 10H), 1.32 – 1.22 (m, 2H), 1.16 – 1.04 (m, 2H), 1.02 – 0.87 (m, 2H), 0.83 (t, J = 7.2 Hz, 3H). **^{13}C NMR** (101 MHz, $CDCl_3$) δ 173.82, 154.94, 136.72, 128.61, 128.19, 128.16, 80.54, 77.48, 77.16, 76.84, 66.55, 56.90, 50.28, 46.10, 37.41, 36.09, 34.14, 28.31, 26.18, 25.25, 25.22, 21.90, 12.97. **HRMS-ESI** (m/z) calc'd for $C_{27}H_{43}N_2O_5$ $[M+H]^+$, 475.3167; found, 475.3163.

Cbz-Aib-AChPA-Aib-AChPA-O^{t}Bu P24

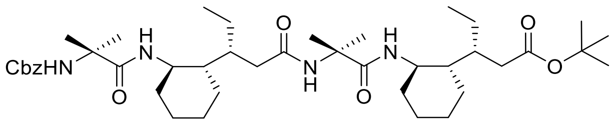

P24

Following general procedure D, **P24** was collected as a white solid.

^{1}H NMR (400 MHz, $CDCl_3$) δ 7.30 – 7.16 (m, 5H), 6.78 (d, J = 8.3 Hz, 1H), 6.60 (s, 1H), 6.49 (d, J = 8.0 Hz, 1H), 6.08 (s, 1H), 5.06 – 4.92 (m, 2H), 3.58 (dtt, J = 11.4, 7.8, 4.1 Hz, 2H), 2.30 – 2.18 (m, 2H), 2.05 – 1.96 (m, 2H), 1.95 – 1.81 (m, 4H), 1.67 – 1.47 (m, 18H), 1.44 – 1.32 (m, 11H), 1.30 – 1.18 (m, 4H), 1.07 – 0.81 (m, 8H), 0.76 (t, J = 7.2 Hz, 6H). **^{13}C NMR** (101 MHz, $CDCl_3$) δ 174.20, 174.10, 173.83, 172.85, 154.78, 136.76, 128.40, 127.88, 127.87, 80.49, 66.15, 56.98, 56.63, 50.45, 50.15, 45.74, 45.02, 38.76, 37.22, 36.51, 35.79, 34.03, 33.96, 28.13, 25.98, 25.22, 25.16, 25.03, 24.63, 22.10, 21.79, 12.92, 12.90. **HRMS-ESI** (m/z) calc'd for $C_{42}H_{69}N_4O_7$ $[M+H]^+$, 741.5161; found, 741.5180.

Cbz-Aib-AChPA-Aib-AChPA-Aib-AChPA-O^{t}Bu 4

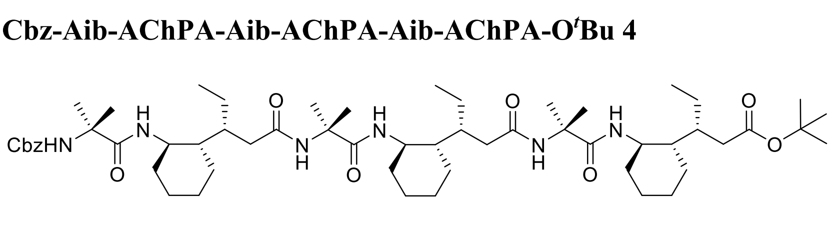

Following general procedure D, Foldamer **4** was collected as a white solid. **^{1}H NMR** (400 MHz, $CDCl_3$) δ 7.41 – 7.23 (m, 5H), 6.98 (d, J = 7.8 Hz, 1H), 6.84 (d, J = 9.7 Hz, 2H), 6.65 (s, 1H), 6.50 (d, J = 7.9 Hz, 1H), 6.16 (s, 1H), 5.14 – 4.96 (m, 2H), 3.77 – 3.53 (m, 3H), 2.31 (dq, J = 16.7, 3.7, 3.2 Hz, 3H), 2.13 – 1.86 (m, 9H), 1.83 – 1.48 (m, 27H), 1.43 (s, 12H), 1.26 (m, 6H), 1.18 – 0.89 (m, 12H), 0.84 (t, J

= 8.2 Hz, 9H). **^{13}C NMR** (101 MHz, $CDCl_3$) δ 174.62, 174.24, 174.17, 173.94, 173.01, 172.77, 154.83, 136.91, 128.51, 127.96, 80.65, 66.09, 57.12, 57.04, 56.70, 50.88, 50.64, 50.25, 45.84, 45.14, 44.73, 38.95, 38.59, 37.28, 36.76, 36.58, 35.83, 34.14, 34.10, 28.25, 26.13, 26.08, 25.61, 25.27, 25.25, 25.20, 25.13, 25.11, 25.02, 24.80, 24.40, 22.40, 22.10, 21.92, 13.14, 13.06, 13.05. **HRMS-ESI** (m/z) calc'd for $C_{57}H_{94}N_6O_9Na$ $[M+Na]^+$, 1029.6975; found, 1029.6995.

2.3 Preparation of Catalytic Octamers

Synthesis of Catalytic α,δ-Foldamer 6

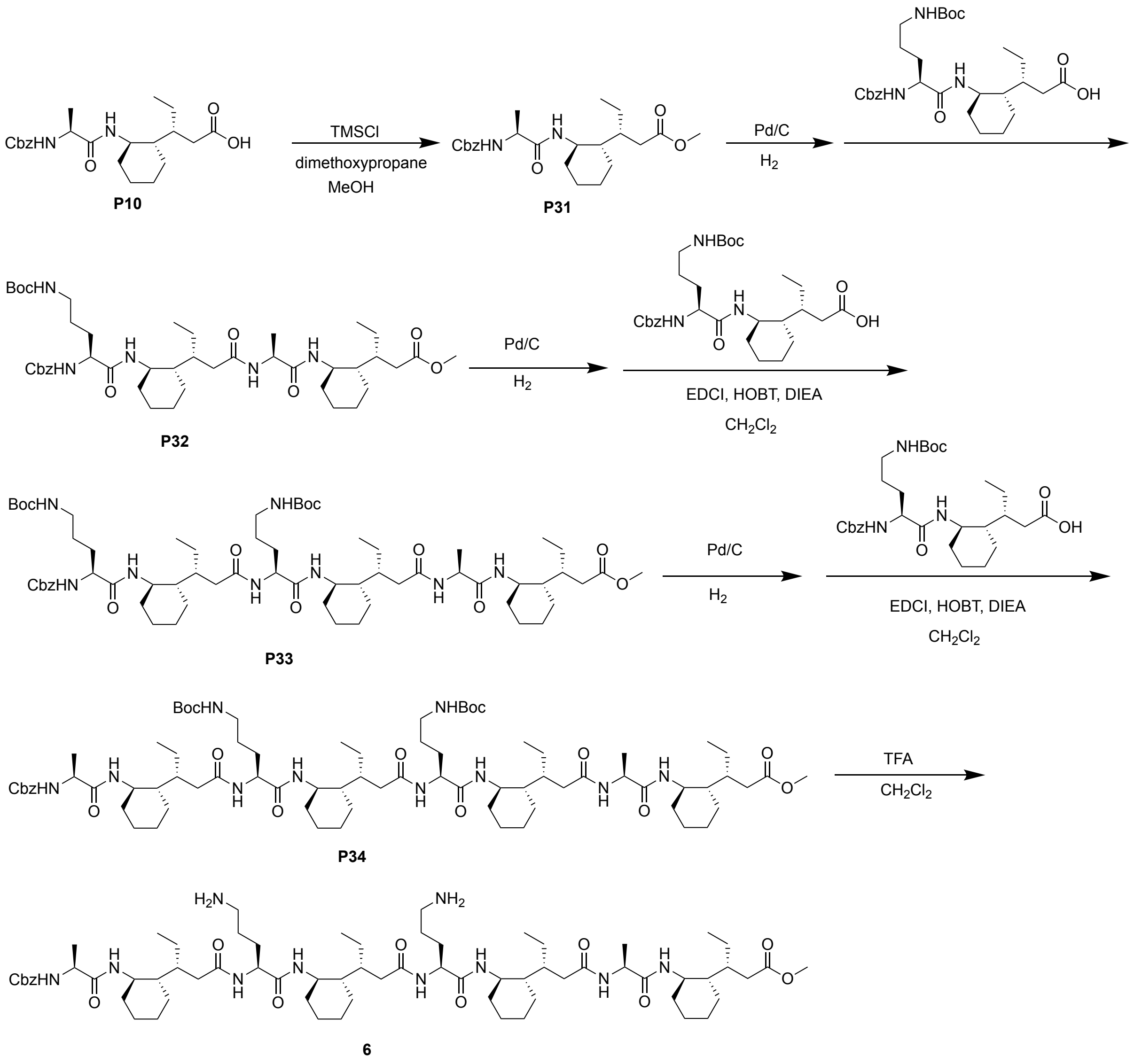

General procedure F: Boc-deprotection

To the *tert*-butyl carbamate-foldamer (50 mg) in dichloromethane (2 mL) was added TFA (2 mL) at 0 °C under nitrogen atmosphere, the mixture was stirred at room temperature for 2 h. $NaHCO_3$ solution

was slowly added at 0 °C and the solution was extracted with ethyl acetate, the organic layer was dried over $MgSO_4$, filtered and concentrated to give the amine catalyst.

Cbz-L-Ala-AChPA-OMe P31

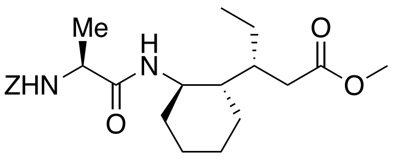

P31

Following general procedure A, **P31** was collected as a white solid. **^{1}H NMR** (400 MHz, $CDCl_3$) δ 7.35 (m, 5H), 6.12 (d, J = 8.9 Hz, 1H), 5.50 (s, 1H), 5.14 (s, 2H), 4.27 (t, J = 7.5 Hz, 1H), 3.75 – 3.66 (m, 1H), 3.65 (s, 3H), 2.37 (dd, J = 16.8, 3.5 Hz, 1H), 2.16 (dd, J = 16.8, 10.9 Hz, 1H), 2.10 – 2.00 (m, 1H), 1.90 (t, J = 10.8 Hz, 1H), 1.77 – 1.60 (m, 3H), 1.54 – 1.43 (m, 1H), 1.41 (d, J = 7.0 Hz, 3H), 1.25 (m, 1H), 1.19 – 0.99 (m, 4H), 0.99 – 0.89 (m, 1H), 0.83 (t, J = 7.2 Hz, 3H). **^{13}C NMR** (101 MHz, $CDCl_3$) δ 174.87, 171.58, 156.00, 136.55, 128.64, 128.27, 128.19, 67.07, 51.74, 50.94, 49.92, 46.30, 35.92, 35.79, 34.12, 26.00, 25.26, 25.15, 21.89, 19.28, 12.95. **HRMS-ESI** (m/z) calc'd for $C_{23}H_{33}N_2O_5$ $[M+H]^+$, 419.2540; found, 419.2550.

Cbz-L-Orn•Boc-AChPA-L-Ala-AChPA-OMe P32

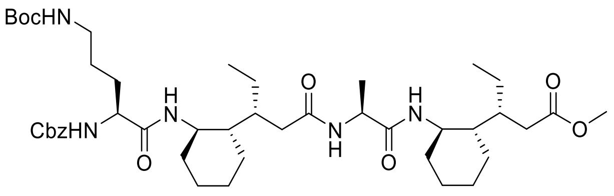

P32

Following general procedure A, **P32** was collected as a white solid.

^{1}H NMR (400 MHz, $CDCl_3$) δ 7.78 (d, J = 9.5 Hz, 1H), 7.41 – 7.27 (m, 6H), 6.23 (d, J = 8.1 Hz, 1H), 5.42 (d, J = 8.3 Hz, 1H), 5.22 – 4.94 (m, 2H), 4.88 (s, 1H), 4.48 (t, J = 7.3 Hz, 1H), 4.17 (d, J = 7.6 Hz, 1H), 3.69 (m, 5H), 3.19 (dd, J = 13.2, 6.5 Hz, 1H), 3.14 – 2.99 (m, 1H), 2.66 – 2.34 (m, 2H), 2.26 – 2.07 (m, 2H), 2.05 – 1.87 (m, 4H), 1.76 – 1.46 (m, 12H), 1.42 (s, 9H), 1.32 (d, J = 7.2 Hz, 3H), 1.30 – 1.22 (m, 3H), 1.08 (m, 8H), 0.90 (m, J = 7.2 Hz, 4H), 0.80 (t, J = 6.9 Hz, 3H). **^{13}C NMR** (101 MHz, $CDCl_3$) δ 174.93, 173.72, 173.46, 171.81, 156.49, 156.10, 136.41, 128.62, 128.28, 128.25, 79.19, 67.13, 54.72, 51.77, 50.51, 49.09, 45.37, 44.69, 39.65, 38.80, 37.03, 35.84, 35.54, 34.32, 34.28, 29.50, 28.58, 26.01, 25.80, 25.49, 25.25, 25.16, 24.76, 22.34, 22.16, 18.53, 13.14, 12.90. **HRMS-ESI** (m/z) calc'd for $C_{44}H_{72}N_5O_9$ $[M+H]^+$, 814.5325; found, 814.5335.

Cbz-L-Orn•Boc-AChPA-L-Orn•Boc-AChPA-L-Ala-AChPA-OMe P33

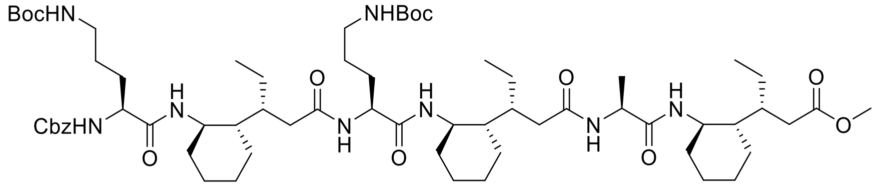

P33

Following general procedure A, **P33** was collected as a white solid. **^{1}H NMR** (400 MHz, $CDCl_3$) δ 8.13 (d, J = 9.7 Hz, 1H), 7.97 (d, J = 9.5 Hz, 1H), 7.50 (d, J = 6.8 Hz, 1H), 7.44 (d, J = 6.4 Hz, 1H), 7.38 – 7.28 (m, 5H), 6.27 (d, J = 8.2 Hz, 1H), 5.27 (m, 3H), 5.03 (q, J = 12.4 Hz, 2H), 4.39 (m, 2H), 4.09 (m, 1H), 3.68 (m, 6H), 3.30 – 3.05 (m, 2H), 2.99 – 2.83 (m, 2H), 2.60 – 2.42 (m, 3H), 2.19 (m, 2H), 2.02 – 1.86 (m, 7H), 1.83 – 1.58 (m, 15H), 1.56 – 1.36 (m, 24H), 1.35 – 1.29 (m, 5H), 1.22 – 1.02 (m, 12H), 1.01 – 0.76 (m, 12H). **^{13}C NMR** (101 MHz, $CDCl_3$) δ 174.86, 174.78, 174.60, 173.66, 172.10, 156.87, 156.24, 139.43, 136.21, 128.64, 128.30, 128.08, 114.21, 78.97, 67.16, 54.30, 53.45, 51.74, 50.41, 49.85, 49.22, 49.09, 45.37, 44.49, 44.33, 39.35, 39.04, 37.71, 37.00, 35.91, 35.35, 34.56, 34.44, 33.86, 32.07, 30.45, 29.84, 28.62, 25.99, 25.67, 25.43, 25.28, 25.19, 25.02, 24.83, 23.02, 22.83, 22.48, 22.27, 18.02, 14.26, 14.13, 13.24, 12.93. **HRMS-ESI** (m/z) calc'd for $C_{665}H_{109}N_8O_{13}$ $[M+H]^+$, 1209.8109; found, 1209.8149.

Cbz-L-Ala-AChPA-L-Orn•Boc-AChPA-L-Orn•Boc-AChPA-L-Ala-AChPA-OMe

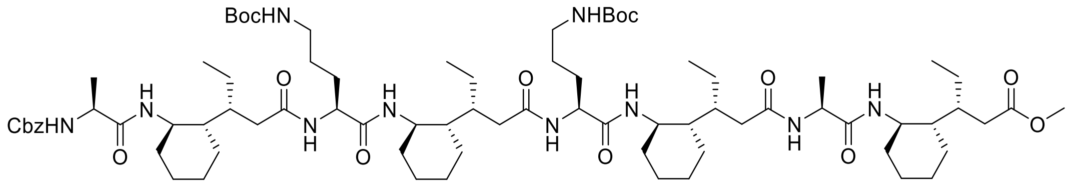

P34

Following general procedure A, **P34** was collected as a white solid. **^{1}H NMR** (400 MHz, $CDCl_3$) δ 8.23 (d, J = 9.8 Hz, 1H), 8.07 (d, J = 9.7 Hz, 1H), 7.98 (d, J = 9.6 Hz, 1H), 7.82 (d, J = 6.7 Hz, 1H), 7.63 (m, 2H), 7.43 – 7.29 (m, 5H), 6.31 (d, J = 8.4 Hz, 1H), 5.70 (s, 1H), 5.31 (s, 0H), 5.27 (d, J = 7.0 Hz, 1H), 5.16 – 5.00 (m, 2H), 4.43 (p, J = 7.3 Hz, 2H), 4.31 (m, 1H), 4.08 (p, J = 7.2 Hz, 1H), 3.71 (m, 7H), 3.18 (m, J = 14.8 Hz, 2H), 2.93 (m, J = 22.1 Hz, 1H), 2.72 (m, 1H), 2.59 (td, J = 13.5, 2.9 Hz, 3H), 2.47 (dd, J = 14.0, 8.0 Hz, 1H), 2.29 – 2.12 (m, 3H), 2.09 – 1.84 (m, 12H), 1.81 – 1.63 (m, 18H), 1.50 (m, 1H), 1.46 (s, 9H), 1.44 (s, 9H), 1.39 (d, J = 6.8 Hz, 3H), 1.35 – 1.25 (m, 11H), 1.21 – 1.06 (m, 13H), 1.01 (m, 7H), 0.95 (t, J = 7.2 Hz, 3H), 0.90 – 0.84 (m, 4H). **^{13}C NMR** (101 MHz, $CDCl_3$) δ 175.21, 174.88, 174.69, 174.21, 173.72, 173.48, 172.72, 156.29, 128.64, 128.28, 128.03, 79.02, 78.76, 67.15, 53.94, 52.96, 51.75, 51.39, 50.45, 49.82, 49.21, 49.16, 48.99, 45.34, 44.57, 44.06, 39.75, 39.40, 39.25, 38.96, 38.16, 37.54, 37.10, 36.95, 35.93, 35.39, 34.56, 34.45, 34.10, 33.87, 28.71, 28.65, 25.99, 25.65, 25.56,

25.46, 25.36, 25.25, 25.03, 24.90, 24.80, 23.10, 22.98, 22.49, 22.29, 18.42, 17.92, 14.22, 14.02, 13.25, 12.86. **HRMS-ESI** (m/z) calc'd for $C_{79}H_{132}N_{10}O_{15}Na$ $[M+Na]^+$, 1483.9766; found, 1483.9779.

Cbz-L-Ala-AChPA-L-Orn•NH$_2$-AChPA-L-Orn•NH$_2$-AChPA-L-Ala-AChPA-OMe 6

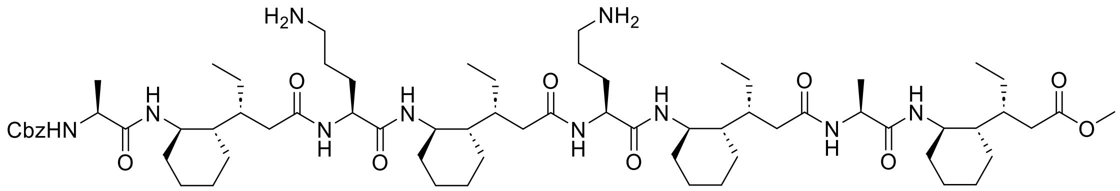

6

Following general procedure F, Foldamer **6** was collected as a white solid.

HPLC analysis: Acetonitrile+0.1% TFA/Water+0.1% TFA, 1.0 mL/min, 25 °C, detection at 210 nm, retention time (min): 22.6.

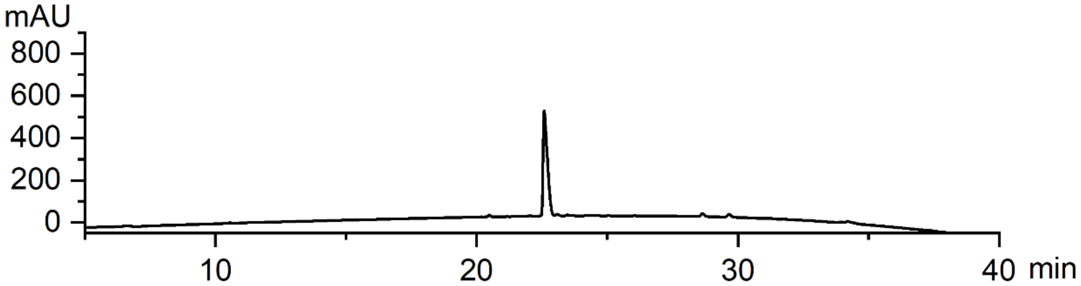

^{1}H NMR spectrum of Foldamer 6 (400 MHz, CDCl3).

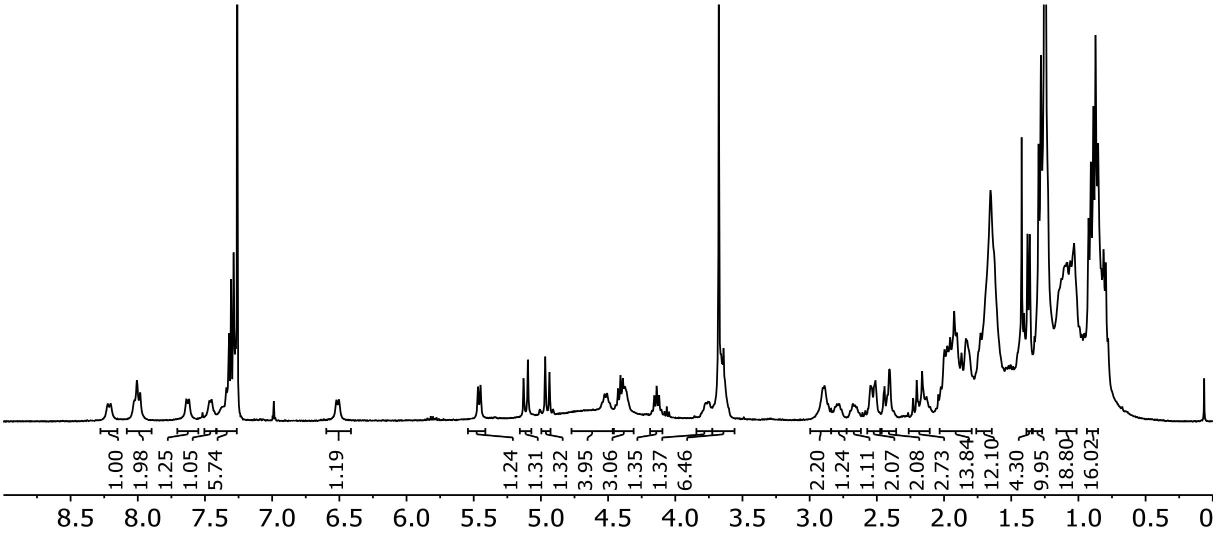

HRMS spectrum of Foldamer 6.

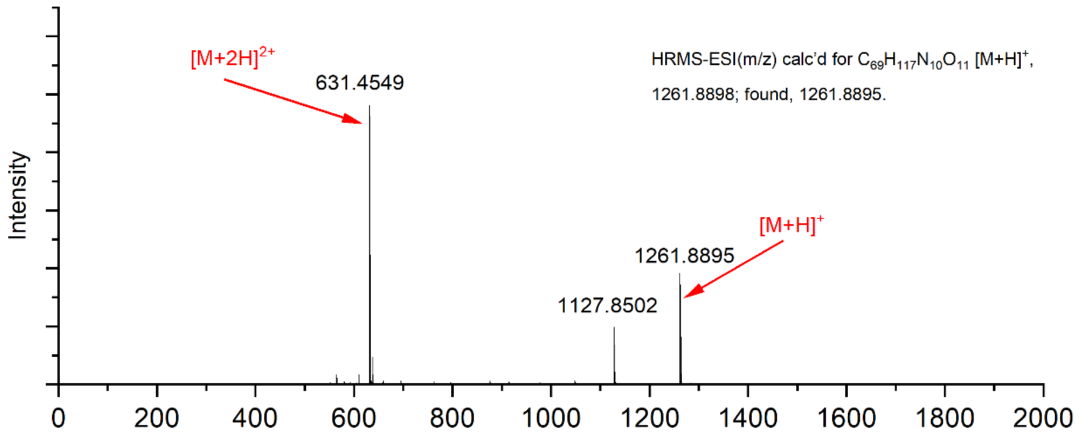

2.4 Preparation of non-catalytic/catalytic Heptamers

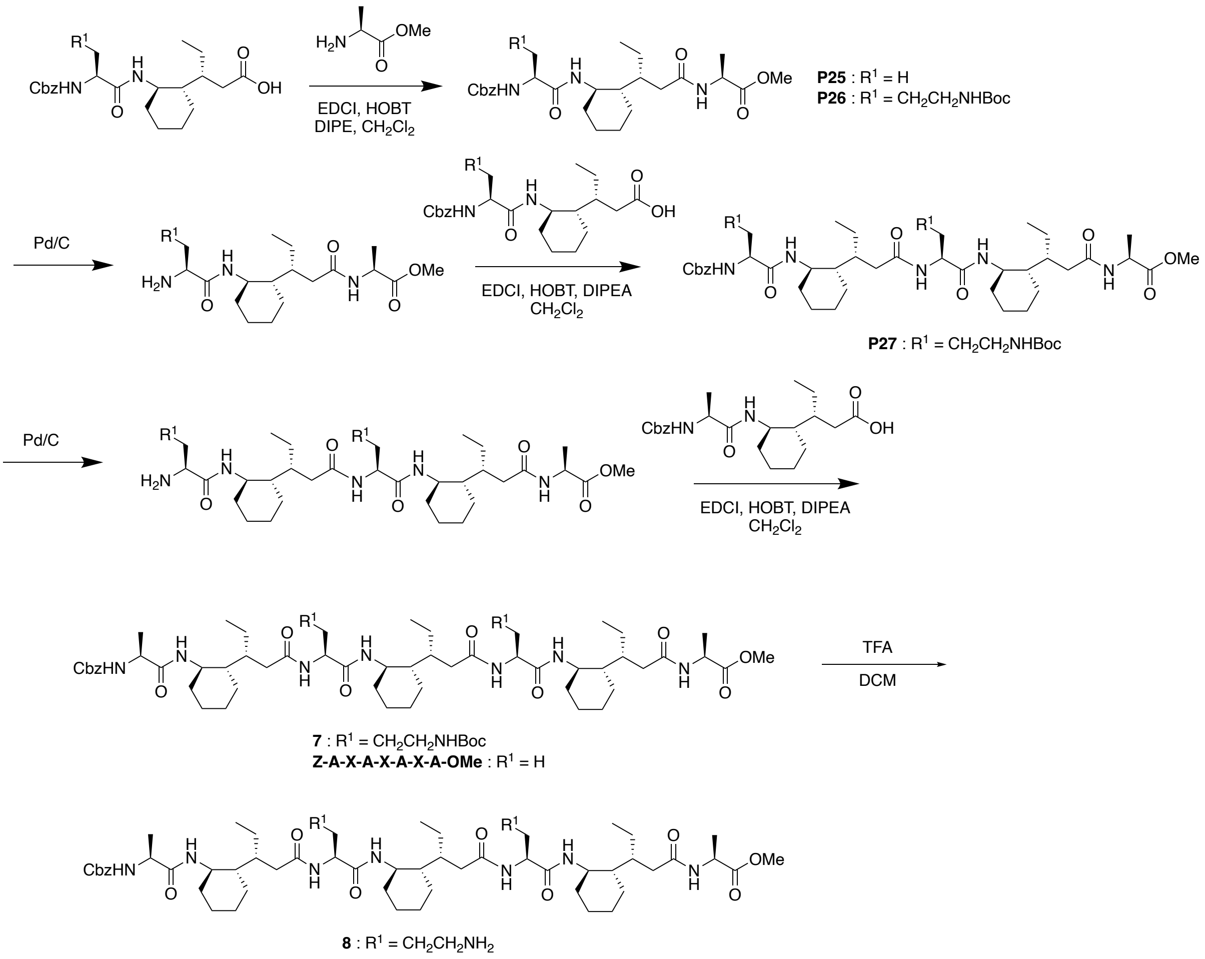

Synthesis of Heptamer Foldamer Z-A-X-A-X-A-X-A-OMe

Cbz-L-Ala-AchPA-L-Ala-Ome P25

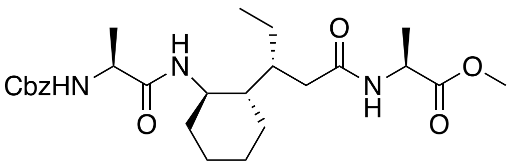

P25

Following general procedure A, **P25** was collected as a white solid. **^{1}H NMR** (400 MHz, $CDCl_3$) δ 7.39 – 7.28 (m, 6H), 7.06 (d, J = 9.8 Hz, 1H), 5.38 (d, J = 7.8 Hz, 1H), 5.09 (d, J = 12.2 Hz, 1H), 4.97 (d, J = 12.2 Hz, 1H), 4.79 – 4.68 (m, 1H), 3.98 (p, J = 7.1 Hz, 1H), 3.75 (m, 4H), 2.47 (dd, J = 13.2, 3.2 Hz, 1H), 1.94 – 1.75 (m, 3H), 1.70 (d, J = 12.8 Hz, 3H), 1.51 (ddt, J = 13.1, 7.9, 6.6 Hz, 1H), 1.36 (dd, J = 7.3, 6.3 Hz, 6H), 1.20 – 1.08 (m, 4H), 1.01 (td, J = 12.3, 3.0 Hz, 1H), 0.85 (ddt, J = 12.4, 9.4, 6.3 Hz, 1H), 0.76 (t, J = 7.1 Hz, 3H). **^{13}C NMR** (101 MHz, $CDCl_3$) δ 175.84, 173.68, 172.47, 156.26, 136.21, 128.63, 128.36, 128.21, 67.19, 52.64, 51.26, 49.14, 47.40, 44.68, 38.67, 37.15, 34.15, 25.74, 25.55, 24.58, 22.20, 18.15, 17.56, 12.79. **HRMS-ESI** (m/z) calc'd for $C_{26}H_{39}N_3O_6Na$ $[M+Na]^+$, 512.2732; found, 512.2770.

Cbz-L-Ala-ACPA-L-Ala-ACPA-L-Ala-ACPA-L-Ala-Ome Z-A-X-A-X-A-X-A-Ome

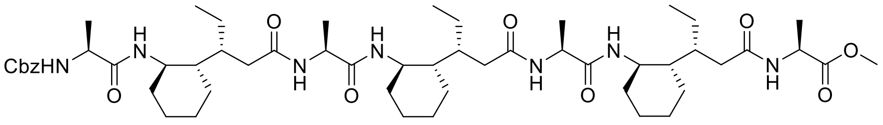

Following general procedure A, the non-catalytic heptameric foldamer was collected as a white solid.

^{1}H NMR (400 MHz, $CDCl_3$) δ 8.05 (d, J = 5.6 Hz, 1H), 7.99 (d, J = 9.5 Hz, 1H), 7.86 (d, J = 7.4 Hz, 2H), 7.67 (d, J = 5.6 Hz, 1H), 7.38 – 7.25 (m, 6H), 5.37 – 5.20 (m, 1H), 5.12 (d, J = 12.2 Hz, 1H), 4.95 (d, J = 12.3 Hz, 1H), 4.65 (p, J = 7.5 Hz, 1H), 4.30 (q, J = 6.7 Hz, 1H), 4.09 (m, 2H), 3.75 (m, 6H), 2.66 – 2.47 (m, 3H), 2.15 – 1.83 (m, 9H), 1.67 (m, 9H), 1.48 (m, 3H), 1.43 – 1.06 (m, 27H), 1.04 – 0.76 (m, 12H). **^{13}C NMR** (101 MHz, $CDCl_3$) δ 176.58, 174.51, 174.34, 173.91, 173.74, 156.12, 136.29, 128.59, 128.24, 128.06, 66.98, 52.66, 50.48, 50.41, 49.12, 49.03, 47.93, 44.91, 44.46, 44.10, 39.57, 39.39, 38.90, 37.46, 36.74, 36.65, 34.40, 34.05, 33.88, 25.68, 25.51, 25.47, 25.38, 25.30, 25.23, 25.02, 24.85, 24.77, 23.01, 18.38, 17.67, 17.34, 16.76, 14.04, 13.96, 12.84. **HRMS-ESI** (m/z) calc'd for $C_{54}H_{88}N_7O_{10}$ $[M+H]^+$, 994.6588; found, 994.6590.

Synthesis of Foldamer 7/Foldamer 8

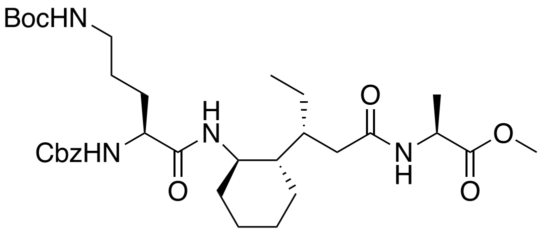

P26

Following general procedure A, **P26** was collected as a white solid. **^{1}H NMR** (400 MHz, $CDCl_3$) δ 7.39 – 7.28 (m, 5H), 7.18 (d, J = 9.7 Hz, 1H), 5.34 (d, J = 8.3 Hz, 1H), 5.09 (d, J = 12.2 Hz, 1H), 4.97 (d, J = 12.2 Hz, 1H), 4.77 (p, J = 7.6 Hz, 1H), 4.59 (s, 1H), 3.90 (q, J = 7.5 Hz, 1H), 3.75 (s, 4H), 3.13 (p, J = 6.9 Hz, 2H), 2.47 (dd, J = 13.4, 3.4 Hz, 1H), 2.00 – 1.51 (m, 6H), 1.50 – 1.32 (m, 12H), 1.25 – 1.07 (m, 4H), 1.06 – 0.93 (m, 1H), 0.92 – 0.81 (m, 1H), 0.76 (t, J = 7.1 Hz, 3H). **^{13}C NMR** (101 MHz, $CDCl_3$) δ 176.03, 173.58, 171.42, 156.42, 156.05, 136.10, 128.66, 128.42, 128.27, 79.40, 67.31, 55.38, 52.74, 49.18, 47.37, 44.57, 40.00, 38.72, 37.25, 34.30, 29.28, 28.52, 26.55, 25.71, 25.50, 24.58, 22.21, 17.63, 12.87. **HRMS-ESI** (m/z) calc'd for $C_{33}H_{53}N_4O_8$ $[M+H]^+$, 633.3858; found, 633.3845.

Cbz-L-Orn•O^{t}Bu-AchPA-L-Orn•O^{t}Bu-AchPA-L-Ala-Ome

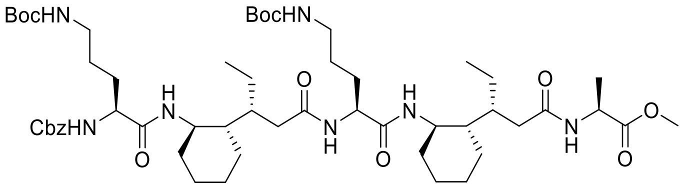

P27

Following general procedure A, **P27** was collected as a white solid. **^{1}H NMR** (400 MHz, $CDCl_3$) δ 7.88 (d, J = 9.5 Hz, 1H), 7.61 (d, J = 7.5 Hz, 1H), 7.46 (d, J = 6.4 Hz, 1H), 7.40 (d, J = 9.7 Hz, 1H), 7.32 (m, 5H), 5.29 (d, J = 8.0 Hz, 1H), 5.08 (m, 2H), 4.99 (d, J = 12.3 Hz, 1H), 4.76 (b, 1H), 4.67 (p, J = 7.8 Hz, 1H), 4.07 (m, 2H), 3.77 (m, 5H), 3.28 – 3.11 (m, 1H), 3.01 (m, 3H), 2.53 (dd, J = 24.3, 10.6 Hz, 2H), 2.00 (d, J = 8.2 Hz, 2H), 1.90 (m, 4H), 1.84 – 1.59 (m, 13H), 1.59 – 1.48 (m, 3H), 1.43 (s, 9H), 1.40 (s, 9H), 1.36 (d, J = 7.5 Hz, 3H), 1.24 – 1.01 (m, 11H), 0.98 (t, J = 7.1 Hz, 3H), 0.94 – 0.87 (m, 1H), 0.87 – 0.78 (m, 3H). **^{13}C NMR** (101 MHz, $CDCl_3$) δ 174.70, 173.92, 173.05, 171.97, 156.87, 156.15, 156.03, 136.21, 128.66, 128.32, 127.97, 79.21, 67.11, 54.49, 52.85, 49.44, 49.09, 47.99, 44.53, 44.37, 39.97, 39.28, 39.10, 38.94, 37.72, 37.11, 34.47, 33.87, 29.12, 28.88, 28.60, 28.55, 26.81, 25.64, 25.46, 25.39, 25.28, 25.07, 24.80, 23.08, 22.44, 16.99, 14.08, 12.95. **HRMS-ESI** (m/z) calc'd for $C_{54}H_{90}N_7O_{12}$ $[M+H]^+$, 1028.6642; found, 1028.6621.

Cbz-L-Ala-AchPA-L-Orn•O^{t}Bu-AchPA-L-Orn•O^{t}Bu-AchPA-L-Ala-Ome 7

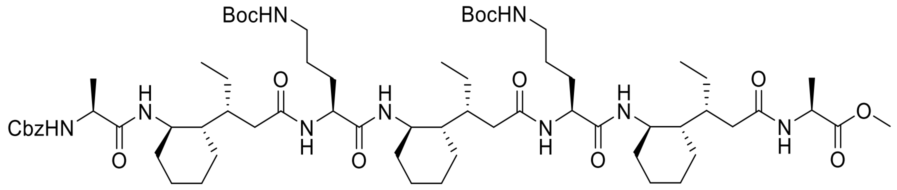

Following general procedure A, foldamer **7** was collected as a white solid. **^{1}H NMR** (400 MHz, $CDCl_3$) δ 7.95 (t, J = 8.9 Hz, 2H), 7.79 (d, J = 6.2 Hz, 1H), 7.70 (d, J = 7.6 Hz, 1H), 7.62 (d, J = 6.3 Hz, 1H), 7.43 (d, J = 9.8 Hz, 1H), 7.39 – 7.27 (m, 5H), 5.47 (b, 1H), 5.26 (d, J = 7.3 Hz, 1H), 5.07 (d, J = 12.5 Hz, 1H), 5.00 (d, J = 12.3 Hz, 1H), 4.83 (b, 1H), 4.68 (p, J = 7.5 Hz, 1H), 4.27 (b, 1H), 4.05 (tq, J = 6.1, 2.9, 2.4 Hz, 2H), 3.76 (m, 6H), 3.17 (m, 1H), 3.06 (m, 2H), 2.72 (m, 1H), 2.64 – 2.49 (m, 3H), 2.06 – 1.83 (m, 9H), 1.76 – 1.60 (m, 14H), 1.42 (m, 12H), 1.41 (s, 9H), 1.37 (d, J = 6.9 Hz, 3H), 1.34 (d, J = 7.5 Hz, 3H), 1.25 – 0.96 (m, 21H), 0.90 – 0.81 (m, 9H). **^{13}C NMR** (101 MHz, $CDCl_3$) δ 176.93, 175.23, 174.63, 173.93, 173.61, 173.37, 172.73, 156.30, 156.24, 156.07, 136.15, 128.64, 128.28, 127.97, 79.27, 78.88, 77.48, 77.16, 76.84, 67.10, 54.96, 53.07, 52.82, 51.34, 49.42, 49.11, 48.99, 47.96, 44.65, 44.60, 44.09, 40.07, 39.65, 39.32, 38.91, 38.42, 37.53, 37.11, 37.04, 34.44, 34.07, 33.85, 31.72, 29.83, 28.77, 28.67, 28.57, 27.67, 27.13, 25.80, 25.61, 25.52, 25.43, 25.31, 25.24, 25.01, 24.88, 24.77, 23.06, 23.01, 22.78, 22.46, 18.40, 16.93, 14.25, 14.19, 14.03, 12.86, -3.43. **HRMS-ESI** (m/z) calc'd for $C_{68}H_{114}N_9O_{14}$ $[M+H]^+$, 1280.8480; found, 1280.8710

Cbz-L-Ala-AchPA-L-Orn•NH$_2$-AchPA-L-Orn•NH$_2$-AchPA-L-Ala-Ome 8

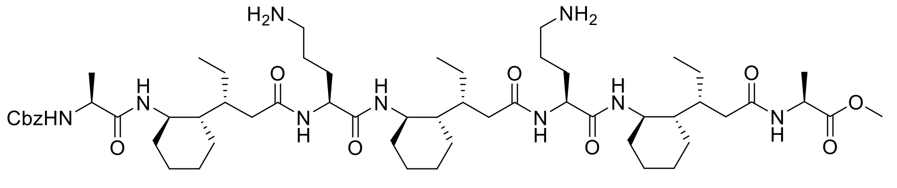

Following general procedure F, Foldamer **8** was collected as a white solid. **^{1}H NMR** (400 MHz, $CDCl_3$) □ 7.97 (m, 2H), 7.76 (d, J = 6.2 Hz, 1H), 7.66 (m, 2H), 7.43 (d, J = 9.7 Hz, 1H), 7.35 – 7.27 (m, 5H), 5.53 (d, J = 7.5 Hz, 1H), 5.14 – 4.93 (m, 2H), 4.65 (p, J = 7.5 Hz, 1H), 4.37 (m, 1H), 4.30 – 4.11 (b, 4H), 4.03 (m, 2H), 3.73 (m, 6H), 2.76 (m, 2H), 2.66 (m, 2H), 2.59 – 2.36 (m, 3H), 2.01 – 1.80 (m, 9H), 1.78 – 1.59 (m, 14H), 1.57 – 1.41 (m, 6H), 1.34 (t, J = 8.6, 7.2 Hz, 6H), 1.21 – 0.96 (m, 16H), 0.96 – 0.75 (m, 11H). **HRMS-ESI** (m/z) calc'd for $C_{58}H_{98}N_9O_{10}$ $[M+H]^+$, 1080.7432; found, 1080.7389

HPLC analysis: Acetonitrile+0.1% TFA/Water+0.1% TFA, 1.0 mL/min, 25 °C, detection at 210 nm, retention time (min): 18.2.

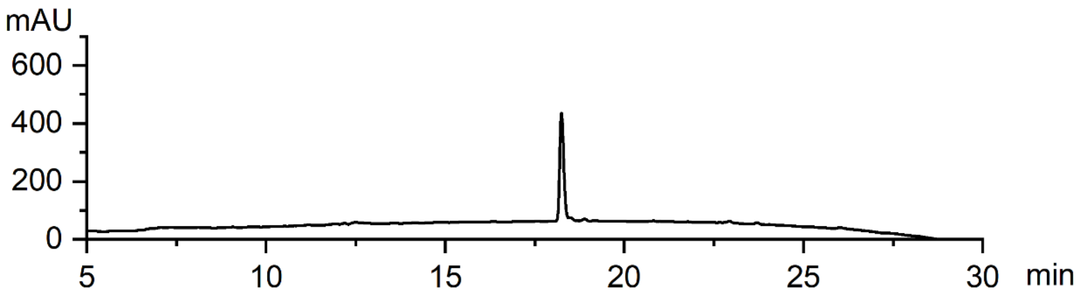

^{1}H NMR spectrum of Foldamer 8 (400 MHz, $CDCl_3$)

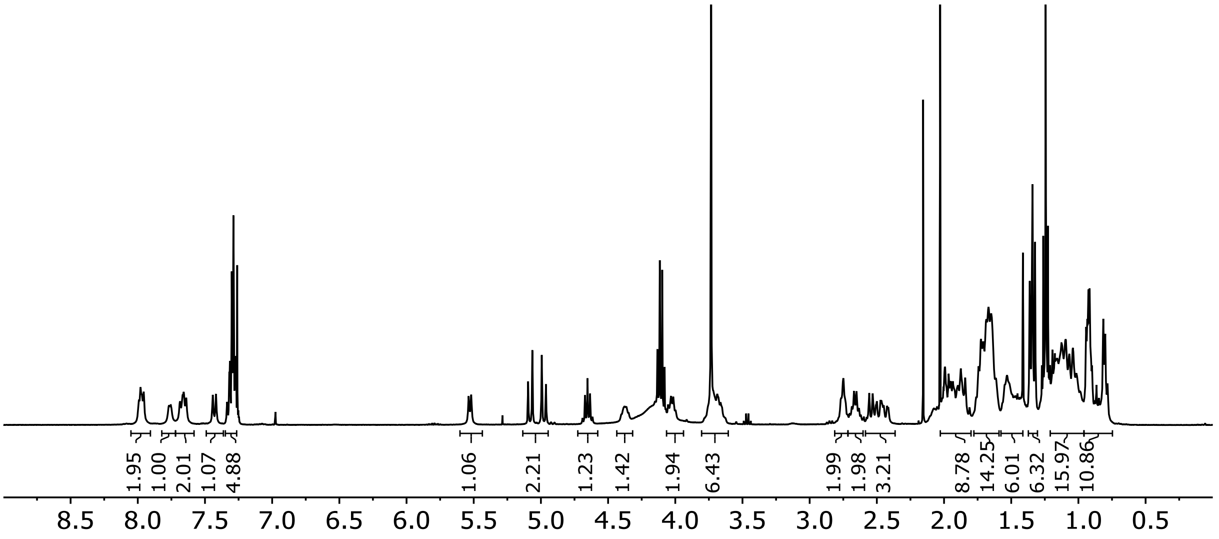

HRMS spectrum of Foldamer 8.

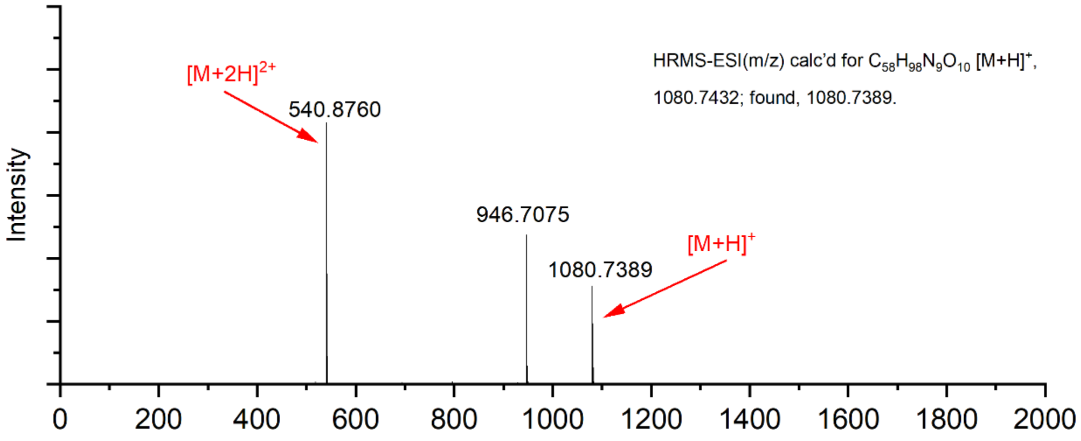

Synthesis of Tripeptide 9

Cbz-L-Boc•NH_2-ACPA-L-Ala-Ome

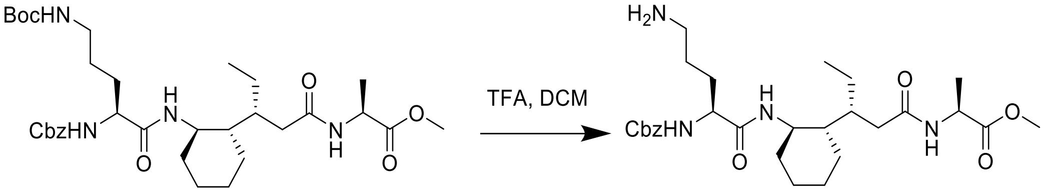

P26 **9**

Following general procedure F, tripeptide **9** was collected as a white solid.

HPLC analysis: Acetonitrile+0.1% TFA/Water+0.1% TFA, 1.0 mL/min, 25 °C, detection at 210 nm, retention time (min): 16.7.

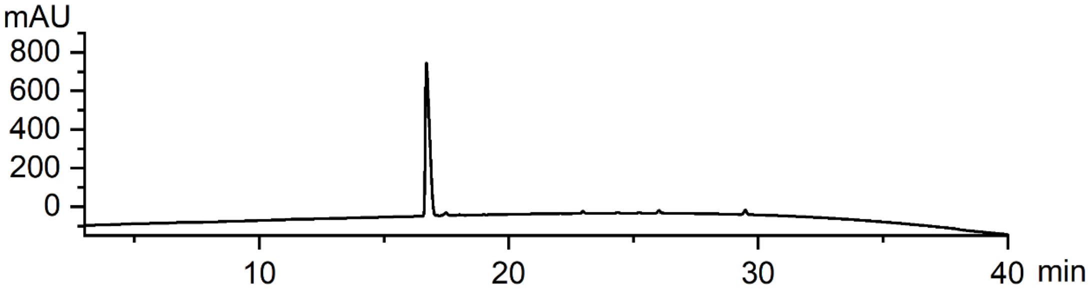

^{1}H NMR spectrum of Tripeptide 9 (400 MHz, $CDCl_3$).

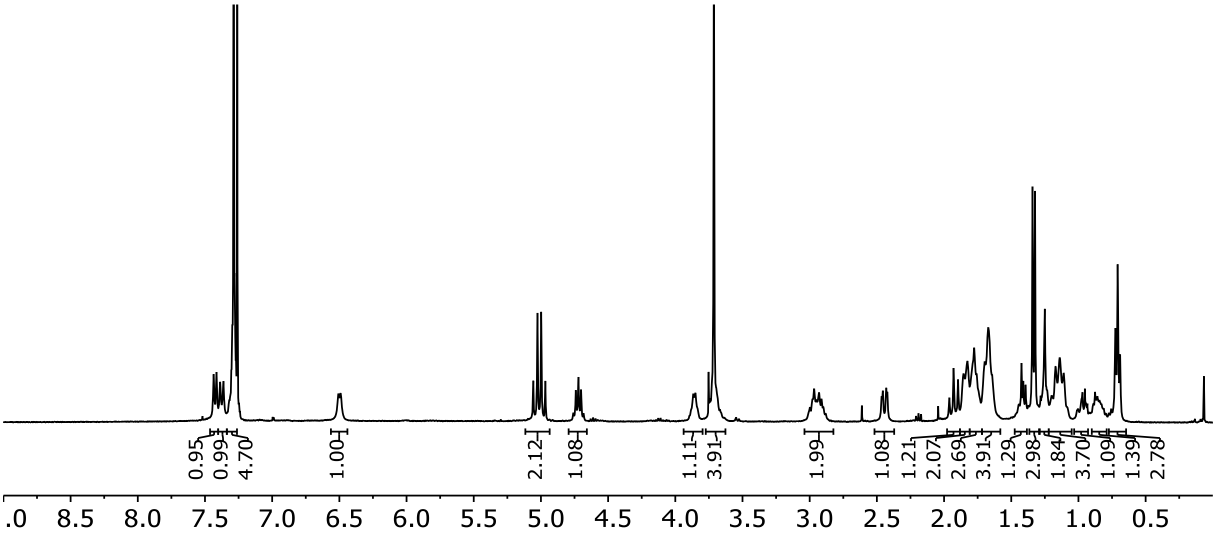

HRMS spectrum of Tripeptide 9.

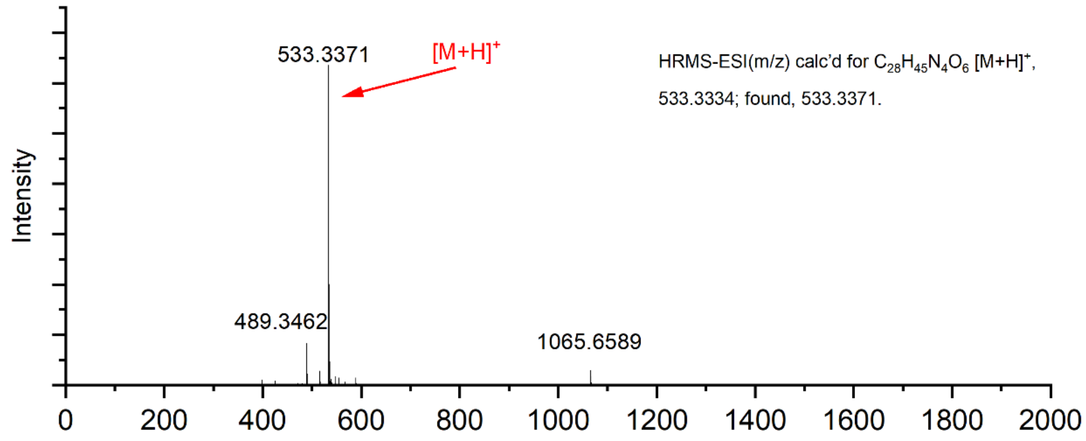

2.5 Characterization of non-catalytic foldamers

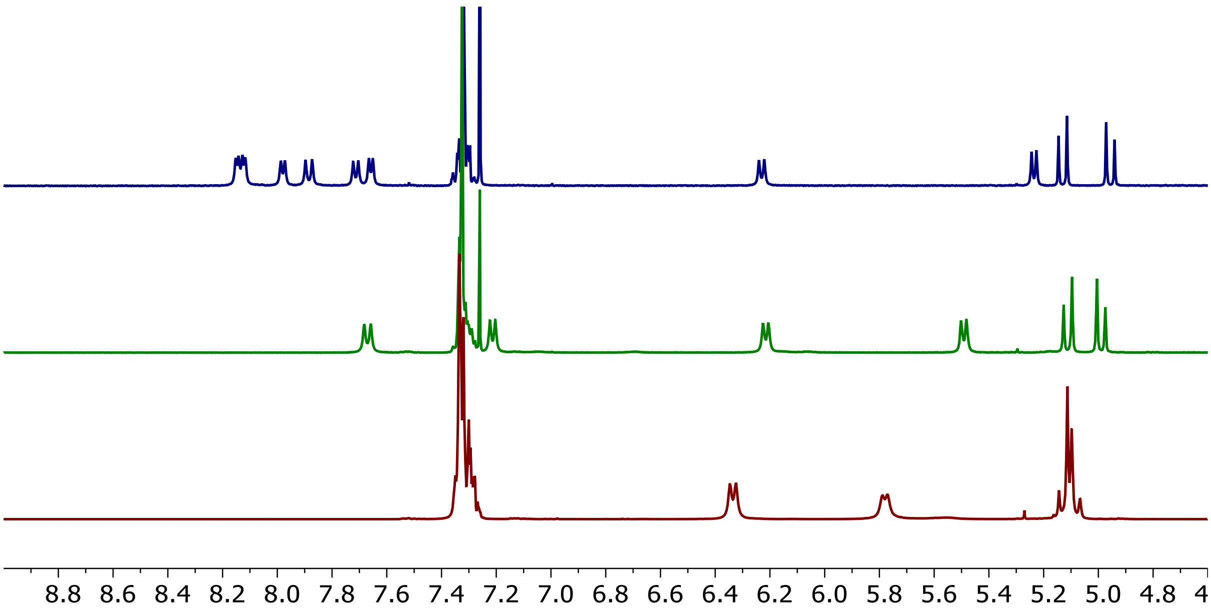

Figure SI1. Stacked ^{1}H NMR spectrum of L-alanine constructed foldamers in $CDCl_3$ from 4.5-9 ppm. Red: **P11**(Dimer)**;** Green:**P12**(Tetramer); Blue: Foldamer **2**(Octamer).

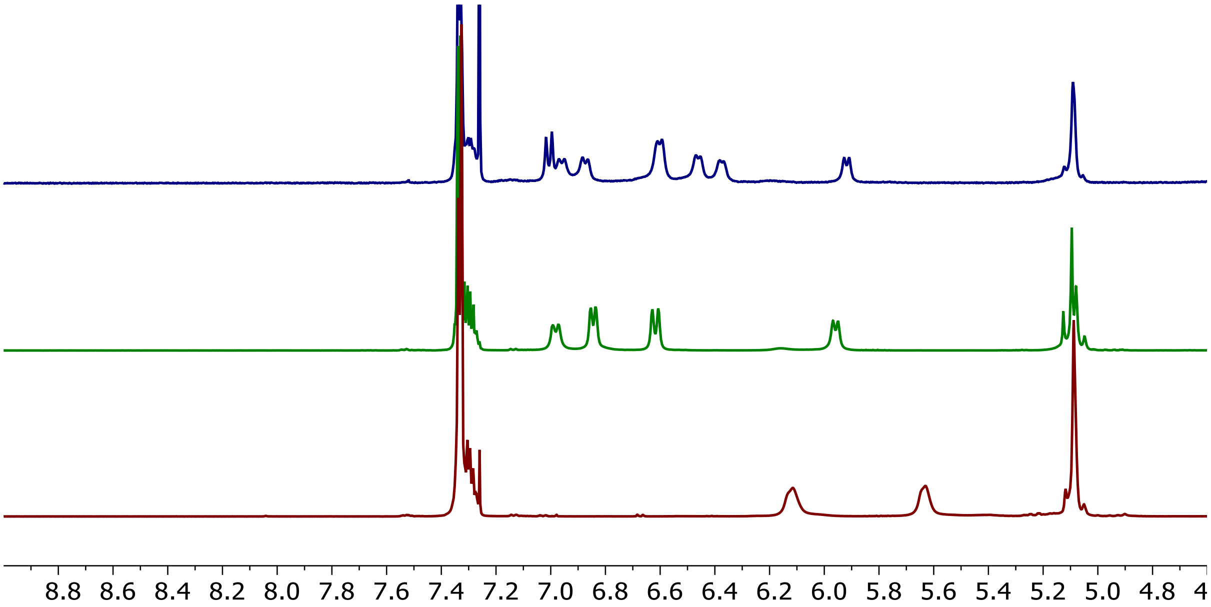

Figure SI2. Stacked ^{1}H NMR spectrum of (D)-alanine constructed foldamers in $CDCl_3$ from 4.5-9 ppm. Red: **P17**(Dimer)**;** Green:**P18**(Tetramer); Blue: Foldamer **3**(Octamer)..

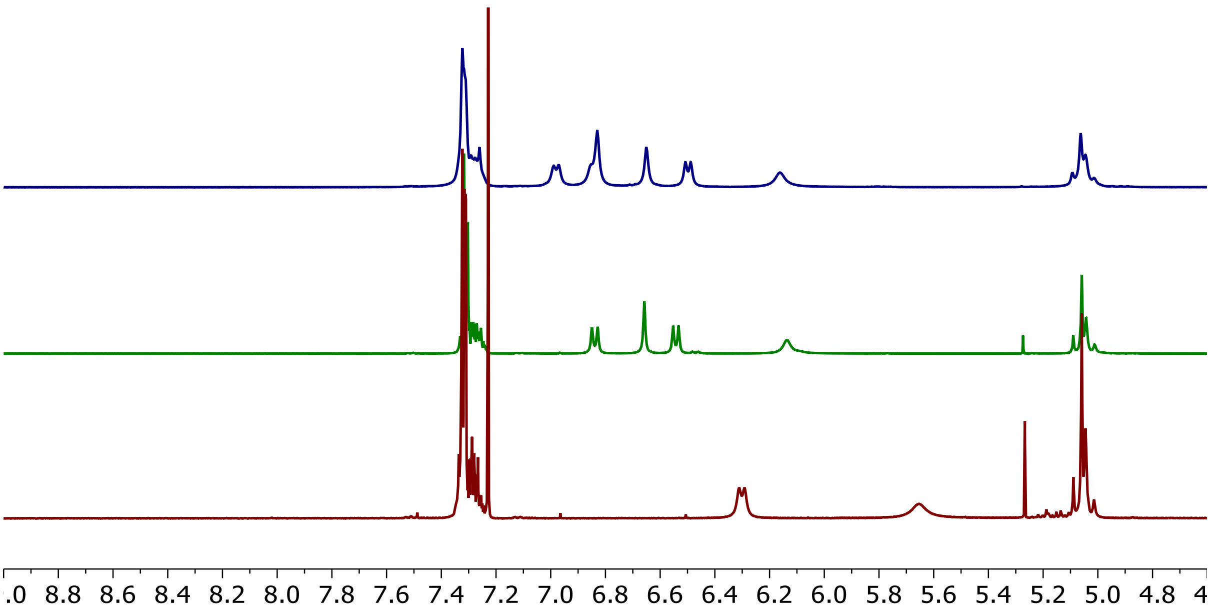

Figure SI3. Stacked ^{1}H NMR spectrum of Aib-amino acid constructed foldamers in $CDCl_3$.
Red: **P23** (Dimer)**;** Green:**P24** (Tetramer); Blue: Foldamer **4** (Hexamer).

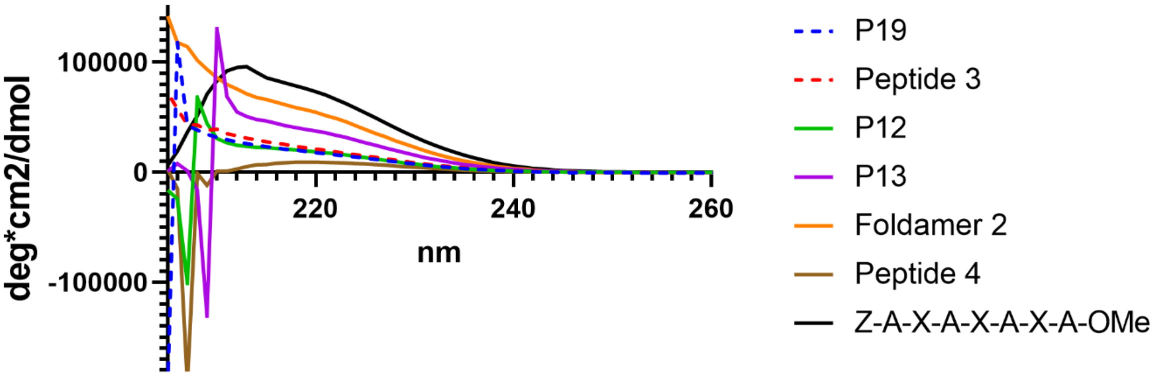

Figure SI4: CD Spectrum of foldamers in MeOH.

3.0 Retro-aldol cleavage in chloroform

1. **Kinetic of retro-aldol reaction in $CDCl_3$ monitored by ^{1}H NMR**

   To a NMR tube, 50.2 uL of methodol（149mM in $CDCl_3$）was added into 368.8 μL $CDCl_3$, the solution was mixed thoroughly, 81 μL of foldamer catalyst (4.63 mM in $CDCl_3$) was added.

   For systems exhibiting burst-phase kinetics, time courses were fit to equation 1.[1]

$$[P] = [E]_0 \left(\frac{k'_1}{k'_1+k_2}\right)\left(\frac{k'_1}{k'_1+k_2}\{1 - e^{-(k'_1+k_2)t}\} + k_2 t\right) \qquad \text{Eq 1,}$$

   where $k'_1 = k_1[Aldol]_0$ and $[E]_0 = 0.75\ mM$. For control amines, a linear equation (Eq 2) was used:

$$[P] = k'_1[E]_0 t \qquad \text{Eq 2,}$$

   Where $k'_1 = k_1[Aldol]_0$ and $[E]_0 = 1.5\ mM$.

2. **Aldolase activity of foldamer 8 in $CHCl_3$ at room temperature.**

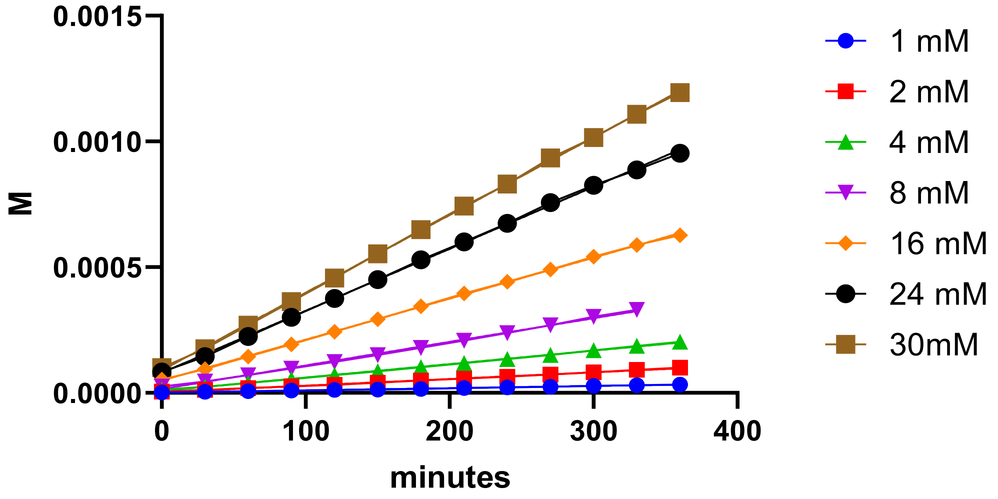

Figure SI6. Rates for retro-aldol reaction of 1~30 mM substrate with 0.375 mM foldamer **8** in 100 uL $CHCl_3$, monitored by HPLC every 30 minutes.

3. **Trapping of intermediates**

15 mM of methodol was reacted with 0.75 mM of Foldamer **8** in 500 μL of $CHCl_3$ for 2 hours, 1 mg of $Na(CH_3COO)_3BH$ (10 mM) was added, the mixture was reacted overnight at room temperature. The reduced intermediates were analysed by HRMS.

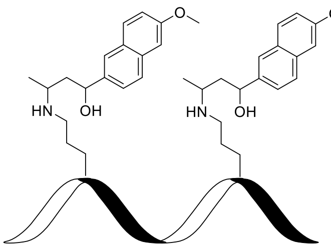

FMM $[M+H]^+$:1536.97
$[M+2H]^{2+}$:768.99

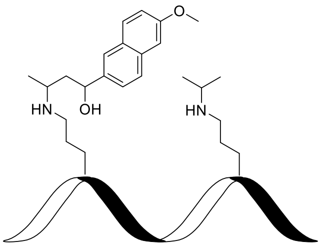

FMK $[M+H]^+$:1350.91
$[M+2H]^{2+}$:675.96

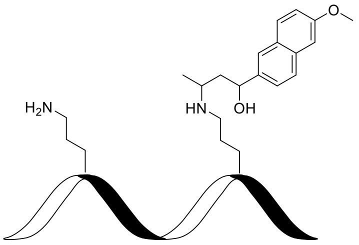

FM $[M+H]^+$:1308.86
$[M+2H]^{2+}$:654.93

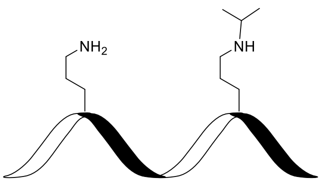

FK $[M+H]^+$:1122.79
$[M+2H]^{2+}$:561.90

Figure SI7. The structure of intermediate and its calculated M/Z

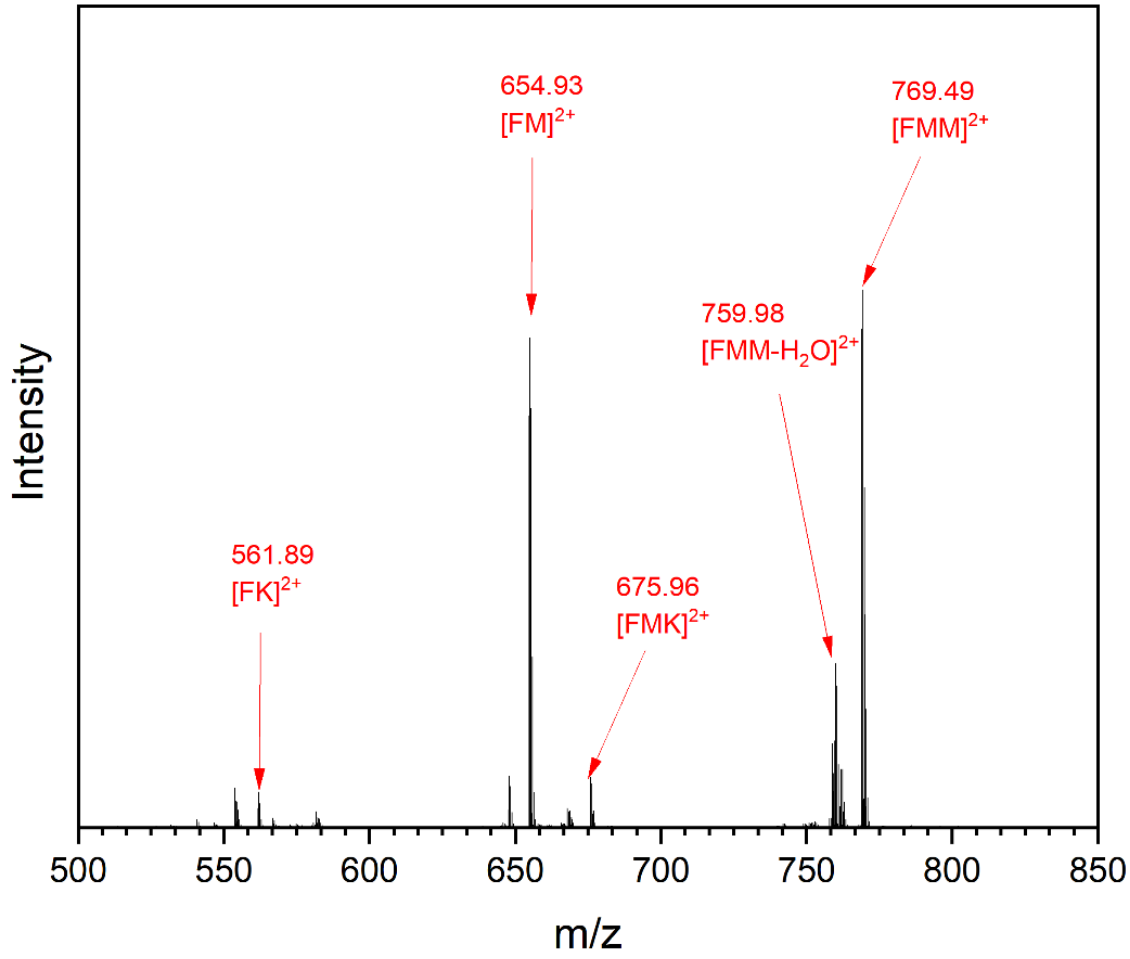

Figure SI8. Observed m/z of reduced imine intermdiates.

(1) Fersht, Structure and Mechanism in Protein Science, ISBN: 978-981-3225-18-3

4.0 Modelling Studies

4.1 Monomer Conformation Predictions

The cyclohexyl δ-residue 1 was shown to comfortably adopt a conformation that would lead to the most stable 13/11-helix according to calculations.

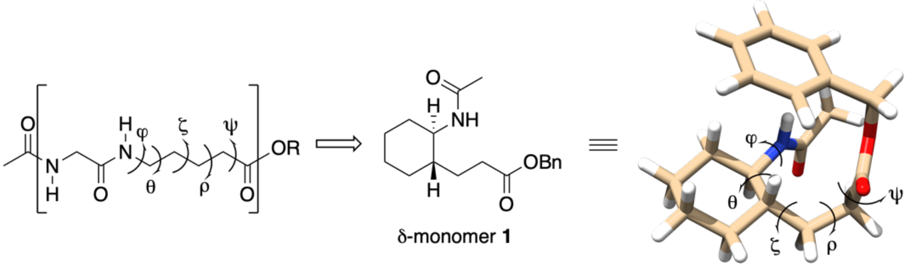

	φ	θ	ζ	ρ	ψ
Proposed angles for a 13,11-helix	-135.6	-72.4	66.9	54.9	-139.3
Low energy conformation of monomer **1**	111.1	-63.6	73.2	48.5	-125.0

4.2 Density functional theory calculations

Methodology

Geometry optimizations were performed at the level of density functional theory (DFT) in two steps using the all-electron DFT-Code FHI-aims.[1] First optimization was performed with the generalized gradient approximation PBE functional[2] employing tier-1 basis sets and light computational settings.[1] The final optimization was performed with the PBE0 hybrid functional,[3] tier-2 basis sets, and tight computational settings.[1] Since DFT at these levels of approximation lacks the description of long-range dispersion, the pairwise Tkatchenko-Scheffler van der Waals scheme[4] was applied at both optimization steps. This selection of methodology has proven reliable for the description of peptide systems.[5]

(1) Blum, V.; Gehrke, R.; Hanke, F.; Havu, P.; Havu, V.; Ren, X.; Reuter, K.; Scheffler, M. Ab initio Molecular Simulations with Numeric Atom-centered Orbitals. *Comp. Phys. Commun.* **2009**, *180*, 2175.
(2) Perdew, J.; Burke, K.; Ernzerhof, M. Generalized Gradient Approximation Made Simple. *Phys. Rev. Lett.* **1996**, 77, 3865.
(3) Adamo, C.; Barone, V. Toward Reliable Density Functional Methods without Adjustable Parameters: The PBE0 Model. *J. Chem. Phys.* **1999**, *110*, 6158.
(4) Tkatchenko, A.; Scheffler, M. Accurate Molecular van der Waals Interactions from Ground-State Electron Density and Free-Atom Reference Data. *Phys. Rev. Lett.* **2009**, *102*, 073005.
(5) Baldauf, C.; Rossi, M.: Going clean: structure and dynamics of peptides in the gas phase and paths to solvation. *J. Phys. Cond. Matter.* **2015**, *27*, 493002.

Comparison of the backbone torsion angles of the two monomers in the crystal cell arising from X-ray and NMR in solution with those of the two most stable 13/11-helices of α,δ-helices predicted by theory

The backbone torsion angles of the two monomers in the crystal cell determined by X-ray analysis and determined by NMR for the solution state can be compared with those for the theoretically predicted most stable 13/11-helices of α,δ-peptides in Table SI1.

Table SI1. Backbone torsion angle values of the L-Ala and δ-amino acid residues in the two octamers from X-ray[a] and NMR compared with the corresponding theoretical data for alternating α,δ-octamers with unsubstituted backbone

Amino acid	Method	Monomer[b]	ϕ	θ	ξ	ρ	ψ
L-Ala	X-ray	1	-71.2				147.5
δ-amino acid	X-ray	1	117.2	-57.6	150.3	-62.0	-61.0
L-Ala	X-ray	2	-77.6				146,7
δ-amino acid	X-ray	2	123.6	-59.7	149.9	-60.2	-57.2
δ-amino acid[c]	X-ray	1	81.5	-159.9	53.8	-175.8	-121.6
δ-amino acid[c]	X-ray	2	110.7	-50.0	-69.5	-85.8	-152.8
L-Ala	NMR		-72.5				149.8
δ-amino acid[d]	NMR		109.7	-56.6	146.9	-62.0	-60.6
Gly	Theory[e]		-69.3				140.4
δ-amino acid	Theory[e]		115.6	-63.9	149.4	-57.9	-59.9
Gly	Theory[f]		-69.4				149.7
δ-amino acid	Theory[f]		135.5	-77.3	63.7	56.9	-140.0

[a] Angles in degrees, averaged over the torsion angle values of the residues 3, 5, and 7 for L-Ala and 2, 4, and 6 for the δ-amino acid constituent [b] Monomer 1: *bis*-axial connection of the cyclohexyl residue in the last δ-amino acid constituent of the octamer; Monomer 2: cyclohexyl residues of all δ-amino acid constituents connected in *bis*-equatorial orientation. [c] last δ-amino acid residue in the two octamers. [d] According to NMR all cyclohexyl residues are in equatorial connection with the backbone. [e] B3LYP/6-31G* level of *ab initio* MO theory for the second best 13/11-helix in alternating α,δ-amino acid octamers with unsubstituted backbone [Ref. 9]. [f] B3LYP/6-31G* level of *ab initio* MO theory for the most stable 13/11-helix in alternating α,δ-amino acid octamers with unsubstituted backbone [Ref. 9].

Calculations on various 13/11-helices of α,δ-hybrid helices with varying backbone substitution

The helix structure found in the X-ray and NMR structure analyses was not the most stable 13/11-helix predicted by theory for α,δ-hybrid helices. Interestingly, the theoretical predictions were confirmed by NMR data for α,δ-hybrid peptides bearing voluminous substituents only in δ-position of the δ-amino acid constituents [Ref. 9]. It seemed to be interesting to find reasons for this stability change.

Table SI2. Average values for the backbone torsion angles[a] in octamers of α,δ-hybrid helices composed of L-Ala and various δ-amino acid constituents in alternating order and energy differences between the helix alternatives I and II

α-/δ-amino acid	Helix[b]	ϕ	θ	ξ	ρ	ψ	ΔE[c]
L-Ala	I	-70.0				148,2	-21.8
	II	-72.3				138.7	
unsubstituted	I	133.2	-76.2	66.0	55.0	-139.1	
	II	114.2	-61.7	151.0	-55.2	-62.7	
L-Ala	I	-71.3				144.8	+25.2
	II	-71.8				140.2	
β-ethyl	I	128.3	-74.6	51.0	69.5	-137.8	
	II	109.7	-60.4	149.7	-54.4	-64.1	
L-Ala	I	-76.3				141.3	+5.6
	II	-76.2				131.6	
γ,δ-cyclohexyl	I	123.9	-64.3	73.4	45.5	-133.2	
	II	113.3	-57.6	154.4	-51.1	-65.9	
L-Ala	I	-77.7				144.0	+60.2
	II	-67.7				144.9	
β-ethyl-γ,δ-cyclohexyl	I	117.6	-60.5	71.6	42.0	-140.7	
	II	115.9	-55.8	147.6	-63.6	-59.1	
L-Ala	I	-67.7				146.2	-13.5
	II	-76.2				128.7	
δ-ethyl	I	141.3	-79.6	62.9	56.0	-138.7	
	II	112.4	-56.7	152.7	-52.3	-63.6	

[a] In degrees, averaged over the torsion angle values of residues 3, 5, and 7 for L-Ala and 2, 4, and 6 for the δ-amino acid constituent. [b] Helix I: most stable 13/11-helix in unsubstituted α,δ-hybrid peptides according to *ab initio* MO theory [Ref. 9]; Helix II: second best 13/11-helix in unsubstituted α,δ-hybrid peptides according to *ab initio* MO theory [Ref. 9].[c] Energy difference between helices I and II in kJ/mol.

Therefore, calculations on the two competitive 13/11-helices I and II were performed with stepwise substitution on the various backbone positions. Helix I backbone corresponds to the helix predicted as most stable for the unsubstituted backbone in [Ref. 9], whereas helix II backbone corresponds to the structure found in this study. The terminal groups of the sequences are again acetyl and N-methyl.
Table SI2 shows average values for the backbone torsion angles of the various helices. The energy differences between the helix alternatives I and II in Table SI2 clearly show the influence of substitution on helix stability and indicate the stability change in favour of helix II with the substitution pattern in

this study. The considerable stabilization of helix type II in case of the triply-substituted backbone can well be understood due to possible apolar interactions between ethyl and cyclohexyl residues. It can be seen that substitution only in δ-position (δ-ethyl) leaves helix I more stable than helix II as it was indeed experimentally found [Ref. 9]. The structure of the helices in Tabel S2 can be obtained from the NOMAD Repository and Archive. The individual calculations, together with a download link, are listed here:

1. α,δ-hybrid peptide, 13/11-helix type I, Ac-(L-Ala-Daa)4-NMe, unsubstituted δ-amino acid, NOMAD ID: zDUTPJdphjqiF0coi5fsDvqXfoQg
2. α,δ-hybrid peptide, 13/11-helix type II, Ac-(L-Ala-Daa)4-NMe, unsubstituted δ-amino acid, NOMAD ID: 1p9cDOF3Q1DrH9yDMnIhVOvenwHS
3. α,δ-hybrid peptide, 13/11-helix type I, Ac-(L-Ala-Daa)4-NMe, β-ethyl-substituted δ-amino acid, NOMAD ID: lmd4jQwc_Sx6rxt0qT-hQ2OL8KY_
4. α,δ-hybrid peptide, 13/11-helix type II, Ac-(L-Ala-Daa)4-NMe, β-ethyl-substituted δ-amino acid, NOMAD ID: LYWKBnw-74XfnM3CS1ze-QyESitr
5. α,δ-hybrid peptide, 13/11-helix type I, Ac-(L-Ala-Daa)4-NMe, γ,δ-cyclohexyl δ-amino acid. NOMAD ID: TkjzamErFrdPtR-qNRIM5g2ty0IL
6. α,δ-hybrid peptide, 13/11-helix type II, Ac-(L-Ala-Daa)4-NMe, γ,δ-cyclohexyl δ-amino acid, NOMAD ID: Eb1l2KHdLzIfnXHf1FwVMIOtwi4n
7. α,δ-hybrid peptide, 13/11-helix type I, Ac-(L-Ala-Daa)4-NMe, β-ethyl-substituted-γ,δ-cyclohexyl δ-amino acid, NOMAD ID: Cd24D45A370XtRrN7YHIEESquyEm
8. α,δ-hybrid peptide, 13/11-helix type II, Ac-(L-Ala-Daa)4-NMe, β-ethyl-substituted-γ,δ-cyclohexyl δ-amino acid, NOMAD ID: I88eWEcYlBcQO-dF5xE5jfXfcES9
9. α,δ-hybrid peptide, 13/11-helix type I, Ac-(L-Ala-Daa)4-NMe, δ-ethyl-substituted δ-amino acid, NOMAD ID: RjXDSv-rPGwPitqX1rpXrqPuqlxB
10. α,δ-hybrid peptide, 13/11-helix type II, Ac-(L-Ala-Daa)4-NMe, δ-ethyl-substituted δ-amino acid, NOMAD ID: eTRQco1ltKzY1eIfgjKZYuKQnq7h

The data can also be downloaded as a set under this ID: oGrna3QYSauqOhIt8vBnyw

5.0 NMR spectra of all products

CDCl$_3$ ^{1}H (400 MHz) and ^{13}C (101 MHz) of **P1**

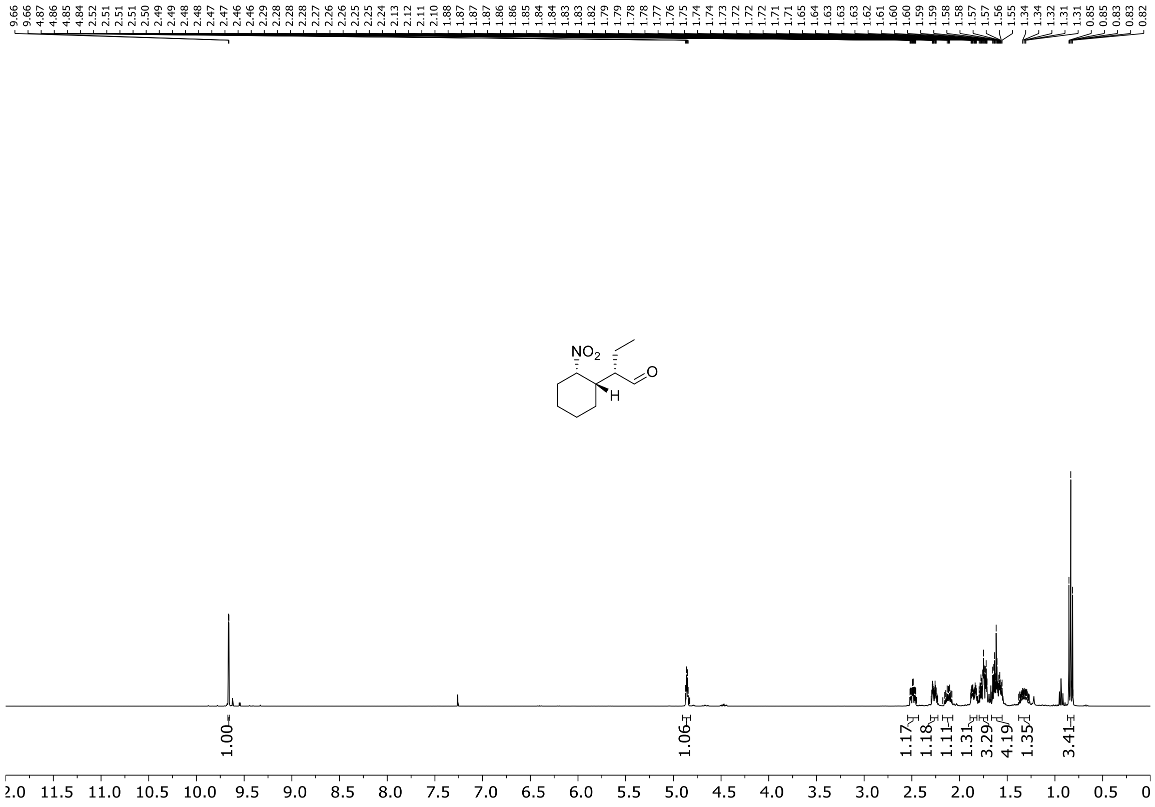

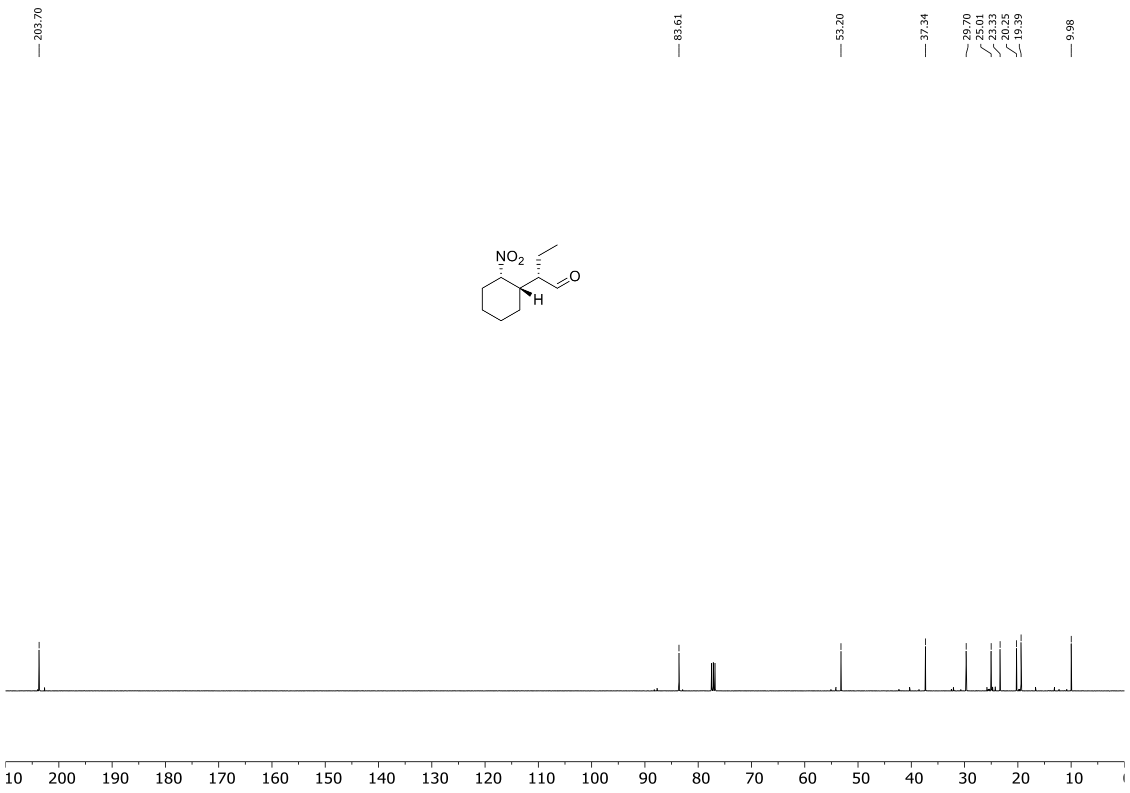

CDCl$_3$ ^{1}H (400 MHz) and ^{13}C (101 MHz) of **P4**

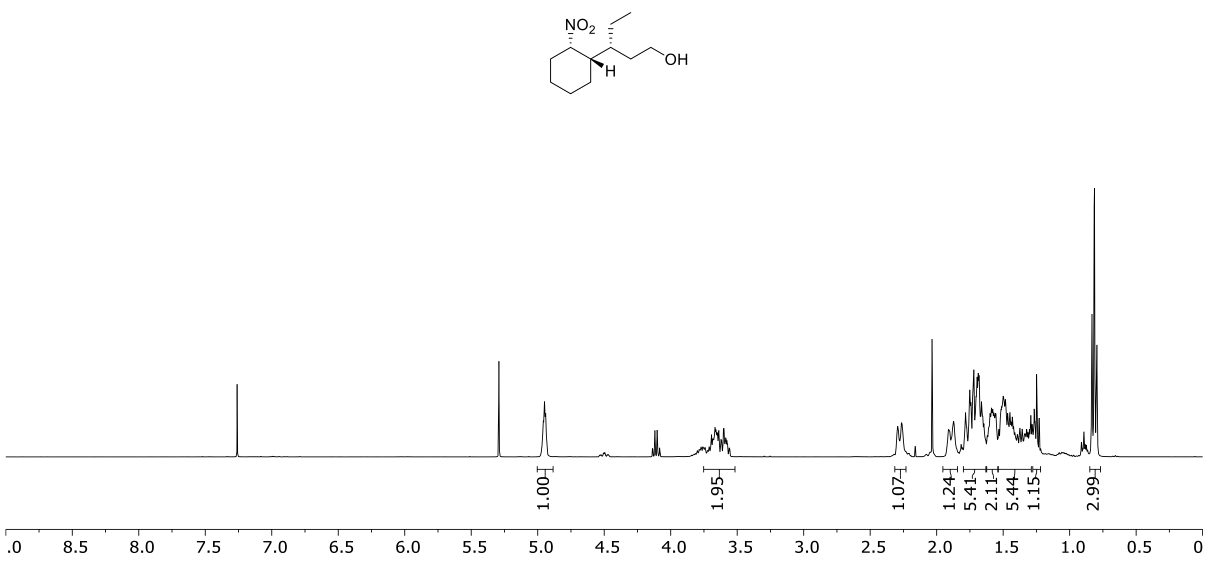

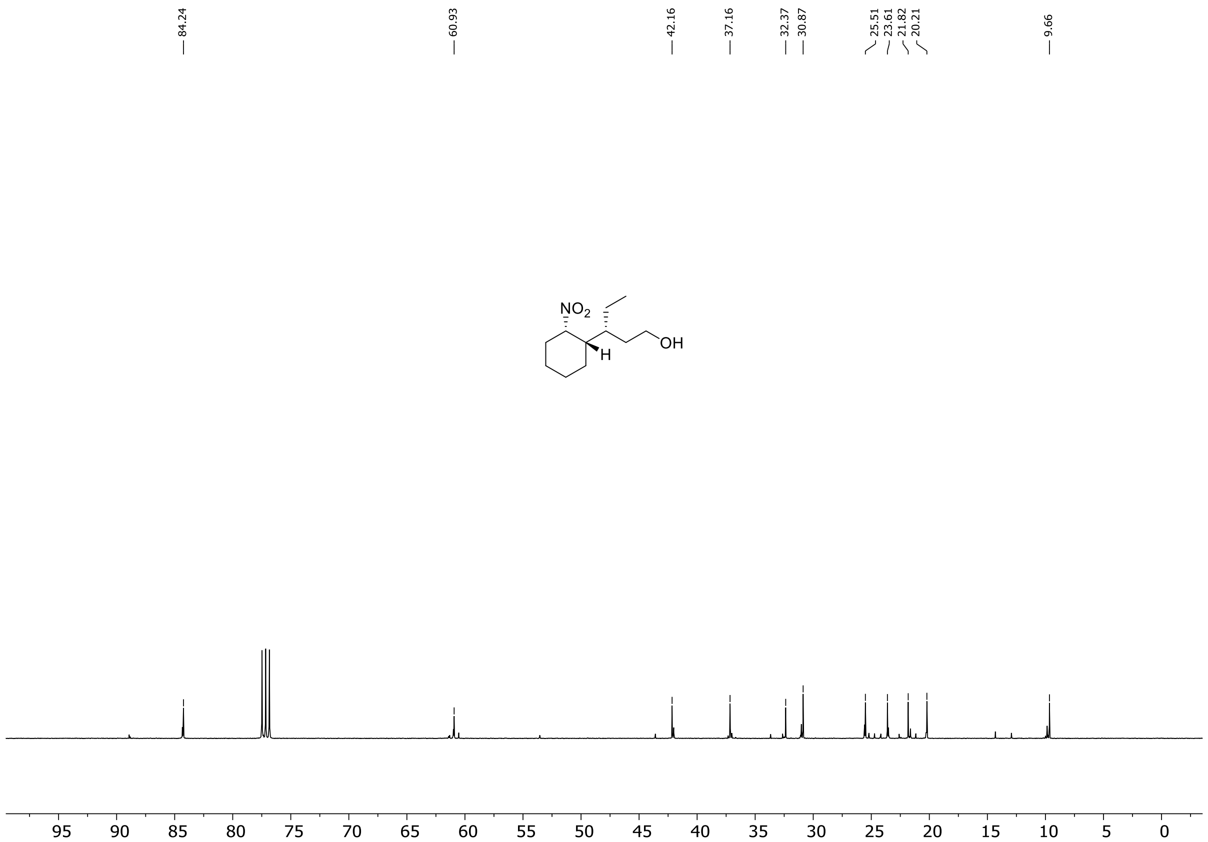

CDCl$_3$ ^{1}H (400 MHz) and ^{13}C (101 MHz) of **P5**

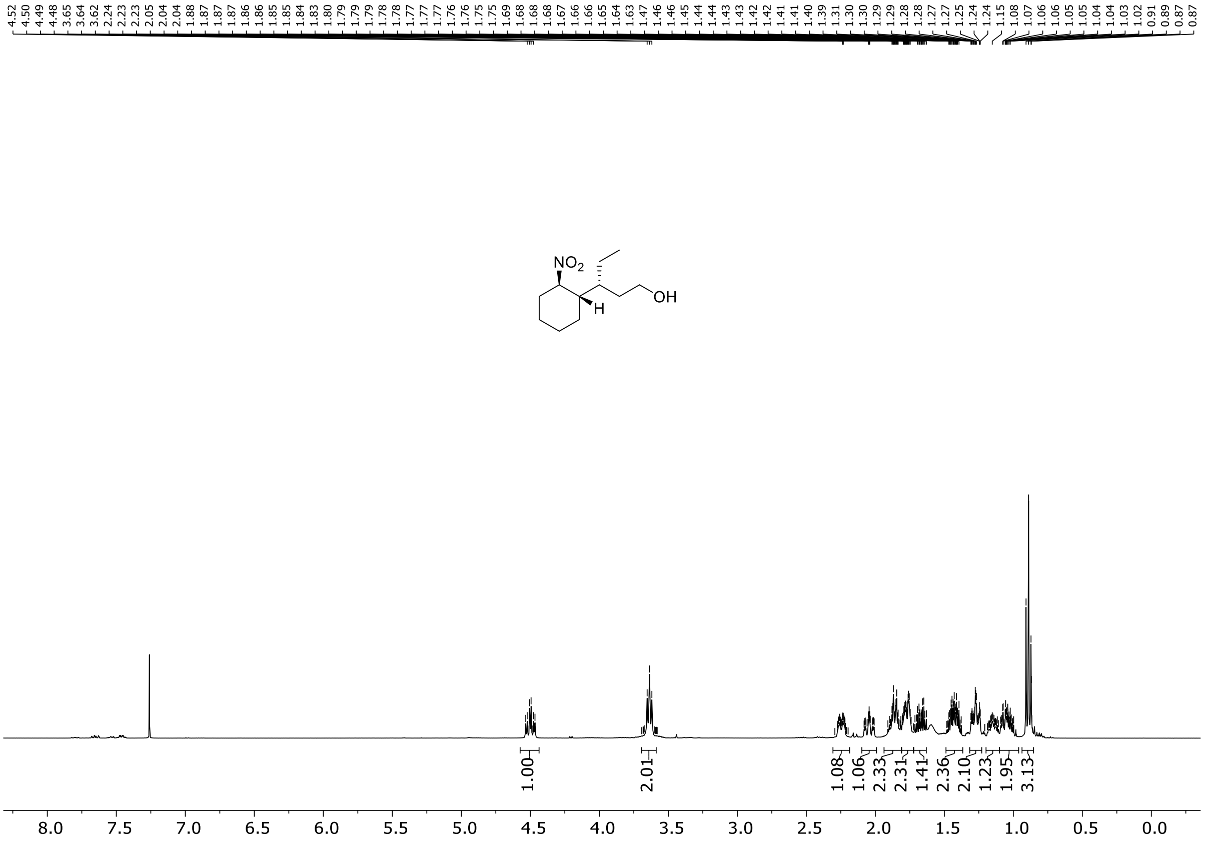

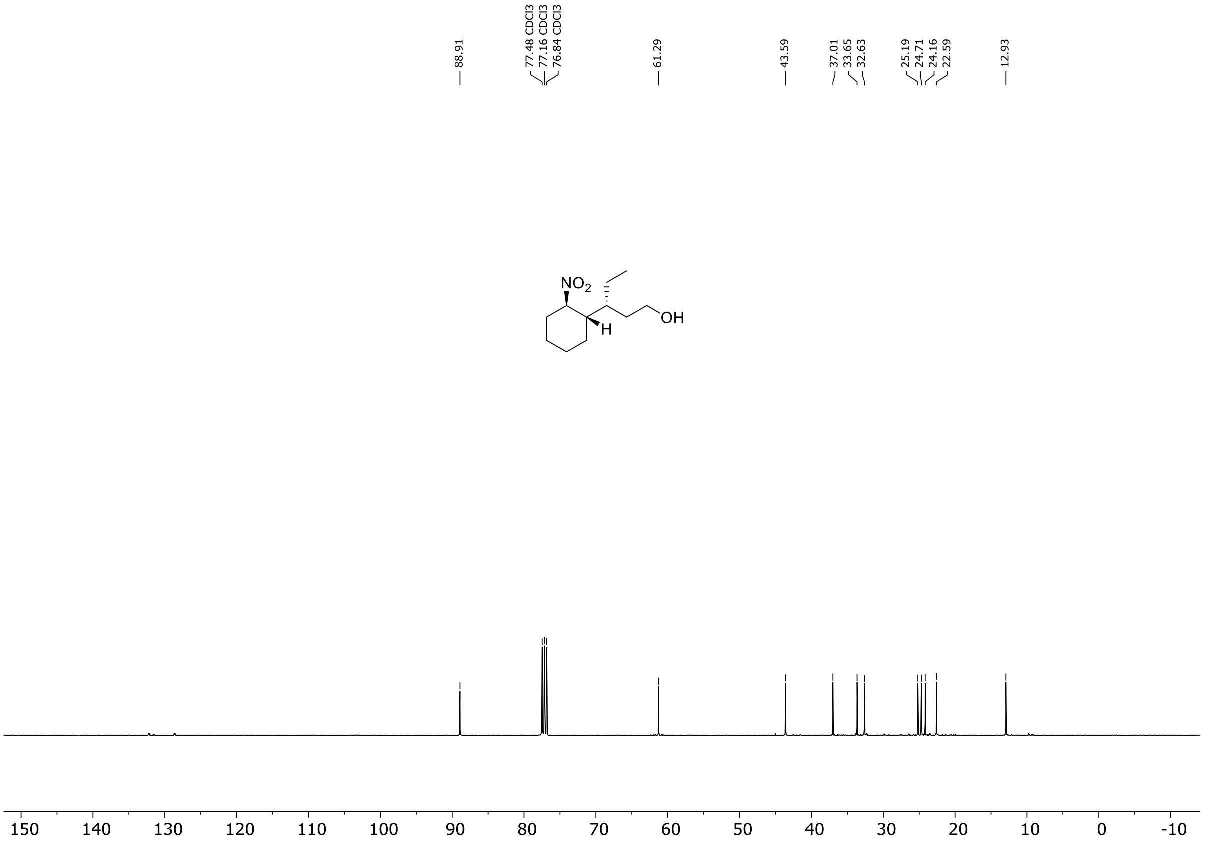

$CDCl_3$ ^{1}H (400 MHz) and ^{13}C (101 MHz) of **P6**

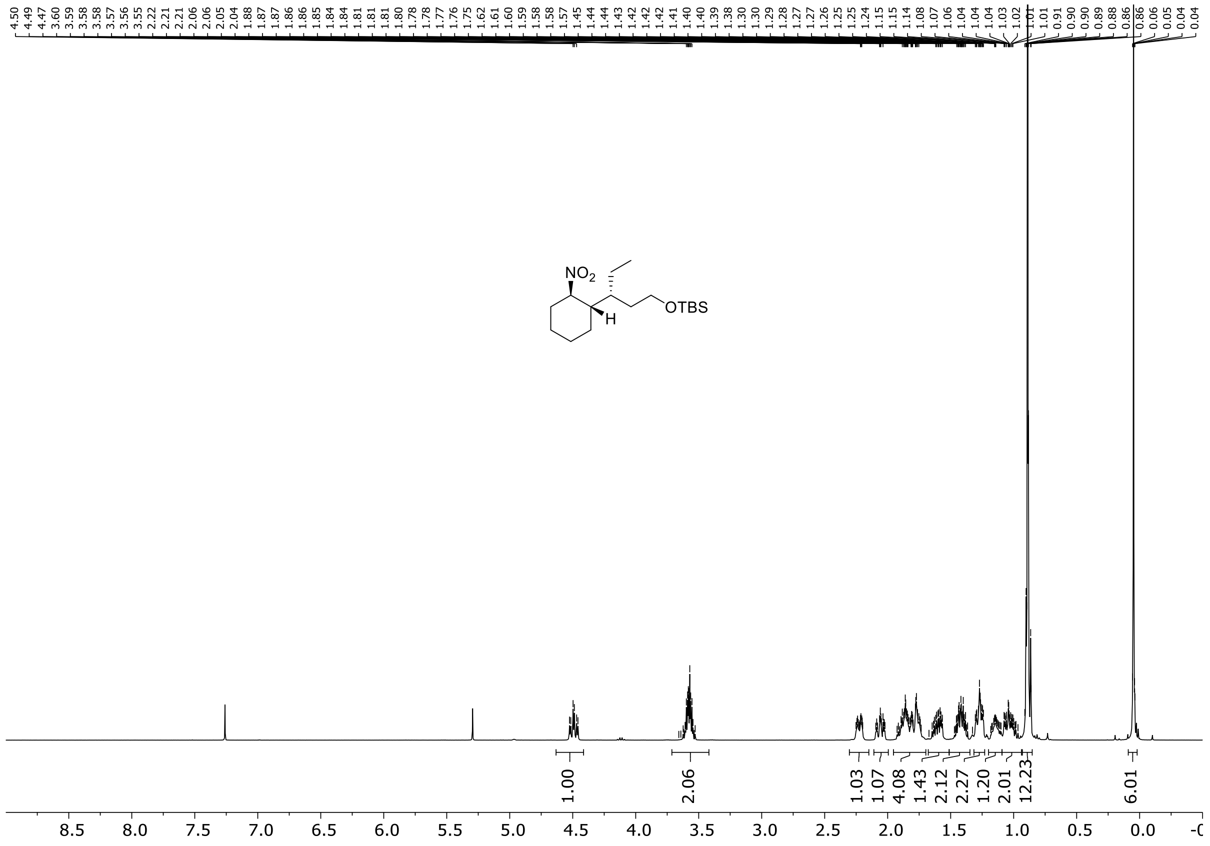

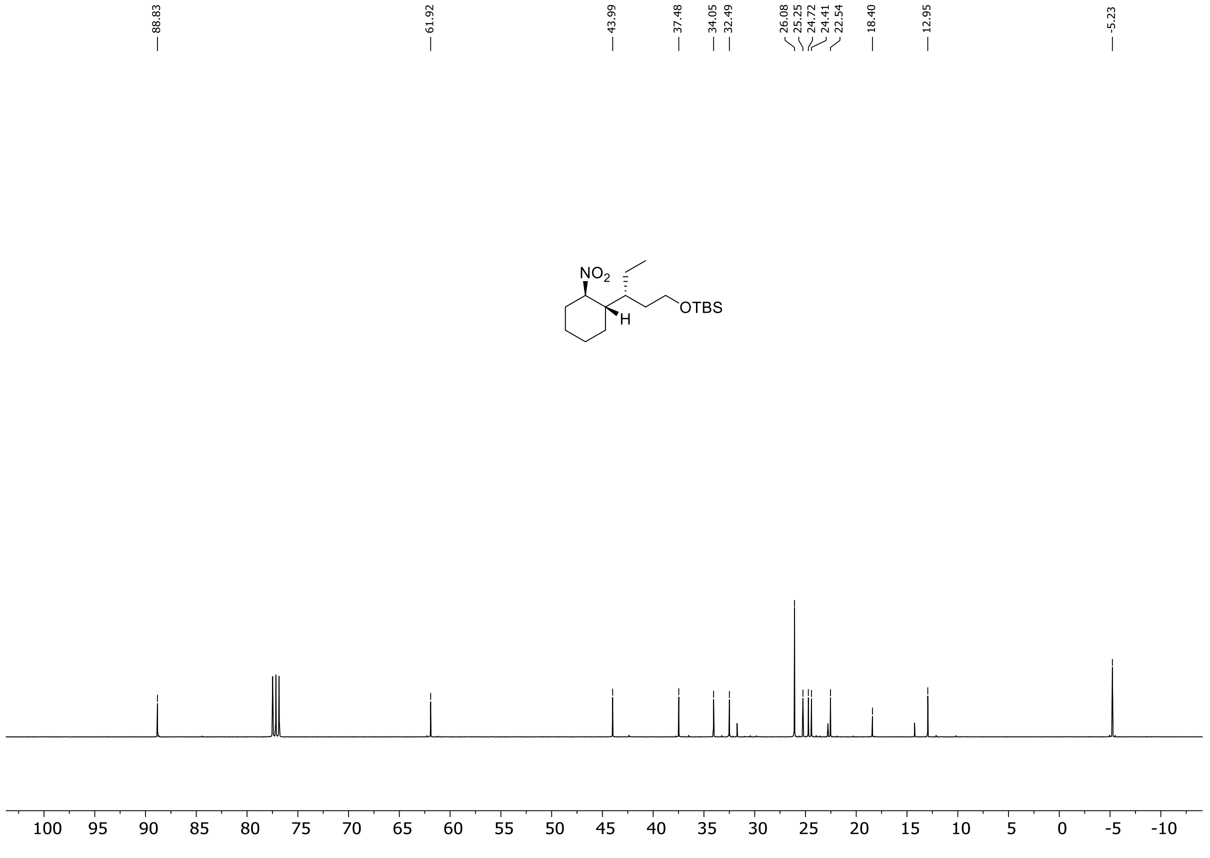

$CDCl_3$ ^1H (400 MHz) and ^{13}C (101 MHz) of **P7**

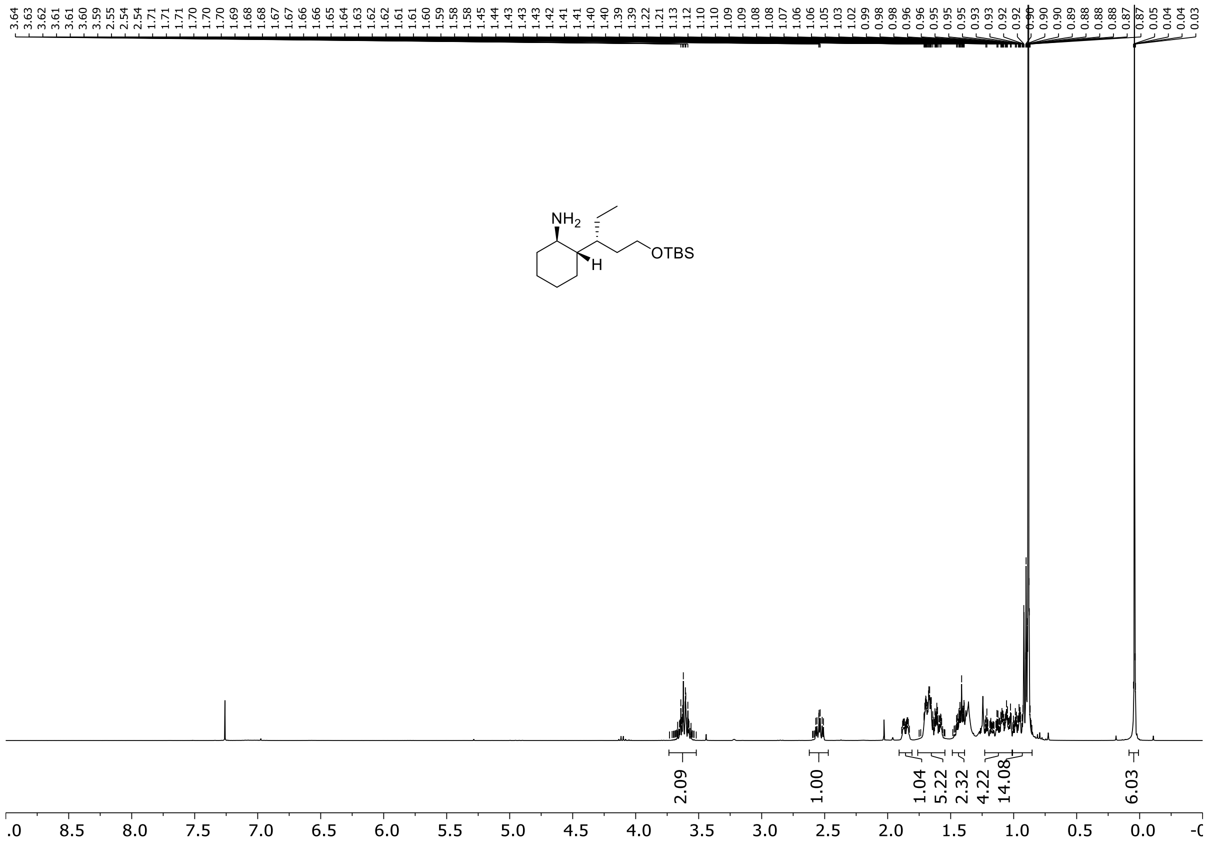

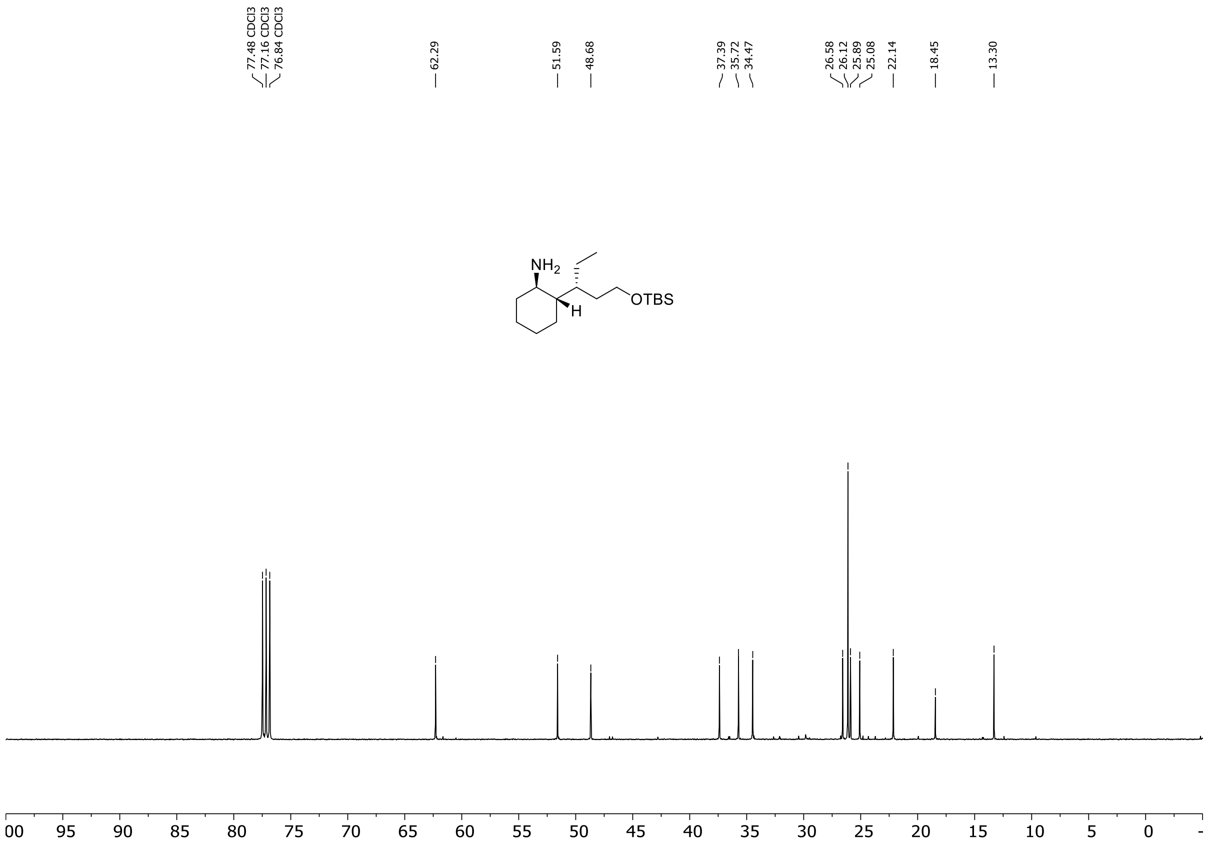

CDCl$_3$ ^{1}H (400 MHz) and ^{13}C (101 MHz) of **P9**

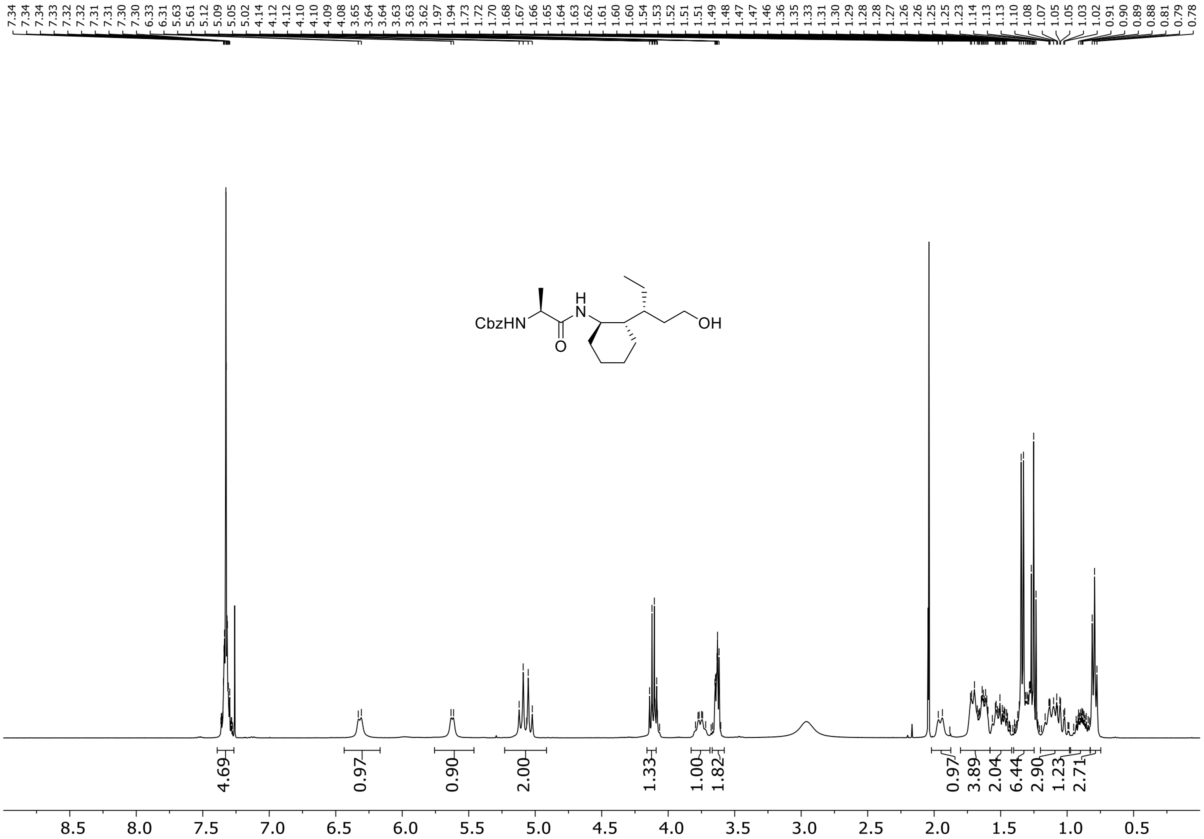

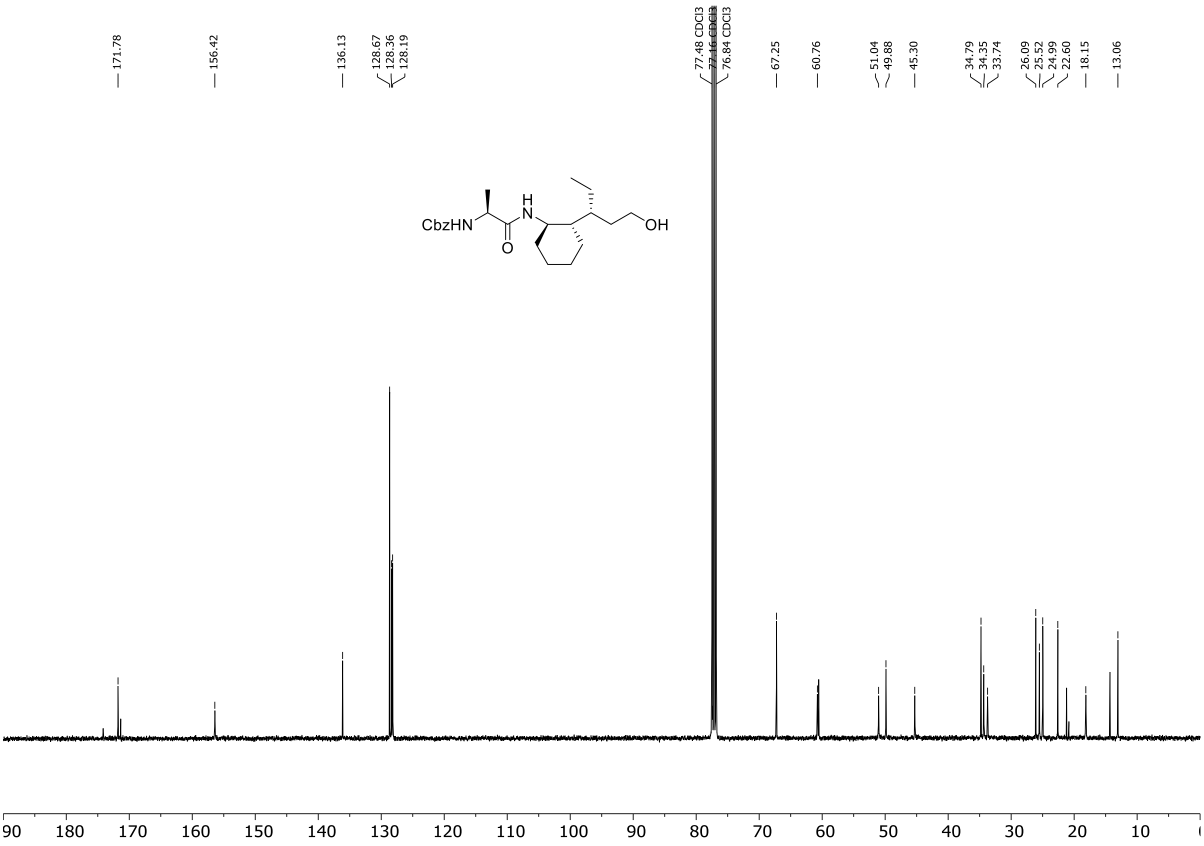

CDCl$_3$ ^{1}H (400 MHz) and ^{13}C (101 MHz) of **P11**

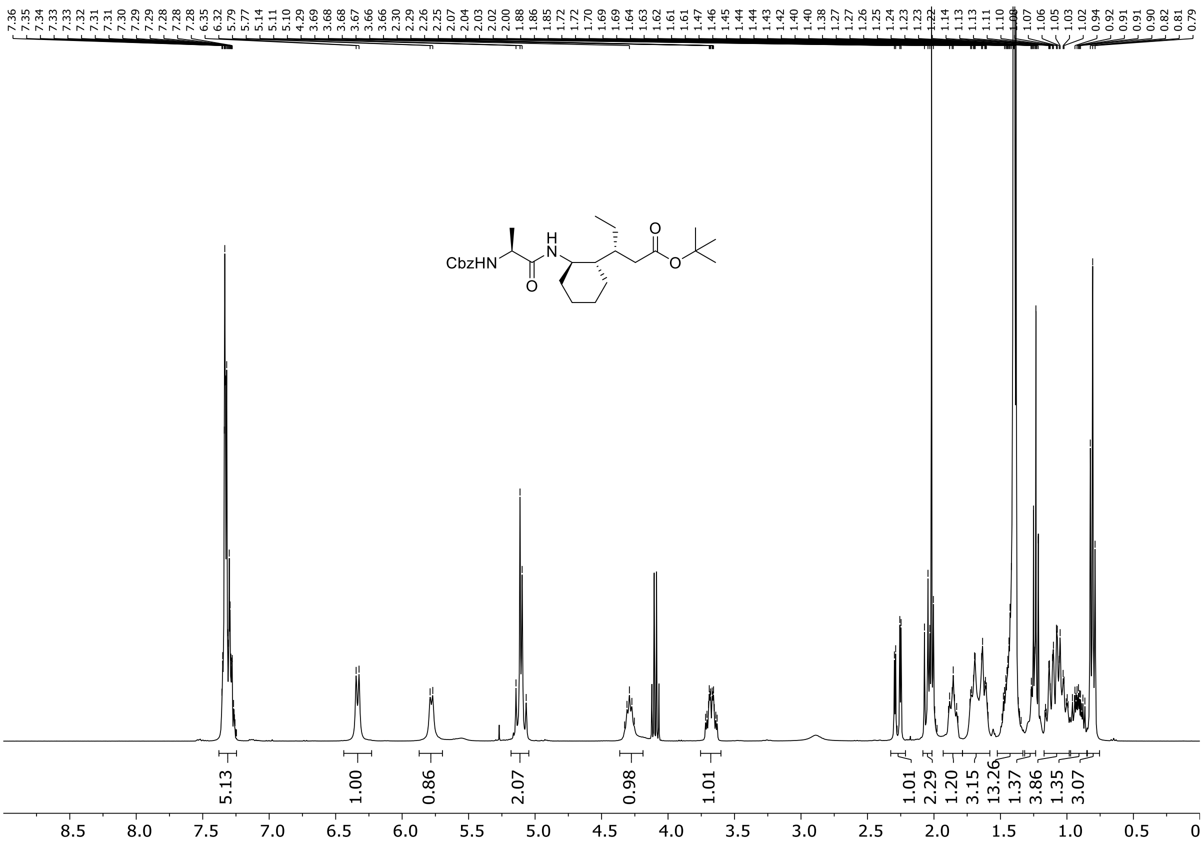

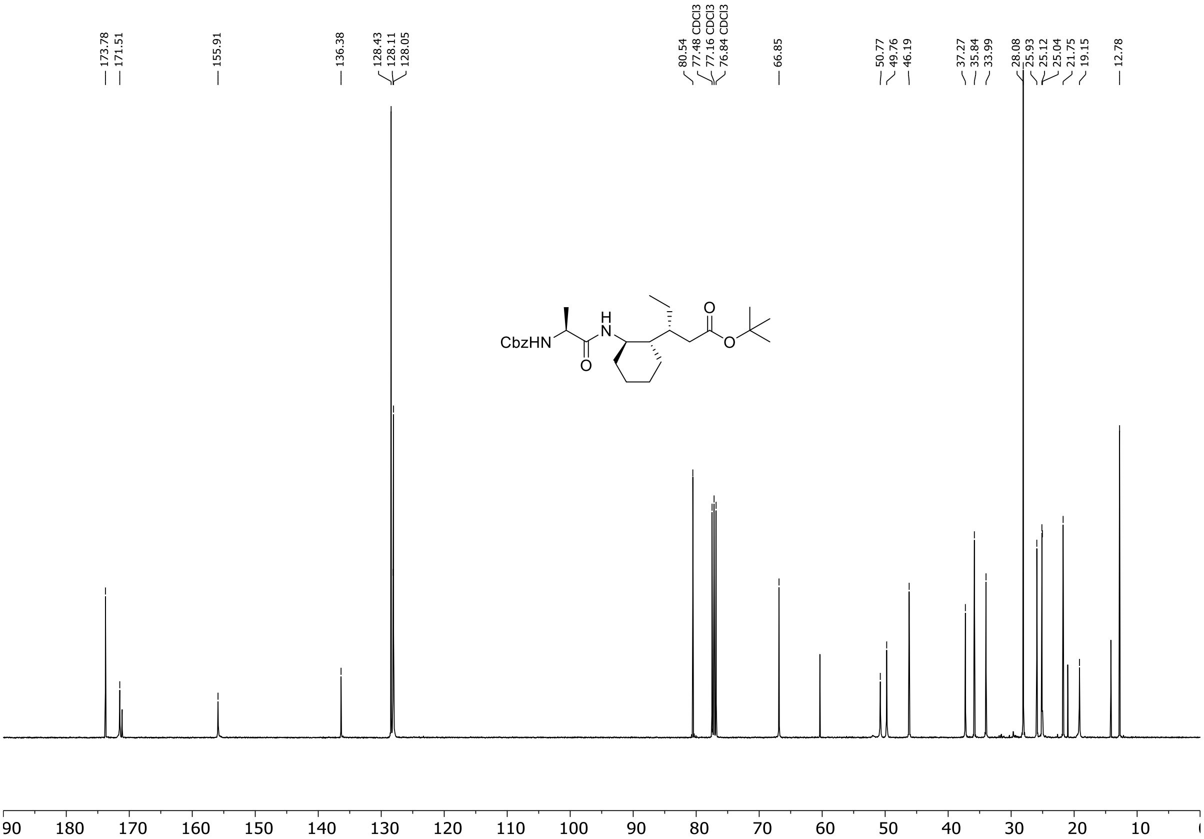

CDCl$_3$ ^{1}H (400 MHz) and ^{13}C (101 MHz) of **P12**

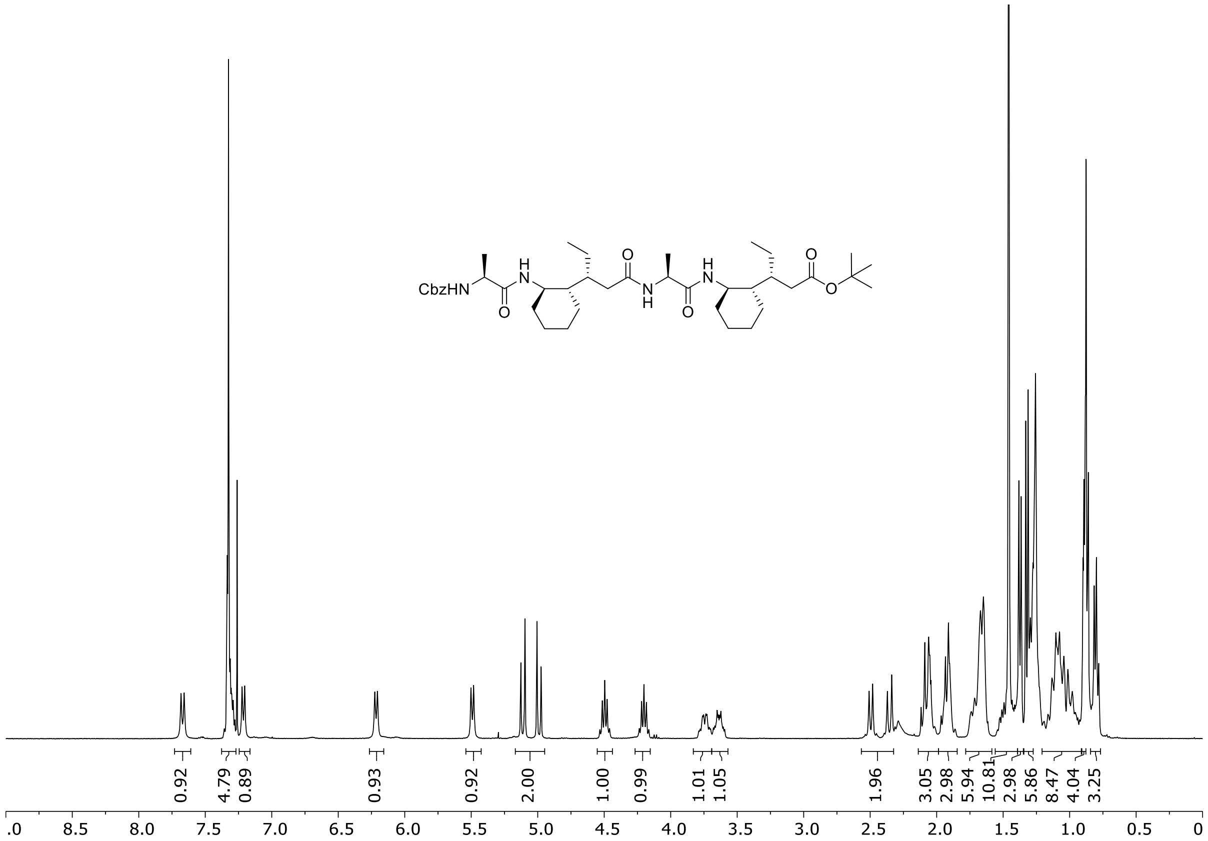

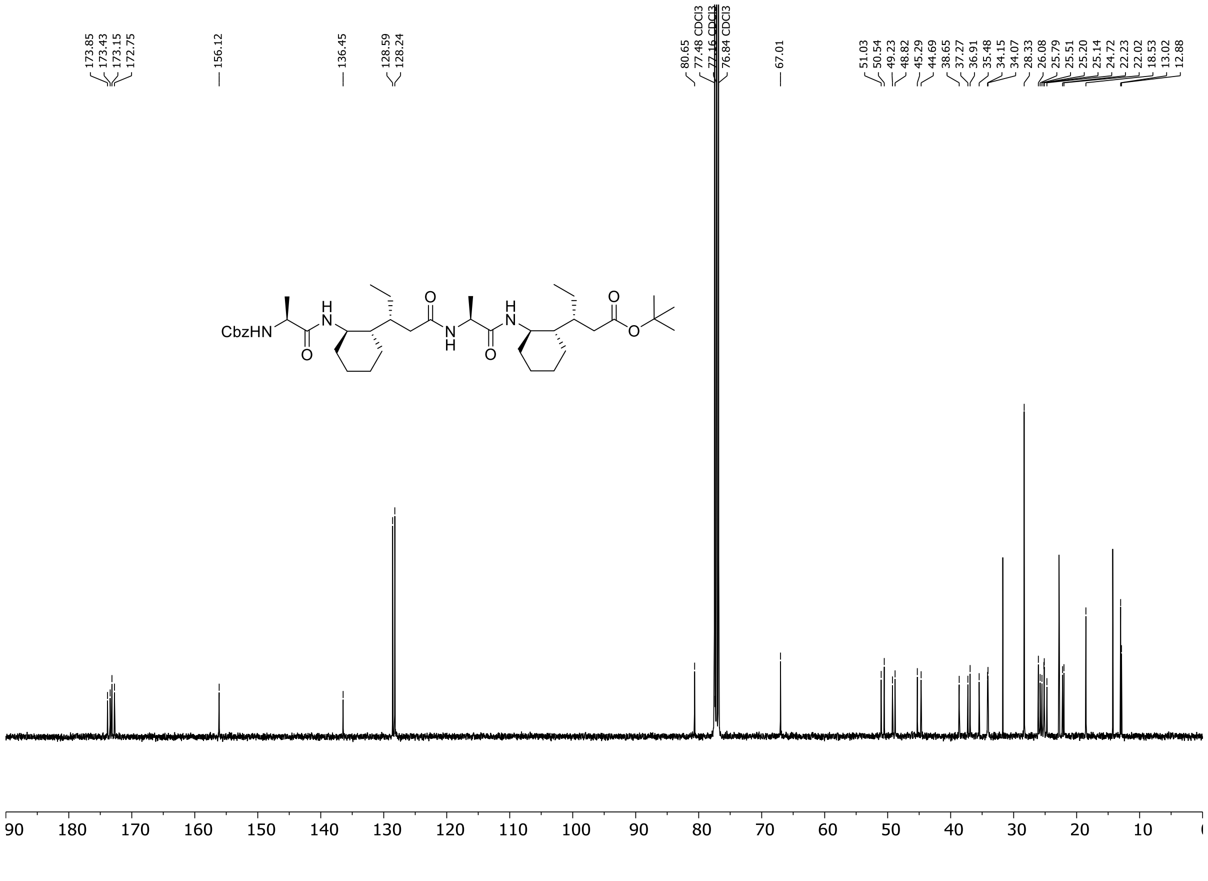

CDCl$_3$ ^{1}H (400 MHz) and ^{13}C (101 MHz) of **P13**

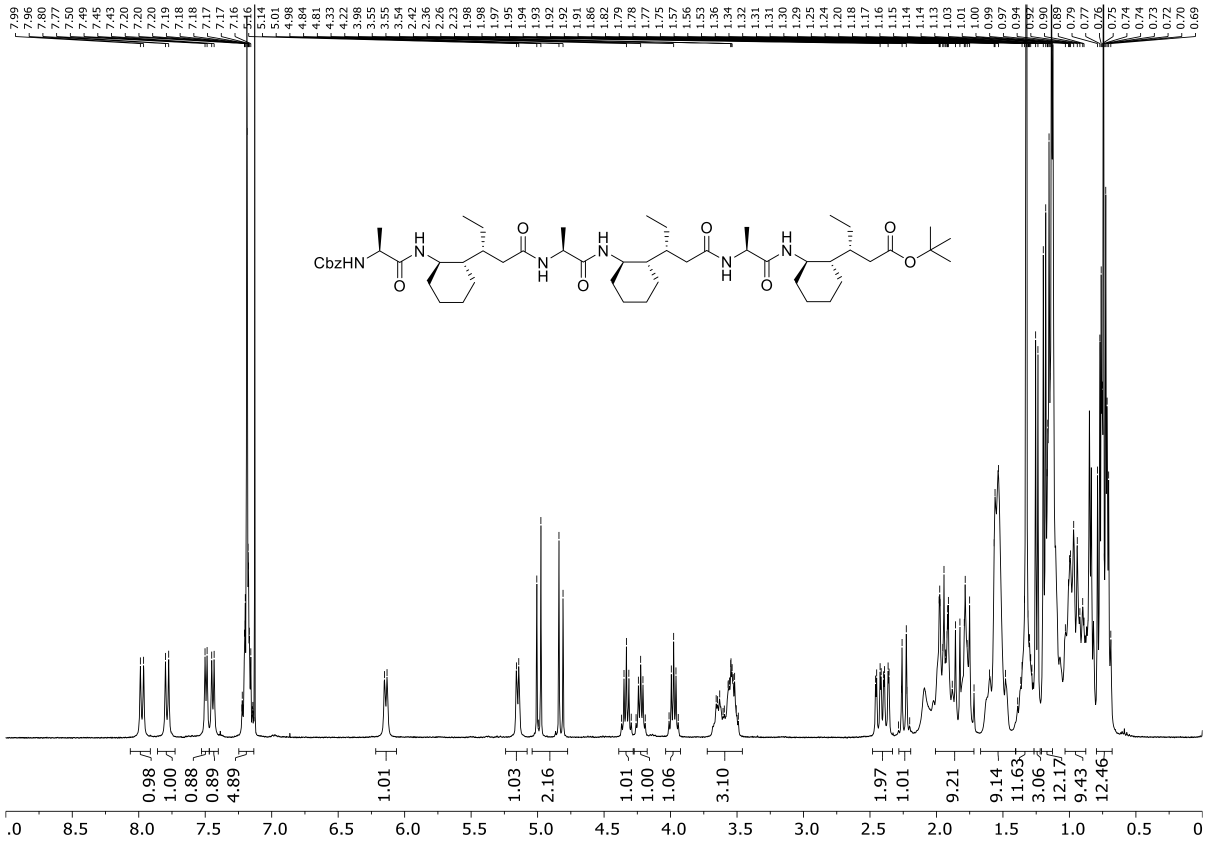

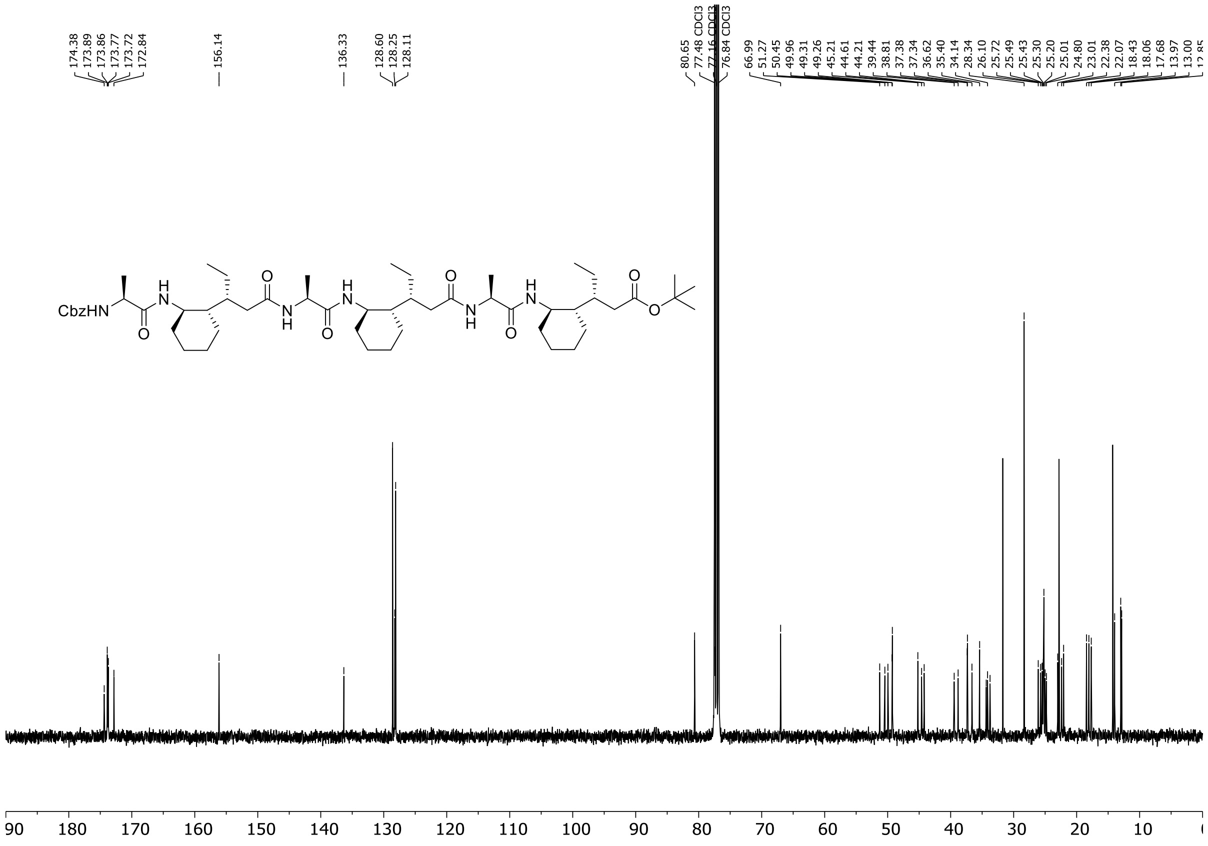

CDCl$_3$ ^{1}H (400 MHz) and ^{13}C (101 MHz) of Octameric Foldamer **2**

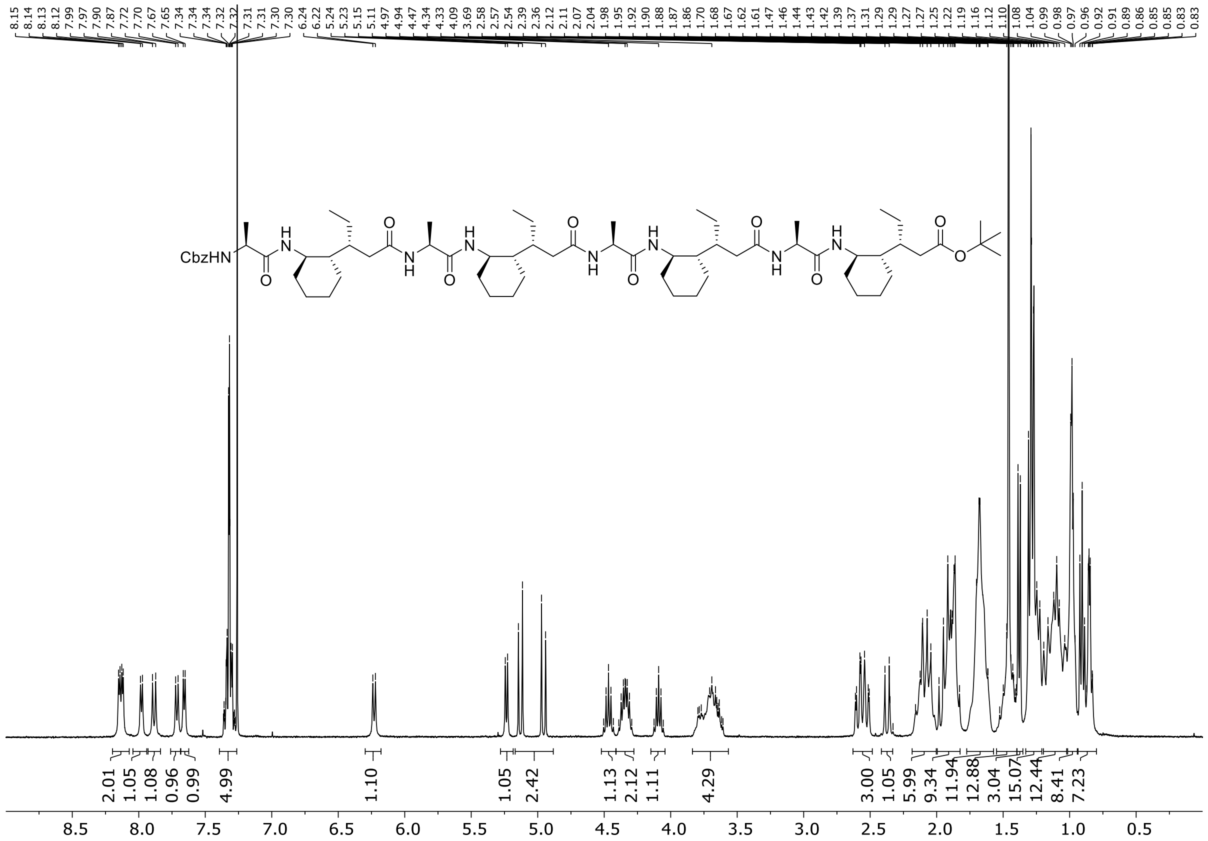

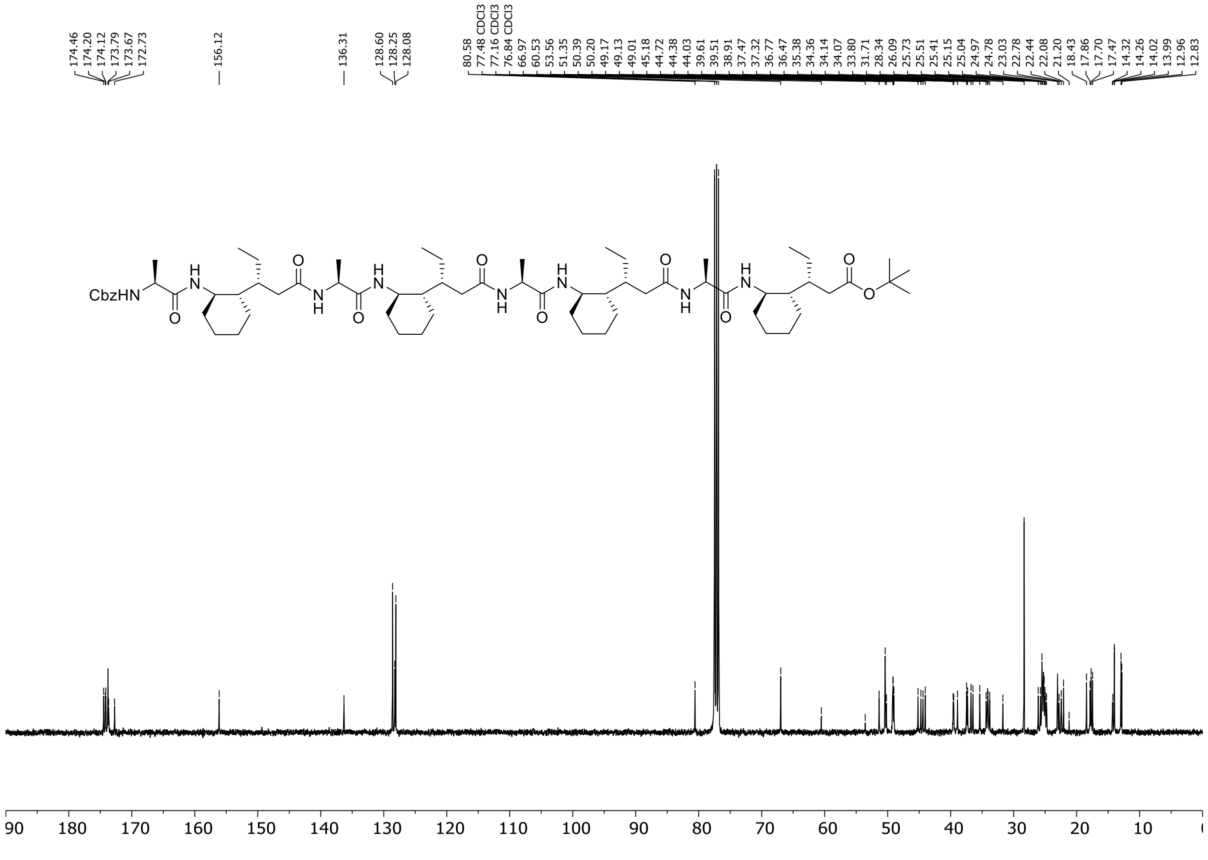

CDCl$_3$ ^{1}H (400 MHz) and ^{13}C (101 MHz) of **P17**

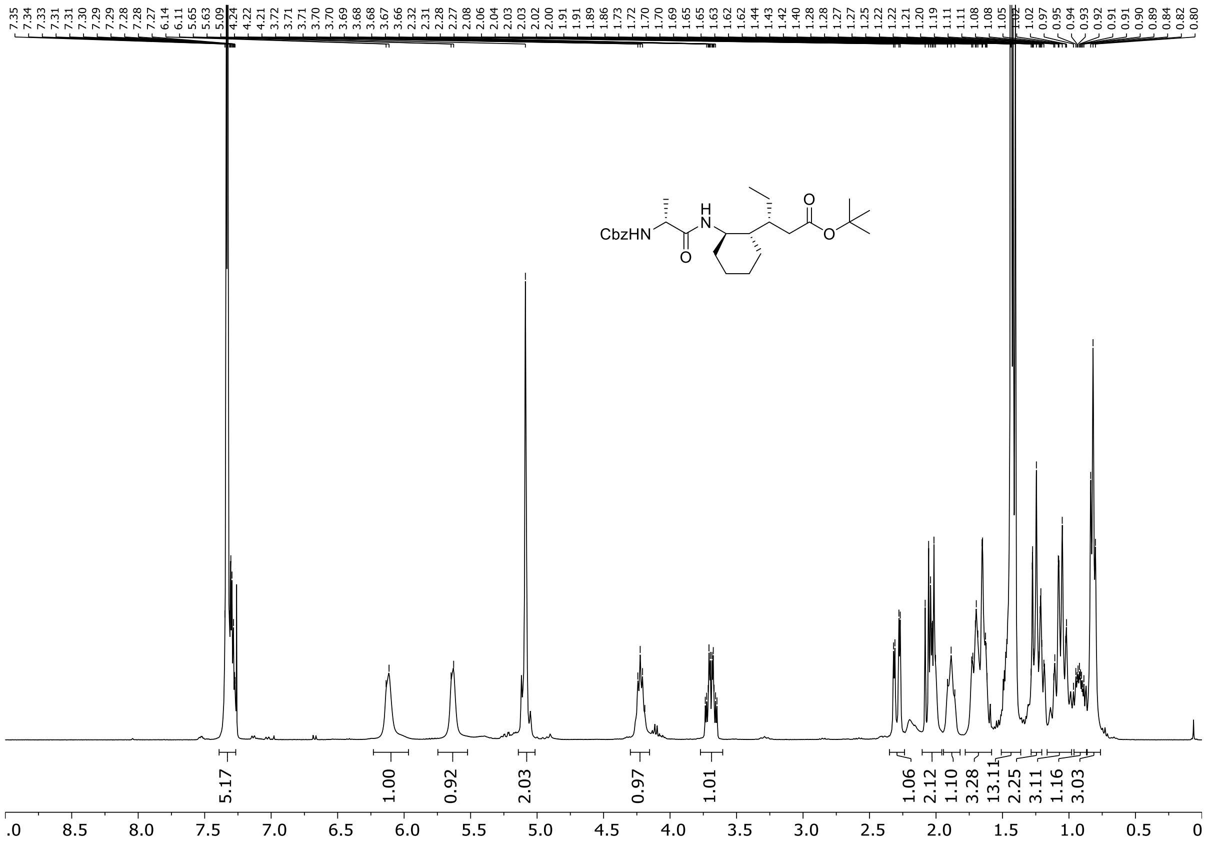

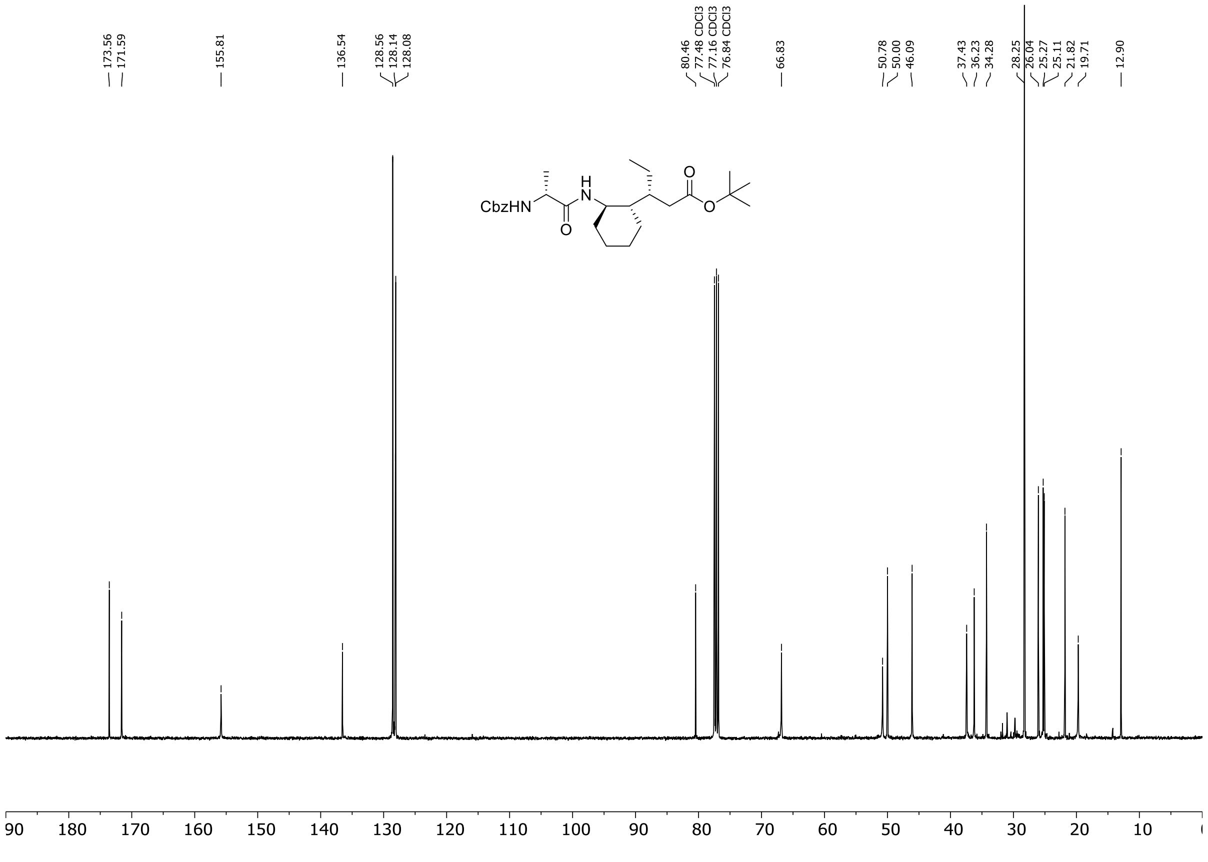

CDCl$_3$ ^{1}H (400 MHz) and ^{13}C (101 MHz) of **P18**

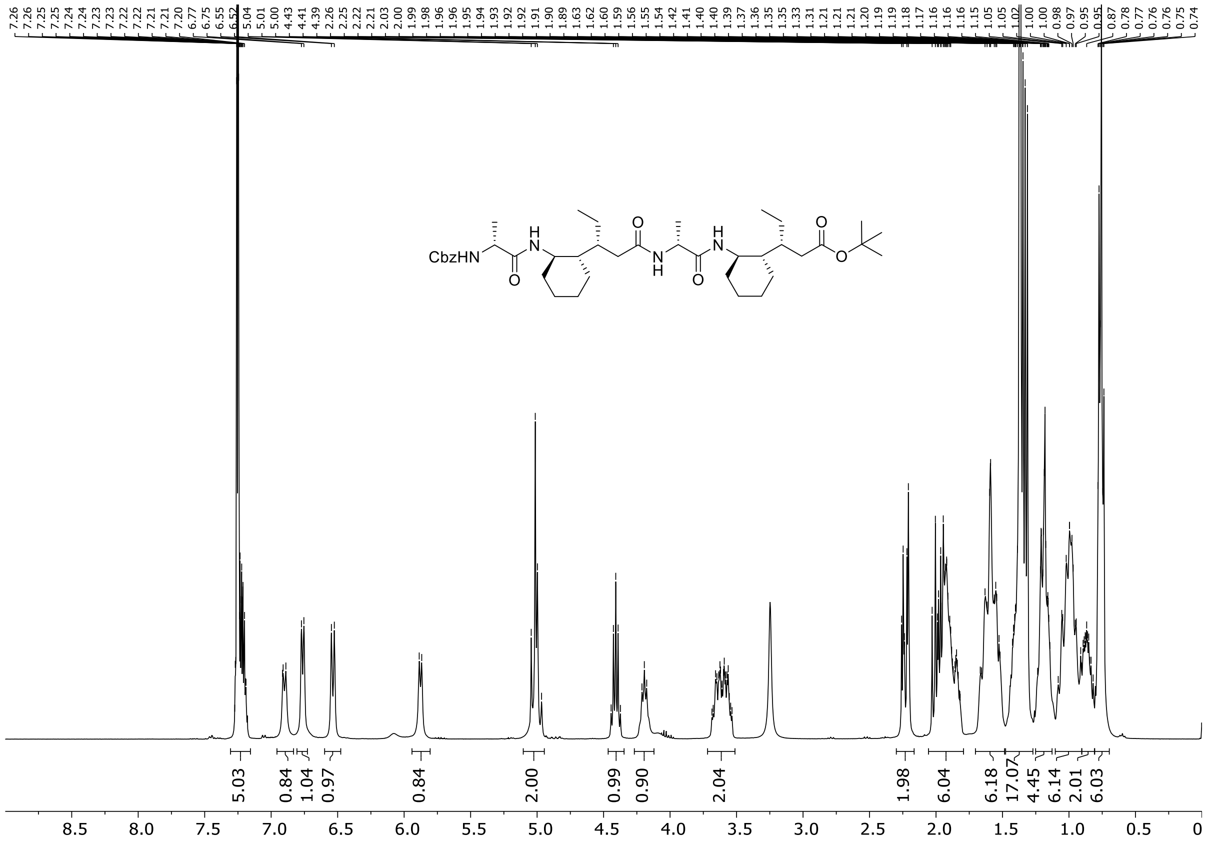

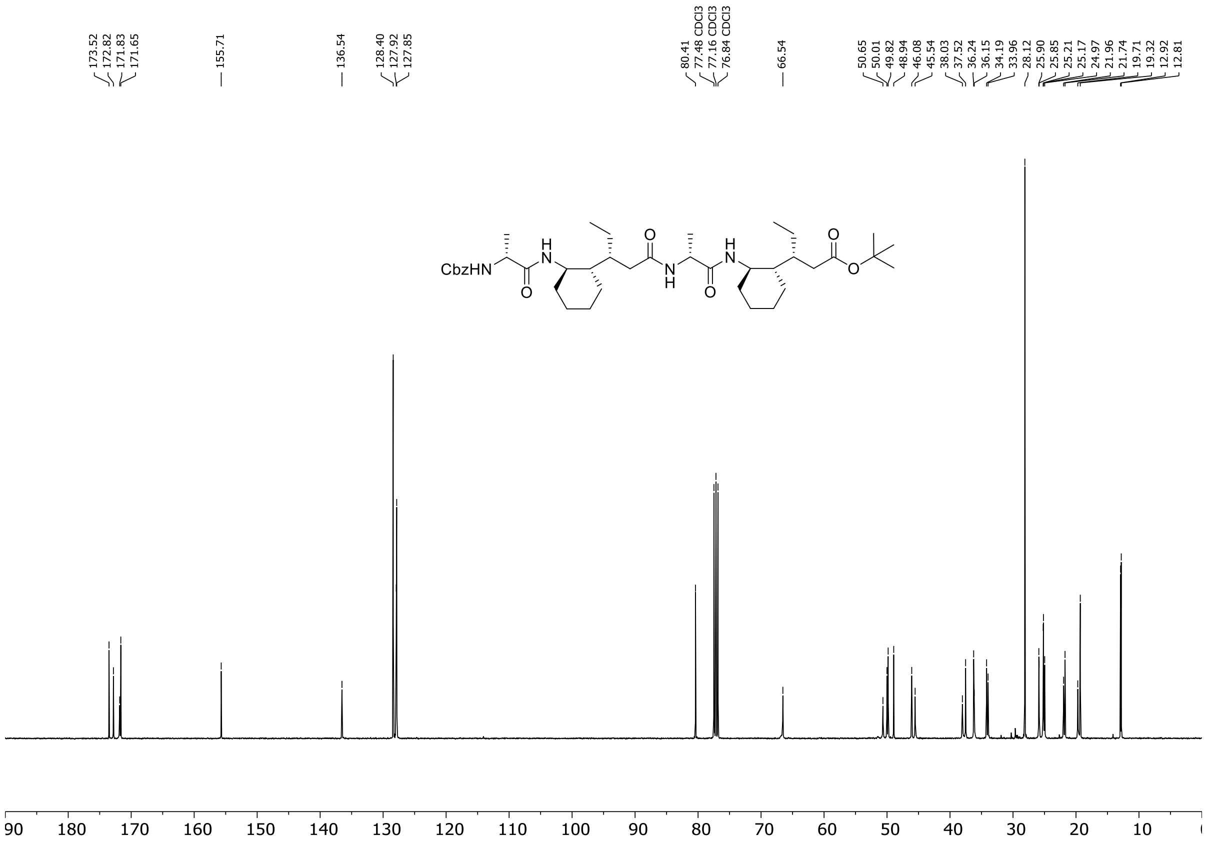

CDCl$_3$ ^{1}H (400 MHz) and ^{13}C (101 MHz) of **P19**

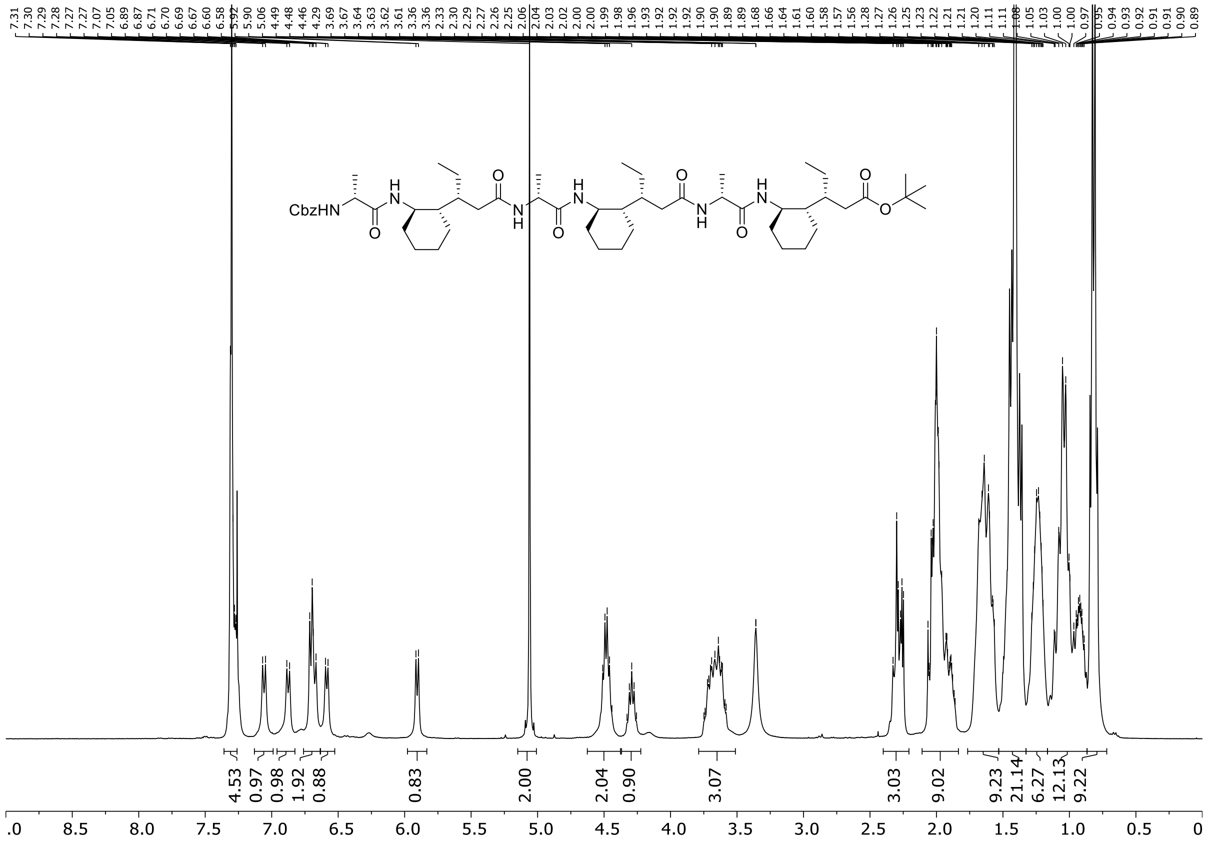

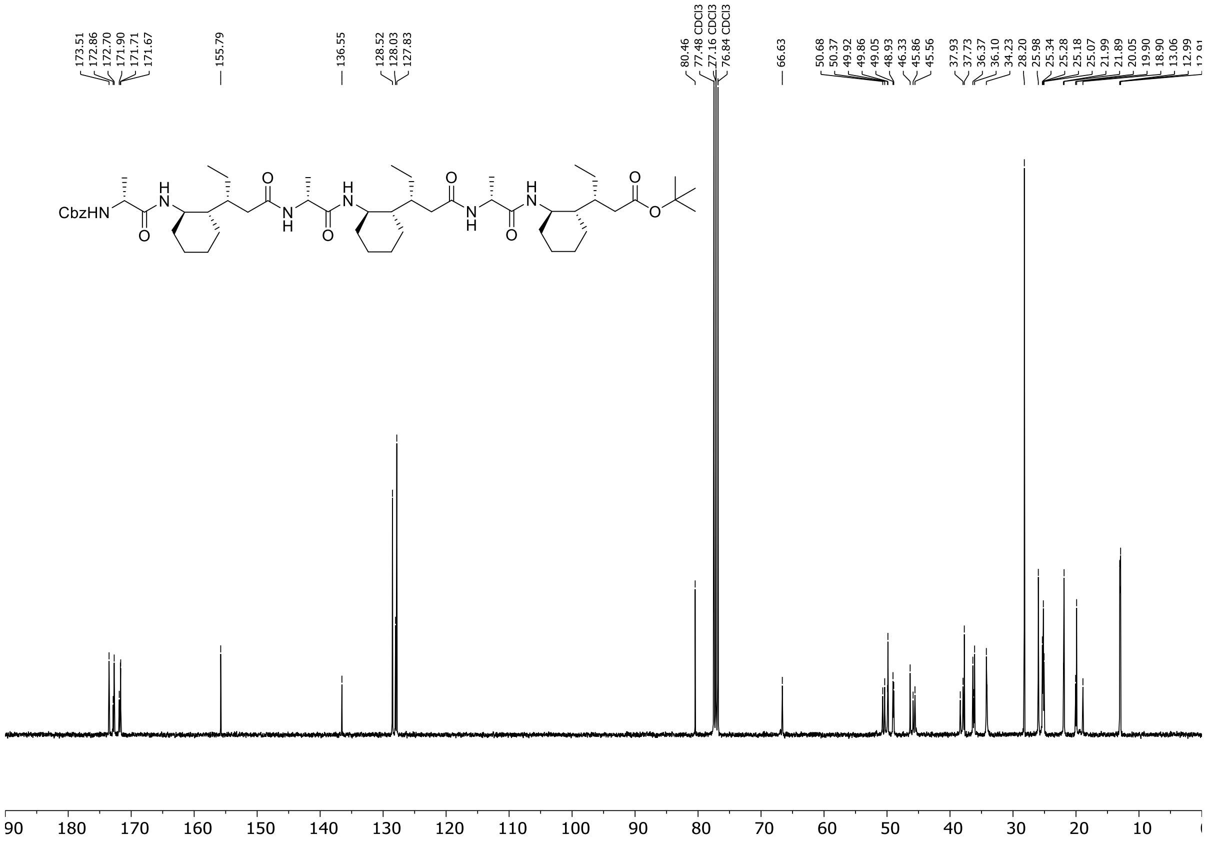

CDCl$_3$ ^{1}H (400 MHz) and ^{13}C (101 MHz) of Oligomer **3**

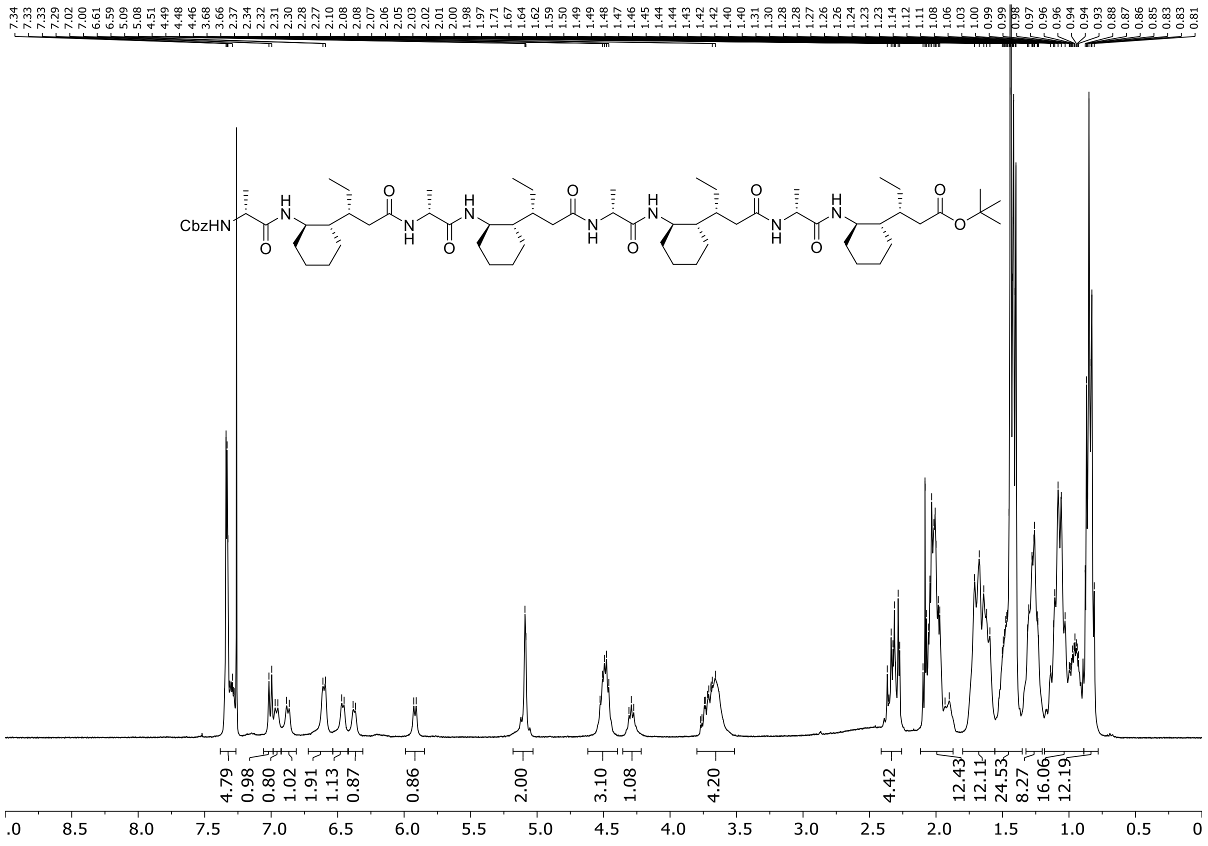

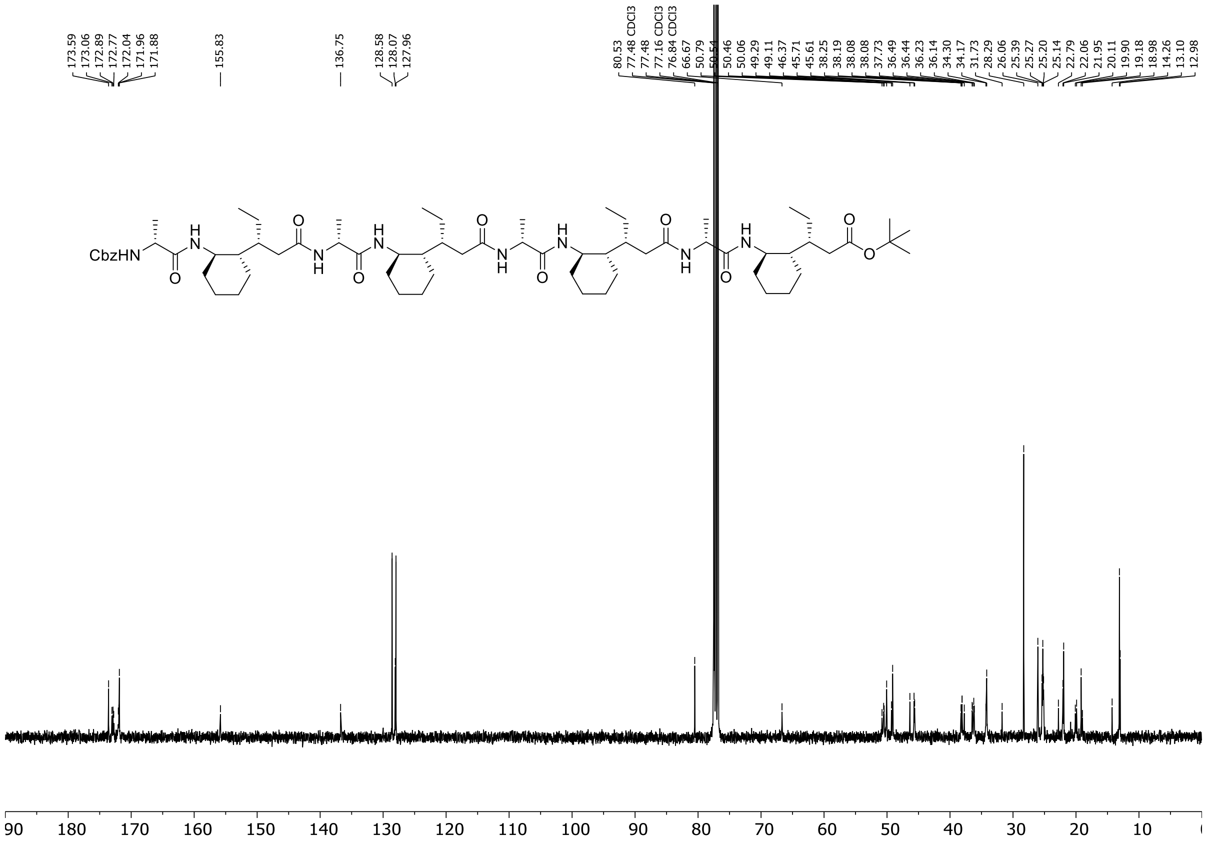

CDCl$_3$ ^{1}H (400 MHz) and ^{13}C (101 MHz) of **P23**

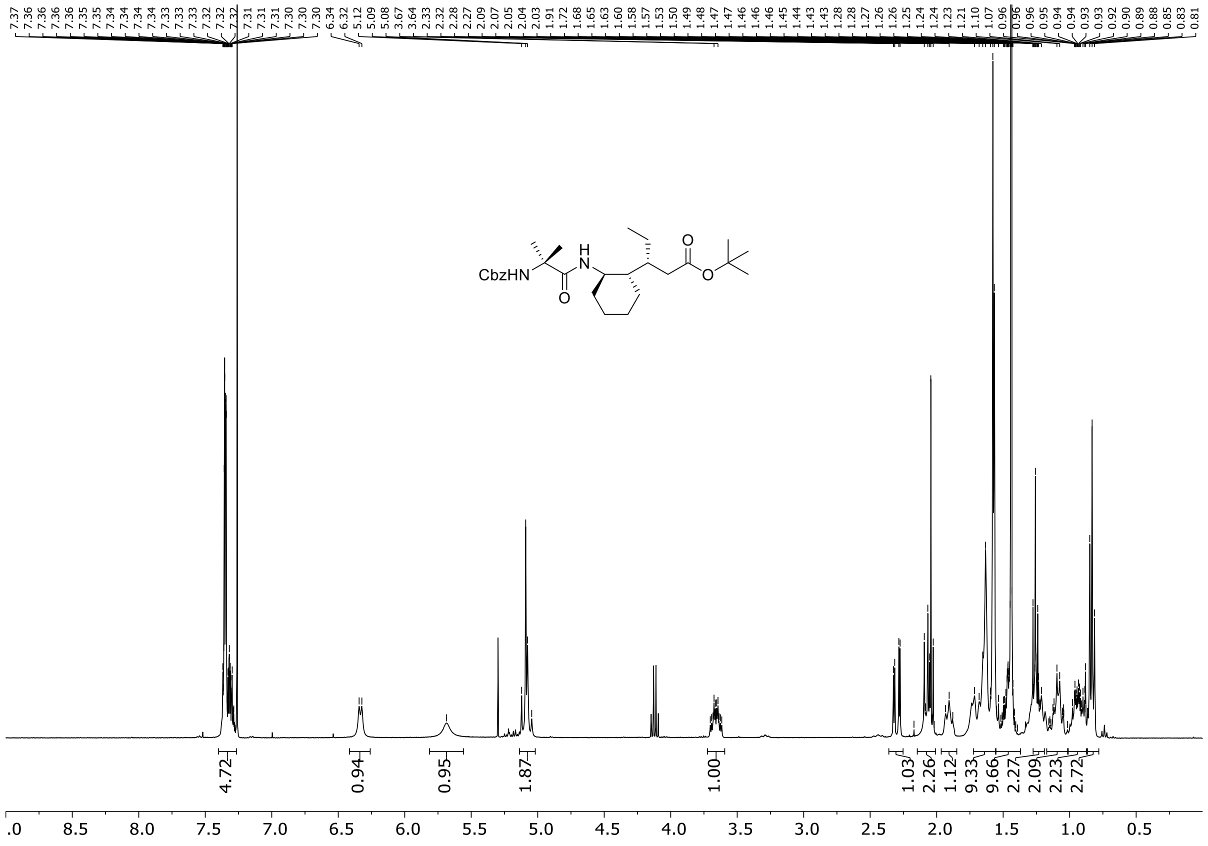

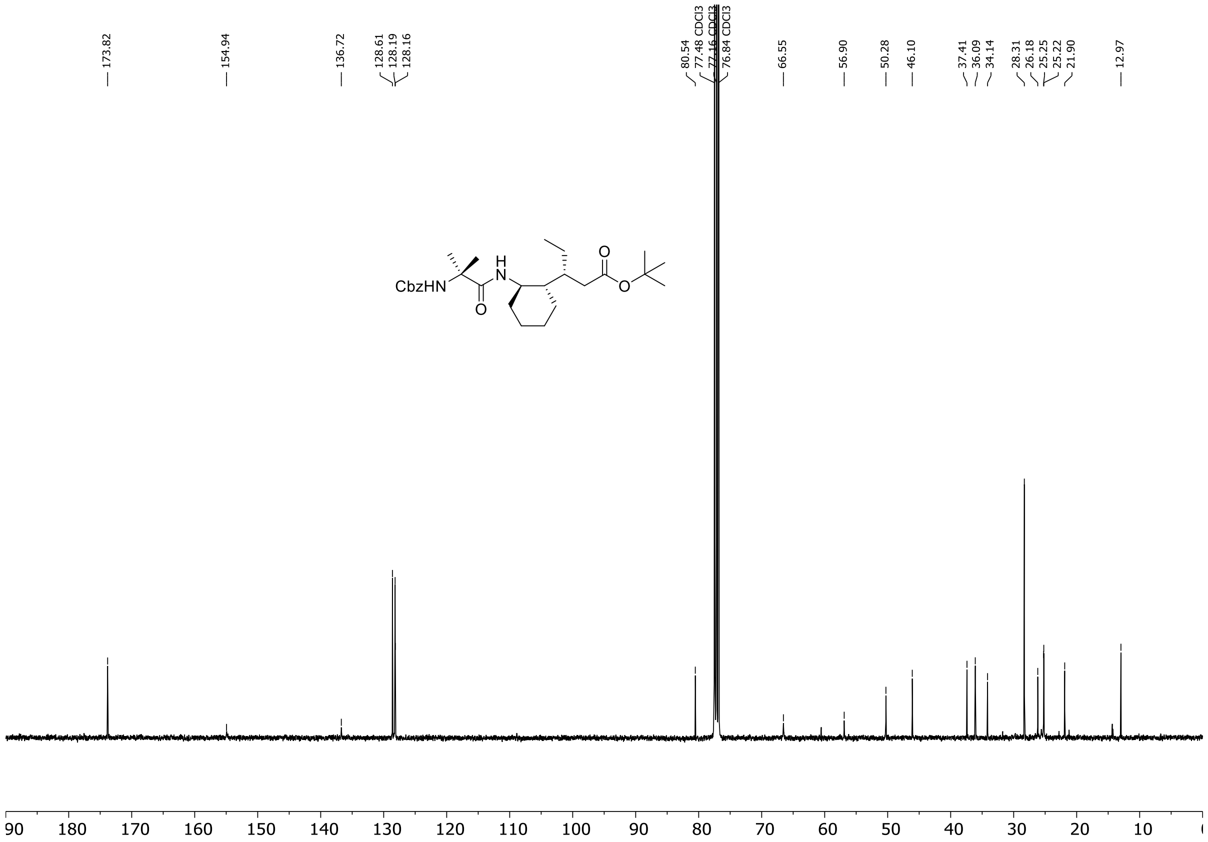

CDCl$_3$ ^{1}H (400 MHz) and ^{13}C (101 MHz) of **P24**

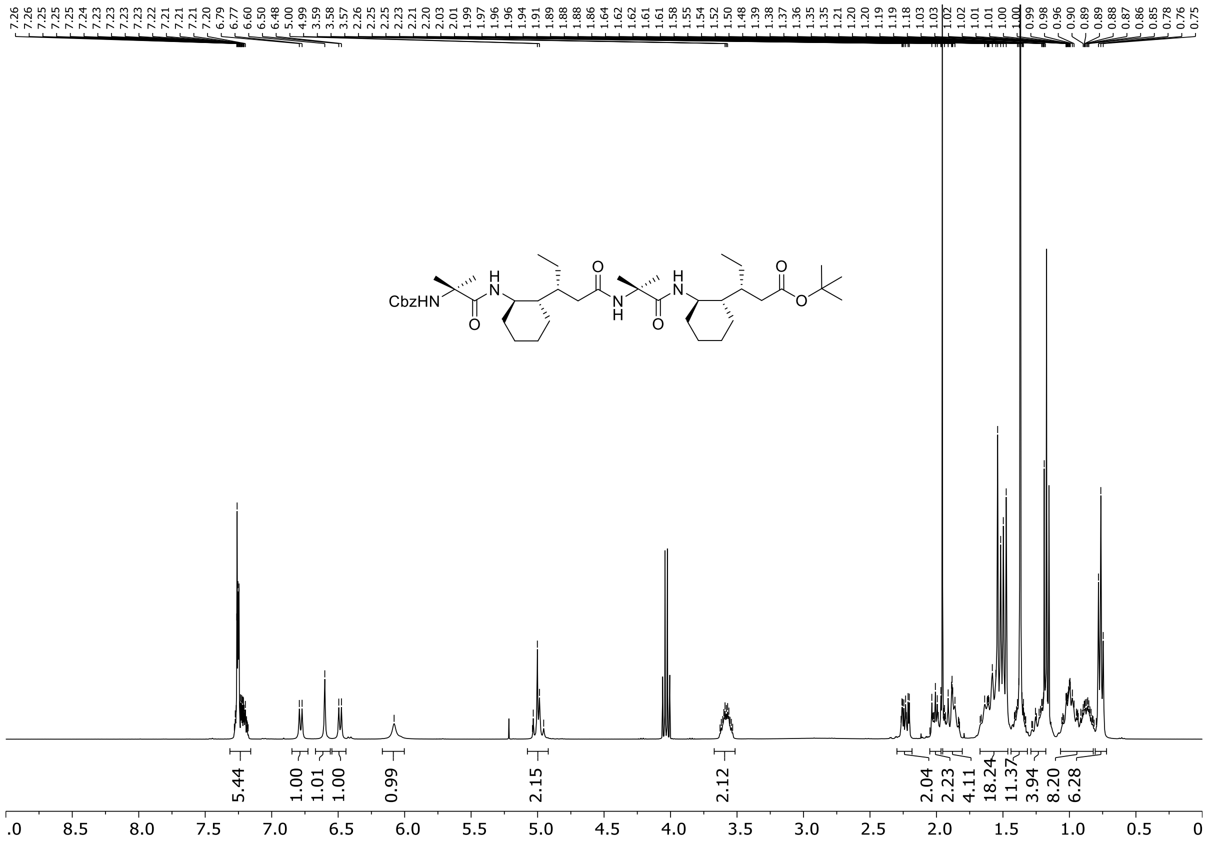

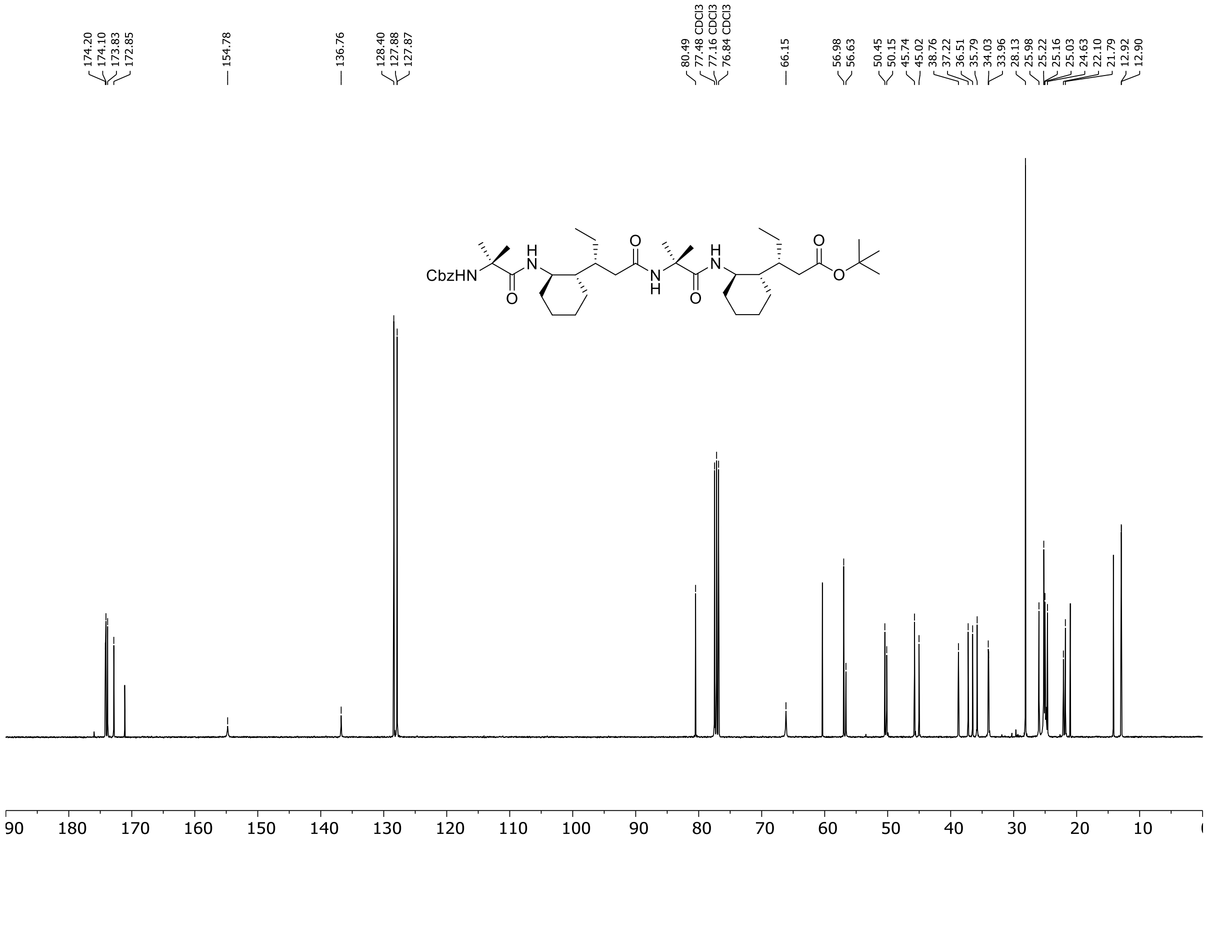

CDCl$_3$ ^{1}H (400 MHz) and ^{13}C (101 MHz) of Oligomer **4**

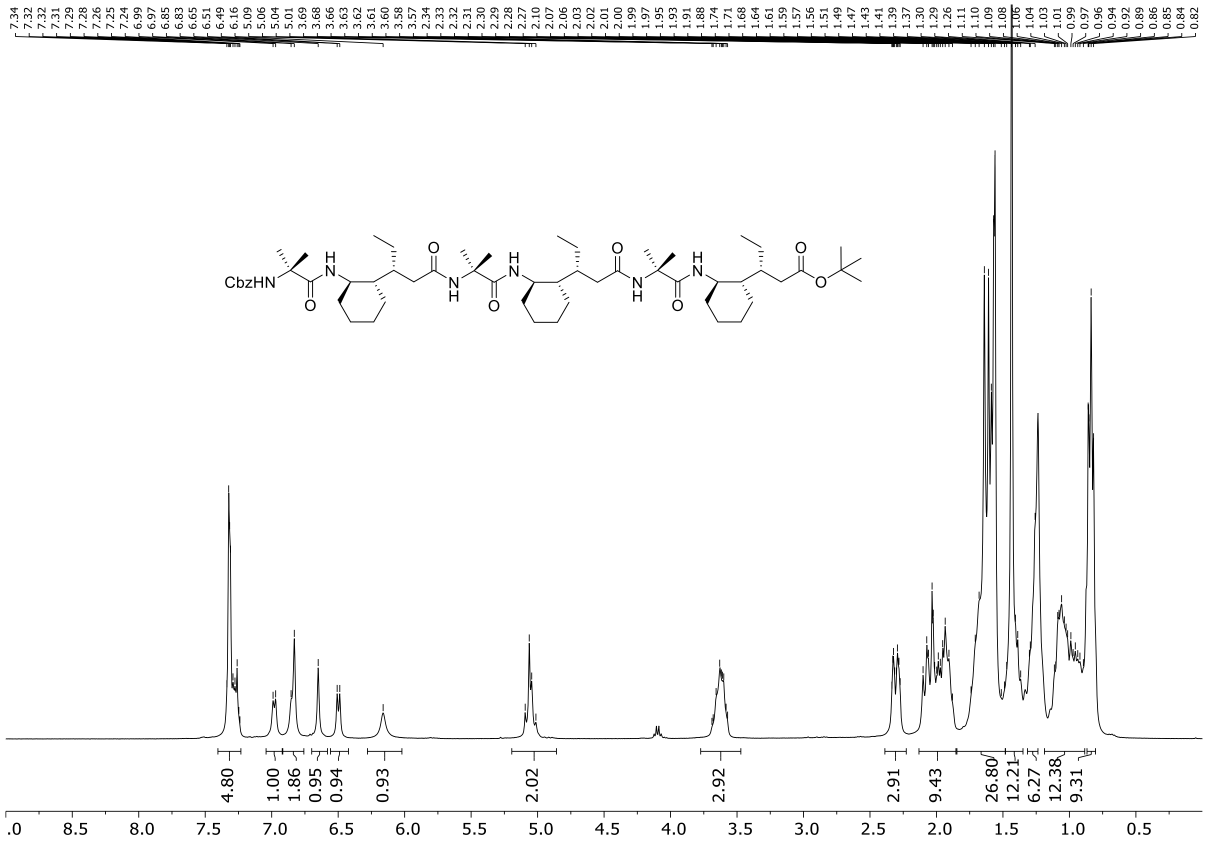

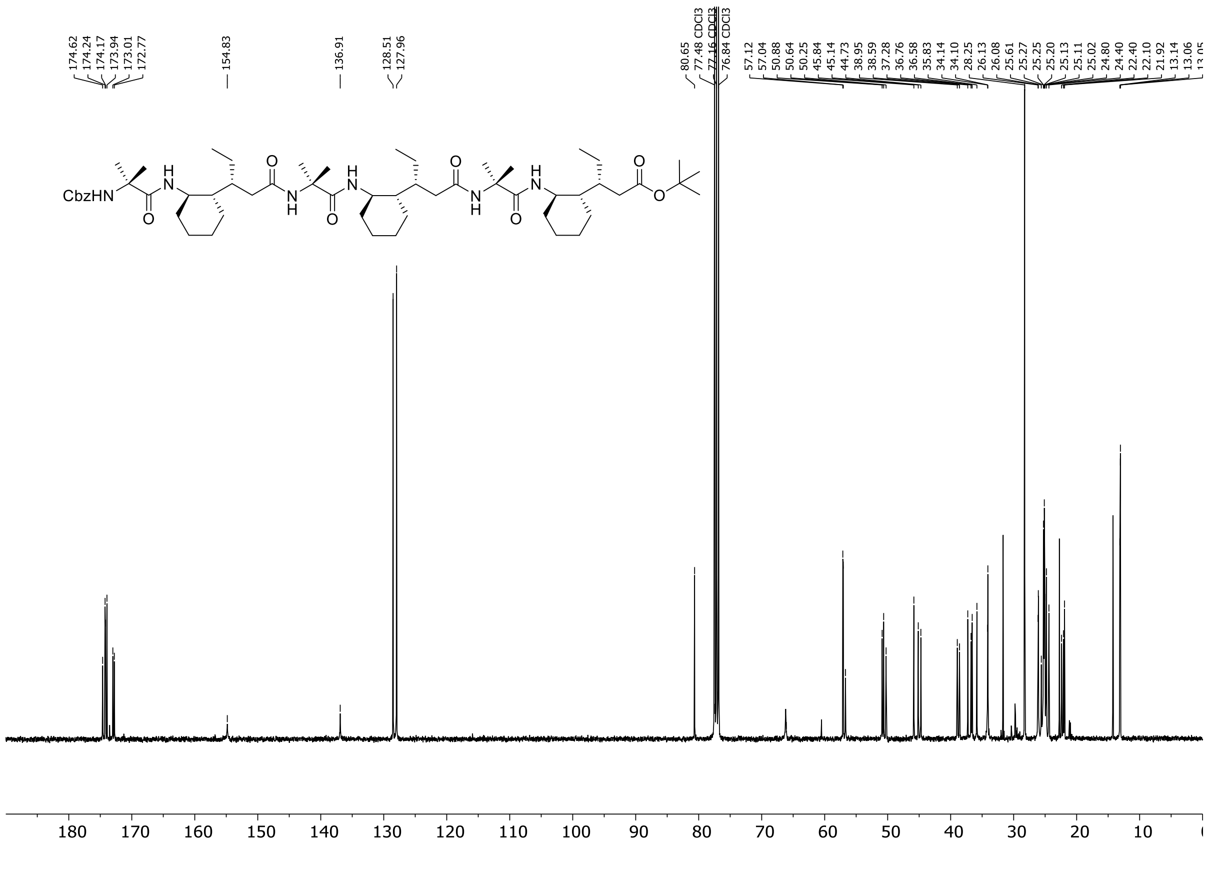

CDCl$_3$ ^{1}H (400 MHz) and ^{13}C (101 MHz) of **P25**

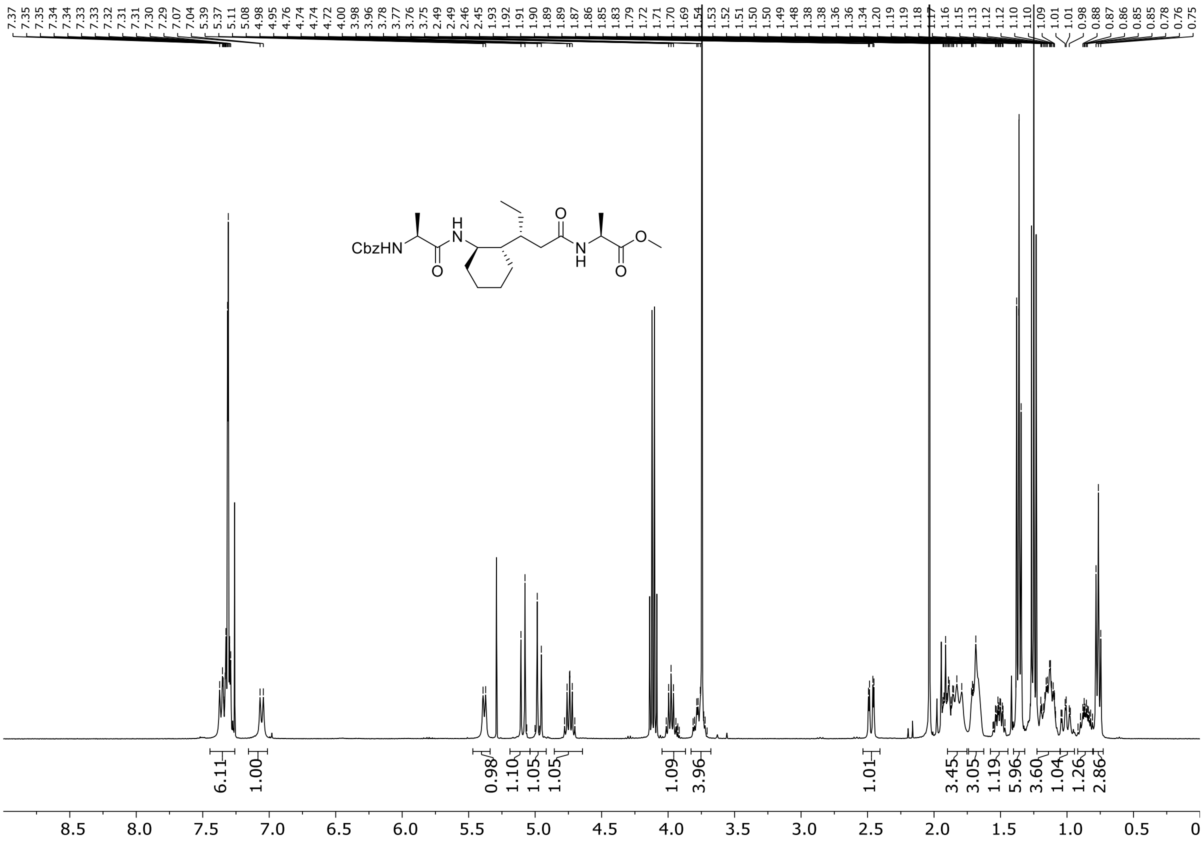

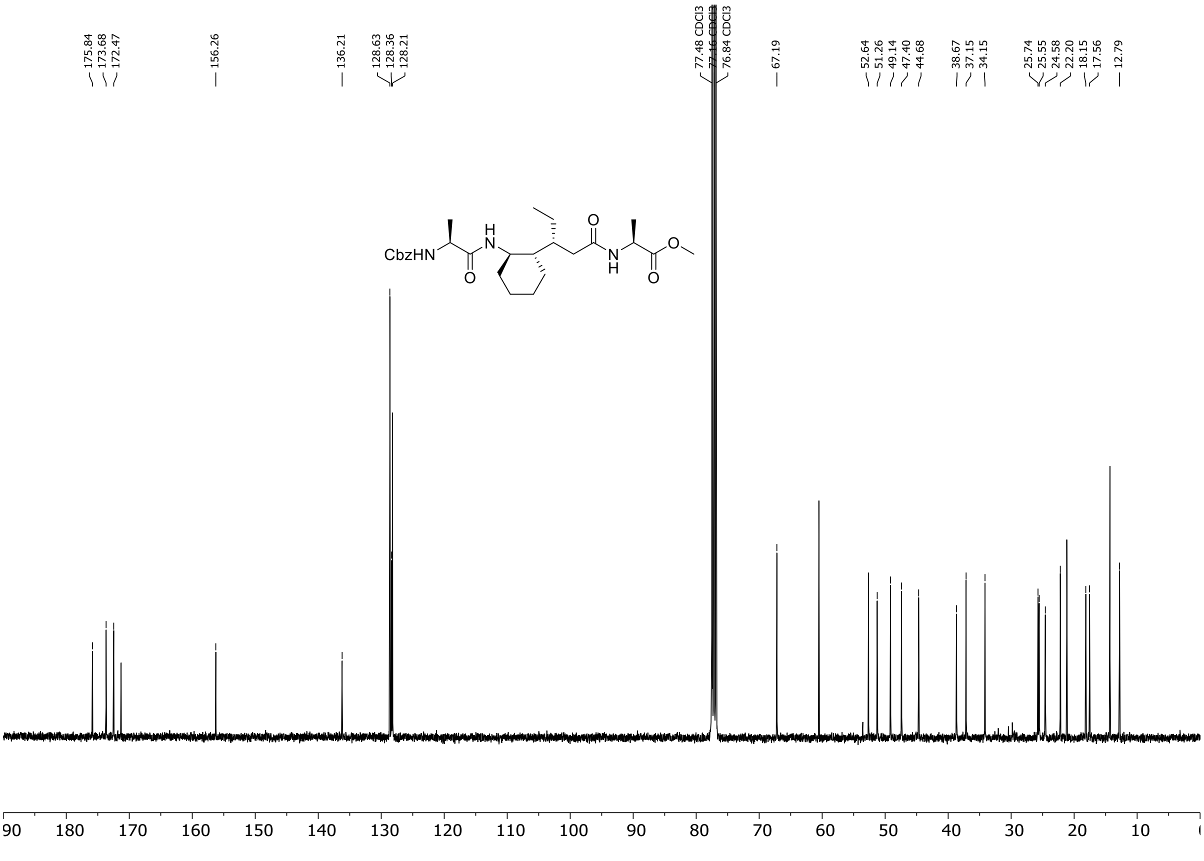

CDCl$_3$ ^{1}H (400 MHz) and ^{13}C (101 MHz) of **Heptameric Foldamer Z-A-X-A-A-A-X-A-OMe**

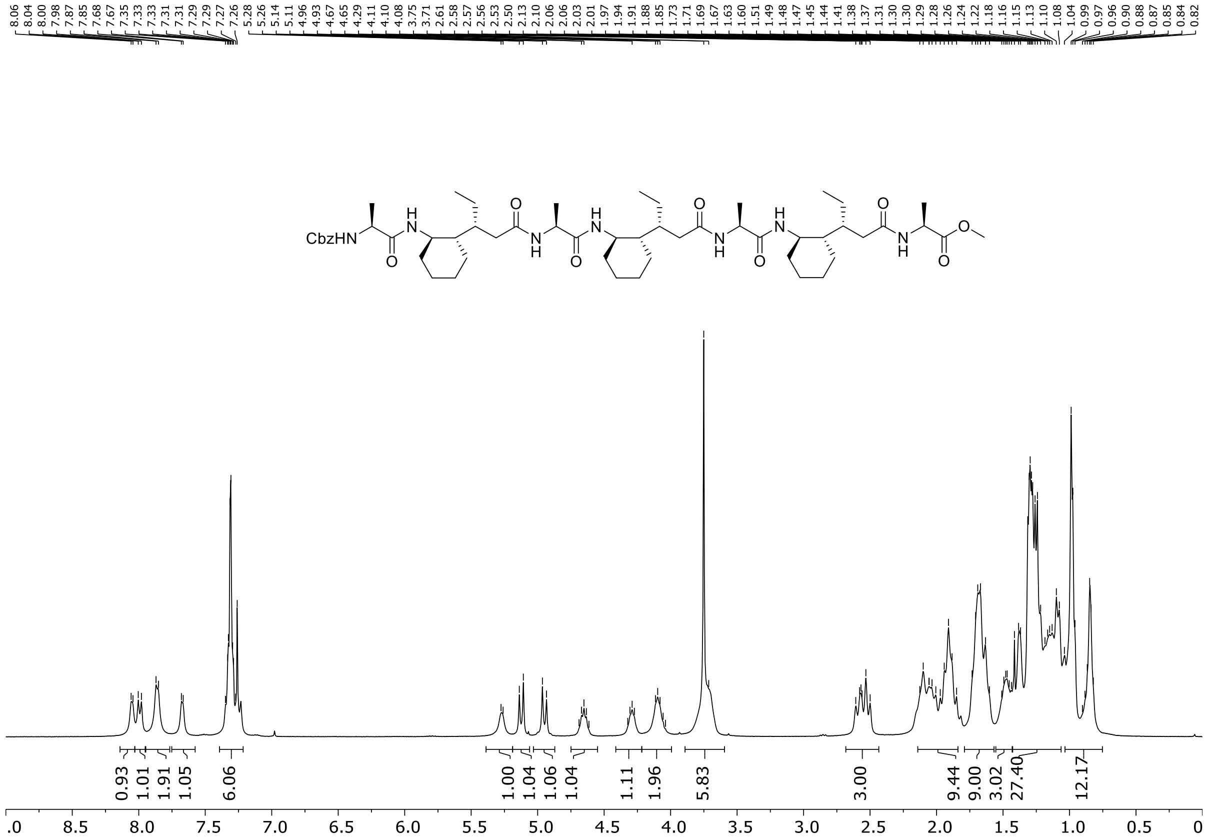

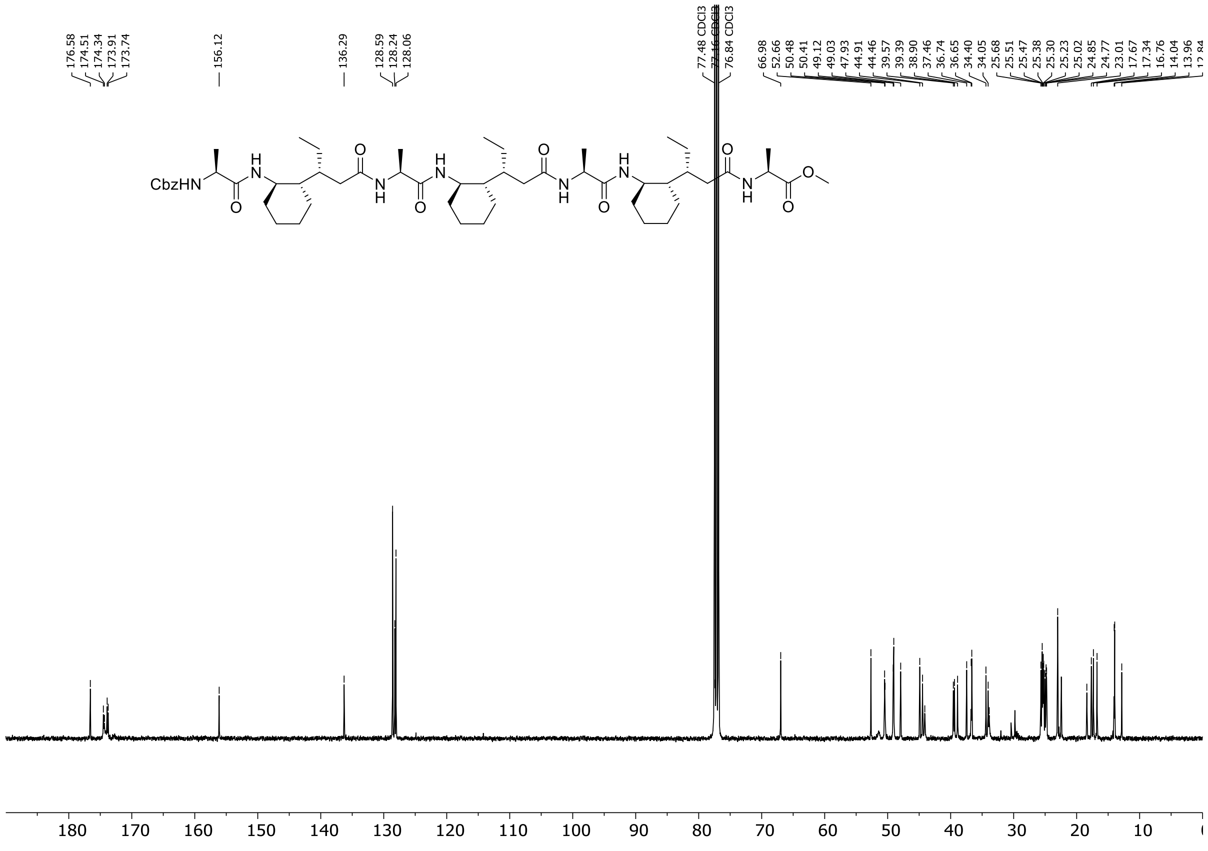

CDCl$_3$ ^{1}H (400 MHz) and ^{13}C (101 MHz) of **P26**

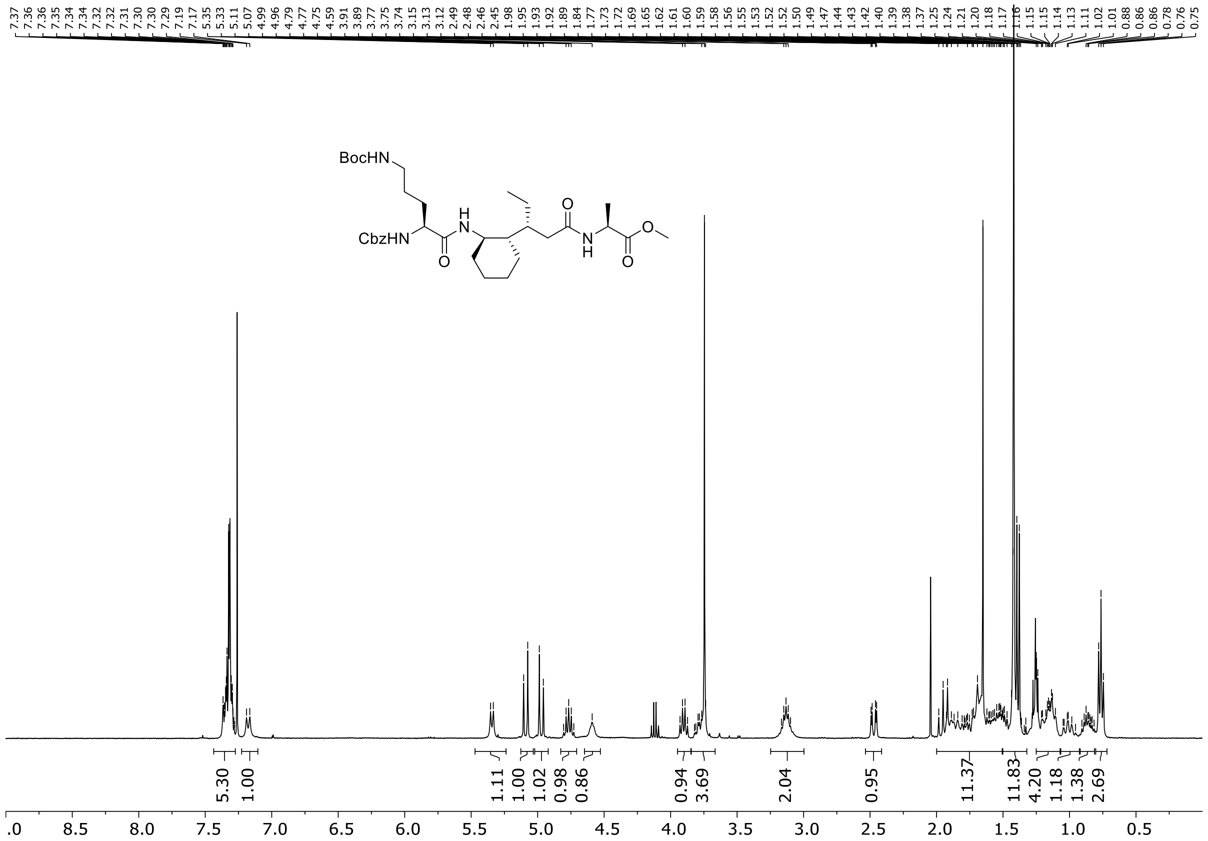

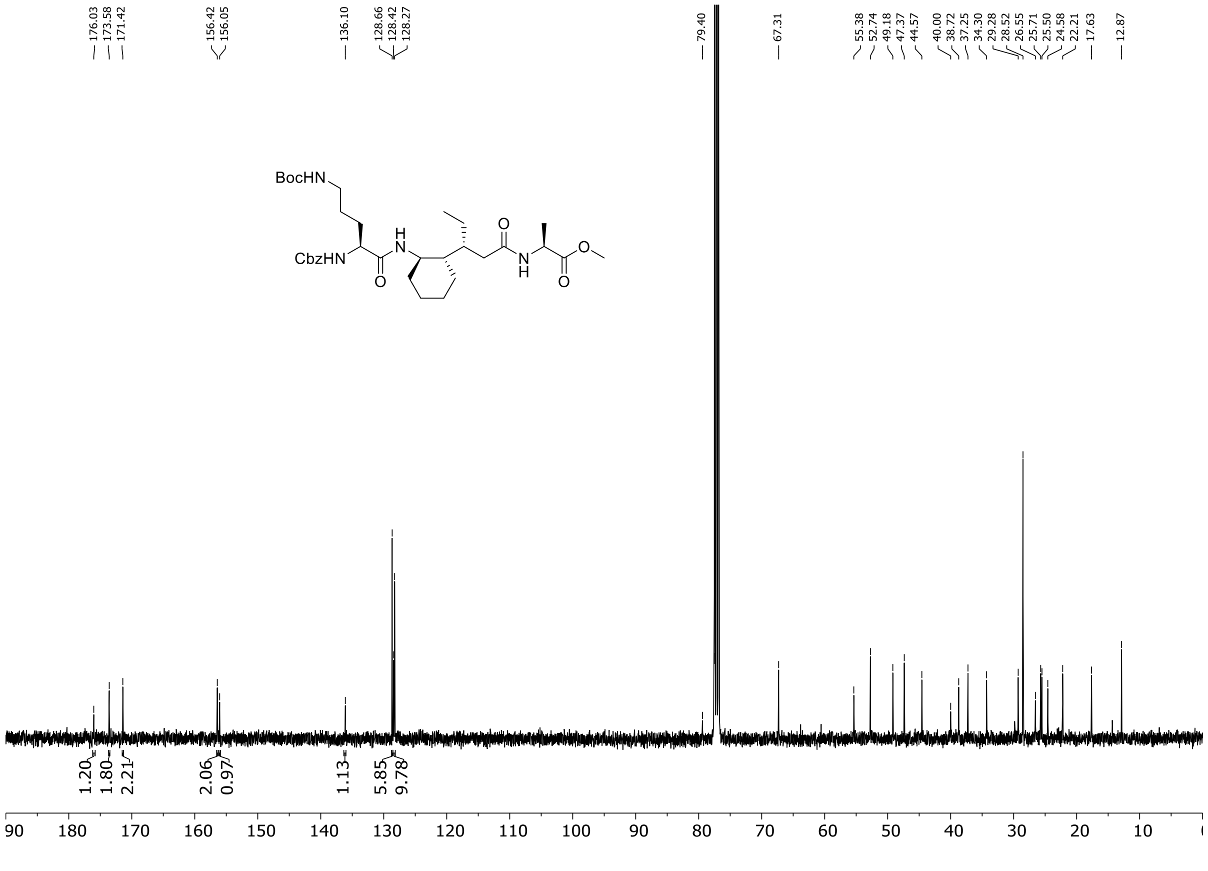

CDCl$_3$ ^{1}H (400 MHz) and ^{13}C (101 MHz) of **P27**

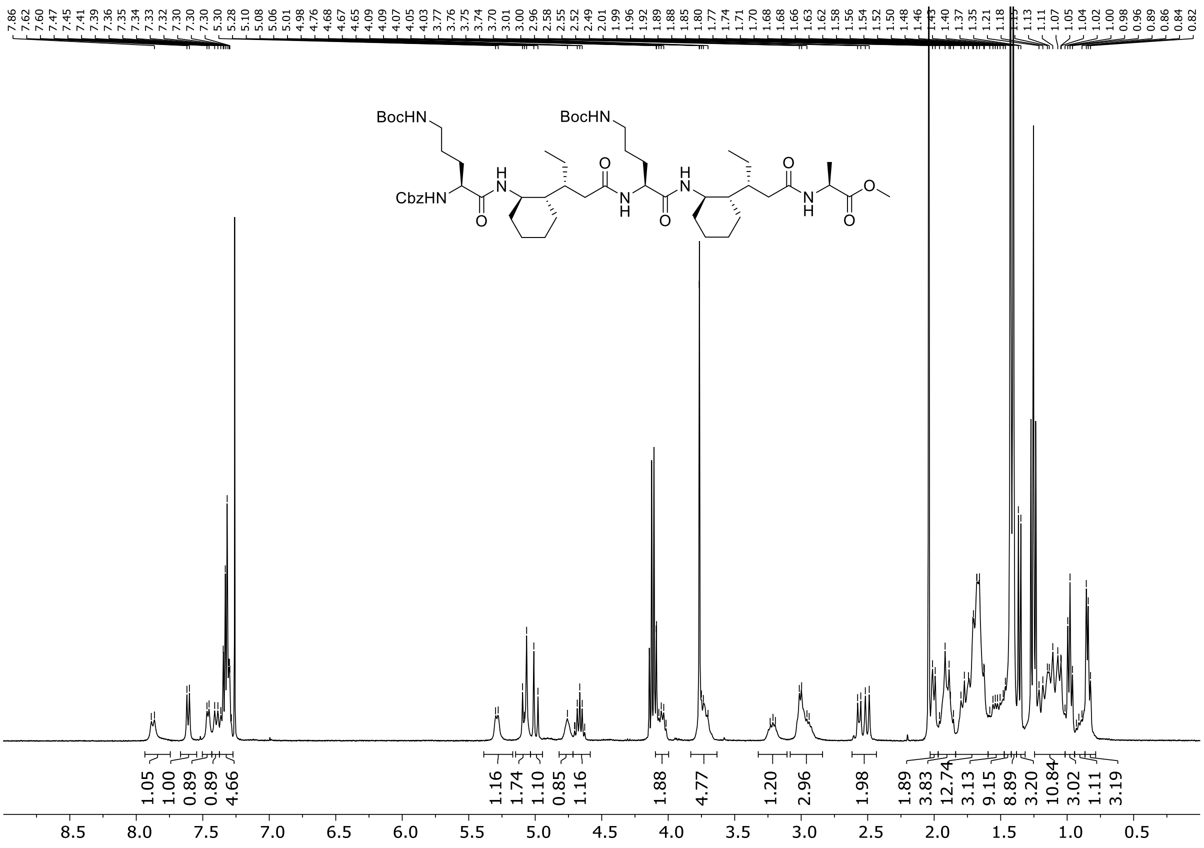

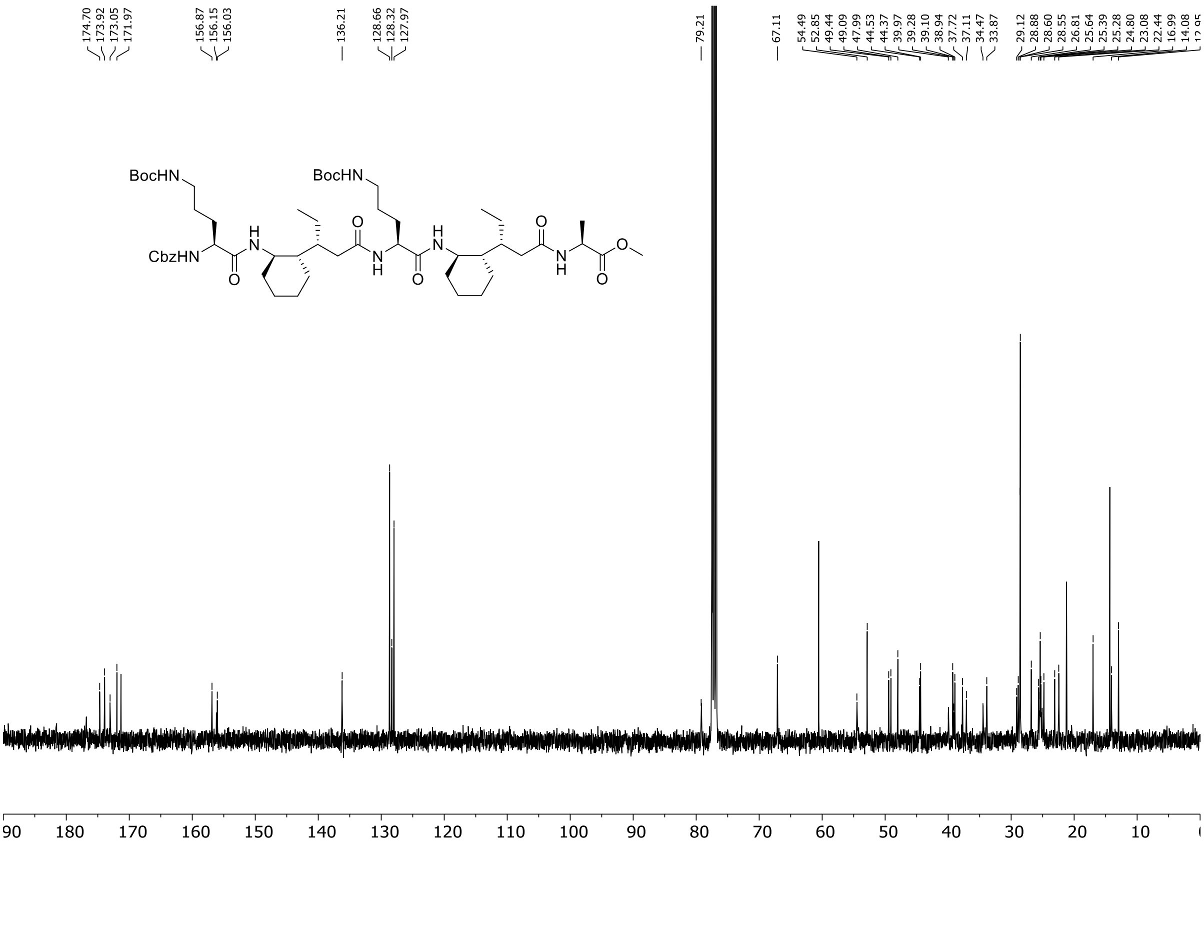

CDCl$_3$ ^{1}H (400 MHz) and ^{13}C (101 MHz) of **P28**

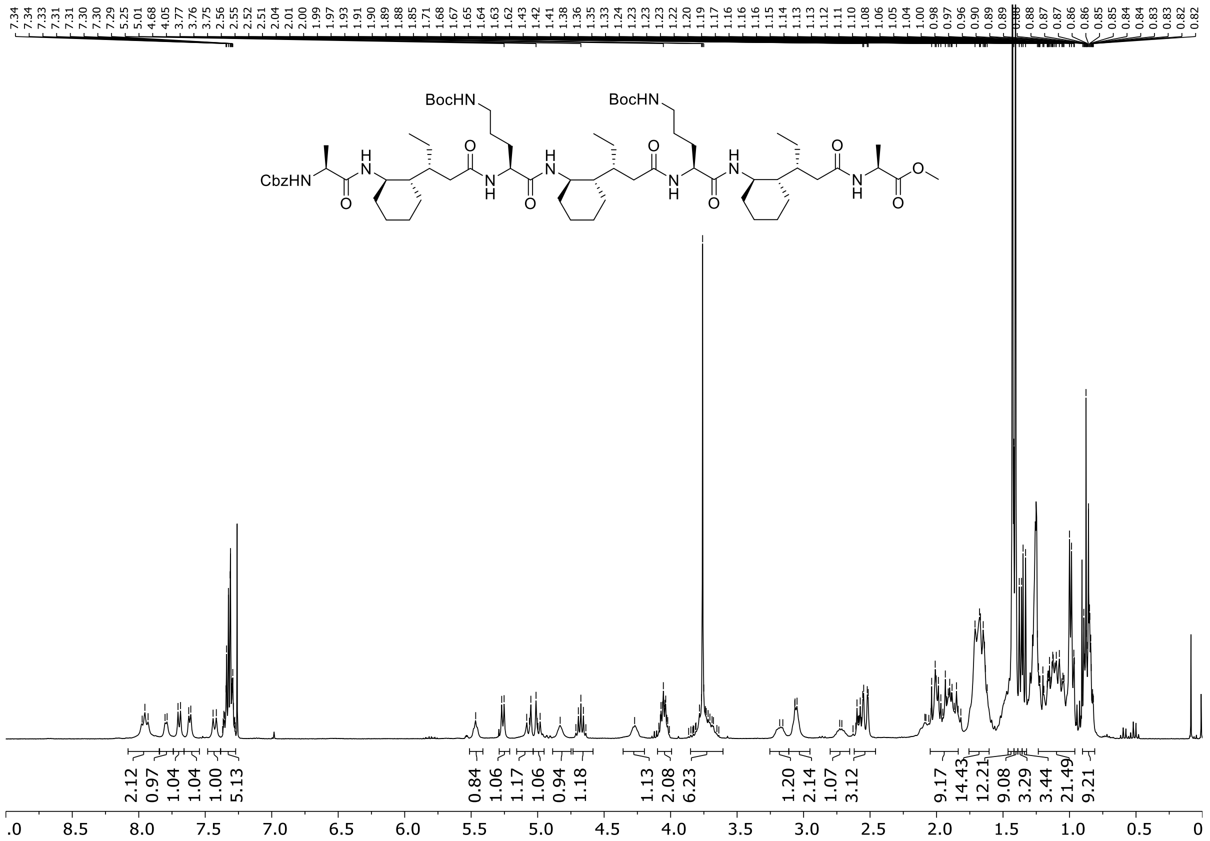

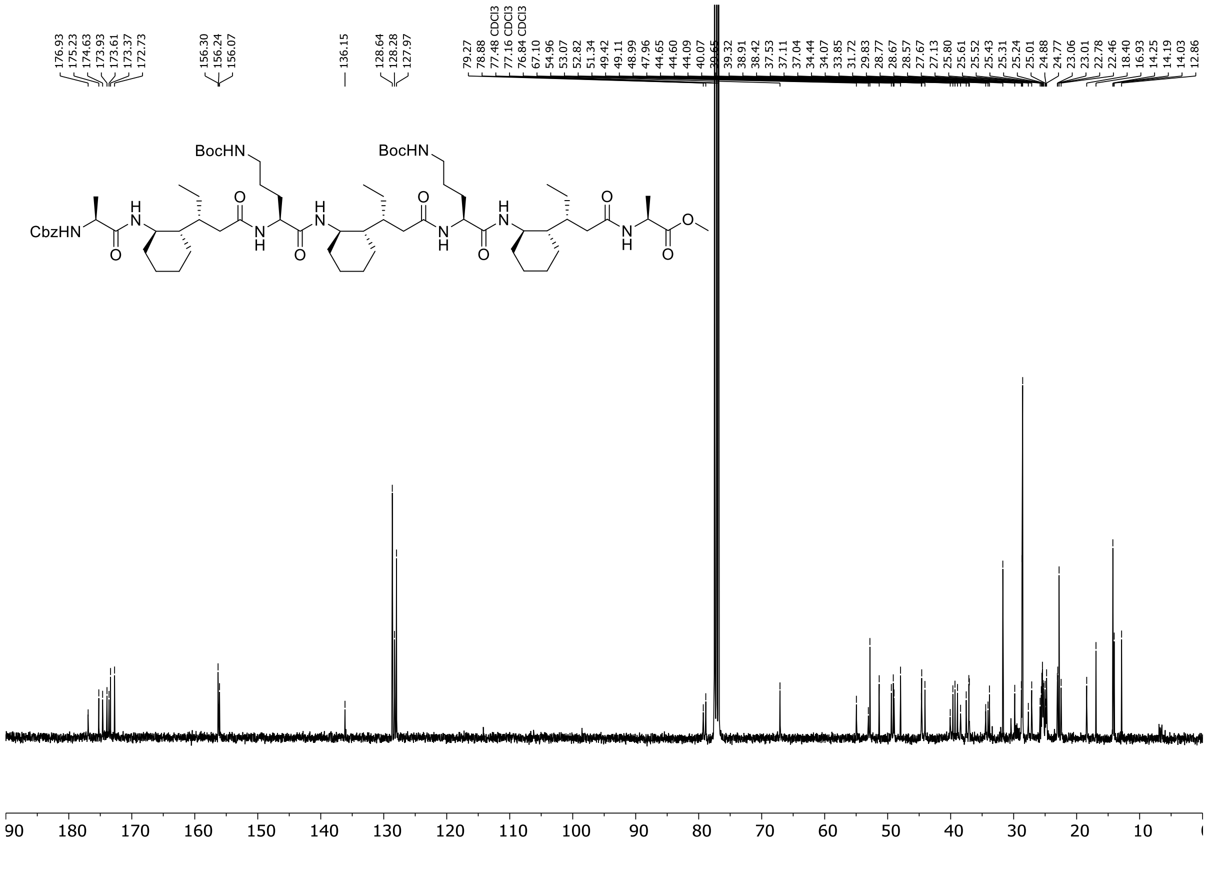

CDCl$_3$ ^{1}H (400 MHz) and ^{13}C (101 MHz) of **P31**

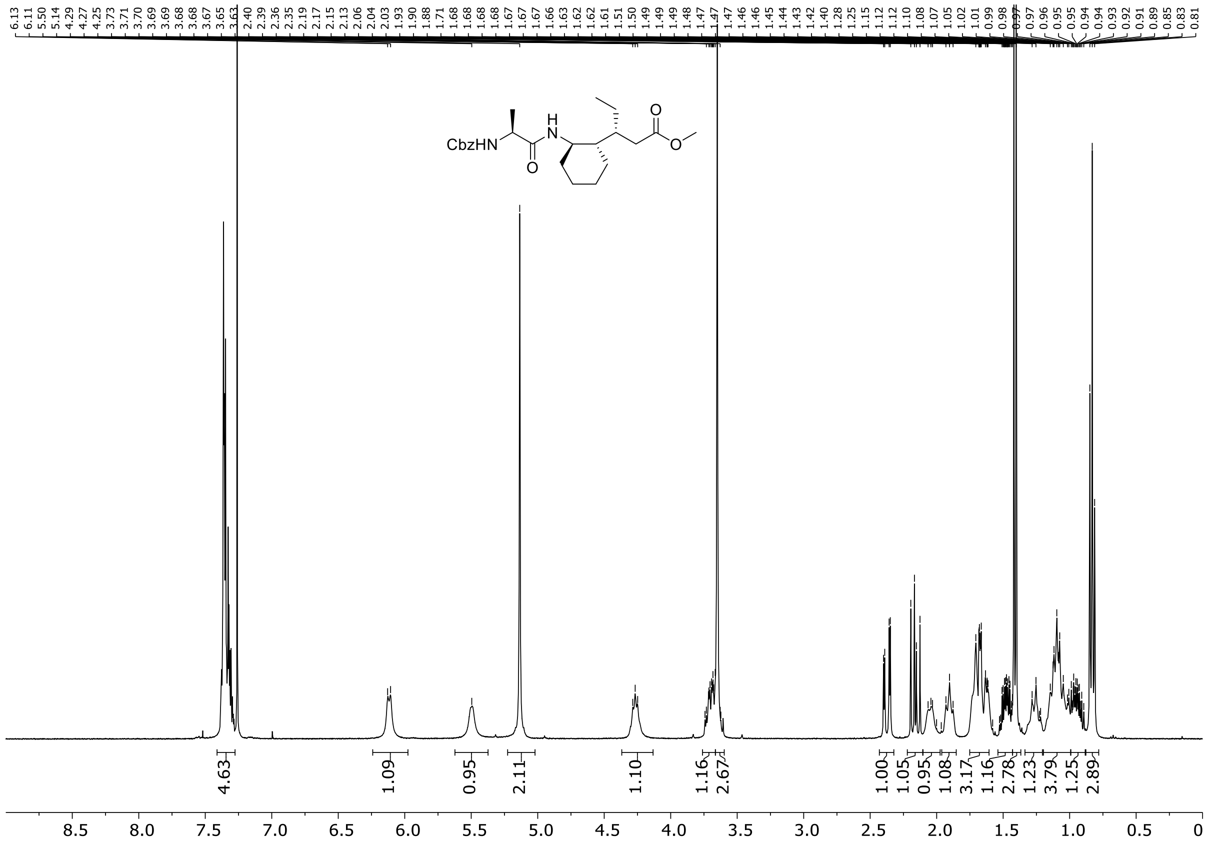

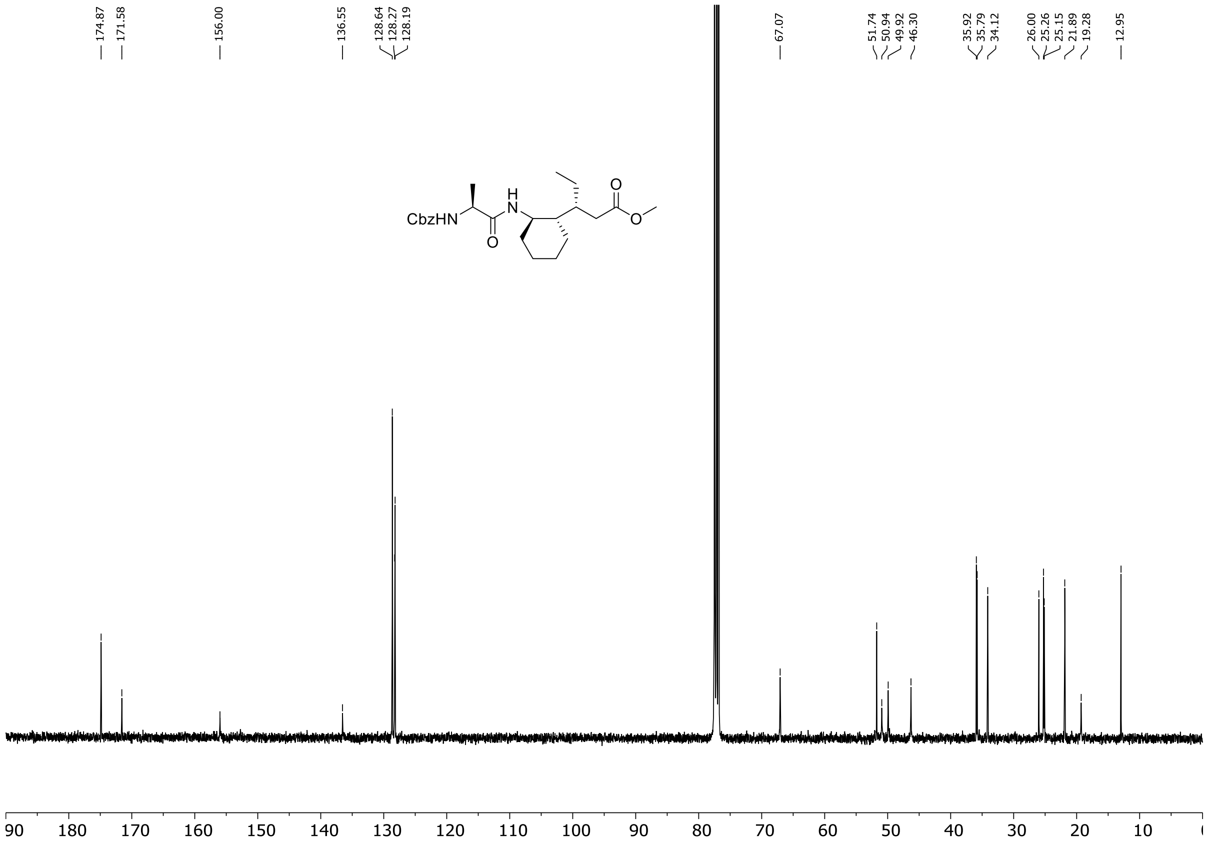

CDCl$_3$ ^{1}H (400 MHz) and ^{13}C (101 MHz) of **P32**

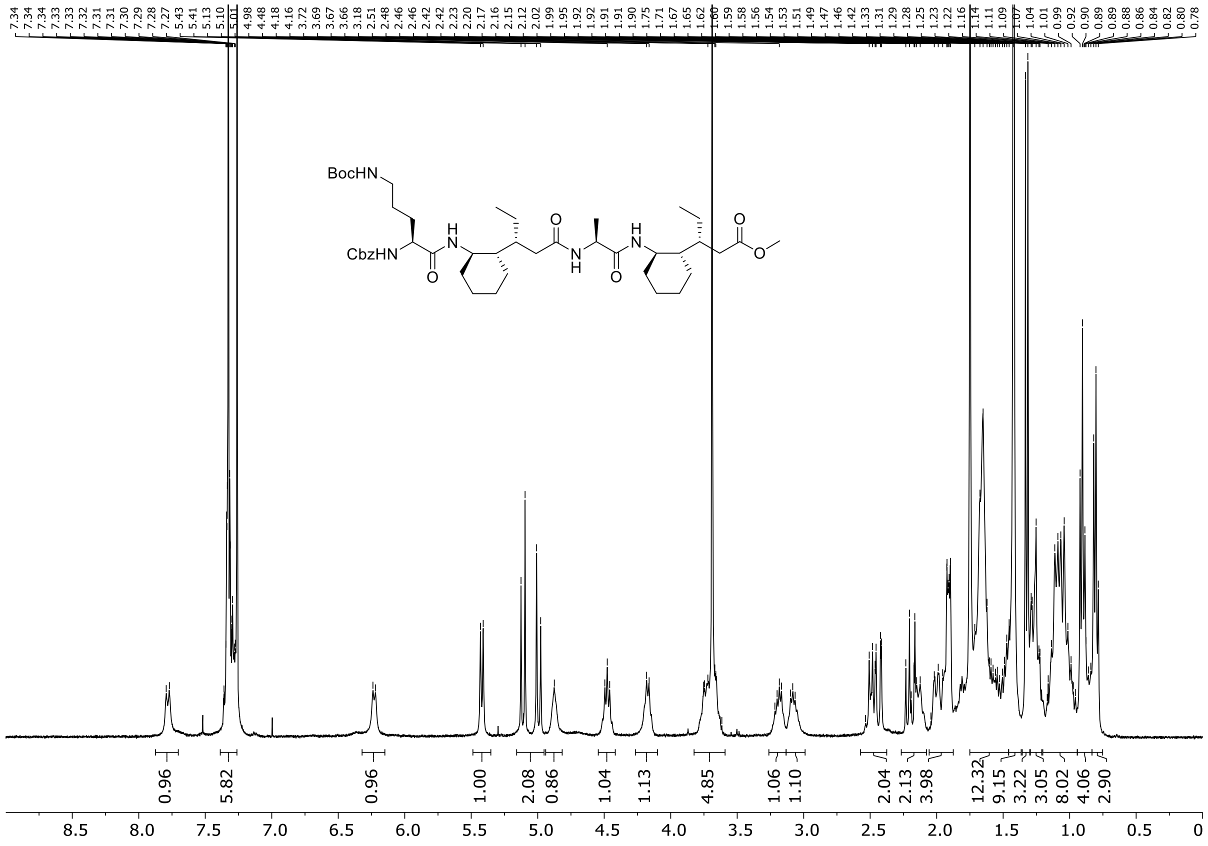

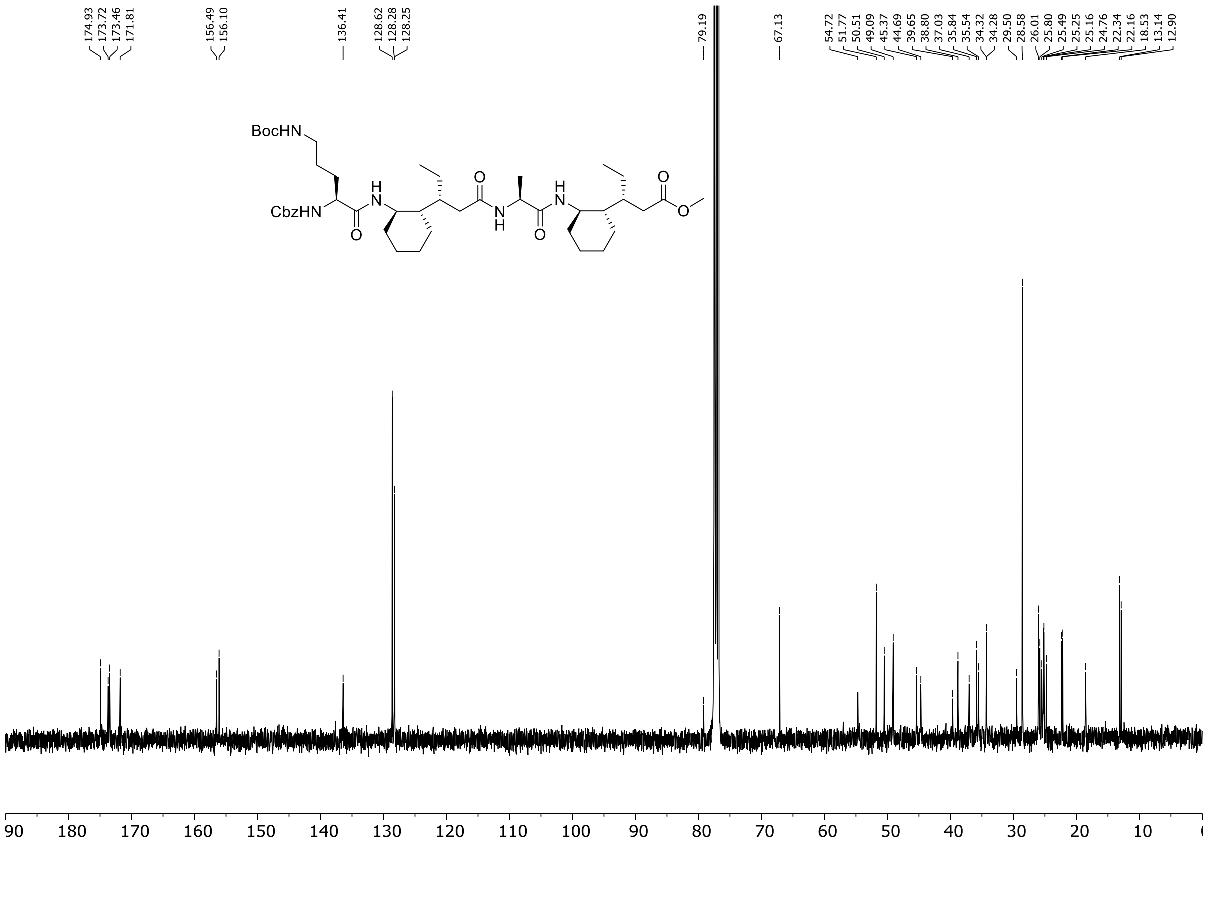

CDCl$_3$ ^{1}H (400 MHz) and ^{13}C (101 MHz) of **P33**

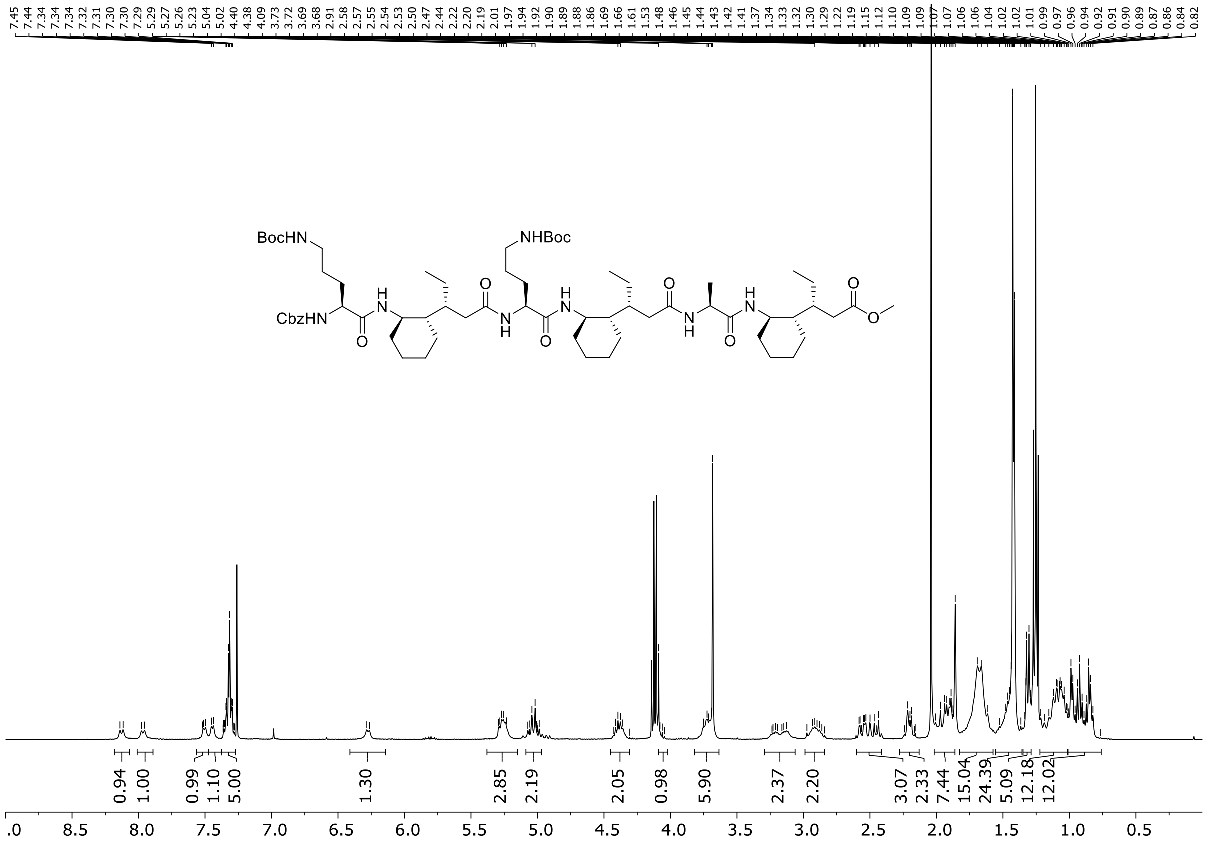

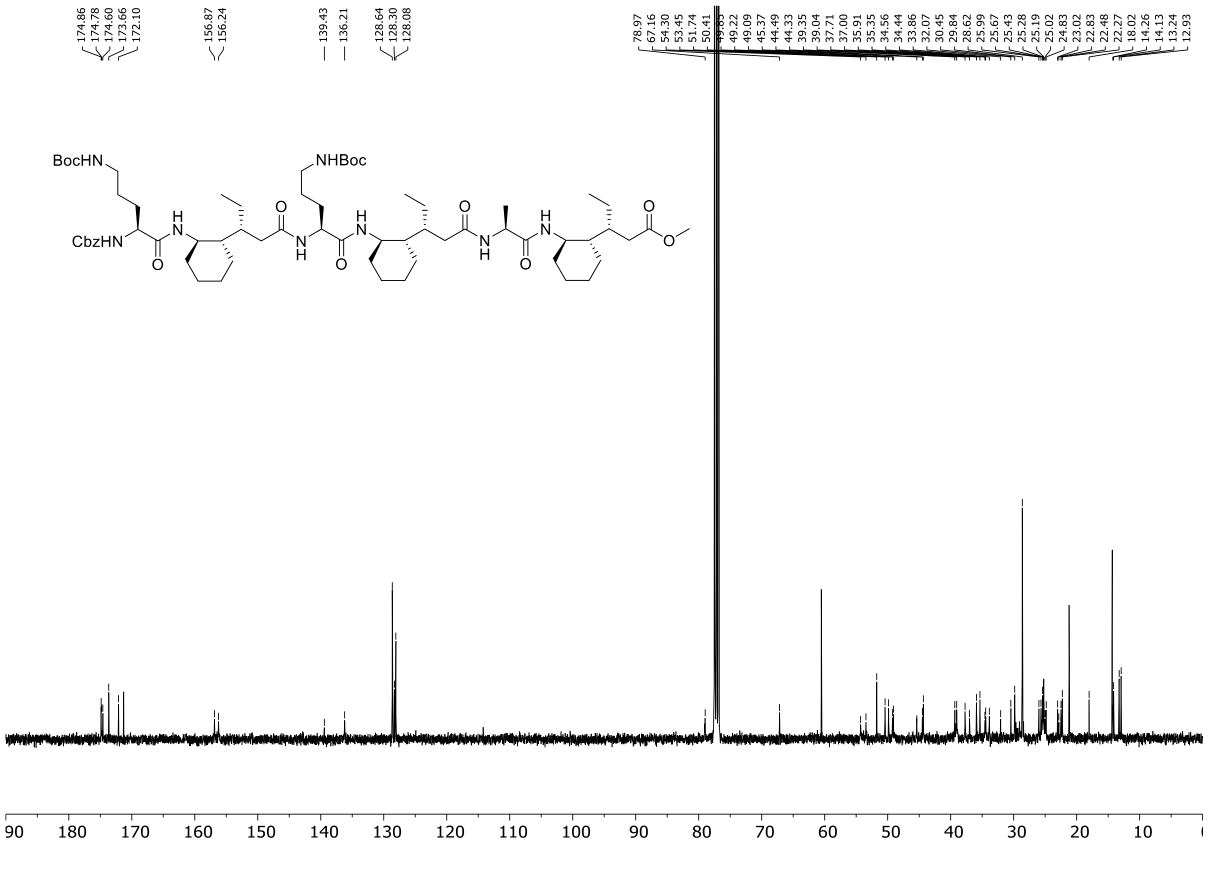

CDCl$_3$ ^{1}H (400 MHz) and ^{13}C (101 MHz) of **P34**

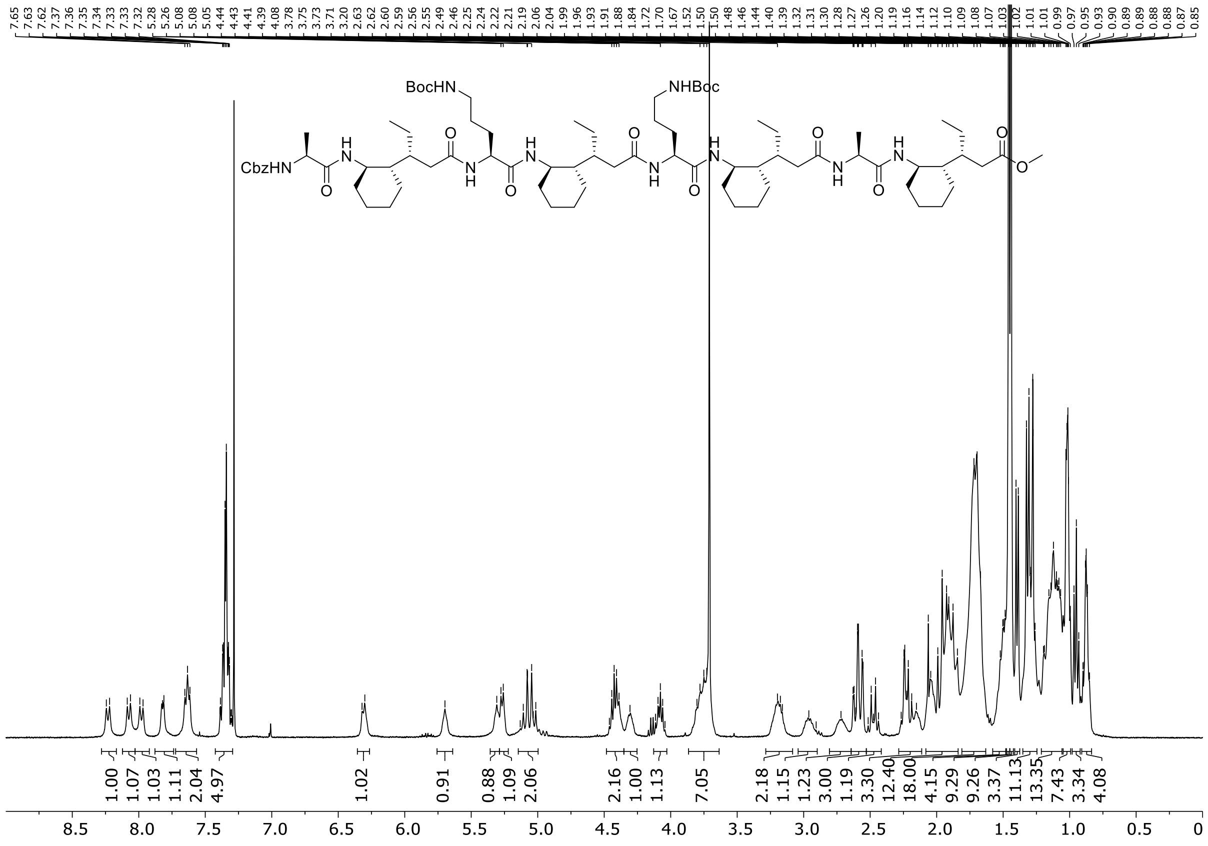

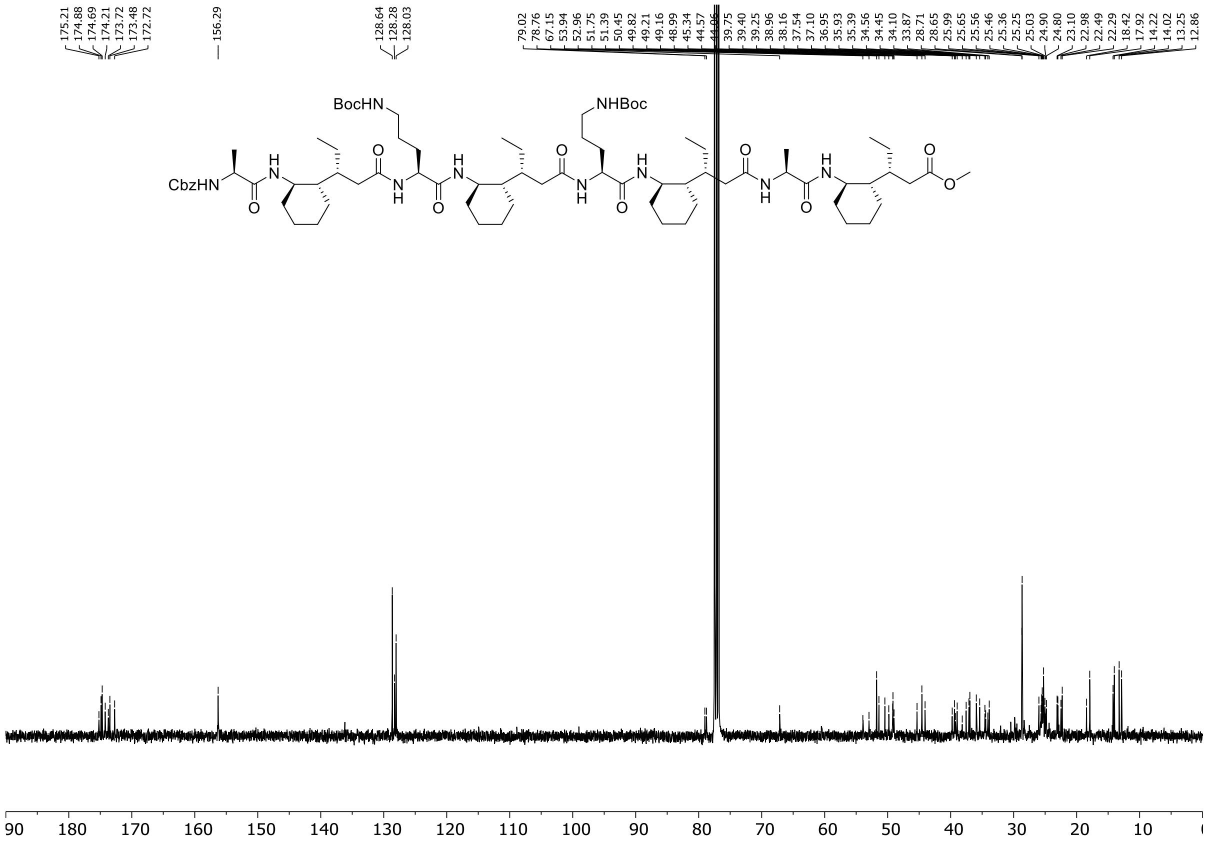

6.0 X-Ray Crystallography

The crystal of Foldamer **2** was obtained from the diffusion of $CHCl_3/CH_3CN$. The crystal of Foldamer Z-A-X-A-X-A-X-A-OMe was obtained from the diffusion of EtOAc/Heptane. The crystal of Foldamer **7** was obtained from slow evaporation of EtOAc, CH_3CN, dichloroethane, methanol, heptane, diethyl ether, Diisopropyl ether. The structures were solved by direct methods using SHELXT[S1] and refined against F^2 on all data by full-matrix least squares with SHELXL[S2], all performed within the OLEX2 suite [S3], following established refinement strategies.[S4] Most of the non-H atoms were refined with anisotropic temperature parameters, and the disordered ones were refined with isotropic temperature parameters. All hydrogen atoms were included in the model at geometrically calculated positions and refined using a riding model. In the case of the foldamer **2**, SHELX ISOR, SIMU and DFIX instructions were used in the refinement of some side chains and FLAT instructions used to geometrically restrain benzene rings. The contribution of the electron density associated with disordered solvent molecules, which could not be modelled with discrete atomic positions, was handled using the solvent mask functionality within OLEX2. Crystallographic data have been deposited with the CCDC.

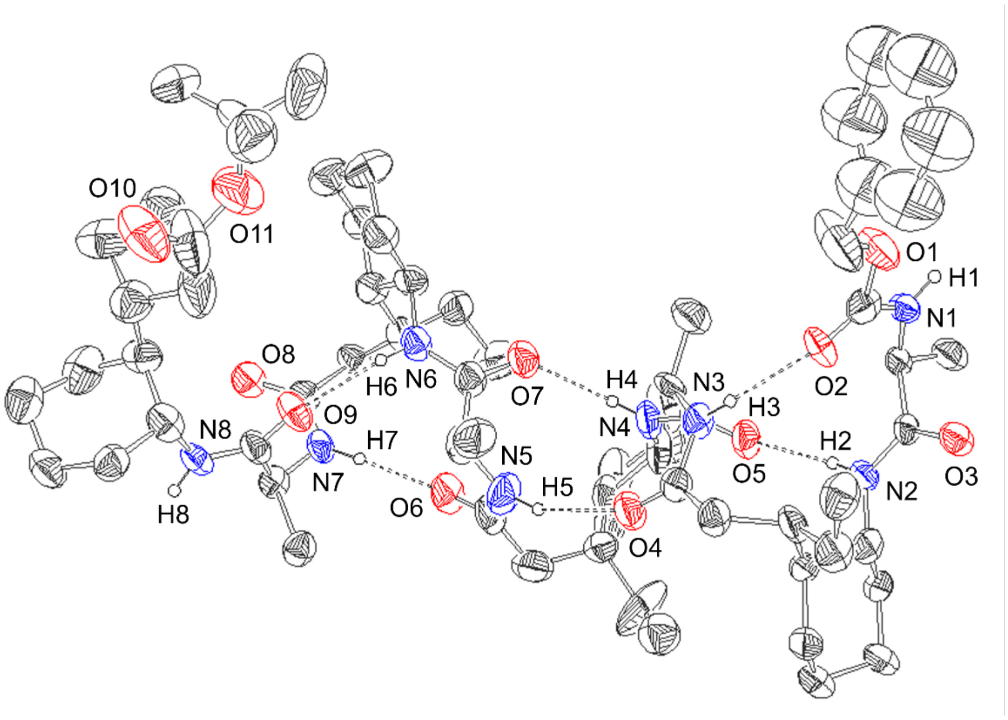

Figure SI9. The molecular structure of octamer **2** (CCDC 2252028). Thermal ellipsoids displayed at the 50% probability level. For clarity, only one of the two molecules in the asymmetric unit is shown and only hydrogen atoms on hetero atoms are pictured as spheres of arbitrary radii.

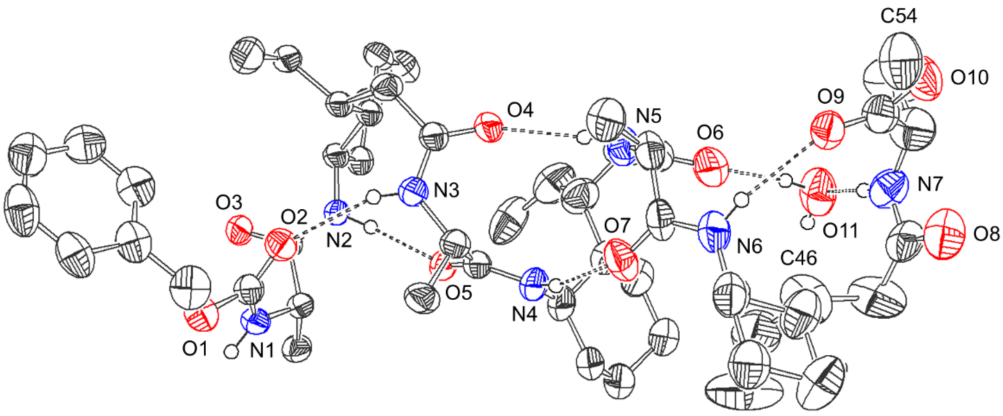

Figure SI10. The molecular structure of heptamer Z-A-X-A-X-A-X-A-OMe (CCDC : 2251914). Thermal ellipsoids displayed at the 50% probability level. Hydrogen atoms are pictured as spheres of arbitrary radii (and most have been omitted for clarity). The terminal –$C(CH_2CH_3)CH_2CONHC(CH_3)COOCH_3$ group (C46, C47, C48, C49, C50, O8, N7 C51, C52, C53, O9, O10, C54) is disordered across two positions, and the position of highest relative occupancy (57%) is displayed. The water molecule (O11) is also disordered across three positions, and the position of highest relative occupancy (57%) is displayed.

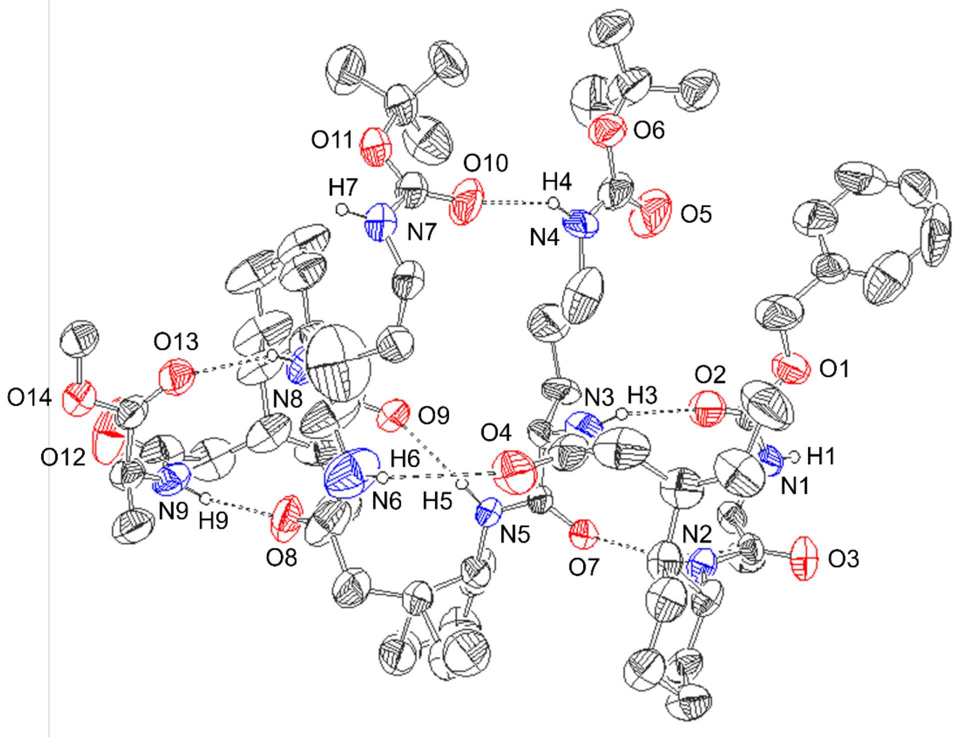

Figure SI11. The molecular structure of bis-Boc-ornithine heptamer 7 (CCDC : 2252029) . Thermal ellipsoids displayed at the 50% probability level. For clarity, only one of the two molecules in the asymmetric unit is shown and only hydrogen atoms on hetero atoms are pictured as spheres of arbitrary radii.

XRD Methodology

Octamer 2

A single crystal (0.03 x 0.06 x 0.28 mm) was mounted on a nylon loop and data measurements were made at 100 K using a Rigaku Synergy diffractometer equipped with a Cu microsource tube (CuKα = 1.54184Å), a Hypix6000HE photon counting detector and an Oxford Cryosystems Cryostream. Crystallographic details are provided in Table SI3. The structure was additionally validated by periodic dispersion-corrected DFT calculations using Quantum Espresso[S5], following the approach of van de Streek[S6]: a fixed-cell optimisation of the non-disordered component of the structure gave an optimised structure that yielded a Mercury[S8] 15-molecule overlay RMS value of 0.105 Å with the input structure.

Heptamer Z-A-X-A-X-A-X-A-OMe : A single crystal of the heptamer (0.20×0.06×0.03 mm) was held in a MiTeGen loop, and measurements were made at 100 K using a Rigaku 007 HF four-circle diffractometer and monochromated CuKα radiation (λ = 1.54178 Å) with a hybrid pixel array HyPix-6000HE detector[S7]. The sample temperature was controlled with an Oxford Cryosystems 800 Series CryoStream. Crystallographic details are provided in Table SI3.

Bis-Boc(Orn) Heptamer 7: A single crystal (0.02 x 0.05 x 0.07 mm) was mounted on a nylon loop and data measurements were made at 100 K using a Rigaku Synergy diffractometer equipped with a Cu microsource tube (CuKα = 1.54184Å), a Hypix6000HE photon counting detector and an Oxford Cryosystems Cryostream. Crystallographic details are provided in Table SI3. The structure was additionally validated by periodic dispersion-corrected DFT calculations using Quantum Espresso, following the approach of van de Streek: a fixed-cell optimisation of the structure gave an optimised structure that yielded a Mercury[S8] 15-molecule overlay RMS value of 0.089 Å with the input structure.

S1 G. M. Sheldrick, *Acta Crystallogr.* 2015, **A71**, 3-8.

S2 G. M. Sheldrick, *Acta Crystallogr.* 2015, **C71**, 3-8.

S3 O. V. Dolomanov, L. J. Bourhis, R. J. Gildea, J. A. K. Howard, H. Puschmann, *J. Appl. Crystallogr*. 2009, **42**, 339-341

S4 P. Müller,*Cryst. Rev*. 2009, 15.

S5 P. Giannozzi, S. Baroni, N. Bonini, M. Calandra, R. Car, C. Cavazzoni, D. Ceresoli, G.L. Chiarotti, M. Cococcioni, I. Dabo et al. *J. Phys. Condes. Matter.,* 2009, **21**, 19

S6 J. van de Streek and M. A. Neumann, *Acta Crystallogr., Sect. B: Struct. Sci*., 2010, **66**, 544-558

S7 S. J. Coles, J. Simon J, D. R. Allan, C. M. Beavers, S. J. Teat, and S. J. W. Holgate. In, *Structure and Bonding.* Berlin, Heidelberg. Springer, pp. 1-72.

S8 C. F. Macrae, I. Sovago, S. J. Cottrell, P. T. A. Galek, P. McCabe, E. Pidcock, M. Platings, G. P. Shields, J. S. Stevens, M. Towler and P. A. Wood, J. Appl. Cryst. 2020, 53, 226-235

Crystallographic Data

Table SI3. Crystallographic data for **ZAXAXAXAOMe**, octamer **2** and heptamer **7**

Compound	**ZAXAXAXAOMe**	Octamer **2**	Heptamer **7**
Empirical formula	$C_{54}H_{89}N_7O_{11}$	$C_{68}H_{112}N_8O_{11}$	$C_{68}H_{113}N_9O_{14}$
Formula weight	1012.32	1217.65	1280.67
Temperature/K	100(2)	100.00(10)	100.15
Crystal system	orthorhombic	monoclinic	monoclinic
Space group	$P2_12_12_1$	$P2_1$	$P2_1$
a/Å	8.91084(12)	8.9665(5)	9.7832(2)
b/ Å	12.2957(2)	32.2159(17)	55.6208(12)
c/ Å	53.1813(7)	25.6602(15)	14.0694(2)
α/°	90	90	90
β/°	90	91.943(6)	90.3234(18)
γ/°	90	90	90
Volume/ Å^3	5826.78(15)	7408.0(7)	7655.7(3)
Z	4	4	4
ρ_{calc}g/cm^3	1.154	1.092	1.111
μ/mm^{-1}	0.650	0.589	0.627
F(000)	2200.0	2656.0	2784.0
Crystal size/mm^3	0.20 × 0.06 × 0.025	0.03 × 0.06 × 0.28	0.02 × 0.05 × 0.07
Radiation	Cu Kα (λ = 1.54178)	Cu Kα (λ = 1.54184)	CuKα (λ = 1.54184)
2Θ range for data collection/°	6.648 to 140.128	5.486 to 157.134	6.282 to 152.85
Index ranges	$-10 \le h \le 10$, $-14 \le k \le 14$, $-60 \le l \le 64$	$-11 \le h \le 11$, $-40 \le k \le 40$, $-20 \le l \le 32$	$-12 \le h \le 11$, $-69 \le k \le 70$, $-17 \le l \le 16$
Reflections collected	86919	62172	122167
Independent reflections	11024 [R_{int} = 0.0631, R_{sigma} = 0.0271]	26732 [R_{int} = 0.1053, R_{sigma} = 0.1060]	29275 [R_{int} = 0.1290, R_{sigma} = 0.1098]
Data/restraints/parameters	11024/1411/795	26732/181/1591	29275/1/1663
Goodness-of-fit on F^2	1.070	1.023	1.040
Final R indexes [I>=2σ (I)]	R_1 = 0.0649, wR_2 = 0.1731	R_1 = 0.1135, wR_2 = 0.3023	R_1 = 0.0908, wR_2 = 0.2340
Final R indexes [all data]	R_1 = 0.0730, wR_2 = 0.1792	R_1 = 0.1386, wR_2 = 0.3254	R_1 = 0.1375, wR_2 = 0.2624
Largest diff. peak/hole/e Å^{-3}	0.56/–0.22	0.81/-0.51	0.35/-0.27
Flack parameter	0.22(7)	0.48(18)	0.46(15)

7.0 Solution Conformational Analysis and Modelling of Foldamer 2

7.1 General Protocol of NMR Assignment and Analysis

^{1}H NMR spectra were recorded at 500 and 950 MHz while ^{13}C NMR spectra were recorded at 125 and 237 MHz (Bruker Cryo500/950). Compound characterisations and assignments are based on 1D and 2D spectroscopies including ^{1}H, ^{13}C, ^{1}H pure-shift (tse-psyche), (pure-shift) HSQC, HMBC, (f1-pure-shift)-TOCSY and HSQC-TOCSY. All NMR experiments were run at 25°C except VT. All chemical shifts are quoted in parts per million (ppm) referenced to the residual solvent peaks ($CDCl_3$ at 7.26 ppm). The proton spin-simulation were performed in the module of *MestreNova* software package, for extracting $^{3}J_{HH}$ scalar coupling constants from TOCSY, 2DJ. For the determination of ROE distance restraints, 2D-ROESY spectra were recorded and processed on Bruker cryo500 and 950 MHz spectrometer with a 5mm TCI Cryoprobe in Oxford (abbr. Oxford cryo950). The default ROESY mixing time was 0.20 or 0.25 s, unless stated otherwise. For 2D spectra, number of scans were conducted between 4-16 under increments of 1024-2048 (f2-phase) and 256-1024 (f1-phase) depending on experiment. For each phase with sufficient signal to noise ratio (S/N), FID data was zero filled 4-fold and forward linear predicted 2-fold to increase digital resolution. Apodisation function was applied if stated. Each ROE cross-peak (η) was integrated and normalised to corresponding diagonal peak for quantitative measurement of distance restraint given by the equation, according to PANIC approach described by Macura, Hu and Butts.[1–3] Both ROE restraints observed from f1 and f2-trace were recorded.

$$\frac{\eta_{ref}}{\eta_i} = \frac{(r_{ref})^{-6}}{(r_i)^{-6}}$$

The interproton distance of **2.96 Å** between diaxial nucleus **17** and **18** on the N-terminal δ residue was adopted as the reference according to NMR-refined DFT calculations.

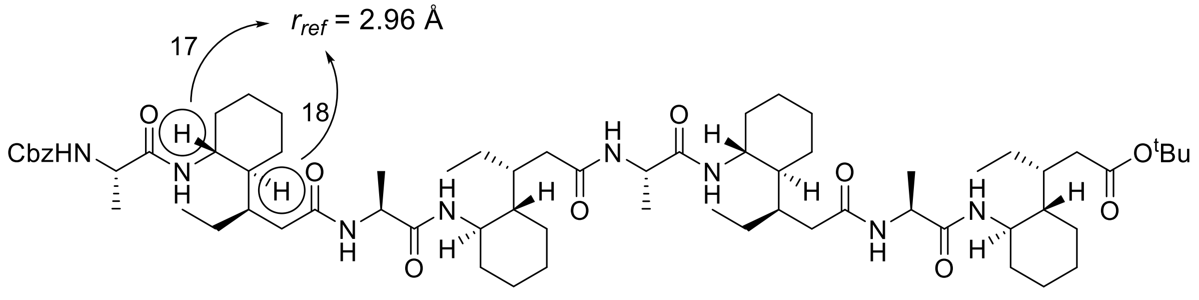

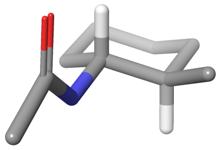

7.2 NMR Assignment and Spectra ($CDCl_3$, 500 MHz)

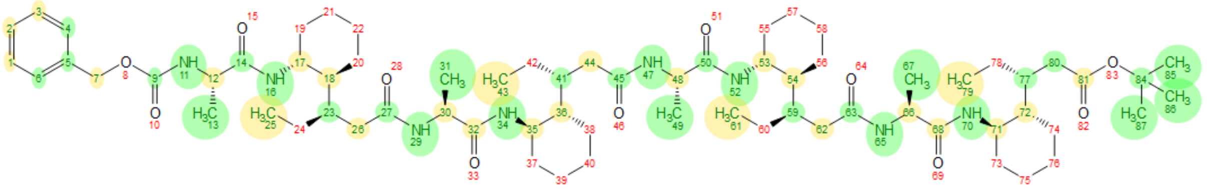

Assignments	
Atom	δ (ppm)
1 C	128.47
H	7.33
2 C	128.11
H	7.33
3 C	128.47
H	7.33
4 C	127.94
H	7.33
5 C	136.19
6 C	127.94
H	7.33
7 C	66.88
H'	4.99
H''	5.15
9 C	156.01
11 N	
H	5.31
12 C	51.21
H	4.13
13 C	18.31
H3	1.45
14 C	172.59
16 N	
H	7.94
17 C	49.11
H	3.80
18 C	37.36
H	2.07
19 C	
H2	

Assignments	
Atom	δ (ppm)
20 C	
H2	
21 C	
H2	
22 C	
H2	
23 C	43.91
H	
24 C	
H2	
25 C	12.69
H3	0.88
26 C	38.79
H'	1.90
H''	2.55
27 C	174.33
29 N	
H	7.67
30 C	50.26
H	4.36
31 C	17.55
H3	1.37
32 C	173.65
34 N	
H	8.19
35 C	49.11
H	3.73
36 C	36.66
H	2.16
37 C	
H2	

Assignments	
Atom	δ (ppm)
38 C	
H2	
39 C	
H2	
40 C	
H2	
41 C	44.25
H	
42 C	
H2	
43 C	13.83
H3	1.02
44 C	39.46
H'	1.95
H''	2.58
45 C	174.08
47 N	
H	7.99
48 C	50.08
H	4.39
49 C	
H3	1.31
50 C	173.99
52 N	
H	8.16
53 C	48.92
H	3.73
54 C	35.26
H	2.15
55 C	
H2	

Assignments	
Atom	δ (ppm)
56 C	
H2	
57 C	
H2	
58 C	
H2	
59 C	44.60
H	
60 C	
H2	
61 C	13.87
H3	1.01
62 C	39.36
H'	1.99
H''	2.61
63 C	173.54
65 N	
H	7.74
66 C	49.11
H	4.49
67 C	
H3	1.31
68 C	173.65
70 N	
H	6.27
71 C	50.33
H	3.68
72 C	36.35
H	2.09
73 C	
H2	

Assignments	
Atom	δ (ppm)
74 C	
H2	
75 C	
H2	
76 C	
H2	
77 C	45.06
H	
78 C	
H2	
79 C	12.81
H3	0.93
80 C	37.21
H'	2.12
H''	2.39
81 C	173.65
84 C	80.44
85 C	28.22
H3	
86 C	28.22
H3	
87 C	28.22
H3	

^{1}H (upper); **^{1}H pure-shift (tse-psyche)** (lower)

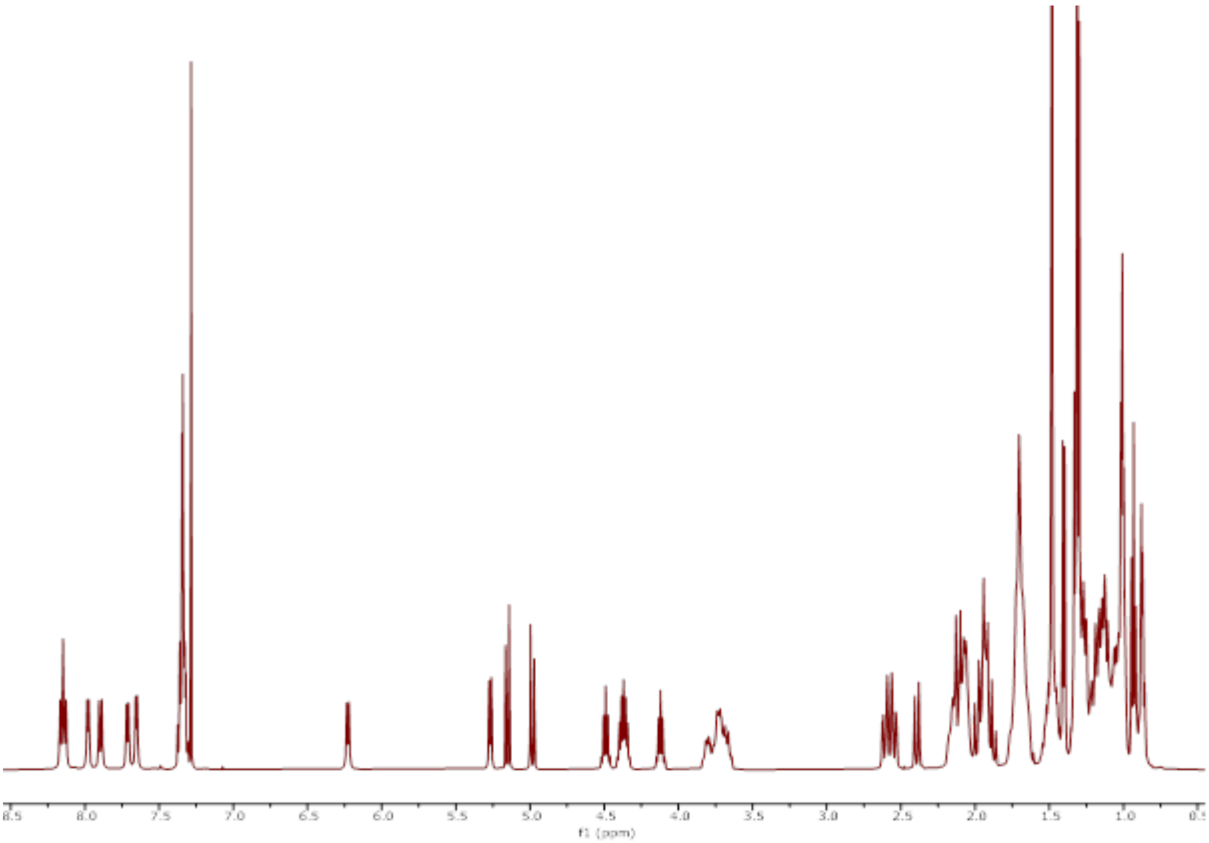

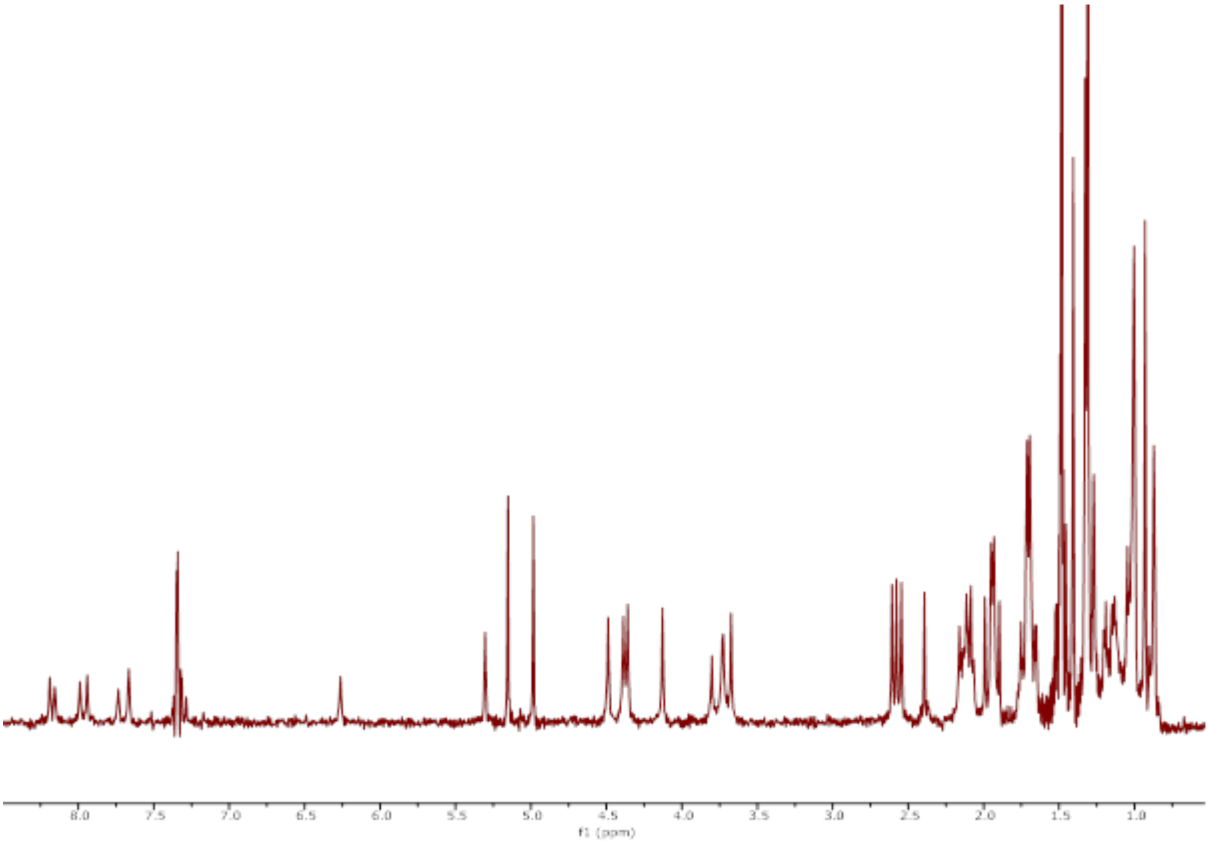

^{13}C

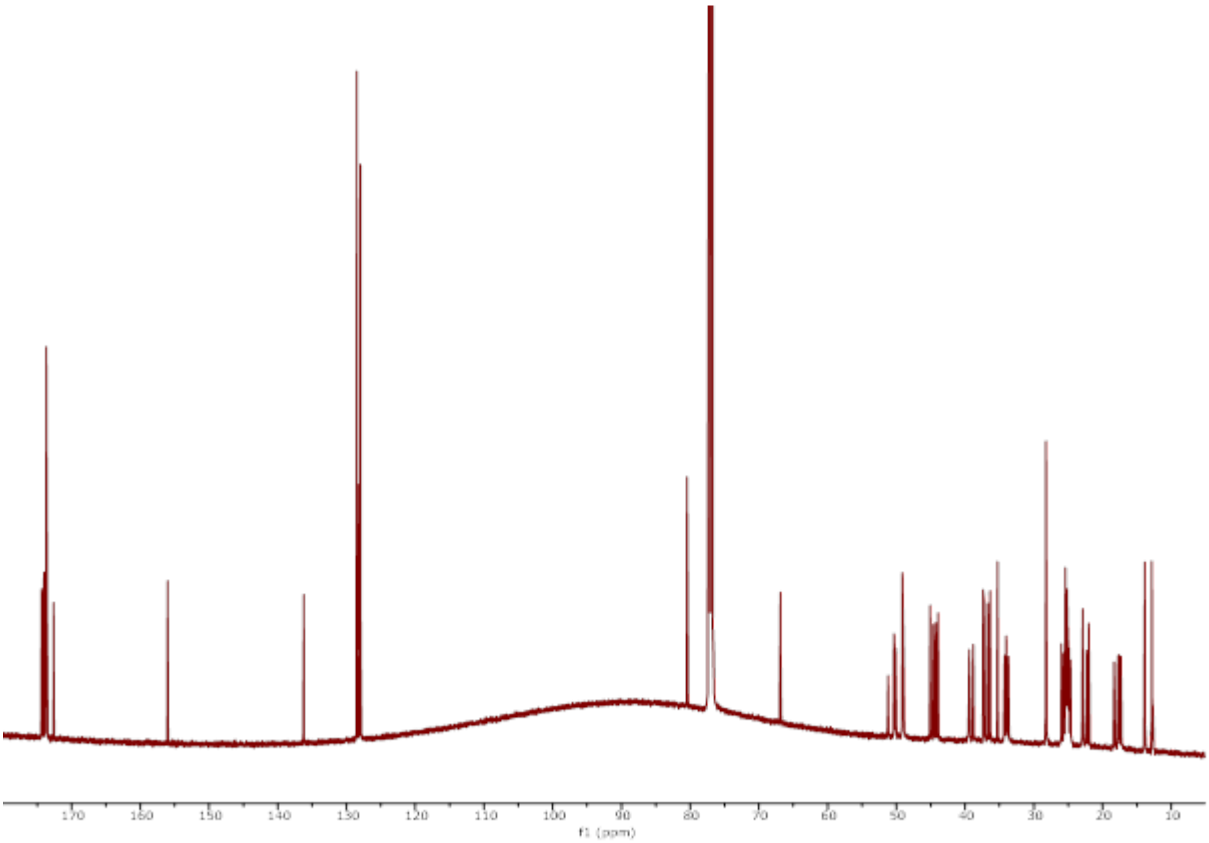

COSY (upper); **2D-TOCSY** (lower)

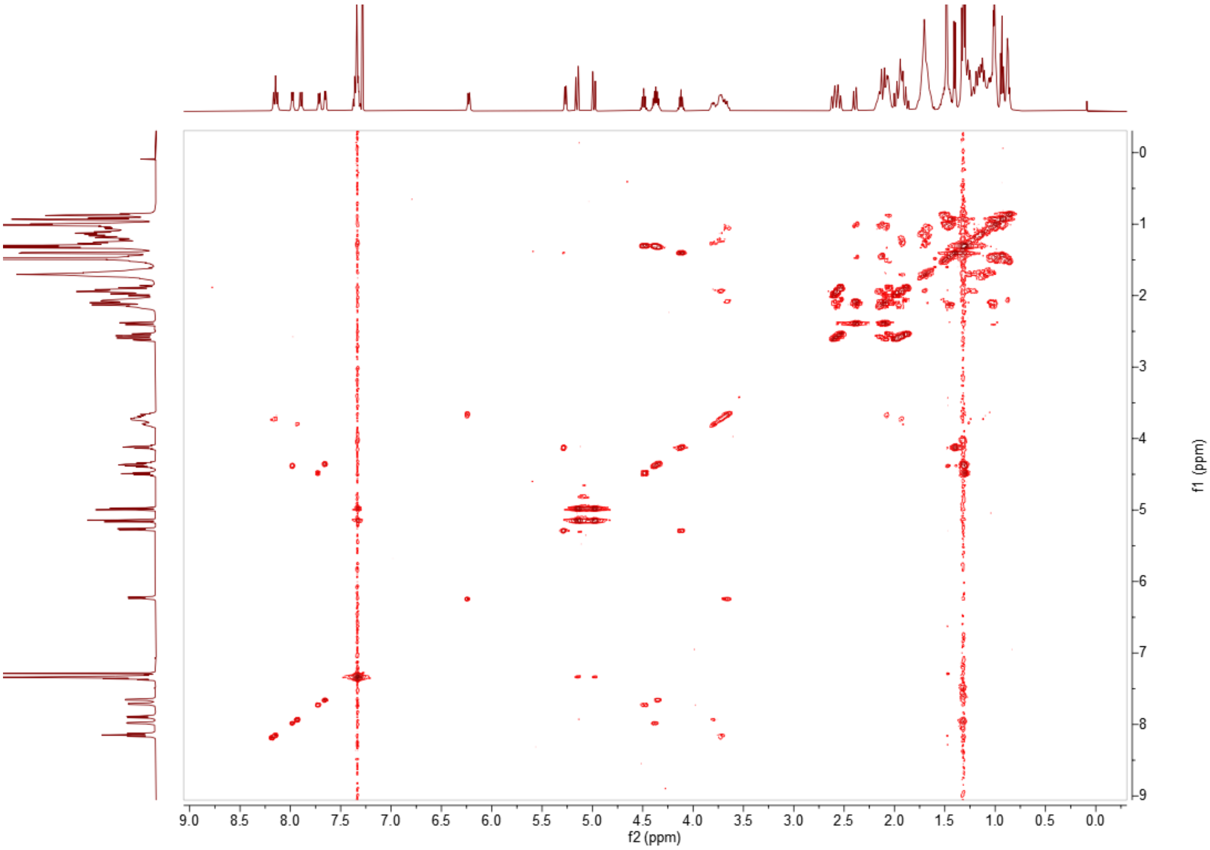

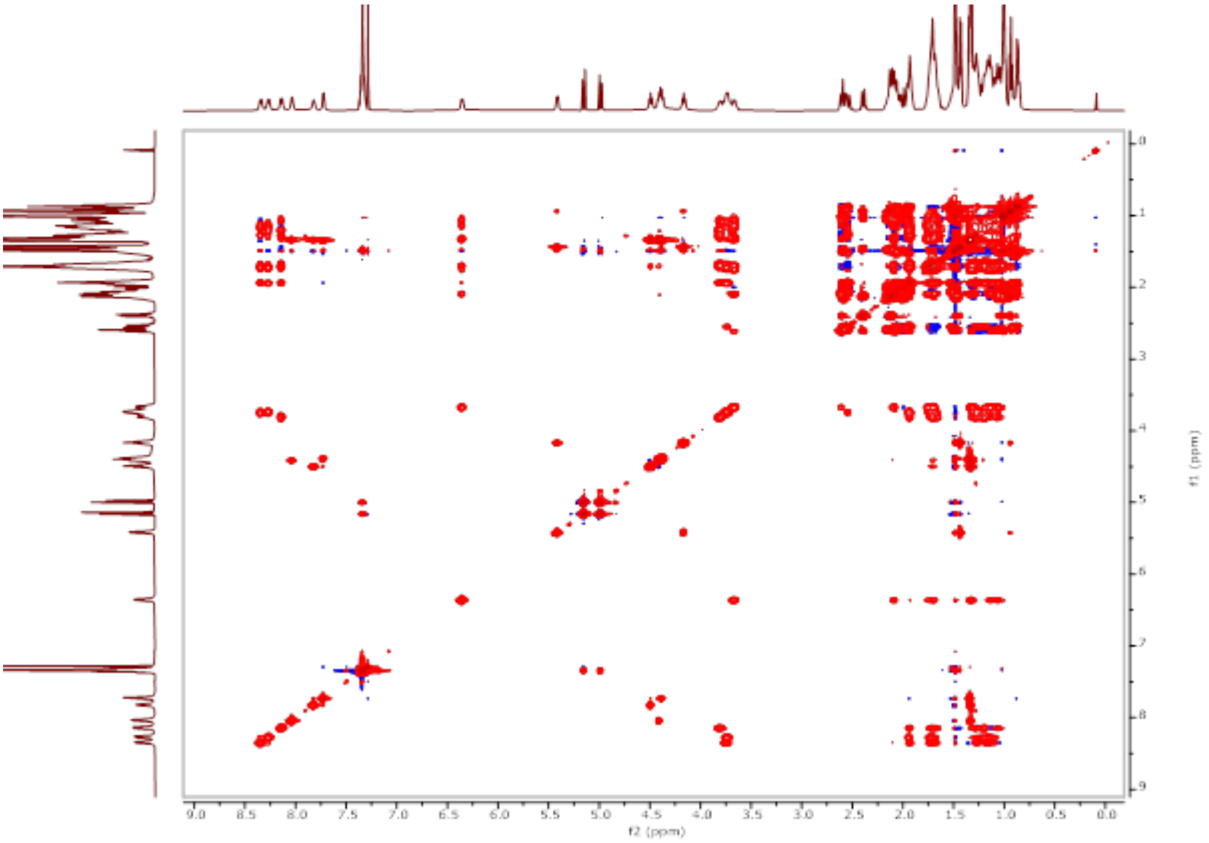

Pure-shift HSQC (upper); **HSQC-TOCSY** (lower)

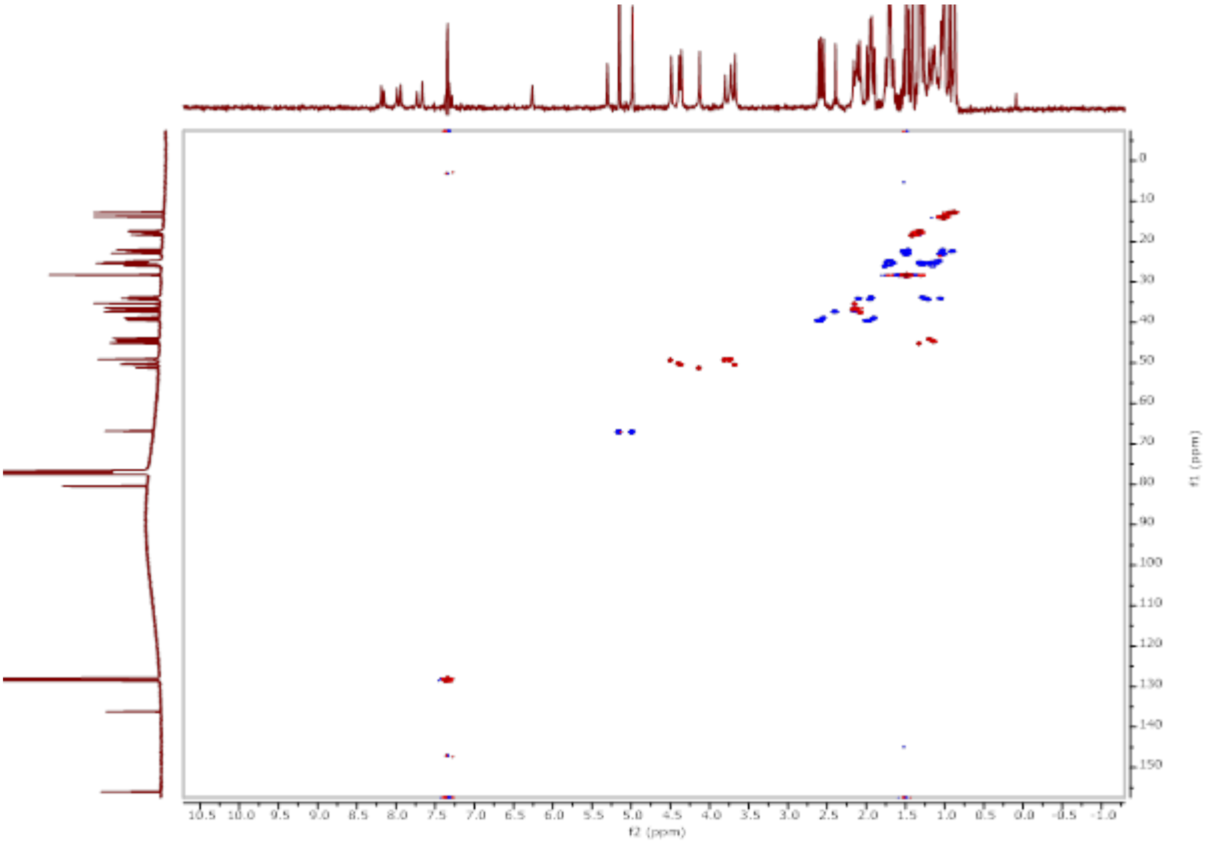

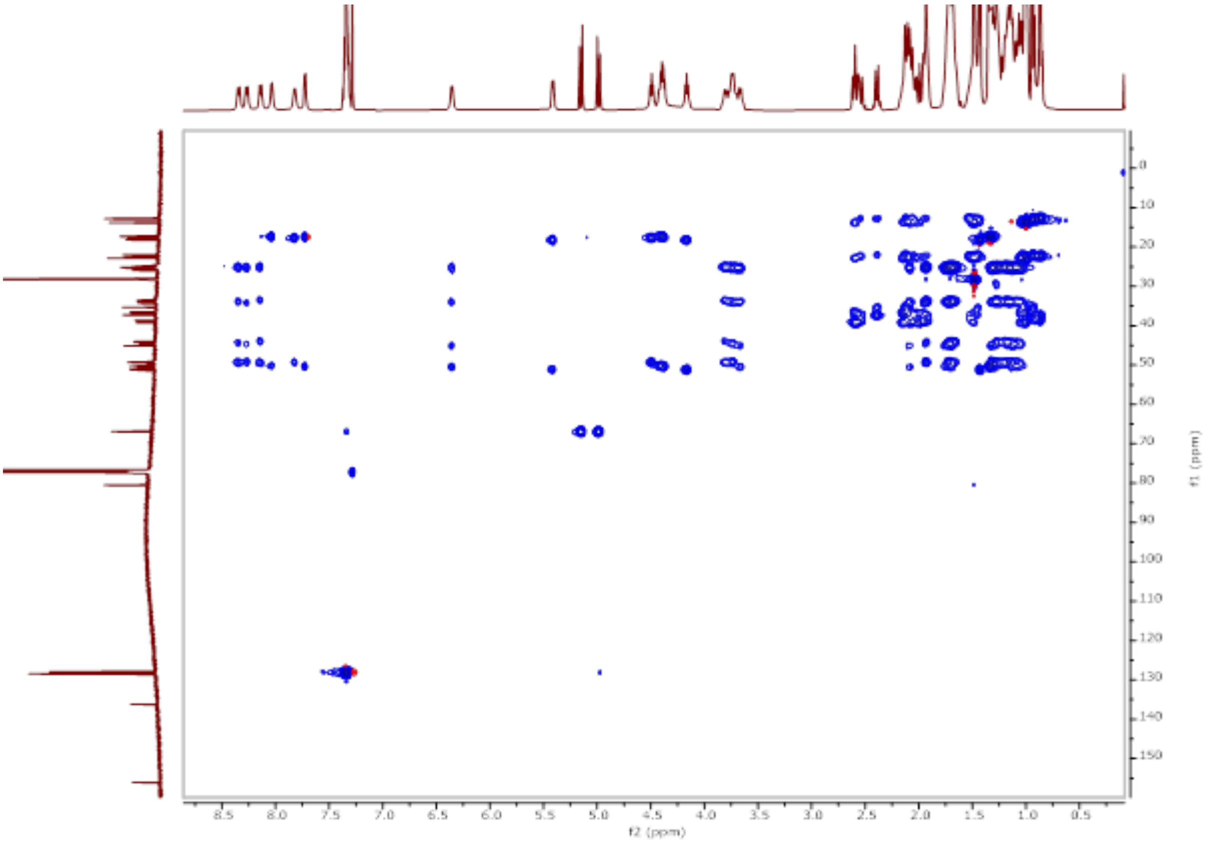

HMBC

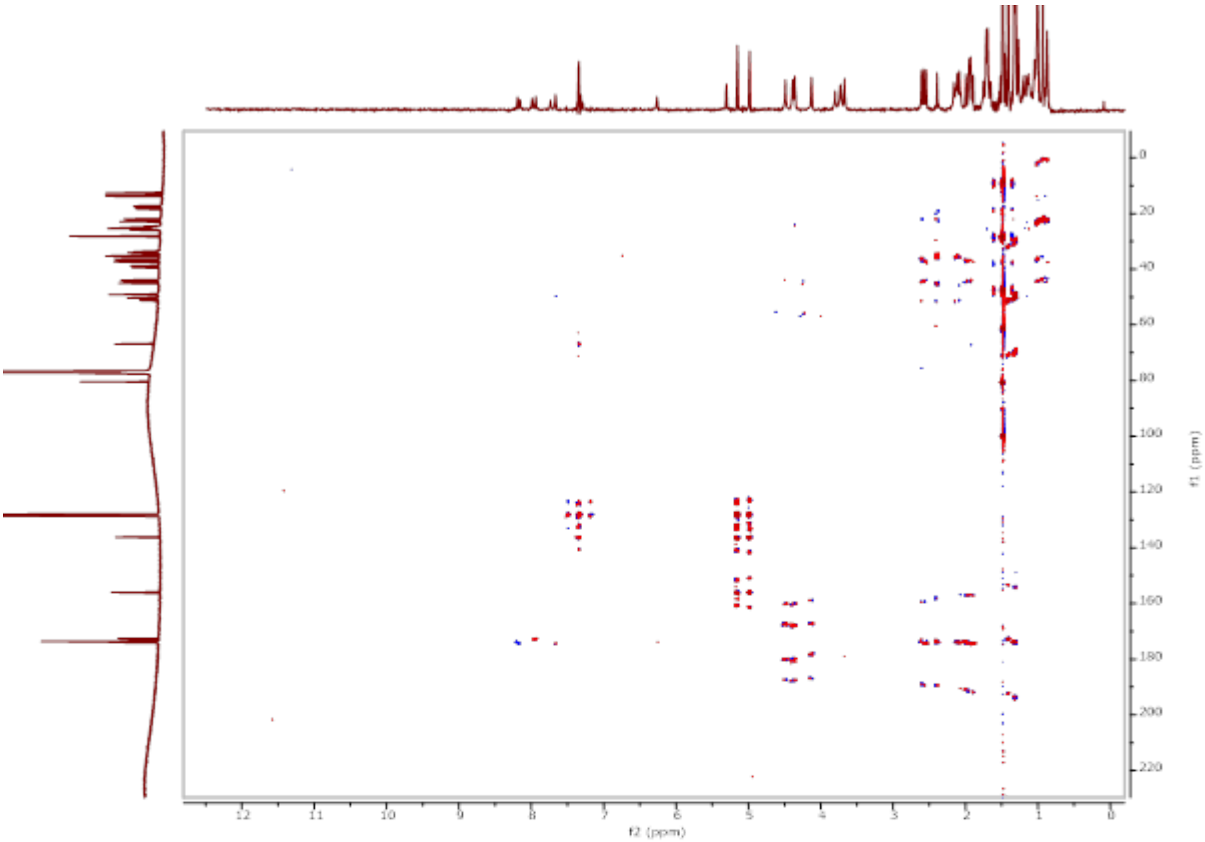

2D-NOESY Scan (upper, 0.5 s mixing); **2D-ROESY Scan** (lower, 0.3 s mixing)

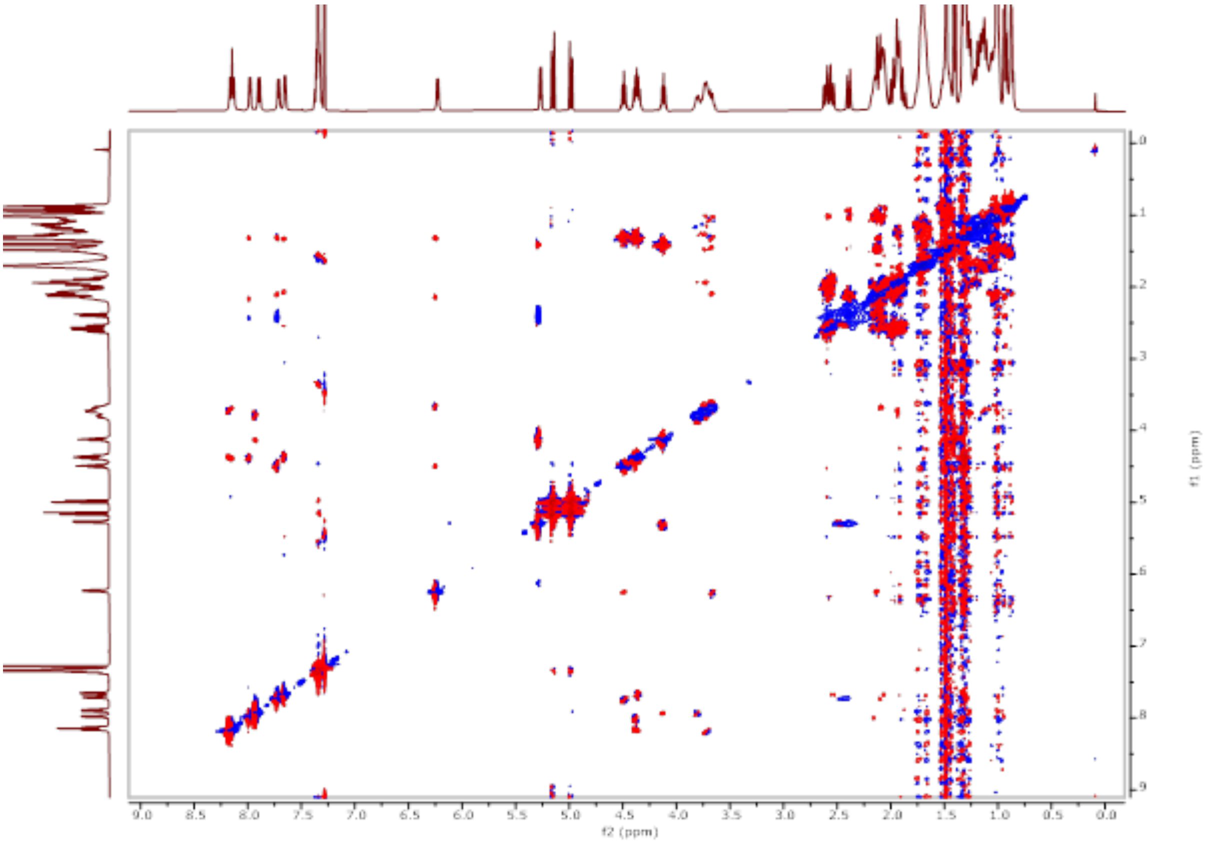

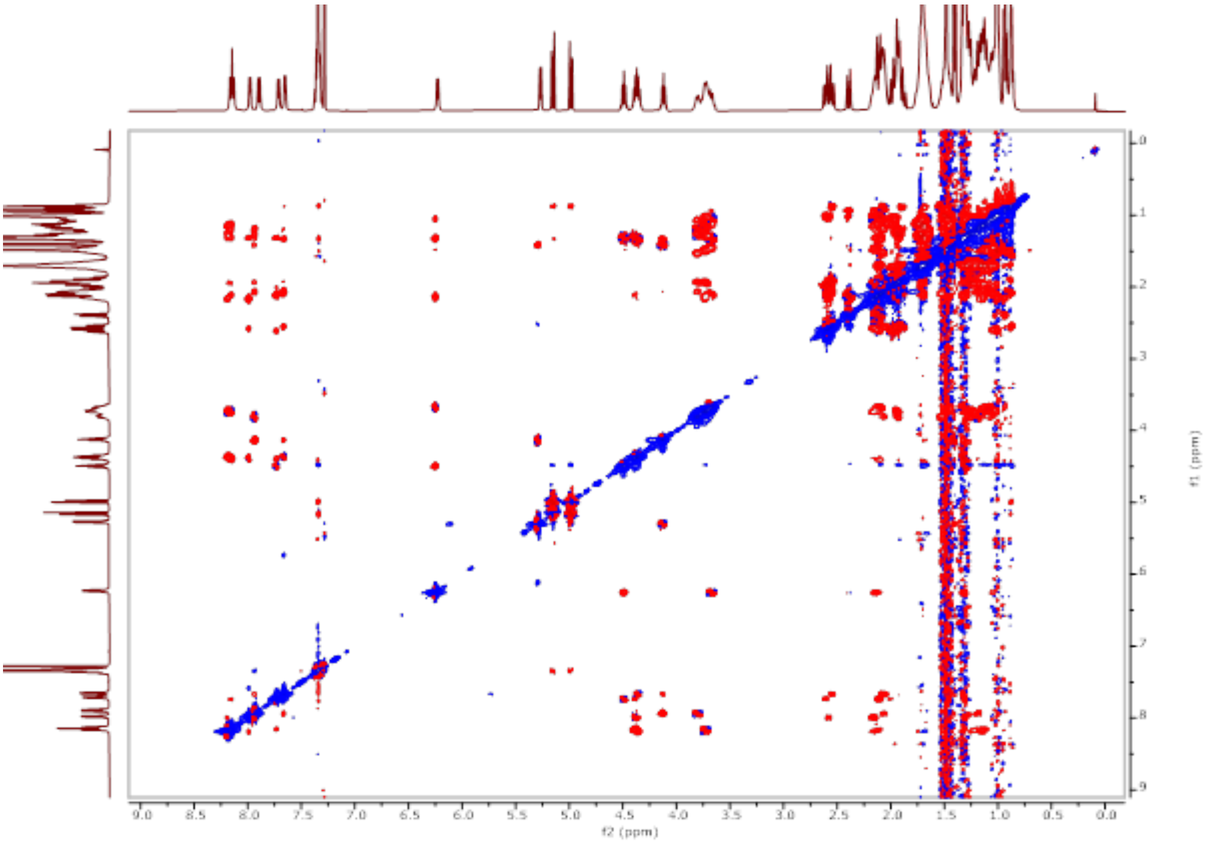

Scan of Mixing Time for ROESY (0.5-0.2 s from upper to lower)

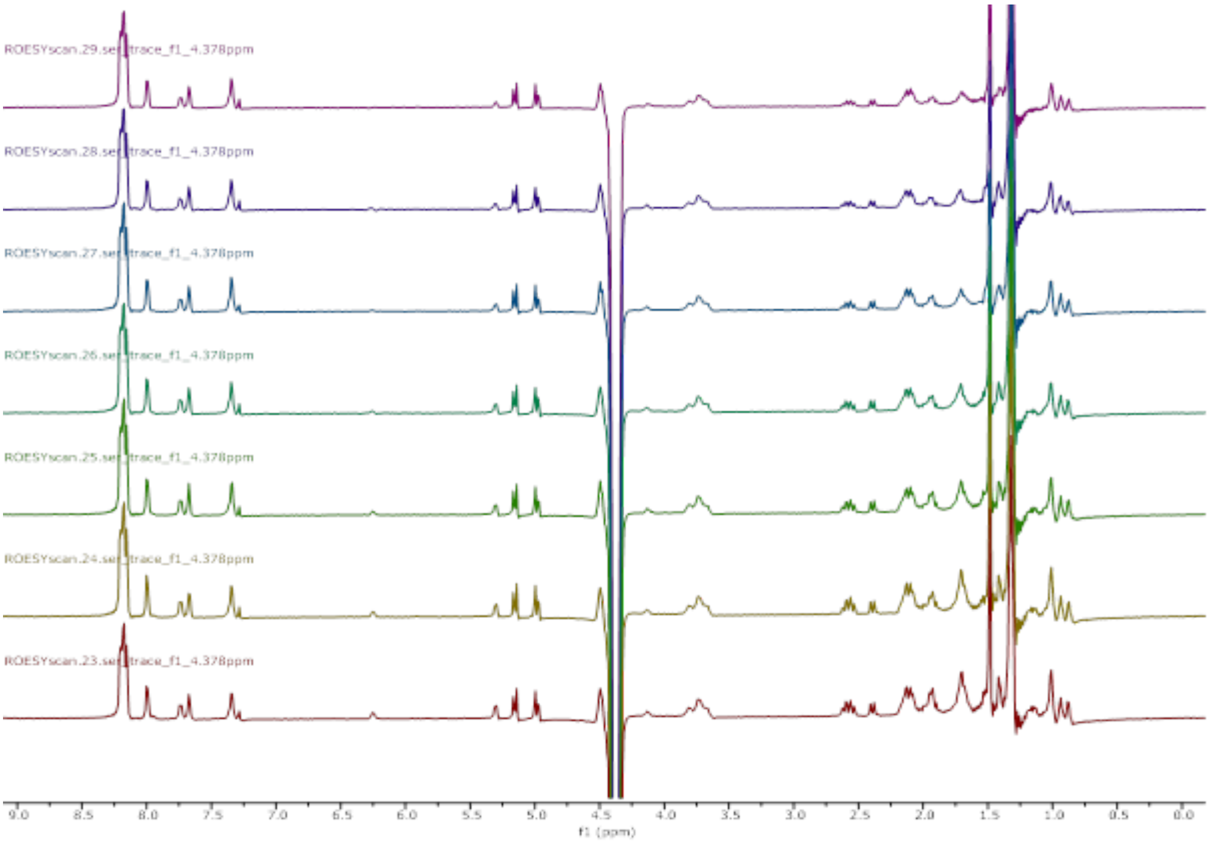

2D-ROESY (0.25 s mixing, 10 scans, acquired size: 1024 for both f1 & f2)

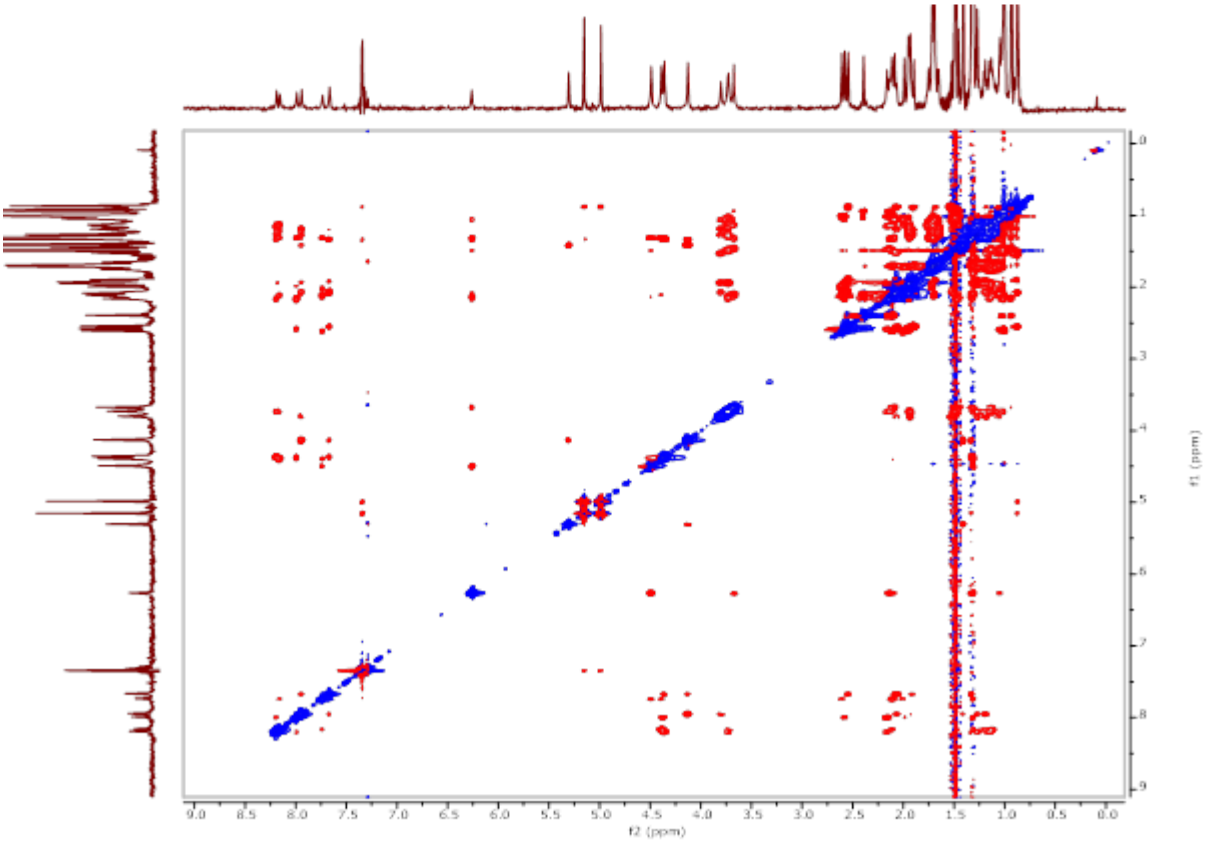

7.3 Additional Spectra ($CDCl_3$, 950 MHz)

^{1}H (up); **^{1}H pure-shift (tse-psyche)** (down)

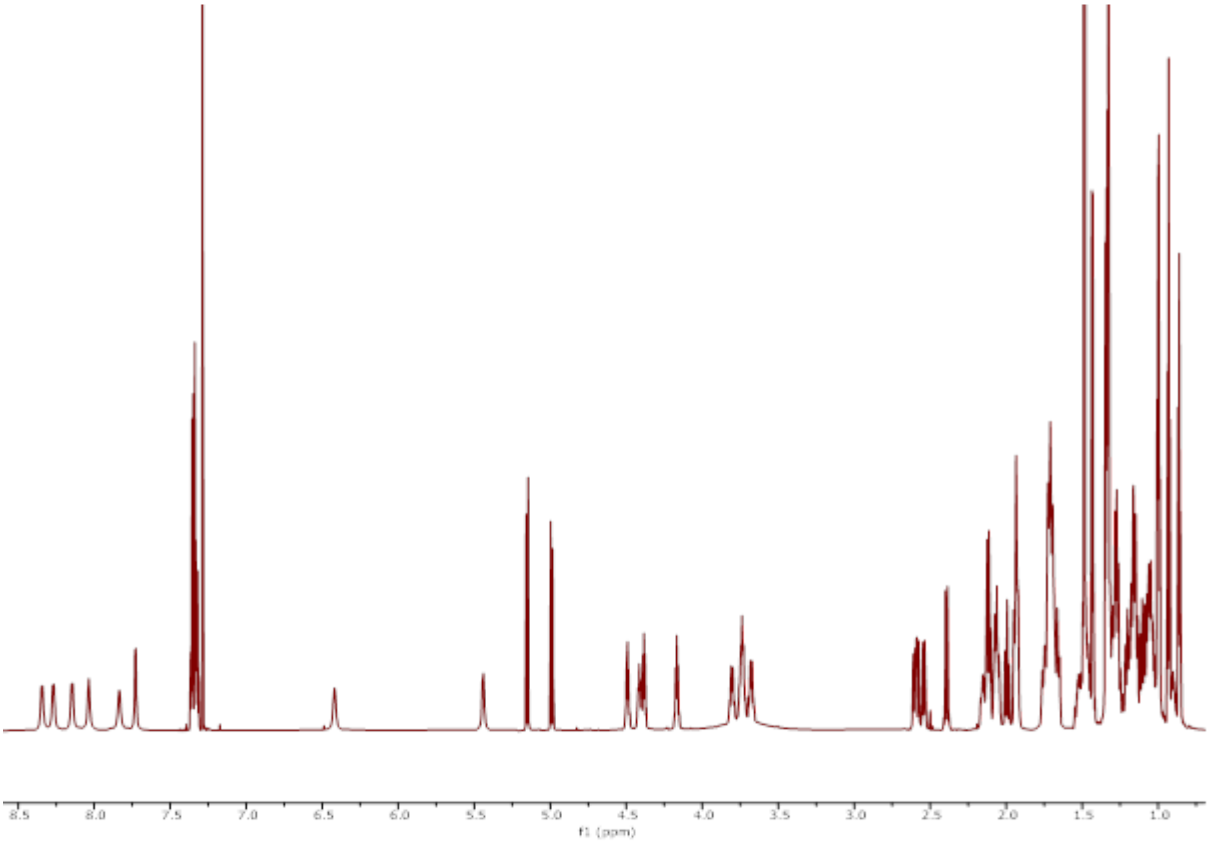

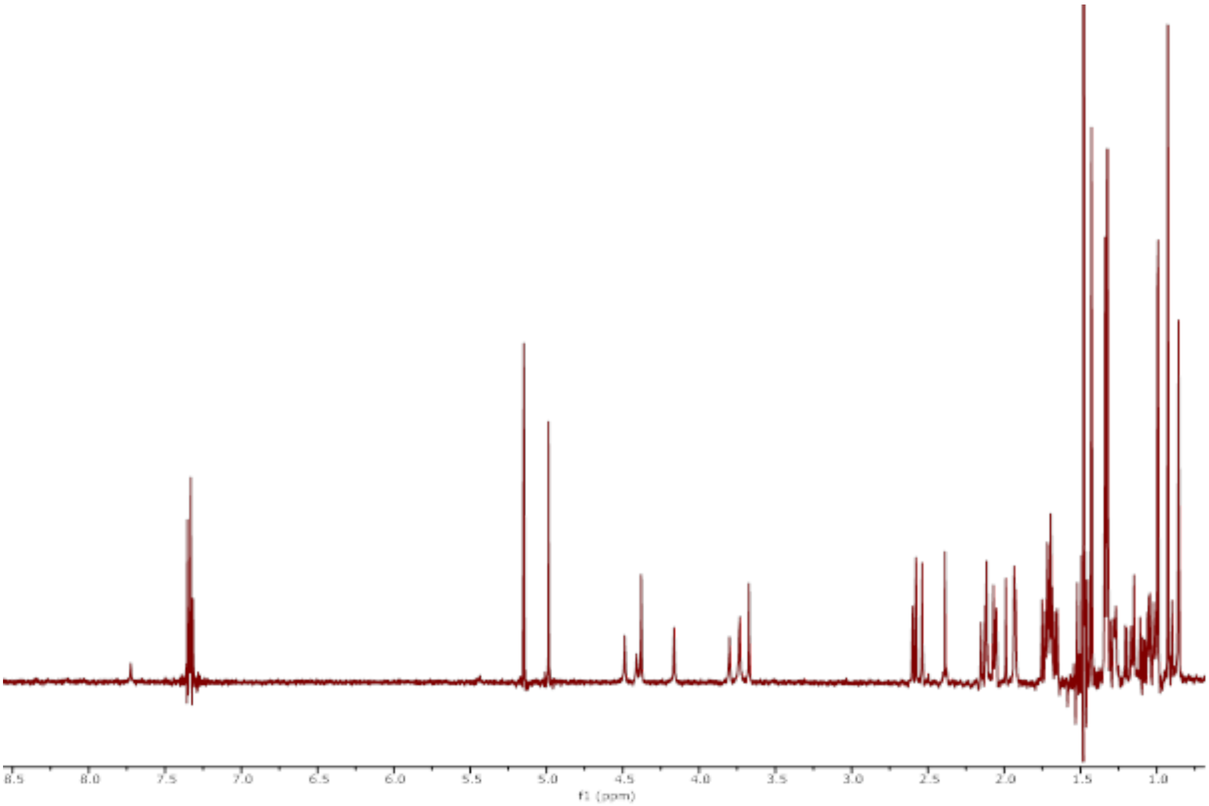

^{13}C

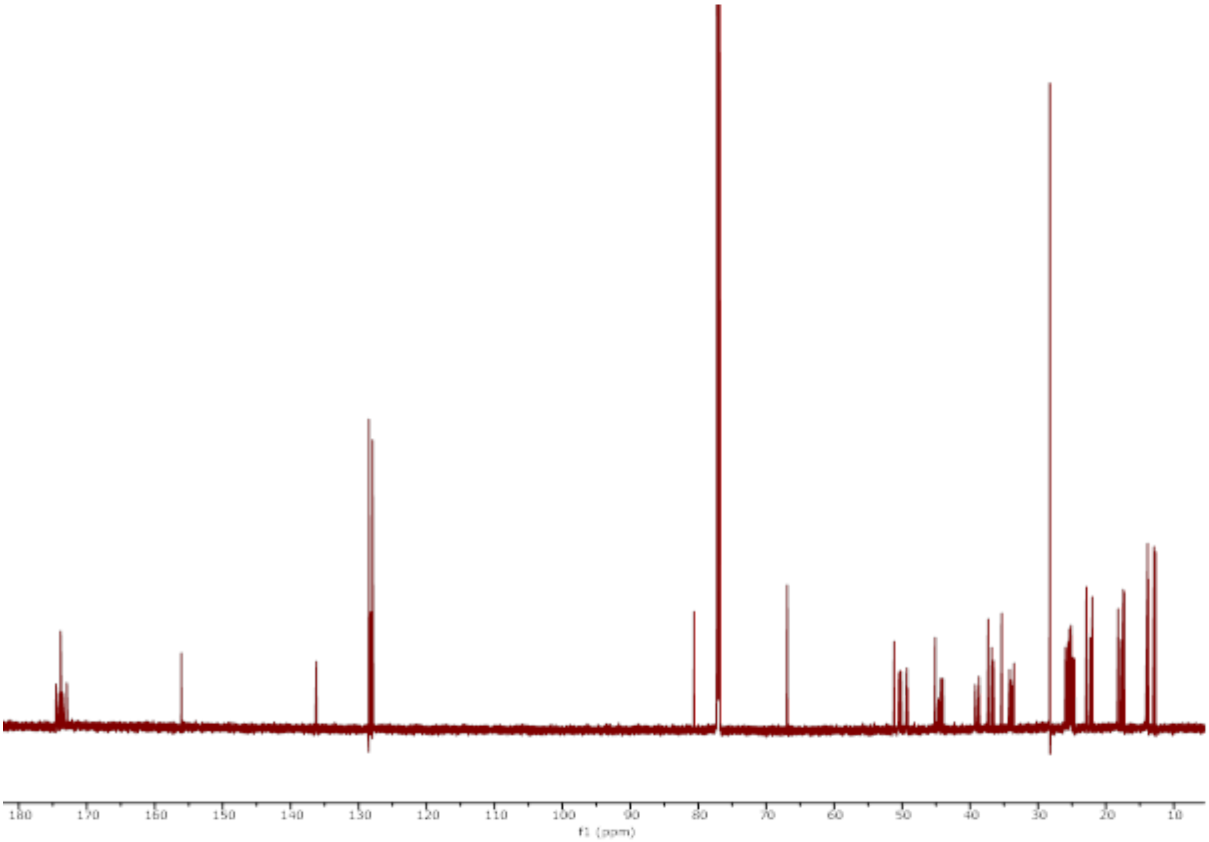

180
170
160
150
140
130
120
110
100
90
80
70
60
50
40
30
20
10
f1 (ppm)

TSE-PSYCHE-2D*J*

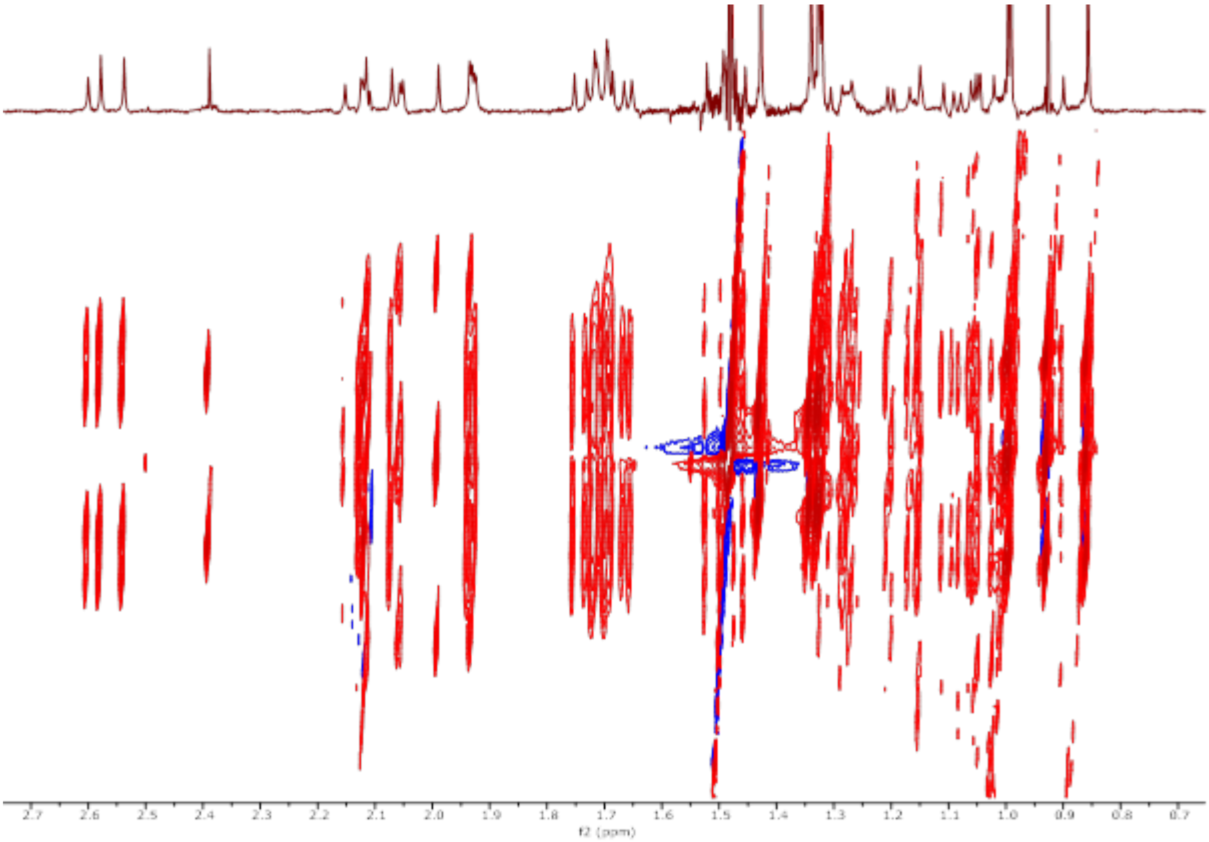

f1 pure-shift 2D-TOCSY (aliphatic region)

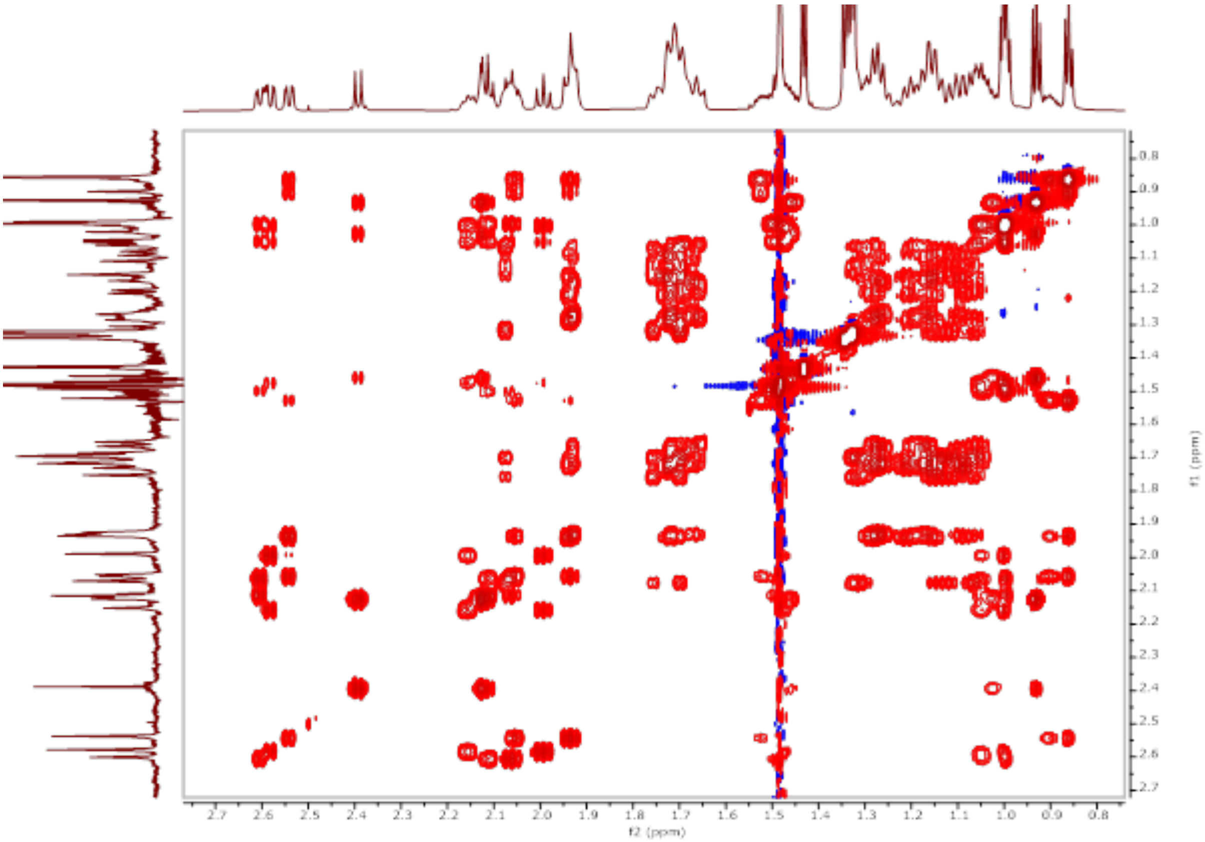

HMBC

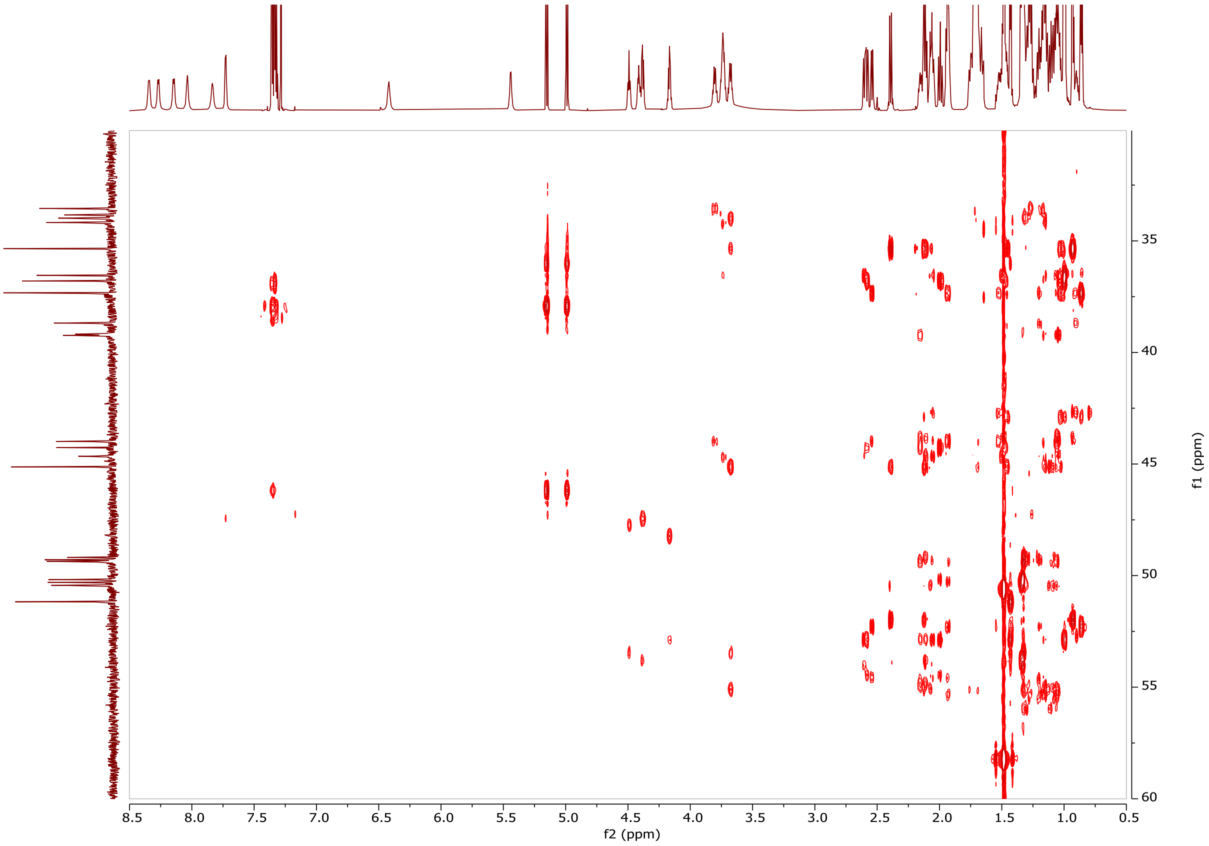

35
40
45
50
55
60
f1 (ppm)
8.5
8.0
7.5
7.0
6.5
6.0
5.5
5.0
4.5
4.0
3.5
3.0
2.5
2.0
1.5
1.0
0.5
f2 (ppm)

2D-ROESY (0.2 s mixing, 8 scans, acquired size: 1024 for f2 & 246 for f1)

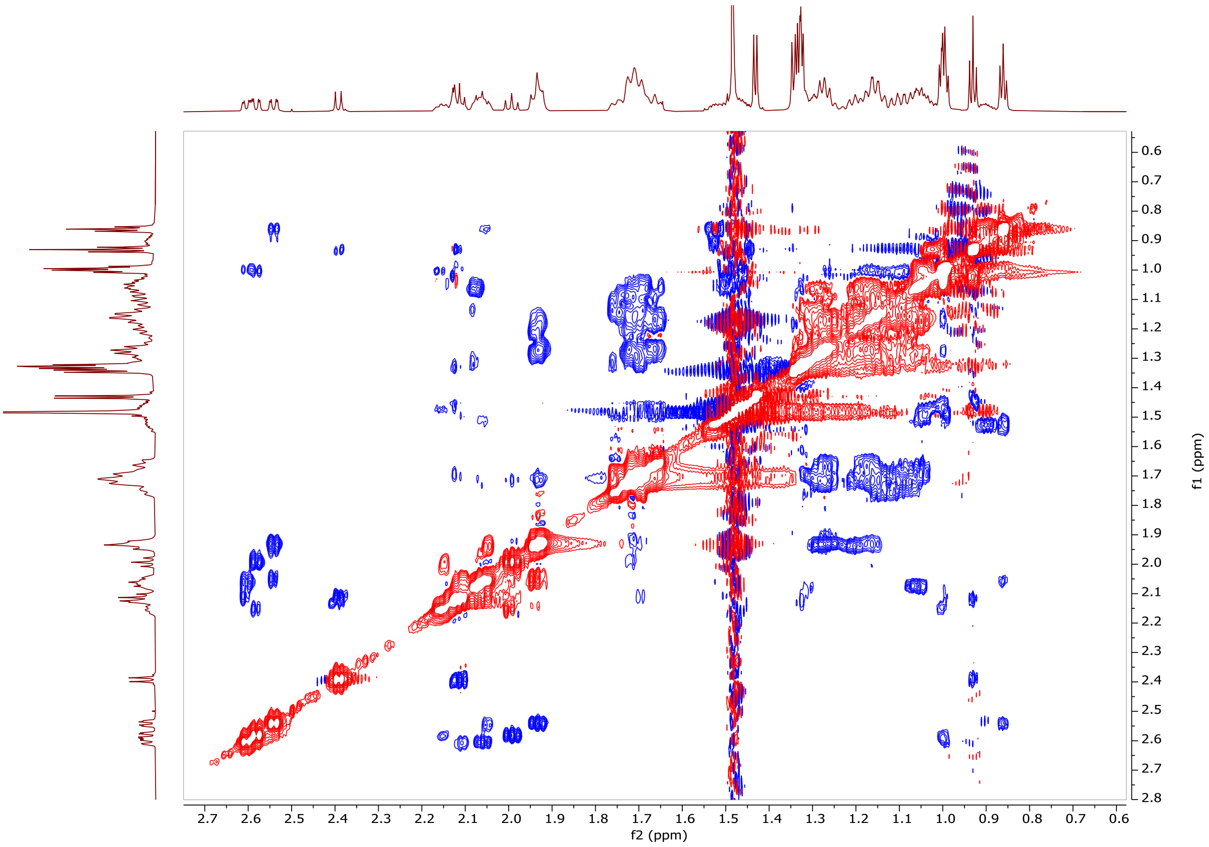

VT (N*H* temperature coefficients in $CDCl_3$, 25-50°C from upper to lower)

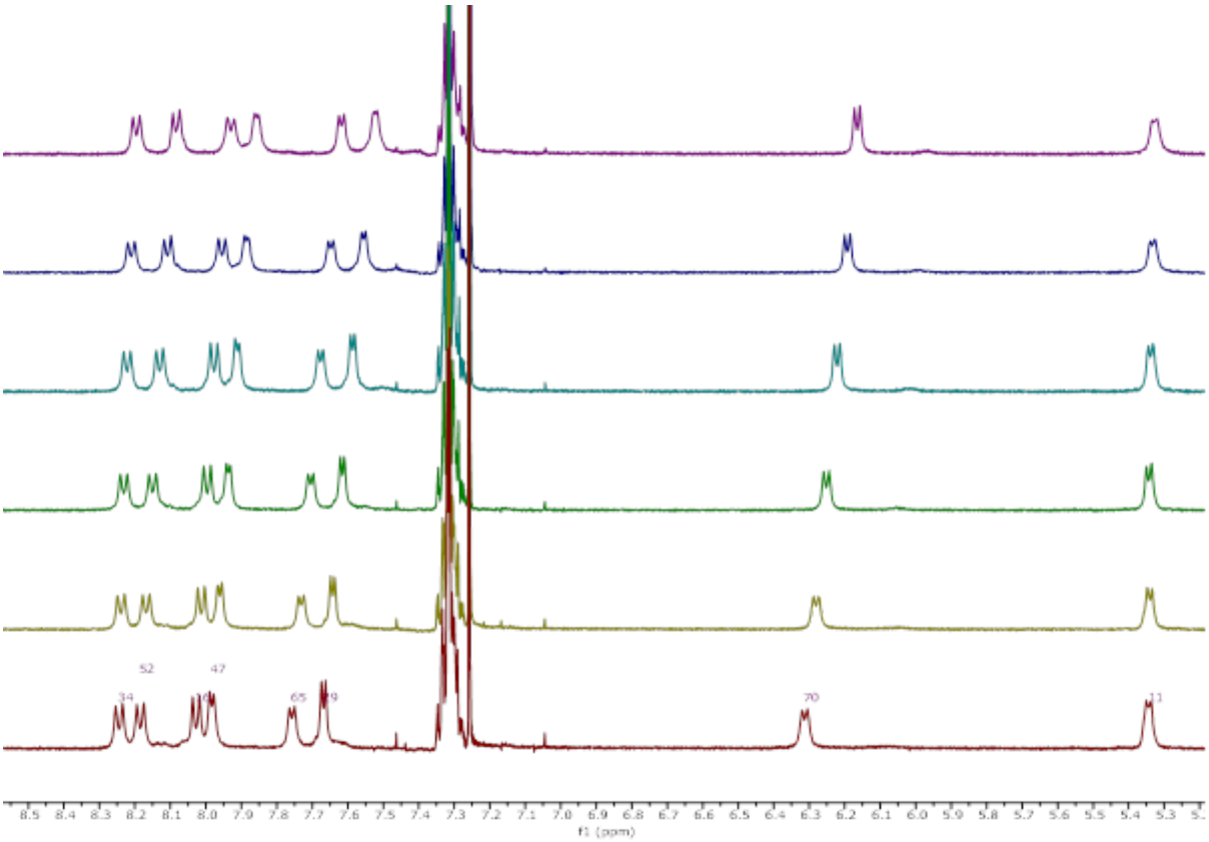

Spin Simulation of signal for Proton 72 from TOCSY ($^{3}J_{H71H72}$ = 10.3 Hz)

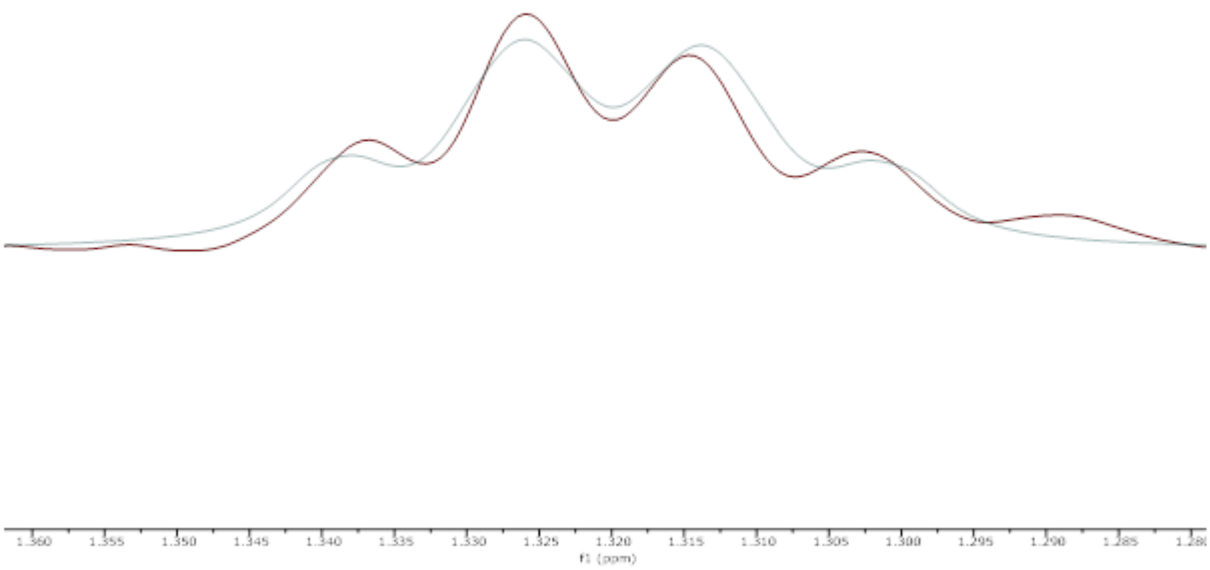

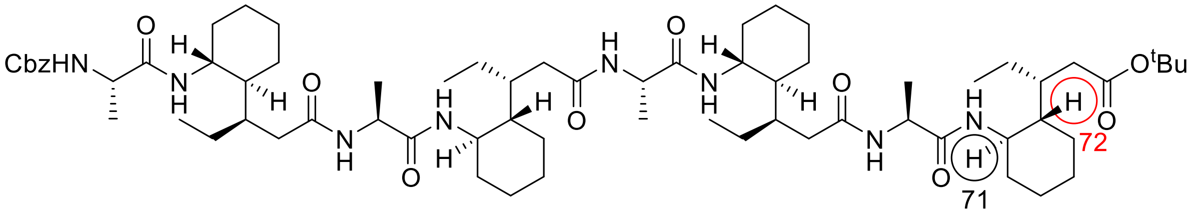

7.1 General Protocol of Computational Modelling and NMR Calculations

1. Software Environment

Unrestrained Monte-Carlo Multiple Minimisations (MCMM) for conformational search and sampling of foldamer and redundant conformer elimination were performed using academic licenced *MacroModel* module in *Maestro* software package powered by *Schrodinger*.

Quantum Mechanics / Density Functional Theory (QM / DFT) for geometry optimisations, frequency analysis, GIAO NMR calculations were performed using *Gaussian* 16 program. Single point energy (SPE) calculations with specific functional and basis set were applied using ORCA 4.2 version.

The in-house script *auto-ENRICH* (https://github.com/wg12385/autoenrich_public) was used for batch data processing of DFT and NMR calculations following general instruction (https://wiki-ae.readthedocs.io/en/latest/index.html).

2. Hardware Environment

All MCMM jobs were performed on Linux operating system of Grendel high-performance computing (HPC) in the school of chemistry, University of Bristol. All MD, QM/DFT and NMR calculation jobs were performed on Linux operating systems of either BlueCrystal Phase 3 (BC3) or BluePebble HPC in advanced computing research centre (ACRC) at the University of Bristol.

3. Procedure of MCMM-DFT Modeling in Solution

For unrestrained MCMM conformational sampling, both Merck Molecular Force Field static version (MMFFs) and Optimised Potentials for Liquid Simulations 2005 version (OPLS_2005) were used to evaluate relative energies of generated conformers based on Mixed torsional/Low-mode sampling method under 50000-100000 steps. The generalised Born/surface area (GB/SA) continuum solvation model of chloroform was applied to match the NMR solvent for study. The method of Truncated Newton Conjugate Gradient (TNCG) with 500 iterations under 0.05 convergence threshold was used for fine minimisations. All conformers within the threshold of 21-42 kJ/mol (5-10 kcal/mol) over instantaneous global minimum were stored. Maximum atom deviation (MAD cut-off = 0.5 Å) was used for initial elimination of redundant conformers. Further clustering based on heavy-atom RMSD (1-2 Å) were performed to degenerate the number of diverse conformers for batch DFT optimisations in HPC feasibly.

For DFT geometry optimisations and single point energy (SPE) calculations of foldamer, conformers sampled from MCMM were submitted to *Gaussian* software in batch jobs. The geometry optimisation was performed under ωb97xD functionals with 6-31G** basis set. Integral Equation Formalism Polarizable Continuum Model (IEFPCM=$CHCl_3$) was applied as the implicit solvation to match NMR experiment. The default Berny algorithm of optimisation with tight convergence criteria (SCF=tight) and ultrafine integration grid (Int=Ultrafine) were used for local minimisations of each conformer. The calculation of frequency (Freq) was performed following each geometry optimisation. For all successful DFT calculations, redundant conformers were eliminated based on both geometry and energy similarities after alignment (0.1 Å cut-off for MAD and 0.1 kcal/mol cut-off for sum of electronic and thermal free energies).

Based on geometry optimised conformers (low-energy ensembles < 20 kJ/mol under ωb97xD/6-31G**), further single-point energy calculations were applied under better DFT level using *ORCA* software. The ωB97M-D3(BJ) functional with def2-TZVP basis set were chosen to calculate single-point electronic energy. The conductor-like polarisable continuum model (CPCM) was specified to account for corresponding solvent effects. The RI-J approximation (def2/J RIJCOX) was used to speed up calculations. For each conformer of foldamer, Gibbs free energy ($\Delta \boldsymbol{G}_{\boldsymbol{f}}^{\circ}$) is calculated by:

$$\mathbf{G_f^{\circ}} = \mathbf{E_{SPE}}(\omega\text{B97M-D3(BJ)/def2-TZVP}) + \mathbf{E_{FREQ}}(\omega\text{B97XD/6-31G**})$$

Relative Gibbs free energy ($\Delta \boldsymbol{G}_{\boldsymbol{f}}^{\circ}$) at standard ambient temperature and pressure (T = 298.15 K, p = 1 atm) was used for population determination based on Boltzmann distribution. For example, one molecule was sampled with $\boldsymbol{n}$ conformers from batch DFT calculations and each optimised conformer $\boldsymbol{i}$ had $\Delta \boldsymbol{G}_{\boldsymbol{f}}^{\circ}$ over the global minimum. Population of each conformer ($\boldsymbol{P_i}$) among ensembles would be:

$$P_i = \frac{e^{\frac{\Delta G_f^{\circ}}{RT}}}{\sum_{i=1}^{n} e^{\frac{\Delta G_f^{\circ}}{RT}}}$$

4. Procedure of Theoretical NMR Calculations from Modeling

Each calculated NOE (ROE) distance was extracted from the ensembles under Boltzmann population average. Based on physical principles of NMR, the r^{-6} scaling was applied necessarily for later comparison with experimental NOE (ROE) restraint. This was given by:

$$\mathrm{r}_{H-H,calc} = \left(\sum_{i=1}^{n} \left(r_{H-H,i}\right)^{-6} * p_i\right)^{-1/6}$$

Where $\mathbf{r}_{\boldsymbol{H-H,calc}}$ is calculated NOE (ROE) distance of conformational ensembles, in which interproton distance of each conformer, $\boldsymbol{r}_{\boldsymbol{H-H,i}}$, was scaled by r^{-6} and population ($\boldsymbol{p}_{\boldsymbol{i}}$) successively. Sum of calculated NOEs (ROEs) may also be adopted, if stated, for comparison with the experiment where corresponding NOE (ROE) cross-peaks (> 1) are integrated together due to overlap. Further details of analysis were described in our previous publications.[4,5]

For DFT calculation of nuclear spin-spin coupling constants, Gauge-independent atomic orbitals (GIAO) method with *ω*B97XD functional and 6-311G** basis set was used for each low-energy conformer (< 15 kJ/mol) following batch DFT optimisations. The two-step spin-spin coupling calculation (Mixed) was applied. The Boltzmann averaged spin-spin coupling constant (${}^{\boldsymbol{n}}_{\boldsymbol{cal}}\boldsymbol{J}_{\boldsymbol{XH}}$) was calculated from DFT-derived absolute coupling constant (${}^{\boldsymbol{n}}_{\boldsymbol{cal}}\boldsymbol{J}^{\boldsymbol{i}}{}_{\boldsymbol{XH}}$) within each conformer $\boldsymbol{i}$ and population $\boldsymbol{p}_{\boldsymbol{i}}$:

$${}^{n}_{cal}J_{XH} = \sum_{i=1}^{n} {}^{n}_{cal}J^{i}{}_{XH}\, p_i$$

Further detailed description of analysis used here can be found from our previous works.[4,6,7]

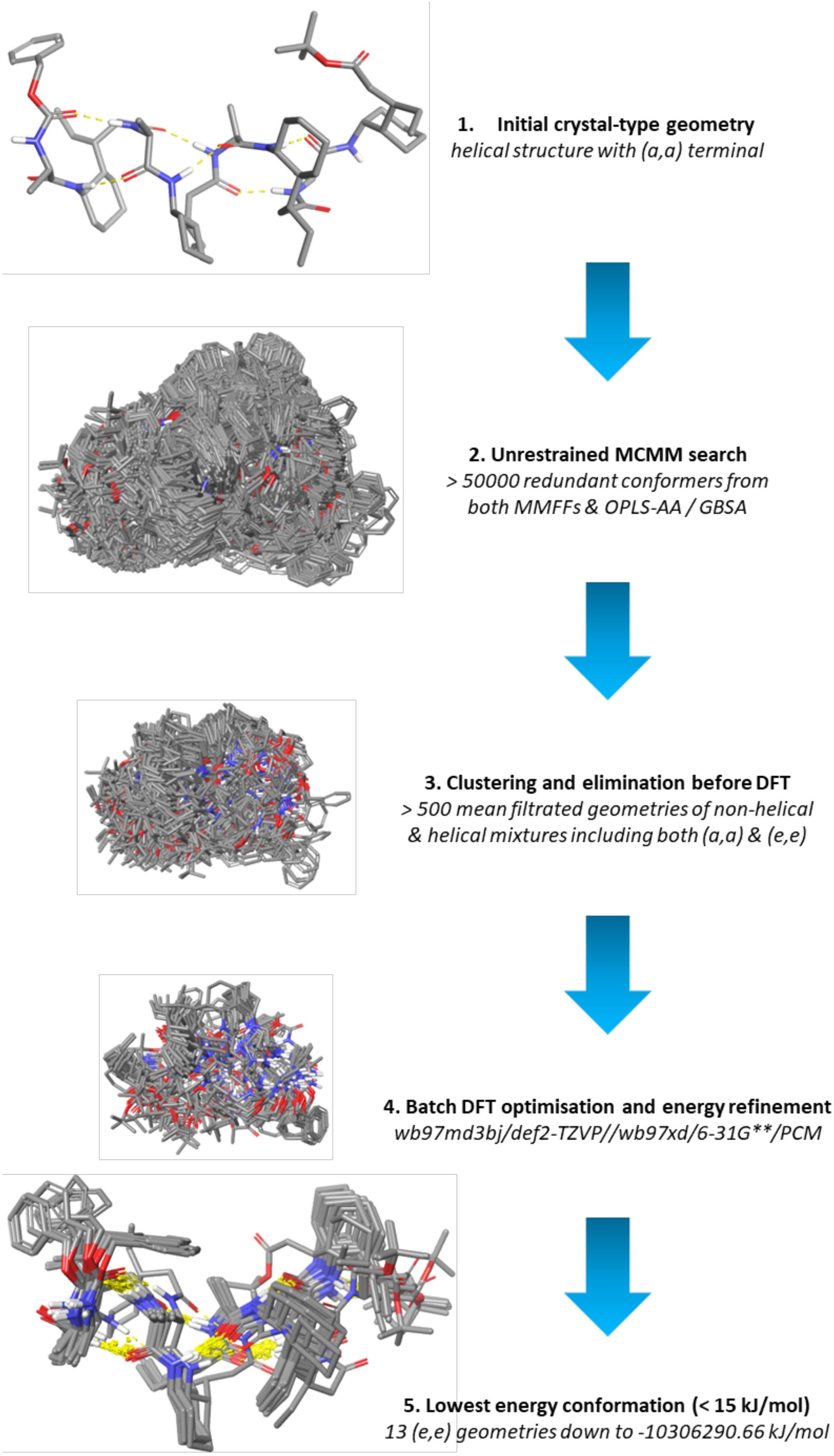

Figure SI19. The workflow of unrestrained MCMM-DFT for global energy minimisations

MCMM-DFT Conformer Numbers	Single Point Energies (ωb97md3bj/def2-TZVP) [h]	Thermal Corrections (ωb97xd/6-31G**/IEFPCM) [h]	Final Gibbs Free Energies [h]	Final Gibbs Free Energies [kJ/mol]	Relative Gibbs free energies [kJ/mol]
29	-3927.117758	1.659348	-3925.45841	-10306290.66	0
9	-3927.119025	1.660791	-3925.458234	-10306290.20	0.46
22	-3927.115868	1.661033	-3925.454835	-10306281.28	9.39
60	-3927.115950	1.661278	-3925.454672	-10306280.85	9.82
5	-3927.116140	1.661828	-3925.454312	-10306279.90	10.76
272	-3927.114077	1.659825	-3925.454252	-10306279.75	10.92
32	-3927.114776	1.660929	-3925.453847	-10306278.68	11.98
125	-3927.116202	1.662377	-3925.453825	-10306278.62	12.04
113	-3927.114261	1.660556	-3925.453705	-10306278.31	12.35
305	-3927.114225	1.661122	-3925.453103	-10306276.73	13.93
210	-3927.117948	1.664891	-3925.453057	-10306276.61	14.06
41	-3927.115032	1.662027	-3925.453005	-10306276.47	14.19
289	-3927.115925	1.663054	-3925.452871	-10306276.12	14.54
…					
(a,a)-crystal	-3927.110113	1.662800	-3925.447313	-10306260.51	30.15

Table SI4. The *ab initio* energies of stable conformers afforded from MCMM systematic search followed by batch DFT optimisation.

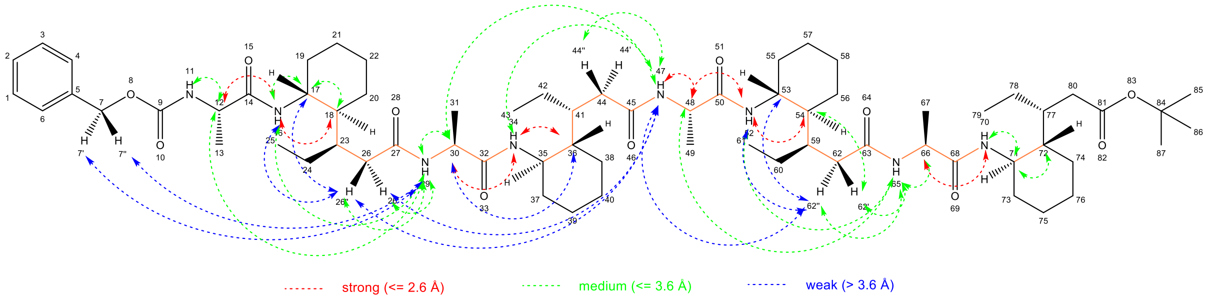

a.

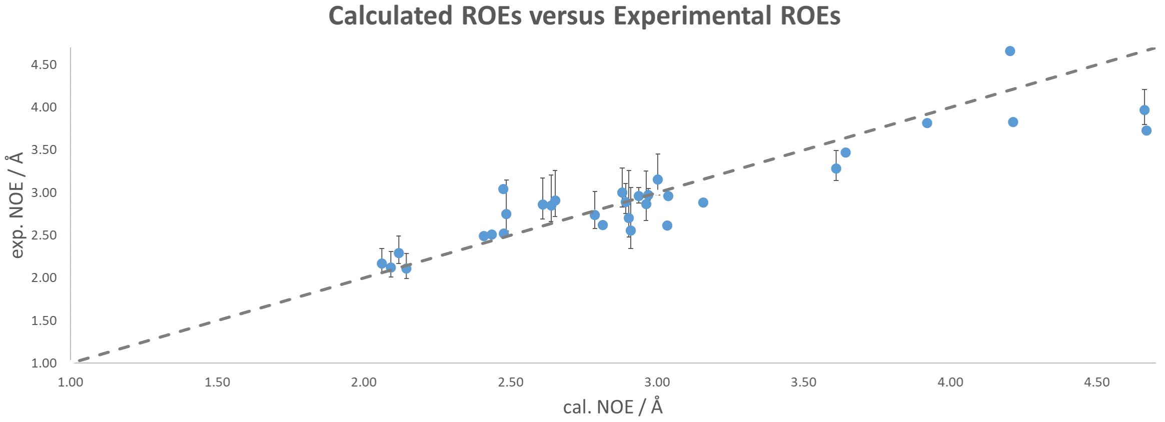

b.

Labels of proton pair (PDB)	124-143	124-120	124-126	148-167	148-144	148-150
Labels of heavy atoms	34-47	34-30	34-36	52-65	52-48	52-54
**cal. ROE**	**2.97**	**2.09**	**2.44**	**3.16**	**2.15**	**2.48**
**exp. ROE (average)**	**2.97**	**2.12**	**2.51**	**2.89**	**2.11**	**2.52**
exp. ROE (upper)	3.05	2.31	2.51	2.93	2.29	2.52
exp. ROE (lower)	2.91	2.01	2.51	2.85	2.00	2.52

143-144	143-141	143-165	143-120	100-119	100-96	100-101	100-102
47-48	47-44''	47-62’’	47-30	16-29	16--12	16-17	16-18
**2.91**	**2.64**	**6.46**	**2.48**	**2.94**	**2.06**	**2.96**	**2.41**
**2.55**	**2.85**	**3.74**	**3.04**	**2.96**	**2.17**	**2.87**	**2.49**
3.06	3.20	3.74	3.04	3.06	2.34	3.25	2.49
2.35	2.66	3.74	3.04	2.88	2.06	2.67	2.49

100-118	167-168	167-144	167-166	167-165	119-117	119-118	119-120
16-26''	65-66	65-48	65-62'	65-62''	29-26'	29-26''	29-30
**4.66**	**2.90**	**2.49**	**3.64**	**2.61**	**2.65**	**3.61**	**2.89**
**3.97**	**2.70**	**2.75**	**3.47**	**2.86**	**2.91**	**3.28**	**2.89**
4.21	3.26	3.15	3.47	3.17	3.26	3.49	3.10
3.80	2.48	2.55	3.47	2.69	2.72	3.14	2.75

119-93	119-94	119-96	172-168	172-173	95-96	120-126	101-102
29-7'	29-7''	29--12	70-66	70-71	11--12	30-36	17-18
**3.92**	**4.94**	**3.00**	**2.12**	**2.88**	**2.79**	**4.21**	**3.04**
**3.82**	**4.36**	**3.15**	**2.29**	**3.00**	**2.74**	**3.83**	**2.96**
3.82	4.36	3.45	2.49	3.29	3.01	3.83	2.96
3.82	4.36	2.97	2.17	2.83	2.58	3.83	2.96

173-174	166-150	117-143	165-148	165-149	118-143	118-101
71-72	62'-54	26'-47	62''-52	62''-53	26''-47	26''-17
**3.03**	**2.81**	**4.67**	**4.74**	**4.95**	**4.20**	**4.72**
**2.62**	**2.62**	**3.73**	**3.77**	**3.66**	**4.66**	**3.70**
2.62	2.62	3.73	3.77	3.66	4.66	3.70
2.62	2.62	3.73	3.77	3.66	4.66	3.70

Figure SI12. a. The fitting of ROEs between DFT calculations and experimental restraints (2D-ROESY, $CDCl_3$, 500 and 950 MHz). **SI2. b.** The list of each ROE restraint between labelled protons.

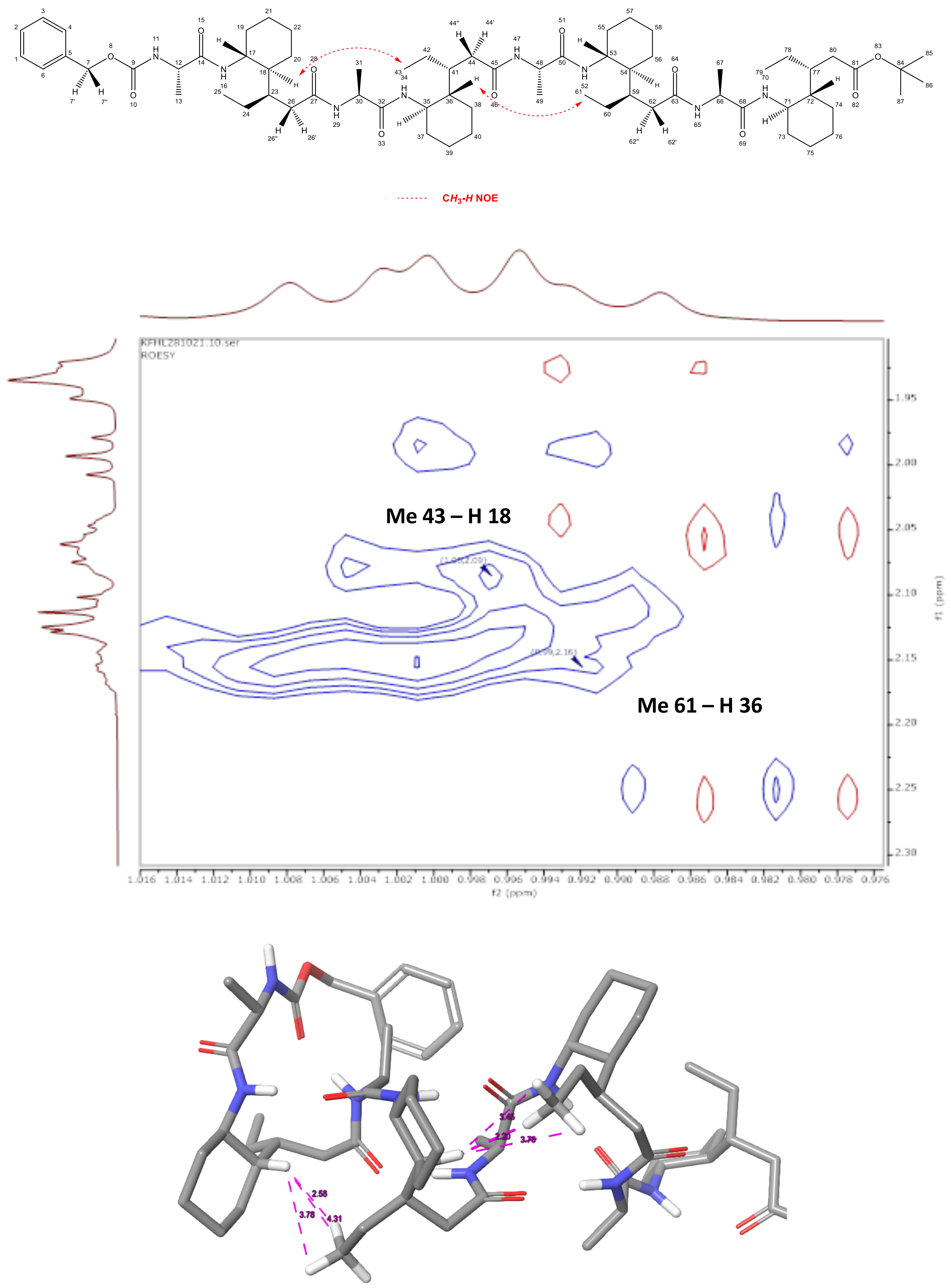

Figure SI13. Two observed ROE correlations between terminal methyl on ethyl sidechain (-CH2C***H₃***) and methine on cyclohexane (C***H***) across residue, probably due to apolar interactions. The geometry of global minimum was used for illustration ($CDCl_3$, 950MHz).

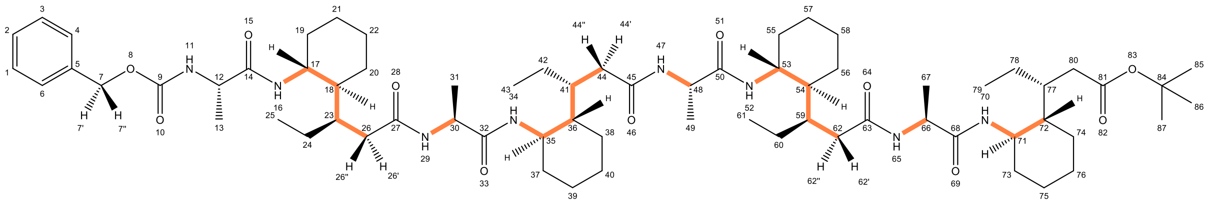

a.

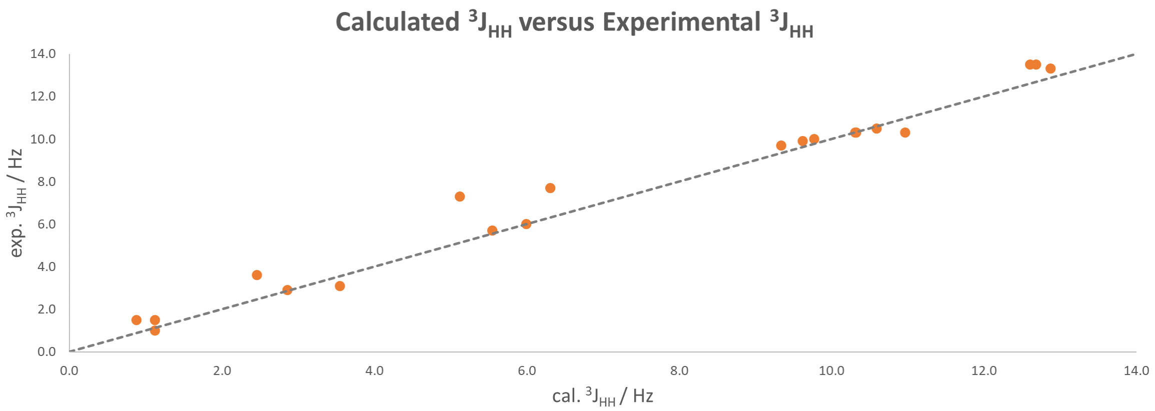

Labels of vicinal protons (PDB)	100-101	101-102	102-111	111-117	111-118
Labels of backbone atoms	16-17	17-18	18-23	23-26'	23-26''
**exp. 3JHH**	**9.7**	**10.3**	**1.0**	**3.1**	**13.3**
**cal. 3JHH**	**9.3**	**10.3**	**1.1**	**3.6**	**12.9**

119-120	124-125	125-126	126-135	135-141	135-142	143-144	148-149
29-30	34-35	35-36	36-41	41-44'	41-44''	47-48	52-53
**5.7**	**9.9**	**10.5**	**1.5**	**2.9**	**13.5**	**6.0**	**10.0**
**5.6**	**9.6**	**10.6**	**0.9**	**2.9**	**12.6**	**6.0**	**9.8**

149-150	150-159	159-165	159-166	167-168	172-173	173-174
53-54	54-59	59-62'	59-62''	65-66	70-71	71-72
**10.3**	**1.5**	**3.6**	**13.5**	**7.3**	**7.7**	**10.3**
**10.3**	**1.1**	**2.5**	**12.7**	**5.1**	**6.3**	**11.0**

b.

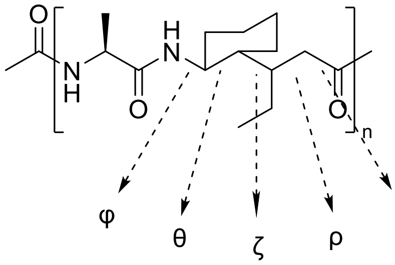

	φ			θ			ζ			ρ			ψ		
**delta residue starting from N terminal**	**delta1**	**delta2**	**delta3**	**delta1**	**delta2**	**delta3**	**delta1**	**delta2**	**delta3**	**delta1**	**delta2**	**delta3**	**delta1**	**delta2**	**delta3**
Conf 5	111.11	106.085	104.956	-54.538	-54.883	-58.261	143.015	144.985	150.426	-64.291	-63.329	-55.409	-63.132	-61.192	-61.423
Conf 9	115.706	115.283	101.163	-58.564	-55.929	-58.351	148.533	144.401	151.149	-61.005	-64.815	-56.11	-57.441	-59.48	-61.062
Conf 22	115.765	114.465	102.753	-58.447	-55.647	-57.254	148.506	144.208	150.071	-61.447	-65.109	-60.954	-57.371	-60.971	-56.577
Conf 29	115.715	113.28	100.156	-58.604	-55.334	-56.021	148.457	144.734	147.495	-61.267	-64.755	-60.058	-56.381	-59.827	-65.671
Conf 32	112.142	105.593	104.602	-54.522	-54.282	-56.359	142.783	144.47	147.533	-64.188	-62.569	-59.073	-62.585	-61.743	-63.986
Conf 41	111.098	105.979	106.206	-54.505	-54.74	-57.909	142.923	145.005	149.931	-64.113	-63.674	-58.453	-63.21	-62.146	-57.348
Conf 60	110.33	107.805	104.436	-53.318	-55.343	-58.204	142.288	144.649	150.432	-60.603	-63.404	-55.387	-65.168	-61.43	-61.276
Conf 113	112.074	106.233	105.416	-54.601	-55.121	-59.165	142.993	145.238	150.268	-64.234	-62.798	-55.763	-62.682	-61.32	-63.42
Conf 125	117.608	113.263	98.991	-57.383	-55.648	-56.327	144.046	144.988	148.209	-59.346	-64.934	-60.375	-56.504	-59.381	-66.061
Conf 210	117.48	111.755	101.012	-58.732	-55.097	-55.867	147.959	144.73	147.016	-61.331	-64.647	-60.113	-56.162	-60.485	-64.755
Conf 272	110.461	106.778	105.916	-53.619	-55.658	-59.816	142.339	145.195	150.622	-60.723	-62.71	-56.736	-65.059	-62.096	-63.387
Conf 289	110.809	107.625	104.731	-54.831	-55.23	-57.398	142.211	145.961	149.115	-63.183	-63.28	-60.739	-66.28	-58.304	-63.893
Conf 305	110.073	106.246	107.886	-53.745	-55.172	-58.649	142.425	144.922	149.981	-60.604	-63.13	-57.389	-64.897	-62.538	-57.859
**max**	117.61	115.28	107.89	-53.32	-54.28	-55.87	148.53	145.96	151.15	-59.35	-62.57	-55.39	-56.16	-58.30	-56.58
**min**	110.07	105.59	98.99	-58.73	-55.93	-59.82	142.21	144.21	147.02	-64.29	-65.11	-60.95	-66.28	-62.54	-66.06
**range**	**98.99 – 117.61**			**-59.82 – -53.32**			**142.21 – 151.15**			**-65.11 – -55.39**			**-66.28 – -56.16**		

Figure SI14. **a.** The DFT-calculated $^3J_{HH}$ fit corresponding restraints extracted from 2D-TOCSY and psyche-2DJ experiments ($CDCl_3$, 500 and 950MHz). **b.** The distribution of dihedral angles on core delta residues in foldamer according to NMR-verified MCMM-DFT ensembles.

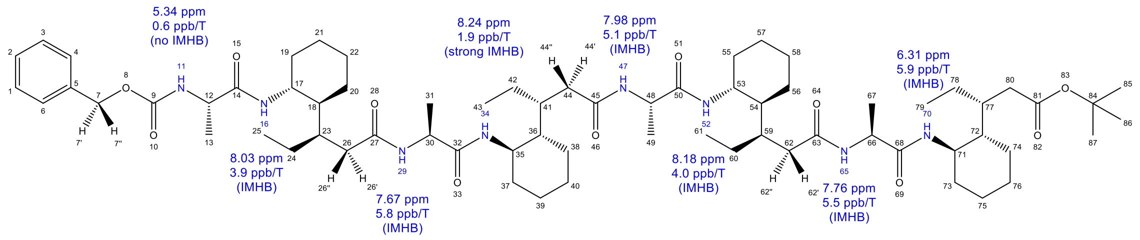

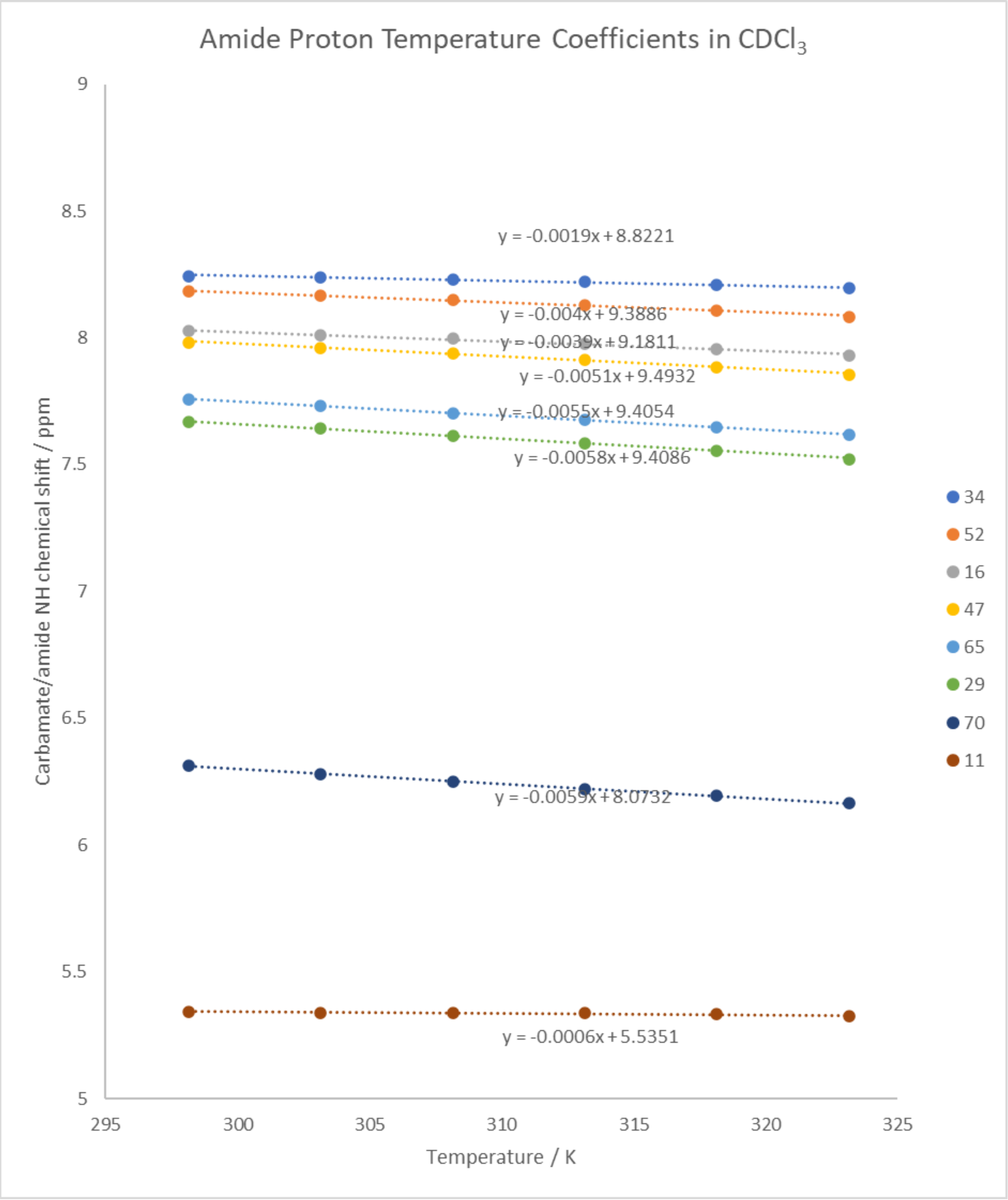

Figure SI15. NH temperature coefficients in foldamer (VT in $CDCl_3$, 500 MHz). No significant exchange was observed for the proton on carbamate.
